# Drug screens of *NGLY1* deficiency in worm and fly models reveal catecholamine, NRF2 and anti-inflammatory-pathway activation as potential clinical approaches

**DOI:** 10.1242/dmm.040576

**Published:** 2019-11-11

**Authors:** Sangeetha Iyer, Joshua D. Mast, Hillary Tsang, Tamy P. Rodriguez, Nina DiPrimio, Madeleine Prangley, Feba S. Sam, Zachary Parton, Ethan O. Perlstein

**Affiliations:** Perlara PBC, 2625 Alcatraz Ave, #435, Berkeley, CA 94705, USA

**Keywords:** *N*-glycanase 1 deficiency, *NGLY1*, *SKN-1*, *Pngl*, Congenital disorder of deglycosylation, Disease model, Aripiprazole

## Abstract

*N*-glycanase 1 (*NGLY1*) deficiency is an ultra-rare and complex monogenic glycosylation disorder that affects fewer than 40 patients globally. *NGLY1* deficiency has been studied in model organisms such as yeast, worms, flies and mice. Proteasomal and mitochondrial homeostasis gene networks are controlled by the evolutionarily conserved transcriptional regulator NRF1, whose activity requires deglycosylation by NGLY1. Hypersensitivity to the proteasome inhibitor bortezomib is a common phenotype observed in whole-animal and cellular models of *NGLY1* deficiency. Here, we describe unbiased phenotypic drug screens to identify FDA-approved drugs that are generally recognized as safe natural products, and novel chemical entities, that rescue growth and development of *NGLY1*-deficient worm and fly larvae treated with a toxic dose of bortezomib. We used image-based larval size and number assays for use in screens of a 2560-member drug-repurposing library and a 20,240-member lead-discovery library. A total of 91 validated hit compounds from primary invertebrate screens were tested in a human cell line in an NRF2 activity assay. NRF2 is a transcriptional regulator that regulates cellular redox homeostasis, and it can compensate for loss of NRF1. Plant-based polyphenols make up the largest class of hit compounds and NRF2 inducers. Catecholamines and catecholamine receptor activators make up the second largest class of hits. Steroidal and non-steroidal anti-inflammatory drugs make up the third largest class. Only one compound was active in all assays and species: the atypical antipsychotic and dopamine receptor agonist aripiprazole. Worm and fly models of *NGLY1* deficiency validate therapeutic rationales for activation of NRF2 and anti-inflammatory pathways based on results in mice and human cell models, and suggest a novel therapeutic rationale for boosting catecholamine levels and/or signaling in the brain.

## Abstract (precis)

**Summary:** Using worm and fly models of an ultra-rare congenital disorder of glycosylation, we performed repurposing screens and identified the FDA-approved drug aripiprazole as a clinical candidate.

## INTRODUCTION

*NGLY1* deficiency was the first congenital disorder of deglycosylation (CDDG) described in the biomedical literature (Lam et al., 2017; Enns et al., 2014). *NGLY1* deficiency has multi-organ presentation and clinical features in patients, such as global developmental delay, a complex hyperkinetic movement disorder, small body size, seizures and alacrimia. *NGLY1* is an ancient gene encoding a cytosolic enzyme called *N*-glycanase 1 – also referred to as PNGase – which catalyzes the hydrolysis and release of *N*-glycans from *N*-glycosylated proteins (Suzuki et al., 2016). NGLY1 is thought to function primarily in an evolutionarily conserved protein-surveillance and -disposal pathway called ERAD, or endoplasmic-reticulum-associated degradation (Suzuki et al., 2016). NGLY1 also regulates the activity of specific glycoproteins by using deglycosylation as a post-translational on/off switch. Cellular models of *NGLY1* deficiency have shown that the transcriptional regulator NRF1 is a specific deglycosylation target of NGLY1, and that knocking out *NGLY1* phenocopies knocking out *NRF1* (Tomlin et al., 2017). Only deglycosylated NRF1 can be proteolytically processed into the mature nuclear-active form. Once in the nucleus, NRF1 controls the expression of proteasomal subunit genes in response to protein-folding stress in mammalian cells (Radhakrishnan et al., 2014), worms (Lehrbach and Ruvkun, 2016) and flies (Grimberg et al., 2011). In flies, NGLY1 regulates the glycosylation status of the ortholog of a bone morphogenetic protein (BMP) signaling ligand (Galeone et al., 2017). Demonstrating the complexity of how loss-of-function mutations in the gene lead to pathophysiology in humans, NGLY1 regulates mitochondrial physiology in human and mouse fibroblasts and in worms through mechanisms that are still under investigation (Kong et al., 2018). Interestingly, mitophagy defects caused by loss of NRF1 function can be rescued by activation of the related transcriptional regulator NRF2, which controls the expression of genes involved in antioxidant and redox-stress responses (Yang et al., 2018).

In the 5 years since the publication of the first *NGLY1* deficiency diagnostic cohort of eight patients (Enns et al., 2014), multiple research groups have contributed to our understanding of disease-causing and loss-of-function mutations in the *NGLY1* gene and its orthologs by generating and characterizing small and large animal models as well as patient-derived cell models. From this marketplace of disease models, a common phenotype emerged: hypersensitivity to proteasome inhibition by bortezomib (Fenteany et al., 1995). In worms, hypersensitivity to bortezomib toxicity was observed in an otherwise normally developing *PNG-1*/*NGLY1*-null mutant, which has a half-maximal growth inhibitory concentration (IC_50_) several hundred times lower than wild-type worms (Lehrbach and Ruvkun, 2016). Using a chemically related proteasome inhibitor lacking the reactive boronic acid group, it was shown that mouse embryonic fibroblasts derived from *NGLY1*-knockout mice and *NGLY1*-knockdown human cell lines are several-fold more sensitive to carfilzomib toxicity compared to controls (Tomlin et al., 2017).

In an effort to phenotype and screen a homozygous loss-of-function *Pngl*/*NGLY1* fly modeling the patient-derived C-terminal premature stop codon allele R401X, we showed that fly *Pngl*^−/−^ homozygous mutant larvae are 25-fold more sensitive than heterozygotes to the toxic effects of bortezomib (Rodriguez et al., 2018). Another group reported that a *Pngl* RNAi-knockdown fly model of *NGLY1* deficiency has constitutively reduced expression of NRF1-dependent proteasomal subunit genes, consistent with findings of hypersensitivity to bortezomib toxicity in the other models (Owings et al., 2018). Loss of NGLY1 causes intolerance to bortezomib that is as evolutionarily conserved as the underlying NRF1-dependent proteasome bounce-back response because they go hand in hand. The prediction that has been confirmed so far in mammalian cells and in nematodes (Lehrbach et al., 2019) is that NGLY1 and its orthologs deglycosylate NRF1 and its orthologs. We reason that small-molecule suppressors of bortezomib will safely activate bypass pathways that rescue or compensate for loss of NGLY1 in a whole animal and will have a higher probability of exhibiting a favorable therapeutic index in mammals.

We used our *Pngl*^−/−^ fly model in a drug-repurposing screen to identify compounds that rescue larval developmental delay (Rodriguez et al., 2018). Homozygote flies fail to thrive in the absence of any exogenous stressor or enhancer such as bortezomib. There are two limitations of our previous screen. First, flies homozygous for a patient-derived nonsense allele are extremely sick and only one fly-specific validated hit was identified – the insect molting hormone 20-hydroxyecdysone. Second, flies homozygous for a patient-derived nonsense allele are further sickened by the organic solvent dimethyl sulfoxide (DMSO) in which all test compounds are solubilized, and all stock solutions prepared. Therefore, drug screens were conducted at the limit of detection and a high false-negative rate was due to under-dosing of test compounds.

Here, we addressed the shortcomings of the initial fly-only drug-repurposing campaign. First, we discovered that the fly *Pngl*^+/−^ heterozygote is two-fold more sensitive to bortezomib toxicity than wild-type animals, it tolerates DMSO, and it has no detectable developmental delay. We hypothesize that it will be easier for a small molecule to suppress larval developmental delay in bortezomib-treated healthy heterozygotes versus constitutively sick homozygotes because otherwise healthy heterozygotes tolerate higher levels DMSO and thus allow for all test compounds to be screened at a five-fold higher concentration. Second, we extended the bortezomib intolerance results of Lehrbach and Ruvkun (Lehrbach and Ruvkun, 2016) to a liquid-based, 384-well-plate quantitative worm larval growth and development assay for drug screening. We hypothesize that a two-species bortezomib-suppressor screen conducted in parallel would reveal hit compounds that target evolutionarily conserved disease modifiers and disease-modifying pathways. Third, we not only cross-tested all hit compounds from the primary worm screen in the fly assay, and vice versa, but we also cross-tested all hit compounds in two different worm assay paradigms (bortezomib treatment versus carfilzomib treatment), in two different fly assay paradigms (bortezomib treatment of heterozygotes versus homozygotes) and in a human cell NRF2 transcriptional activity reporter assay. This cross-validation scheme generated a short list of generic drugs and nutriceuticals in which we had the highest degree of confidence of reproducibility and clinical trial actionability. Finally, we screened not only the same repurposing library as described in Rodriguez et al. (2018) but also a ten-fold larger lead-discovery library. We hypothesize that, in some instances, the same known mechanisms of action of repurposable drugs will be targeted by novel chemical entities, which serve as starting points for lead optimization toward a best-in-class clinical candidate.

## RESULTS

There are currently no FDA-approved treatments for *NGLY1* deficiency despite the unmet medical need. Drug repurposing involves finding new uses for old drugs and is the shortest path to a therapy for ultra-rare disease communities with limited financial resources and few dedicated researchers (Pushpakom et al., 2019). We used a model-organism-based disease-modeling and phenotypic drug-screening approach, which is enabling precision medicine to bridge bench to bedside (Li et al., 2019).

### Developing high-throughput bortezomib-modifier assays for nematode and fly larvae

**Fig. 1.**
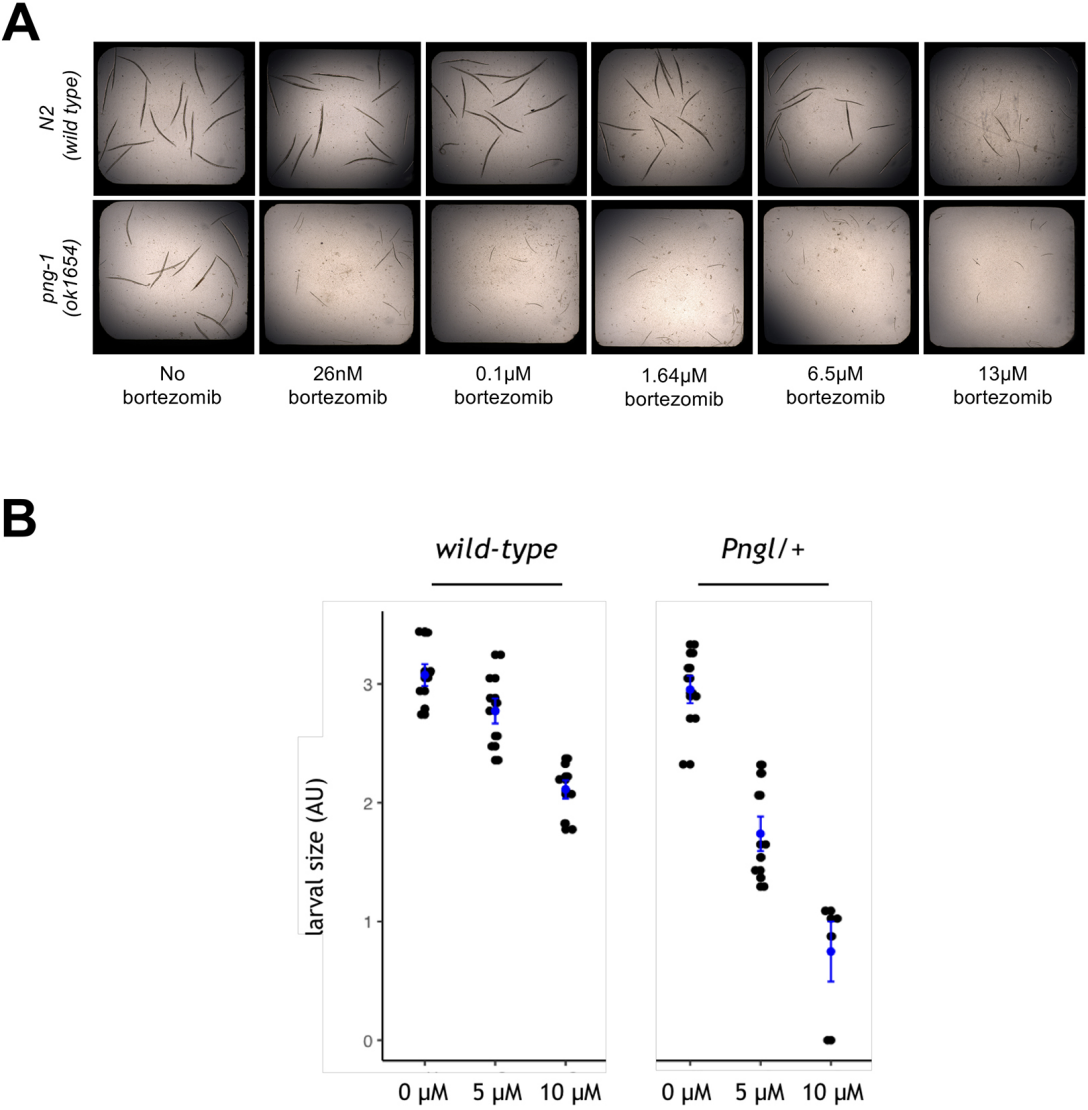
**Determining a half-maximal effective concentration (EC_50_) for bortezomib in *NGLY1*-deficient worms and flies.** (A) Wild-type N2 worms (top row) and *png-1* homozygous mutant worms (bottom row) were grown in liquid media in the presence of increasing concentrations of bortezomib (left to right). (B) Wild-type and *Pngl*^+/−^ heterozygous mutant fly larvae were grown on solid media in the presence of either 5 µM or 10 µM bortezomib. Fly larvae were imaged and their sizes were plotted in arbitrary units (AU).

The nematode ortholog of *NGLY1* is *PNG-1*. The strain used here is *ok1654*, which contains an 800-base-pair deletion in the *PNG-1* open reading frame, resulting in a null mutant. We confirmed that *ok1654* does not have an intrinsic growth defect but is markedly hypersensitive to bortezomib toxicity (Fig. 1A). Bortezomib exacerbates the proteasomal stress that *NGLY1*-deficient worms are already experiencing because of the concomitant loss of NRF1 activity, and results in an exaggerated disease phenotype, in this case toxicity in the form of early larval growth arrest (Lehrbach and Ruvkun, 2016). Because *png-1* homozygous mutant worms do not have a constitutive growth or developmental defect, we decided to use bortezomib to induce larval arrest and screen for compounds that restore normal growth and development as measured by the size and number of worms in each well of a 384-well plate. From the bortezomib dose-response data, we established that a worm *png-1*-null mutant is sensitive to bortezomib toxicity down to the low nanomolar range. After further assay optimization studies in which we tested additional doses between 100 nM and 1 µM, we selected 205 nM bortezomib as the concentration for the primary screens and for secondary retest and cross-test experiments to validate hit compounds because, at this dose, the mean size of *png-1* mutant worms is reduced by approximately 85% compared to untreated control worms (Fig. S1).

The ortholog of *NGLY1* in flies is *Pngl*. We previously developed a *Pngl*^−/−^ fly model based on a recurrent C-terminal nonsense allele found in NGLY1 patients, optimized a high-throughput larval size assay, and screened a 2560-member drug-repurposing library on fly *Pngl*^−/−^ larvae (Rodriguez et al., 2018). That effort culminated in a single validated hit compound, 20-hydroxyecdysone (20E). 20E is an insect-specific sterol-derived molting hormone. In order to identify clinically actionable hits, we increased the overall hit rate and robustness of fly screens. We generated bortezomib toxicity dose-response data for fly *Pngl*^+/−^ heterozygote larvae and determined that these animals are two-fold more sensitive than wild-type animals (Fig. 1B). After further assay optimization studies with doses between 5 µM and 10 µM, we selected 9 µM bortezomib as the concentration for the primary screens and for secondary retests and cross-tests to validate hit compounds.

### Worm repurposing screen and hit validation

**Fig. 2.**
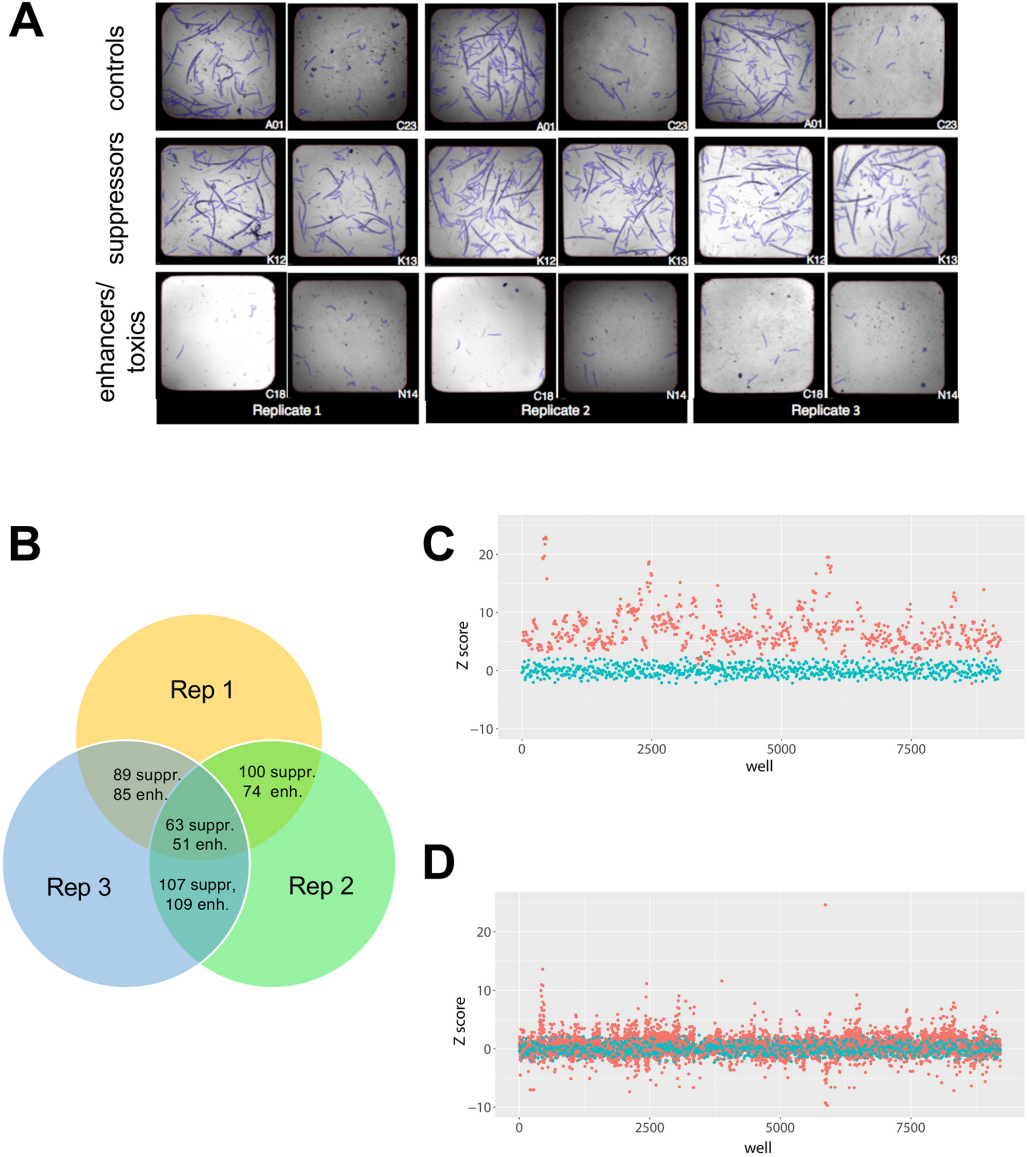
**2560-compound drug-repurposing screens of *png-1* homozygous mutant worms and *Pngl* heterozygous mutant flies.** (A) 15 L1 *png-1* mutant larvae were sorted into each well, and plates were incubated for 5 days at 20°C while shaking. Worm screen images of a representative positive control well (A01), a representative negative control well (C23), two presumptive suppressors (K12, K13), and two presumptive enhancers/toxic compounds (C18, N14). Worms were pseudo-colored blue during image processing and analysis. (B) Venn diagram of overlapping hits from three replicate screens. (C) Three fly first-instar *Pngl* heterozygous mutant larvae were sorted into each well, and plates were incubated for 3 days at room temperature. Z-score plot of three replicates of fly repurposing screen positive control (red circles) versus negative control (cyan circles) wells. (D) Z-score plot of three replicates of fly repurposing screen test compounds (red circles) versus negative (cyan circles) control wells.

In the presence of 205 nM bortezomib, *png-1*-null mutant worms arrest as L1 larvae while wild-type animals treated with the same dose of bortezomib develop normally into progeny-bearing adults. A worm repurposing hit is defined as a compound that rescued bortezomib-treated *png-1*-null worms such that their size and number were indistinguishable from control wells containing vehicle-treated *png-1*-null worms. All test compounds were screened at a final concentration of 25 μM. Images of a representative positive control well and a representative negative control well, and two examples of wells containing suppressors and enhancers/toxic compounds, are shown in Fig. 2A. The Venn diagram in Fig. 2B summarizes the overlap of screening positives between three independent replicates. A total of 63 suppressors have Z-scores greater than two in all three replicates. The hit rate for worm suppressors is 63/2560 or 2.5%. A total of 51 enhancers have Z-scores less than two in all three replicates. The hit rate for enhancers/toxic compounds is 51/2560 or 2%. Enhancers/toxic compounds were not further investigated in this study. In total, 60/63 worm suppressors were available for reorder as fresh powder stocks, retested in the primary bortezomib assay paradigm, and then scored in a secondary non-bortezomib assay paradigm.

Some compounds can directly inactivate bortezomib, leading to a false-positive result. For example, polyphenols and, in particular, polyphenols containing multiple catechols are known to form covalent adducts with bortezomib by boronate-catechol complexation (Glynn et al., 2015; Golden et al., 2009). In order to filter out those compounds, we tested the 60 worm suppressors on the worm *png-1*-null mutant treated with the chemically related proteasome inhibitor carfilzomib, which lacks the reactive boronic acid that renders bortezomib vulnerable to covalent attack. We established a carfilzomib dose-response curve for *png-1*-null mutant worms (Fig. S1). We directly compared these data to worms treated with 205 nM bortezomib in order to determine at which concentration we observe comparable larval growth arrest. We retested all 60 worm suppressors in the presence of 27.3 µM carfilzomib, meaning that carfilzomib is significantly less potent than bortezomib.

A total of 48/60 (80%) worm suppressors retested in the bortezomib assay paradigm. In total, 15/60 (25%) worm suppressors scored positively in the carfilzomib assay paradigm. Overall, 15/60 (25%) worm suppressors rescued in both worm assay paradigms, i.e. in the presence of either bortezomib or carfilzomib. Those 15 compounds are the worm repurposing hits (Table 1): aripiprazole, benserazide, ellagic acid, epicatechin monogallate, epigallocatechin-3-monogallate, ethylnorepinephrine, gossypetin, koparin, phenylbutazone, pomiferin, purpurogallin-4-carboxylic acid, quercetin, theaflavin monogallate, triamcinolone and 3,4-didesmethyl-5-deshydroxy-3′-ethoxyscleroin.

**Table 1.**
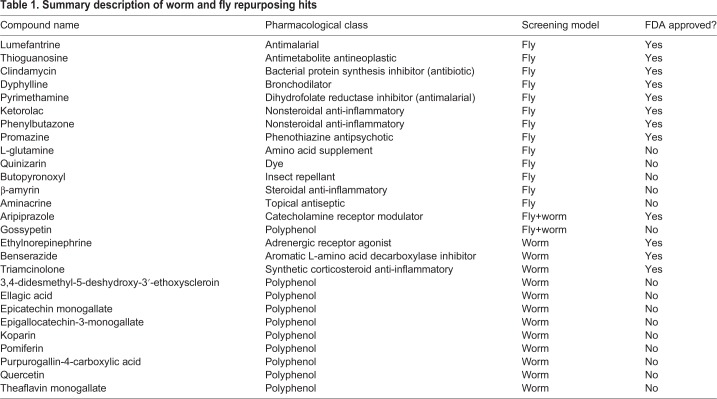
**Summary description of worm and fly repurposing hits**

A total of 10/15 (66%) worm repurposing hits are plant-based polyphenols. Epicatechin monogallate and epigallocatechin-3-monogallate are structural analogs, the latter containing an additional hydroxyl group. They are both abundant in tea leaves. The two plant flavonols quercetin and gossypetin are structural analogs, the latter containing an additional hydroxyl group. Quercetin is found in many foods and gossypetin is found in *Hibiscus* flowers. Aripiprazole, benserazide and ethylnorepinephrine act on catecholamine levels and/or signaling in the brain. Phenylbutazone is a decades-old first-generation non-steroidal anti-inflammatory drug, or NSAID, but is not currently approved and marketed in the United States. Triamcinolone is a synthetic corticosteroid and generic topical anti-inflammatory drug. These worm data suggest the existence of at least three mechanistic classes of suppressors: (1) polyphenolic antioxidants; (2) catecholamine pathway modulators; and (3) anti-inflammatories.

### Fly repurposing screen and hit validation

In the presence of 9 µM bortezomib, *Pngl*^+/−^ flies are developmentally delayed as first-instar larvae. A fly repurposing hit is defined as any compound that in triplicate rescued bortezomib-treated fly *Pngl^+/−^* larval growth and size such that they were indistinguishable from control wells containing DMSO-treated heterozygotes. All test compounds were screened at a final concentration of 32 μM. Separation between positive and negative control wells is shown in Fig. 2C. As expected, most test-compound wells do not affect bortezomib-treated heterozygotes as determined by quantifying larval area per well and remain close to the negative-control Z-score (Fig. 2D). We identified 31 fly suppressors that rescued larval size with Z-score greater than 2.5 in at least two out of three replicates, yielding a hit rate of 1.2%, or half the hit rate of the worm repurposing screen (Fig. S2) but 30 times the hit rate of the previously published fly *Pngl*^−/−^ drug repurposing screen (Rodriguez et al., 2018).

In order to eliminate suppressors that directly inactivate bortezomib or that fail to rescue in the absence of bortezomib-induced proteasomal stress, we retested suppressors in a 12-well Petri dish assay at 0, 10 µM, 50 µM or 100 µM of compound on either fly *Pngl*^+/−^ larvae in the presence of bortezomib or fly *Pngl*^−/−^ larvae without bortezomib. We could not retest suppressors in a carfilzomib assay paradigm because fly *Pngl*^+/−^ larvae were resistant to carfilzomib toxicity at the highest concentration tested. Note that we might not observe rescue at 100 µM in the DMSO-hypersensitive homozygous animals because of the increased amount of DMSO (0.2%) needed to test that concentration of compound. Although we did not confirm by chemical analysis, any compound that strongly rescued only in the bortezomib assay paradigm is likely a bortezomib inactivator. The top candidate bortezomib inactivators are tannic acid and gossypetin (Fig. S2). Gossypetin contains two catechols per molecule and tannic acid contains five catechols per molecule. These two compounds are the only overlapping primary screening hits between the worm and fly repurposing screens.

A total of 30/31 fly suppressors were reordered as fresh powder stocks (one compound failed to dissolve at 10 mM as stock solution). In total, 21/30 (70%) fly suppressors retested in the primary heterozygote assay paradigm, which is comparable to the retest rate of worm suppressors. A total of 20/30 (66%) fly suppressors scored positively in the secondary homozygote assay paradigm; 13/30 (43%) suppressors validated in both homozygote and heterozygote assay paradigms, which is higher than the worm cross-validation rate. Those 13 compounds are the fly repurposing hits (Table 1): aminacrine, aripiprazole, β-amyrin, butopyronoxyl, clindamycin, dyphylline, L-glutamine, ketorolac, lumefantrine, promazine, pyrimethamine, quinizarin and thioguanosine.

The fly repurposing hits span a range of mechanisms, including an overlap with the worm repurposing hits that fall into the three aforementioned pharmacological classes. Only one of the fly repurposing hits appears to be fly specific, i.e. the insect repellent butopyronoxyl. Ketorolac is a first-generation NSAID anti-inflammatory drug but is structurally distinct from the worm repurposing hit and NSAID phenylbutazone. β-amyrin is a triterpene natural product with anti-inflammatory effects. As stated above, aripiprazole is a catecholamine receptor modulator. The remaining fly repurposing hits appear to be mechanistic singletons and do not have obvious functional or structural analogs with worm repurposing hits. There are two antimalarial compounds, pyrimethamine and lumefantrine, and two DNA-damaging cancer drugs, aminacrine and thioguanosine. Dyphylline is a xanthine derivative with bronchodilator and vasodilator effects. Clindamycin is a lincosamide antibiotic used for a range of bacterial infections.

### Cross-validation of worm and fly repurposing hits

In order to quantify the overlap between worm and fly repurposing hits, we selected 11 worm repurposing hits for cross-testing in both fly assay paradigms, and 19 fly repurposing hits for cross-testing in both worm assay paradigms. The fraction of worm repurposing hits that rescue in flies is higher than the fraction of fly repurposing hits that rescue in worms. All [11/11 (100%)] worm repurposing hits scored positively in the fly heterozygote assay paradigm. Six (55%) of these compounds cross-validated in both fly assay paradigms. There may be several reasons why a compound scored positively in a secondary retest in one organism but was not a hit in the primary screen in the same organism. This is especially true in flies, because fly primary screens have more variance than primary screens in worms due to fewer animals per well, greater complexity of food source and increased developmental stochasticity.

**Fig. 3.**
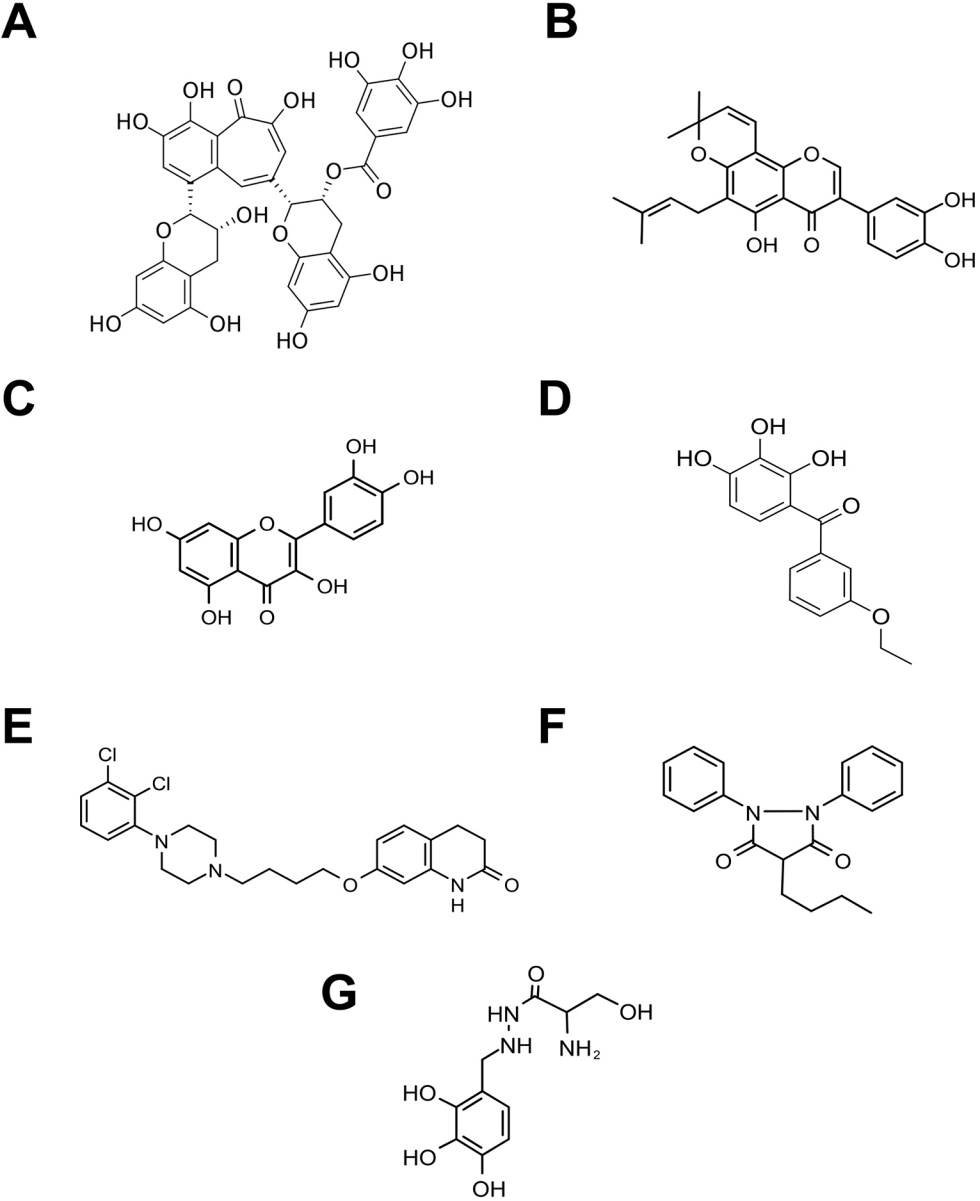
**Chemical structures of cross-validated drug repurposing hits.** (A) Theaflavin monogallate; (B) pomiferin; (C) quercetin; (D) 3,4-didesmethyl-5-deshydroxy-3′-ethoxyscleroin; (E) aripiprazole; (F) phenylbutazone; (G) benserazide.

However, 3/19 (16%) fly repurposing hits scored positively in the worm bortezomib paradigm; 3/19 (16%) fly repurposing hits scored positively in the worm carfilzomib paradigm. Only 1/19 (5%) fly repurposing hits cross-validated in both worm assay paradigms: aripiprazole. Of the 30 repurposing hits that were cross-tested, seven compounds (23%) are active in both worm assay paradigms and in both fly assay paradigms: aripiprazole, benserazide, phenylbutazone, pomiferin, quercetin, theaflavin monogallate and 3,4-didesmethyl-5-deshydroxy-3'-ethoxyscleroin. Their chemical structures are shown in Fig. 3.

**Fig. 4.**
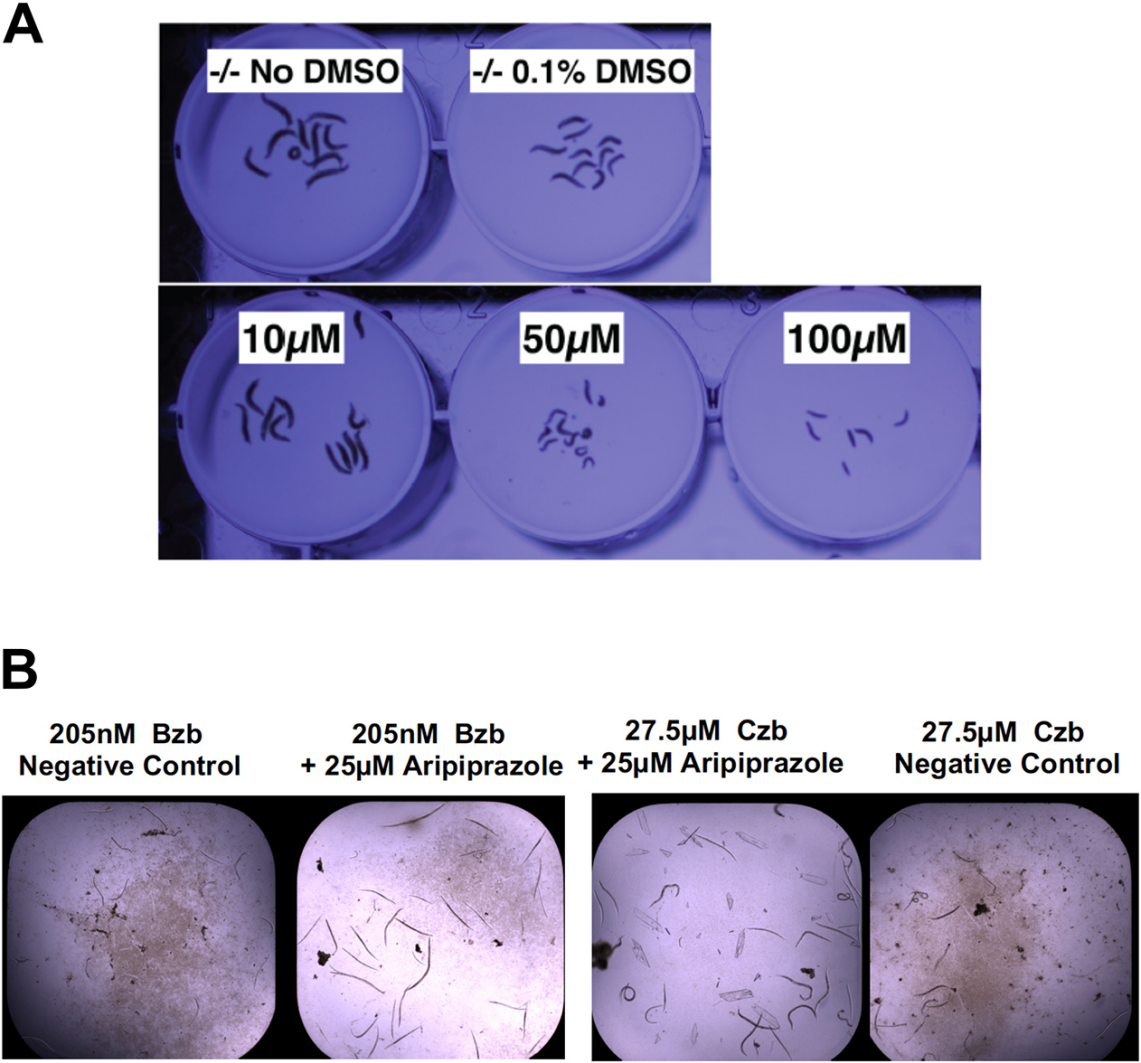
**Aripiprazole worm and fly cross-validation data.** (A) Fly *Pngl*^−/−^ homozygote larvae treated with 10 µM, 50 µM and 100 µM aripiprazole compared to untreated controls. (B) Worm *png-1* homozygote larvae treated with 25 µM aripiprazole in the presence of 205 nM bortezomib (Bzb) or 27.5 µM carfilzomib (Czb).

Aripiprazole is an atypical antipsychotic drug with broad polypharmacology at catecholamine receptors in the brain, including agonist effects on dopamine receptors. It is approved for use alone or in combination in adults and children for the treatment of symptoms of many CNS diseases, including schizophrenia, autism spectrum disorder and bipolar disorder (Shapiro et al., 2003; Kikuchi et al., 1995). Aripiprazole has been available in generic form since 2015. As shown in Table 1, aripriprazole is the only FDA-approved drug independently discovered in both worm and fly drug repurposing screens, and cross-validated in all four secondary retesting paradigms. Aripiprazole fully rescued fly *Pngl^−/−^* homozygous mutant larvae at 10 µM with dose-limiting toxicity at 50 µM at 100 µM (Fig. 4A). At the same time, 25 µM aripiprazole fully rescued growth and development of worm *png-1* homozygous mutant larvae treated with either bortezomib or carfilzomib (Fig. 4B).

Benserazide is an aromatic L-amino-acid decarboxylase inhibitor that has been co-administered with levodopa (L-Dopa) for decades to boost dopamine levels in the brain in the treatment of Parkinson disease. As mentioned above, phenylbutazone is a decades-old NSAID and quercetin is a plant flavonol with complex pharmacology, including anti-inflammatory effects. Theaflavin monogallate is a polyphenol found in black tea leaves. Pomiferin is a prenylated isoflavone found in osage orange trees.

### Drug discovery screens and cross-validation of novel chemotypes

**Fig. 5.**
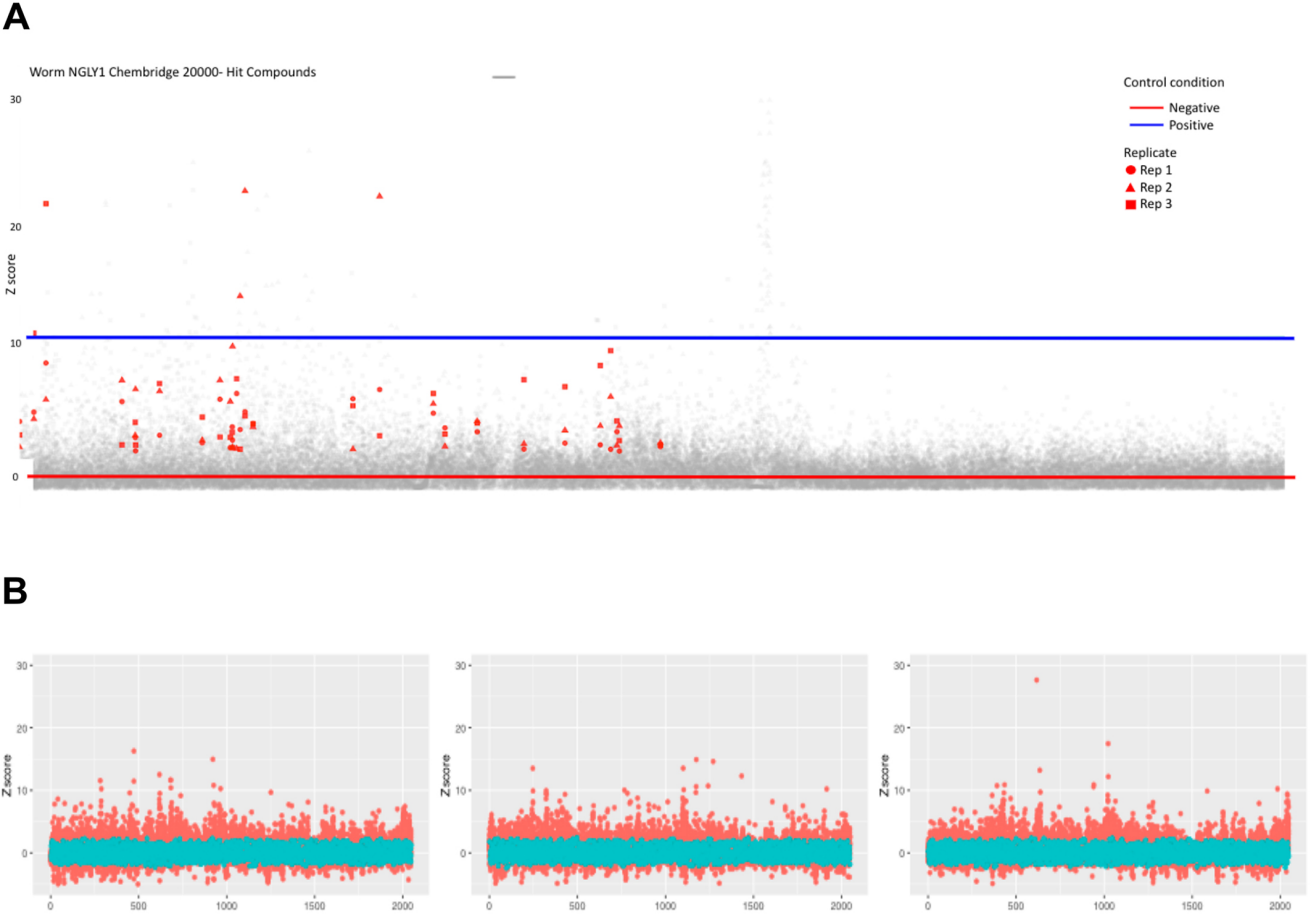
**20,240-compound novel lead-discovery screens of *png-1* homozygous mutant worms and *Pngl^+/−^* heterozygous mutant flies.** (A) Worm screen Z-score plot of 20,240 test compounds in triplicate. Replicate 1 is shown as red circles. Replicate 2 is shown as red triangles. Replicate 3 is shown as red squares. The mean negative control Z-score is indicated by the red line. The mean positive control Z-score is indicated by the blue line. (B) Fly screen Z-score plot of 20,240 test compounds in triplicate. Replicate 1 is the left panel. Replicate 2 is the center panel. Replicate 3 is the right panel.

Using the same screening conditions and hit-calling analysis as the worm repurposing screen, we scaled up efforts with worm *png-1*-null mutant larvae and a 20,240-member lead-discovery library. We identified 28 novel worm suppressors and six novel worm enhancers/toxic compounds with a combined hit rate of 0.143% (Fig. 5A; Fig. S3). Enhancers/toxic compounds were not further investigated in this study. In total, 12/28 (43%) novel worm suppressors retested in the bortezomib assay paradigm; 10/28 (36%) novel worm suppressors scored positively in the carfilzomib assay paradigm. A total of five novel worm suppressors (18%) validated in both paradigms, which is comparable to the cross-validation rate observed for worm repurposing hits. Consistent with the results of the repurposing screens, the fraction of novel worm suppressors that are active in flies is higher than the fraction of novel fly suppressors that are active in worms. A total of 4/5 (80%) of novel worm suppressors cross-validated in both fly secondary assays (Table 2). These hits represent novel chemotypes and are starting points for mechanism-of-action studies in human cellular models of *NGLY1* deficiency.

**Table 2.**
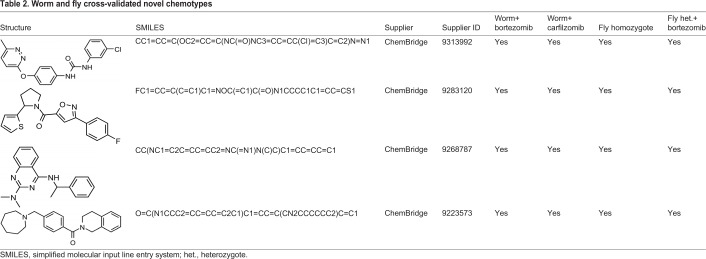
**Worm and fly cross-validated novel chemotypes**

Using the same screening conditions and hit-calling analysis for the fly repurposing screen, we scaled up efforts with fly *Pngl*^+/−^ larvae and a 20,240-member lead-discovery library. We identified 16 novel fly suppressors, resulting in a hit rate of 0.07% (Fig. 5B; Fig. S4). In total, 15/16 novel fly suppressors were reordered as fresh powder stocks; 10/15 (67%) novel fly suppressors retested in the primary heterozygote assay paradigm; and 13/15 (87%) novel fly suppressors scored positively in the secondary homozygote assay paradigm. A total of ten novel fly suppressors (67%) validated in both paradigms. Of the 13 novel fly suppressors, 12 were retested in both worm assay paradigms. Surprisingly, none of the novel fly suppressors scored positively in the bortezomib assay paradigm, but 8/12 (66%) compounds scored positively in the worm carfilzomib assay paradigm.

That result is similar to what we observed in the drug-repurposing cross-validation experiments, namely the likelihood of a worm novel hit cross-validating in flies is higher than the likelihood of a fly novel hit cross-validating in worms. A combination of evolutionary divergence and experimental assay differences between worms and flies likely explain the failure of a given compound to cross-validate in both species. For example, the known NRF2 activator sulforaphane at 50 µM did not rescue in either worm assay, potentially due to compound solubility differences in worm versus fly media, but it did score positively in the fly *Pngl*^−/−^ homozygote assay paradigm, rescuing this mutant to 100% of the untreated homozygote positive control (Fig. S5).

In the most striking example of overlap between the repurposing library and discovery library, we identified two novel fly suppressors that are structural analogs of pyrimethamine, sharing a diaminopteridine group present in folic acid analog inhibitors of DNA synthesis enzymes, such as methotrexate. Both pyrimethamine and one of the novel fly suppressors rescue homozygotes to near 100% of the heterozygote control. A second novel fly suppressor also rescues homozygotes to near 100% of the heterozygote control. The chemical structures of those and all drug discovery hits are summarized in the supplementary material.

### Keap1-NRF2 activation assay in human cells

Although cross-validation of hits in worms and flies indicates that the target and mechanism of action of the test compound are conserved between these two species, it does not guarantee that the said target and said mechanism of action are also conserved in humans. In an attempt to winnow down the list of hits to at least one FDA-approved clinical candidate for *NGLY1* deficiency, a total of 91 repurposing hits and novel suppressors from both worm and fly screens were cross-tested in a Keap1-NRF2 activation assay in the U2OS osteosarcoma cell line. A total of 7/91 (8%) compounds are active with a half-maximal effective concentration (EC_50_) between 2-30 µM (Table 3), or 10- to 100-fold less potent than the positive-control NRF2 activators, e.g. sulforophane. The remaining 84 compounds have EC_50_ values above 50 µM and were not considered further for lack of potency. Listed here in order of percentage of maximal response normalized to the positive control CDDO methyl ester: aripiprazole (78%)=pyrogallin (78%)>fisetin (59%)>purpurogallin-4-carboxylic acid (53%)=3-methoxycatechol (50%)>rhamnetin (43%)>pyrimethamine (27%). Of these seven, only fisetin has been previously shown to activate NRF2 target gene expression (Smirnova et al., 2011). To increase confidence in the assay, we also tested three additional known NRF2 inducers: sulforaphane, omaveloxolone and dimethyl fumarate. None of the novel chemotypes in Table 2 are NRF2 inducers.

**Table 3.**
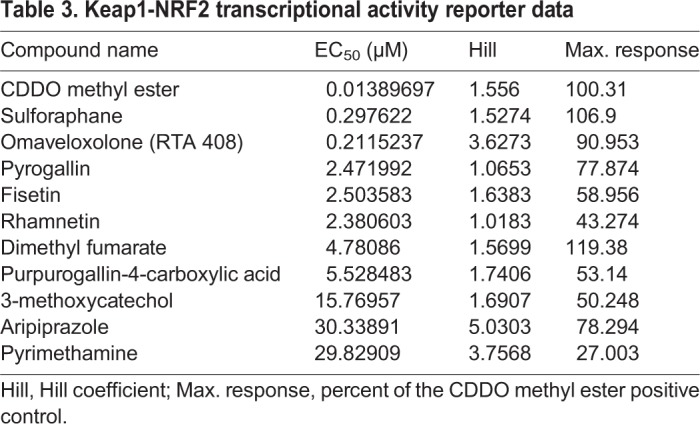
**Keap1-NRF2 transcriptional activity reporter data**

## DISCUSSION

In summary, we demonstrated the therapeutic relevance of unbiased phenotypic drug screens of worm and fly models of *NGLY1* deficiency. We addressed the major shortcoming of our previous drug screening effort by increasing the hit rate and reproducibility with the more permissive fly *Pngl*^+/−^ heterozygote assay as the primary screen followed by a secondary retest in the more restrictive fly *Pngl*^−/−^ homozygote assay paradigm, which selected for the subset of hit compounds that specifically rescues loss of NGLY1. We addressed the other significant shortcoming of our previous drug screening effort by including worms as a second species in the primary screening stage, which increased the total number and mechanistic diversity of hits. Additionally, by including human cells as a third species in the hit validation stage, we selected for hit compounds that act on conserved targets and pathways. We identified three clinic-ready therapeutic approaches by focusing on the subset of hit compounds that rescue larval growth and development in both worms and flies and in the presence and absence of bortezomib: (1) NRF2 activators/inducers; (2) catecholamine-boosting drugs; and (3) anti-inflammatory drugs. Only one compound was found to be active in all three species (worm, fly and human cells) and in all assay paradigms (plus bortezomib, plus carfilzomib, and NRF2 reporter): the atypical antipsychotic aripiprazole.

Before discussing why we observed those three pharmacological classes and the evidence that supports their testing in clinical trials, we consider reasons why any given hit compound might be active in one or a combination of the species and assay paradigms tested herein. Some reasons have to do with compound stability, compound solubility, bioavailability, drug metabolism and pharmacokinetics, not to mention the differences between worm screens versus fly screens. The following is by no means an exhaustive list of salient factors. Worm screens were all performed in simple liquid media that is a buffered salt solution plus essential nutrients and contains bacteria as a food source. By contrast, the fly screens were performed in a nutrient-rich solid media (‘fly food’) with a molasses base. The worm screen is a 6-day assay that spans all stages of the worm life cycle, while the fly screen is a 3-day assay that only spans the larval stages of fly development. The worm screen was performed with 205 nM bortezomib while the fly screen was performed with 9 µM bortezomib. Primary drug screens with flies involve as few as three animals per test well, while primary drug screens with worms involve a minimum of 15 animals per test well. Secondary retest assays involved multi-well plates with larger well volumes and more animals per well than primary screening conditions, which could explain instances of false negatives. For example, the four novel chemotypes described in Table 2 rescued fly *Pngl^+/−^* heterozygous larvae in the secondary assay format but not in the primary drug screen.

Other reasons why a hit compound is active in one species but not the other may have to do with pharmacodynamics; that is, the drug targets are not evolutionarily conserved or the drug-binding sites of shared drug targets are too divergent between worm and fly orthologs. Resolving which pharmacokinetic and pharmacodynamic variables and their relative contributions explain the failure of a hit compound in any given assay or species paradigm is a very important research question that is beyond the scope of the present study. However, what we can conclude from this study is that NRF2, anti-inflammatory and catecholamine pathways appear to be evolutionarily conserved between worms, flies and human cells.

Indeed, NRF2 pathway activators and anti-inflammatory drugs have been proposed as clinical approaches to address the underlying defects in mitochondrial physiology observed in *NGLY1*-deficient cells, specifically the defect in mitophagy and excessive mitochondrial fragmentation (Yang et al., 2018). In addition to its more-well-studied role as a transcriptional regulator of the proteasome bounce-back response, NRF1 also controls the expression of mitochondrial homeostasis and mitophagy genes not only in human cells but also in worms (Paek et al., 2012) and flies (Tsakiri et al., 2013). A unifying theory of *NGLY1* deficiency is that disease phenotypes are primary or secondary consequences of the loss of conserved NRF1-dependent gene expression programs in cell types and tissues that are vulnerable to proteotoxic, oxidative or redox stress. A recent *n*-of-1 clinical study of an NGLY1-CDDG patient who died of complications due to adrenal insufficiency suggests that steroidogenic secretory tissues fit the criteria of a vulnerable cell type (van Keulen et al., 2019). The *Pngl*^−/−^ fly has defects in its physiologically analogous neuroendocrine tissues, which fail to produce and secrete the cholesterol-derived hormone 20-hydroxyecdysone (Rodriguez et al., 2018). That said, there is only a single ortholog of both NRF1 and NRF2 in worms and flies (*SKN-1* and *cnc*, respectively), so the functions of worm *SKN-1* and fly *cnc* may not recapitulate all functionality of mammalian NRF1 and NRF2.

Why do anti-inflammatory drugs rescue worm and fly models of *NGLY1* deficiency? Defects in mitophagy lead to the release of mitochondrial genomic DNA and mitochondrial-encoded RNA from fragmented and damaged mitochondria into the cytoplasm, which drives constitutive cGAS-STING and related innate immunity responses in *NGLY1*^−/−^ mice and cell lines (Yang et al., 2018). These innate immunity pathways are conserved in flies (Martin et al., 2018) and worms (Wu et al., 2014). We speculate that mitochondrial fragmentation and mitophagy defects are present in cells and tissues of *png-1*-null mutant worms and *Pngl*^−/−^ mutant flies. Mitophagy defects and oxidative stress were observed in *SKN-1* mutant worms (Palikaras et al., 2015). We therefore hypothesize that the steroidal and nonsteroidal anti-inflammatory drugs that rescue worm and fly models of *NGLY1* deficiency, e.g. phenylbutazone, are suppressing basally elevated innate immune responses. There is evidence for this inflammation hypothesis in the recent NGLY1 literature. *SKN-1* is required for pathogen resistance in worms (Papp et al., 2012). Transcriptional profiling of *Pngl*-knockdown flies revealed that genes involved in the innate immune response are upregulated (Owings et al., 2018). The expression of interferon genes is constitutively upregulated in *Ngly1^−/−^* murine embryonic fibroblasts (Yang et al., 2018). It is tempting to speculate further that hyperactive innate immunity responses contribute to larval growth arrest and developmental delay in *NGLY1*-deficient animals, and that these anti-inflammatory responses are dampened by inhibitors of DNA synthesis, specifically purine biosynthetic enzymes targeted by the fly repurposing hits thioguanosine and the dihydrofolate-reductase-like inhibitor pyrimethamine (Dziekan et al., 2019), and possibly the diaminopteridine-containing novel lead compounds that resemble pyrimethamine. Even more so because the same mechanism of action appears to have been revealed by parallel repurposing and discovery screens.

At this time, the use of catecholamine boosters are supported by measurements of reduced catecholamine precursors in the cerebral spinal fluid of NGLY1-CDDG patients (Lam et al., 2017), as well as the fact that an adult and wheelchair-bound NGLY1-CDDG patient has received the dopamine precursor L-Dopa. Interestingly, there is evidence for dopamine insufficiency from a *Pngl* RNAi-knockdown fly model of *NGLY1* deficiency, where the expression of genes involved in dopamine biosynthesis (e.g. tyrosine hydroxylase and the dopamine transporter DAT) are constitutively downregulated (Owings et al., 2018). Future natural history studies of *NGLY1* deficiency should focus on catecholamine insufficiency as a potential driver or axis of pathophysiology. The effects of aripiprazole in worms depends on catecholamine pathway genes (Osuna-Lugue et al., 2018). In flies, aripiprazole was shown to reduce the levels of an aggregated polyglutamine-expanded mutant protein in a model of Machado-Joseph disease, or spinocerebellar ataxia type 3 (Costa et al., 2016). The mechanism of rescue appears to rely on activation of proteotoxic and antioxidant stress responses. It is tempting to conjecture that aripiprazole achieved its rescue effects in the Machado-Joseph fly model and in our *NGLY1*-deficiency fly model by activating both NRF2 and catecholamine pathways, and, further, that the unique polypharmacology of aripiprazole combines catecholamine pathway activation and NRF2 pathway activation.

## MATERIALS AND METHODS

### Strains and compound libraries

The *png-1* deletion mutant *ok1654* was previously described (Lehrbach and Ruvkun, 2016) and is available from the CGC Stock Center (strain ID: RB1452). The nonsense allele *Pngl* fly was previously described (Rodriguez et al., 2018) and is available from the Bloomington Stock Center. The 2560-compound Microsource Spectrum Collection was available for purchase before the distributor went out of business. The 20,240-compound lead-discovery library was purchased from Chembridge Corporation. All compounds were dissolved in DMSO and stored at −80°C in Labcyte-compatible LDV plates until use.

### High-throughput larval growth assays in worms

Positive controls for all screens are *png-1* mutants+DMSO (vehicle control) and the negative controls are *png-1* mutants+DMSO+205 nM bortezomib. Both drug libraries were screened in triplicate with controls on each 384-well drug-screening plate. The final DMSO concentration in each well was 0.27%. Worms tolerated up to 1% DMSO before showing signs of toxicity. Using the Echo550 liquid handler (Labcyte Inc.), bortezomib was acoustically dispensed into the destination plates the day before the worm larvae sort. In total, 5 µl of HB101 bacteria were dispensed into 384-well plates containing S-medium (prepared in-house) in each well. Using the BioSorter (Union Biometrica), 15 L1 *png-1* mutant larvae were sorted into each well, and plates were incubated for 5 days at 20°C while shaking. After 5 days of benchtop incubation, 15 µl of 8 mM sodium azide was added to each well to immobilize worms prior to imaging in a custom worm imager. Finally, automated image processing was run on each plate.

### High-throughput fly larval growth assays

Drug screens of *Pngl* heterozygous flies were conducted in the presence of 9 µM bortezomib. The negative controls in the screen are *Pngl* heterozygous flies+DMSO+bortezomib and the positive controls are *Pngl* heterozygous flies+DMSO. Both drug libraries were screened in triplicate. The final DMSO concentration in each well was 0.1%. Flies tolerated up to 1% DMSO before showing signs of toxicity. Using the Echo550 liquid handler (Labcyte Inc.), test compound was acoustically dispensed and standard fly food media (molasses, agar, yeast, propionic acid) lacking cornmeal, but carrying 0.025% Bromophenol Blue, was dispensed using a Multi-Flo (Bio-Tek Instruments) into each well of a 96-well plate for drug screens or a 12-well plate for hit retests. Then, the BioSorter was used to dispense fly *Pngl* heterozygous larvae, three per well. At 3 days post-incubation with the compounds, the plates were scored for larval size rescue in a custom fly imager.

### High-throughput drug screening and analysis

We used the statistical program R (https://www.r-project.org/) in all Z-score calculations described herein. Hit compounds rescued worm development to adulthood, i.e. increased the number of worms (total area taken up by worms) in the well. An increase in the total area occupied by worms over the course of the 5-day liquid growth assay is caused by the 15 L1 worm larvae growing in size to adults as well as by these adults producing progeny. Therefore, the output of the image processing was worm area per well. Each plate contained 32 wells of positive controls (worms raised in the absence of bortezomib) and 32 wells of negative controls (worms raised in the presence of bortezomib). The average area occupied by worms in positive and negative control wells as well standard deviation (s.d.) of control wells were calculated. Outlier elimination was performed by identifying those wells that were greater than 1.5 times the interquartile range (1.5× IQR). Z-scores for test wells were calculated by normalizing by mean area s.d. of negative control wells. For each replicate, a test well that had a Z-score ≥2 was counted as a hit. Quality control was performed to ensure that the control well grew as expected, the worm *png-1* mutants were sensitive to bortezomib as expected, there were no obvious plate effects, and dispense errors were eliminated. Because image artifacts can often confound area measurements, we also manually verified each well by visual analysis after the unbiased quantitative analysis to make sure no false positives were counted as true hits. We count wells as hits that have a Z-score greater than two across all three replicates of the screen and were free of image artifacts.

Fly screening conditions were previously described (Rodriguez et al., 2018). Briefly, we imaged the plates using a custom fly imager and analysis tool to determine the area that the larvae occupy per well. Then, we counted the number of larvae per well to normalize the total-area-per-well data and identified suppressors of the phenotype. We also manually scored the wells for contaminants that may give false positives. Similar to the statistical analysis for the nematode assay, test wells were assigned Z-scores relative to mean negative controls. Any well that had a Z-score >2.5 in at least two out of three replicates was counted as a hit.

### Keap1-NRF2 activation luciferase reporter assay in U2OS cells

We tested compounds using the PathHunter^®^ eXpress Keap1-NRF2 Nuclear Translocation Assay (DiscoverX, San Diego). Briefly, PathHunter cells are plated and incubated for 24 h at 37°C. Ten µl of test compound was added and cells were incubated with compound for 6 h at room temperature. Working detection reagent solution was added and plates were incubated for 60 min at room temperature. Chemiluminescence signal was read by a SpectraMax M3.

## Supplementary Material

Supplementary information
