# Supplementary material for: Drug screens of NGLY1 deficiency in worm and fly models reveal catecholamine, NRF2 and anti-inflammatory-pathway activation as potential clinical approaches
Source: Dis Model Mech. 2019 Nov 11;12(11):dmm040576. doi: 10.1242/dmm.040576 (PMC6899034; doi:10.1242/dmm.040576)
Supplement: Supplementary information [file dmm-12-040576-s1.pdf]

Supplemental Material. Summary of worm and fly cross-validation and secondary assays for worm and fly hit compounds from the Microsource Spectrum library (drug repurposing) and a Chembridge diversity library (drug discovery).

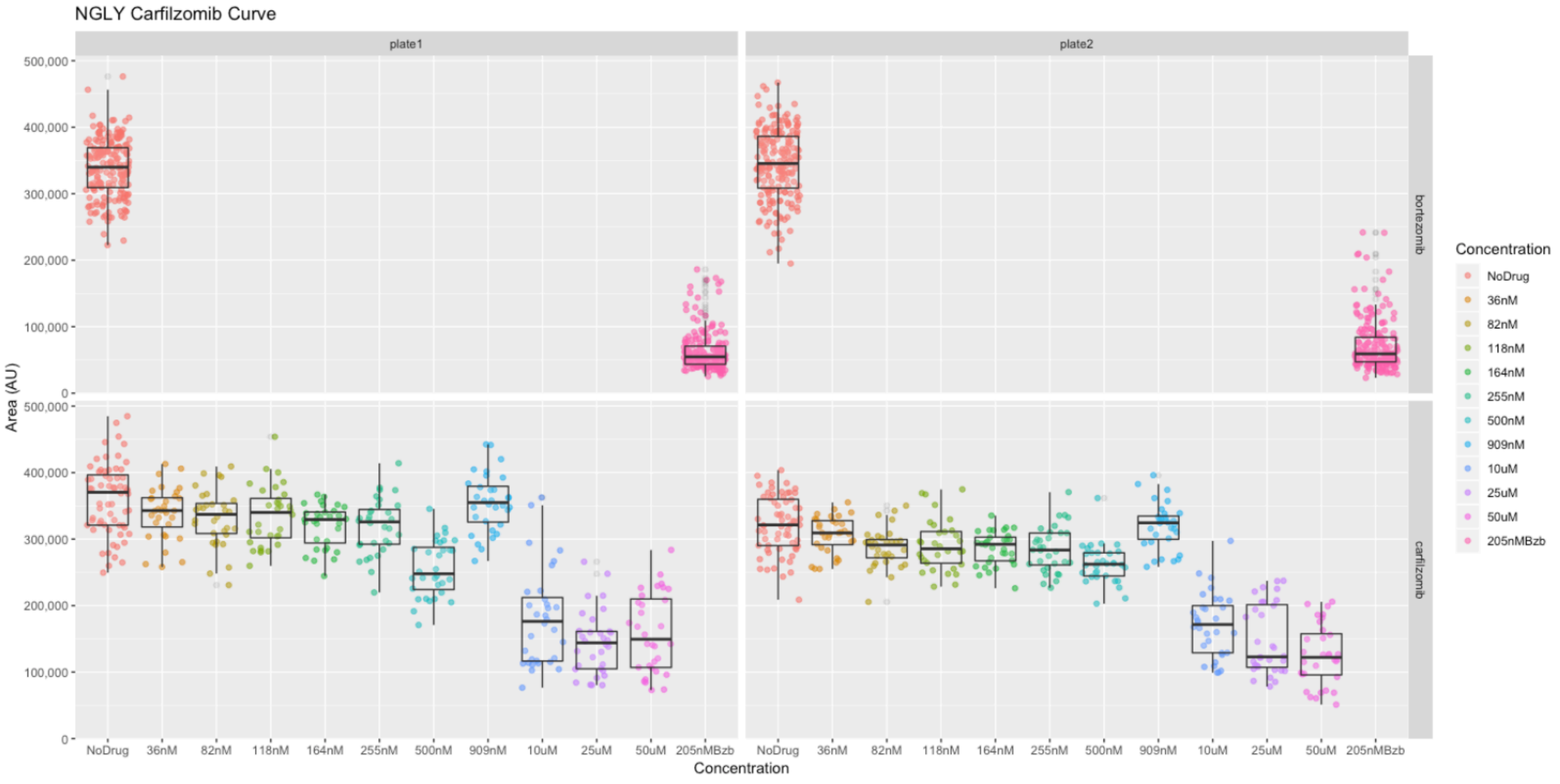

Figure S1. Carfilzomib dose-response experiment in *png-1* homozygous worms. Results from two technical replicates are shown. The y-axis is well area occupied by worms.As a comparison, the top panels show *png-1* homozygous worms treated with 205nM bortezomib. The bottom panels show *png-1* homozygous worms treated with ascending doses of carfilzomib.

Disease Models & Mechanisms • Supplementary information

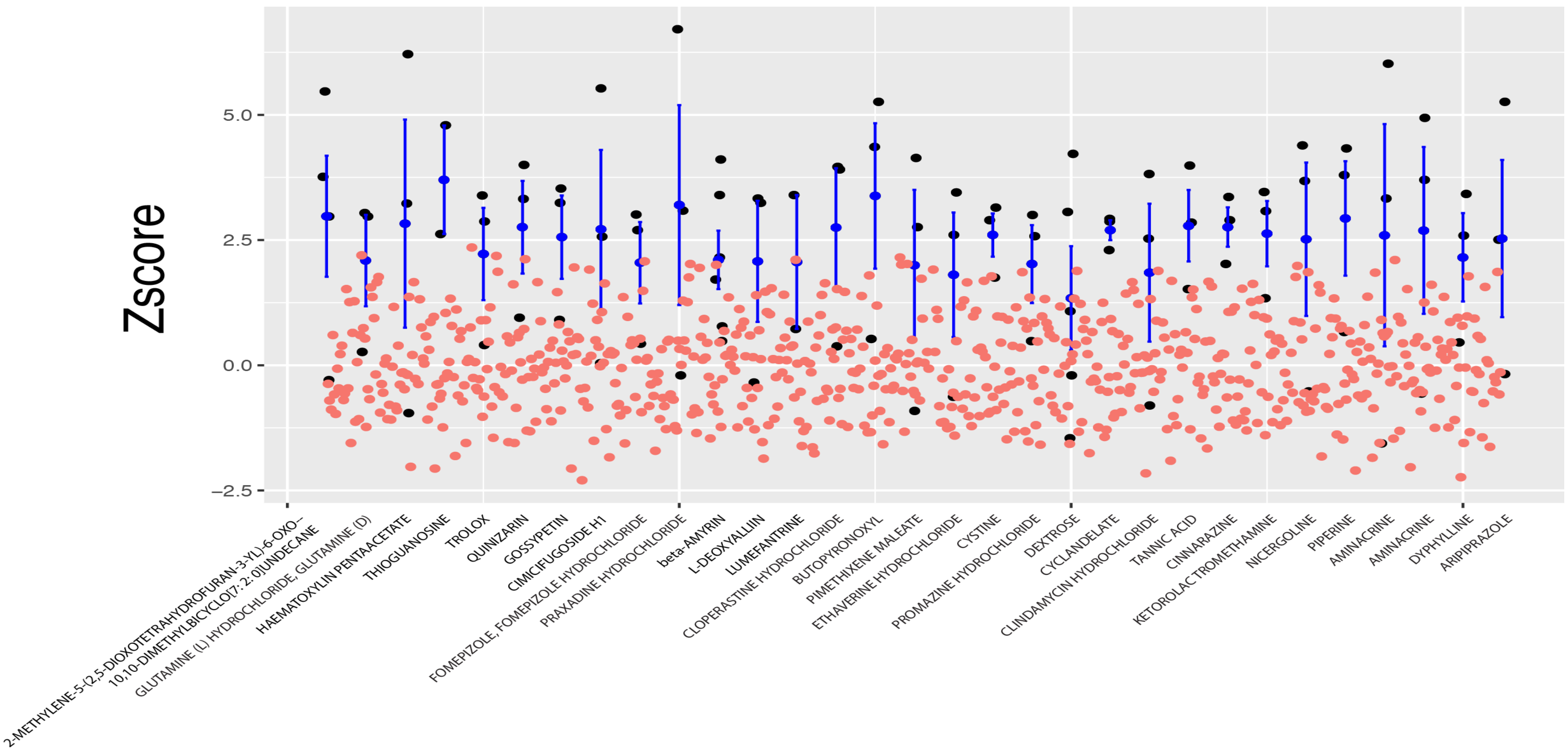

Figure S2. Z-score plot of 31 Microsource Spectrum repurposing hits from the *Pngl* $^{+/-}$ fly primary screen. Red circles denote negative controls, black circles denote hit compounds wells, and blue circles correspond to the average of replicates of hit compounds.

Disease Models & Mechanisms • Supplementary information

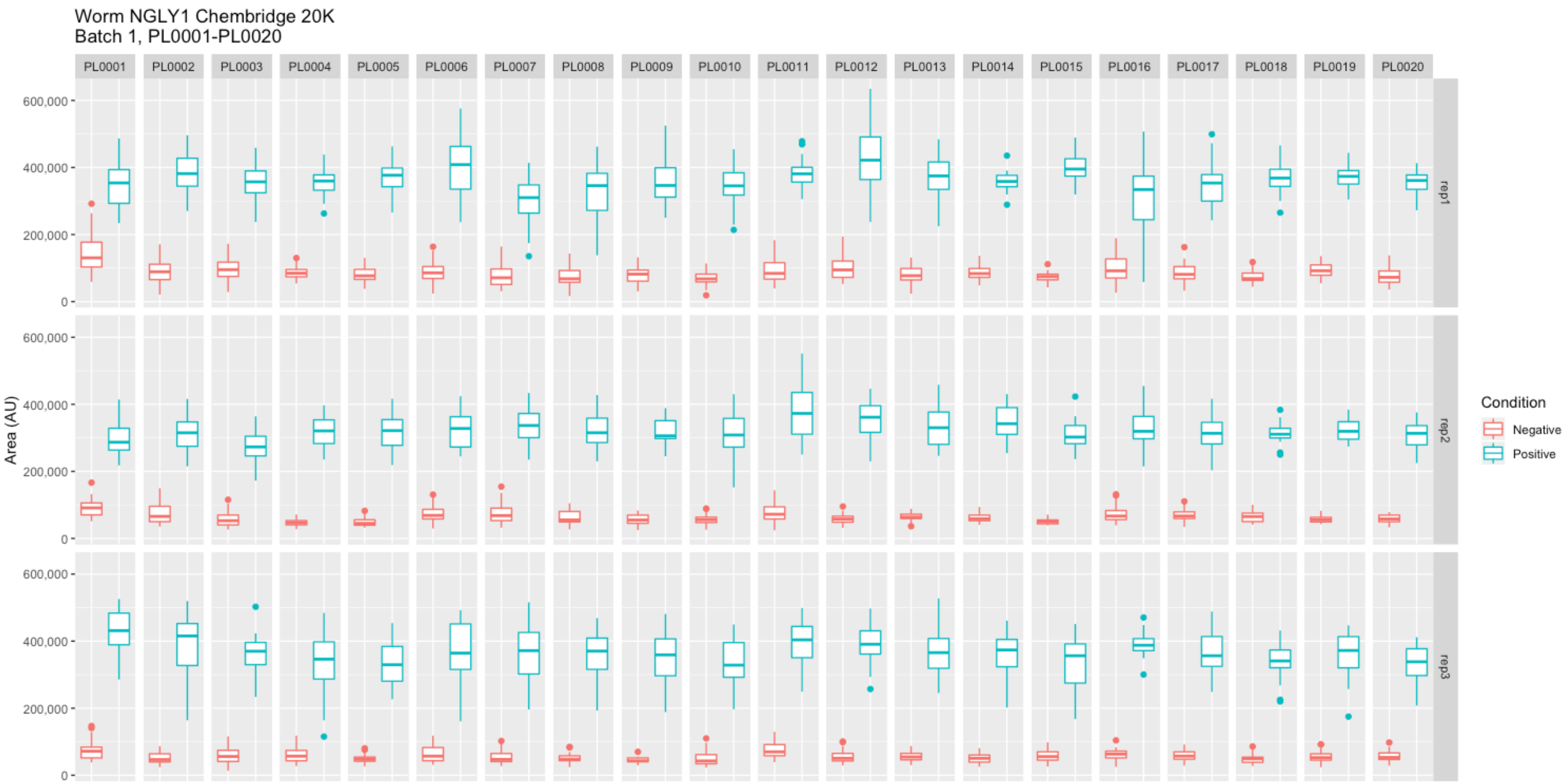

Figure S3. Box plots showing size separation of positive control versus negative control wells in the 20,240-compound lead discovery screen of *png-1* homozygous worms.Area (Arbitrary Units) refers to the area of each well that is occupied by worms.

Disease Models & Mechanisms • Supplementary information

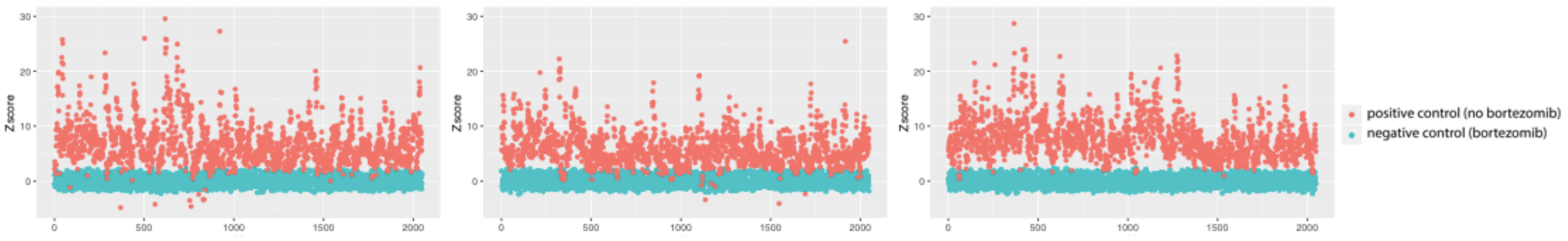

Figure S4. Box plots showing size separation of positive control versus negative control wells in the 20,240-compound lead discovery screen of *Pngl* $^{+/-}$ fly larvae. *Z* scores were calculated as described in Methods and Materials.

Disease Models & Mechanisms • Supplementary information

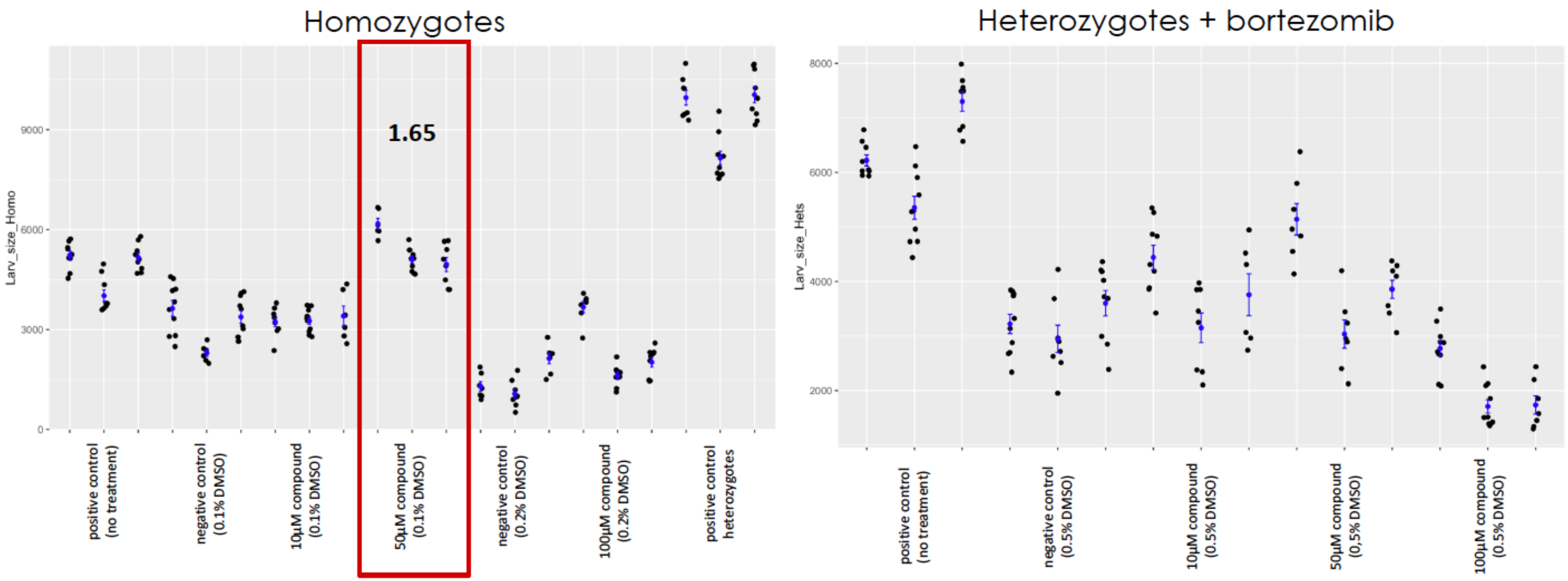

Figure S5. Fly *Pngl*$^{-/-}$ homozygote and 9µM bortezomib-treated *Pngl*$^{+/-}$ heterozygote larval size plots. Black dots represent individual larval sizes with means indicated by blue dots and standard deviations indicated by blue bars. Positive controls are untreated homozygotes. Negative controls are DMSO-treated homozygotes. 50µM sulforaphane fully rescues DMSO-treated homozygotes (red box). 1.65 refers to the fold change in larval size difference between sulforaphane-treated homozygotes and the negative control.

Fly Microsource Spectrum Retests

All compounds that scored as hits in the fly *Pngl*$^{+/-}$ Microsource Spectrum (drug repurposing) screen were retested in two fly secondary assays : rescue of *Pngl*$^{-/-}$ homozygotes and rescue of bortezomib-treated *Pngl*$^{+/-}$ heterozygotes. Red boxes indicate compounds that successfully retested. The number indicates the size ratio of compound-treated fly larvae to DMSO-treated control homozygous fly larvae. For example, 2 means that a compound increased the size of mutant larvae by twofold.

(Please note that some homozygote experiments included untreated heterozygote controls)

Fly Microsource retest –2-METHYLENE-5-(2,5-DIOXOTETRAHYDROFURAN-3-YL)-6-OXO--10,10-DIMETHYL

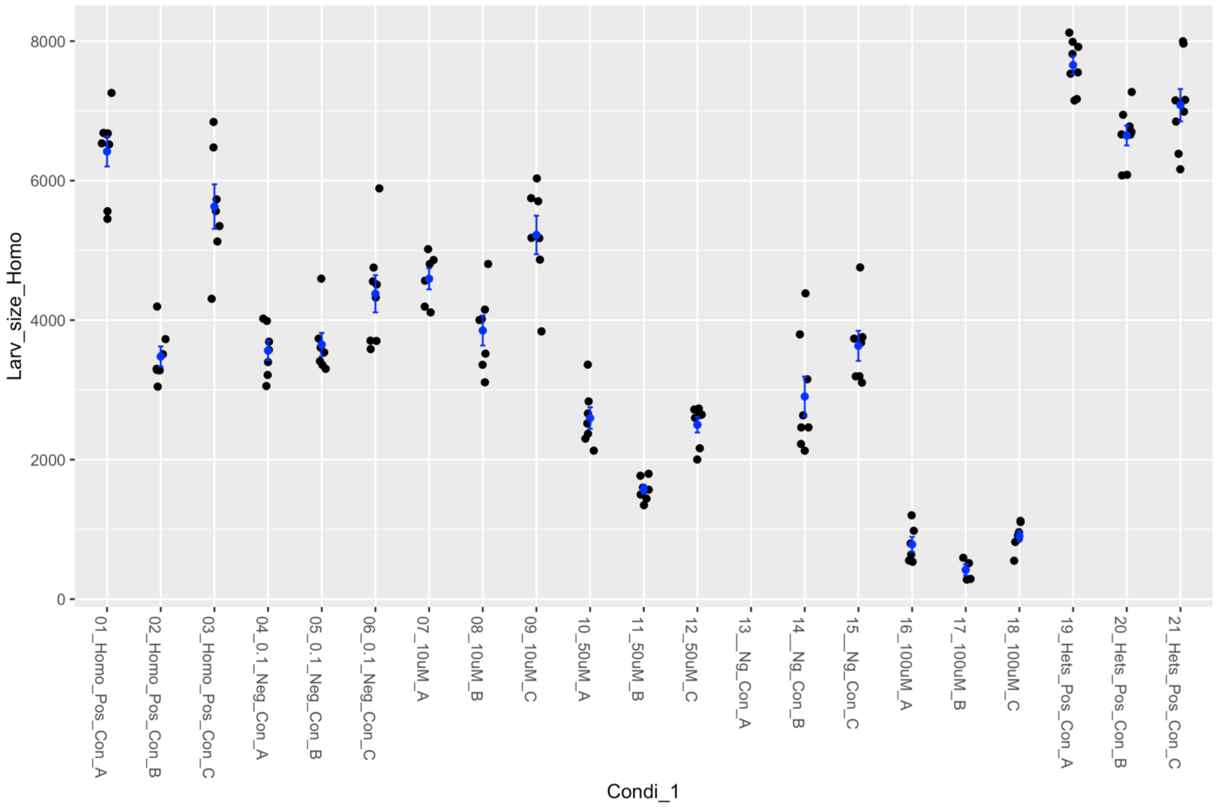

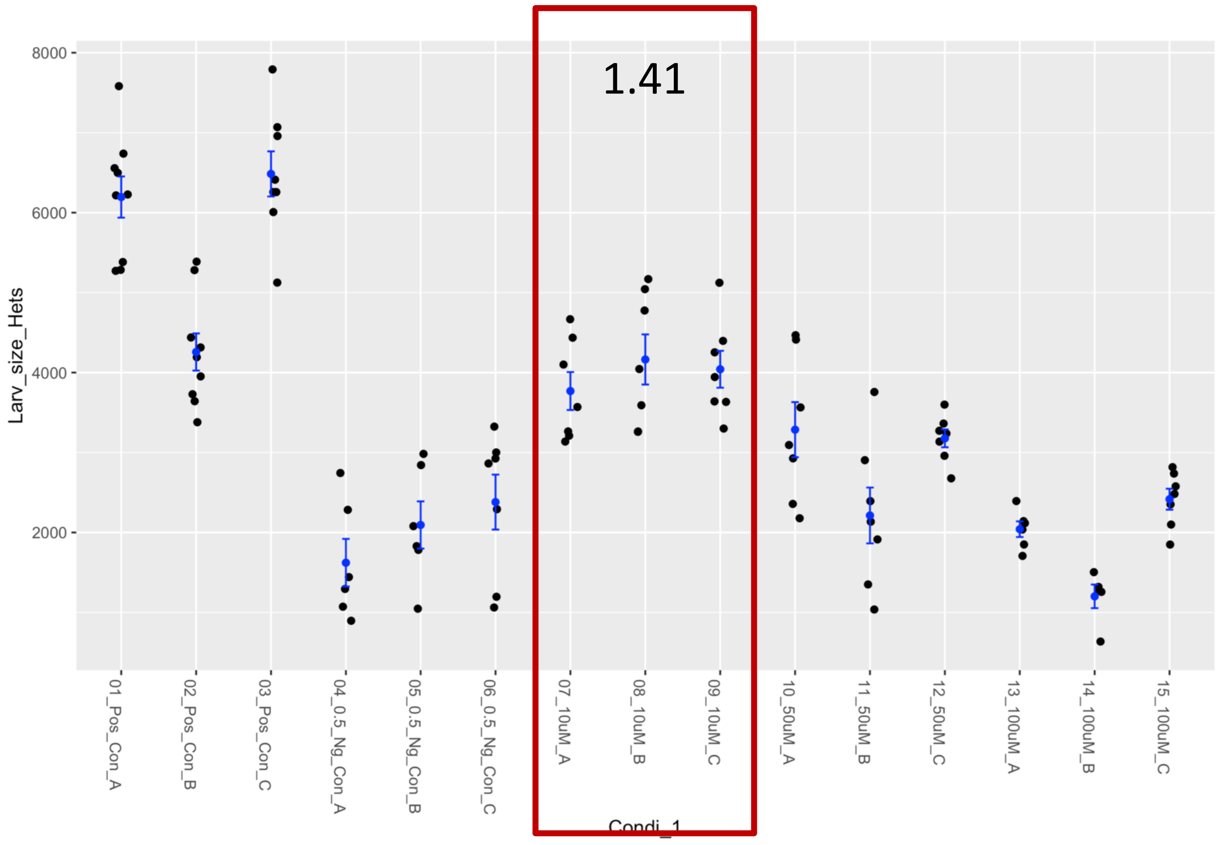

Disease Models & Mechanisms • Supplementary information

Fly Microsource Retest – AMINACRINE

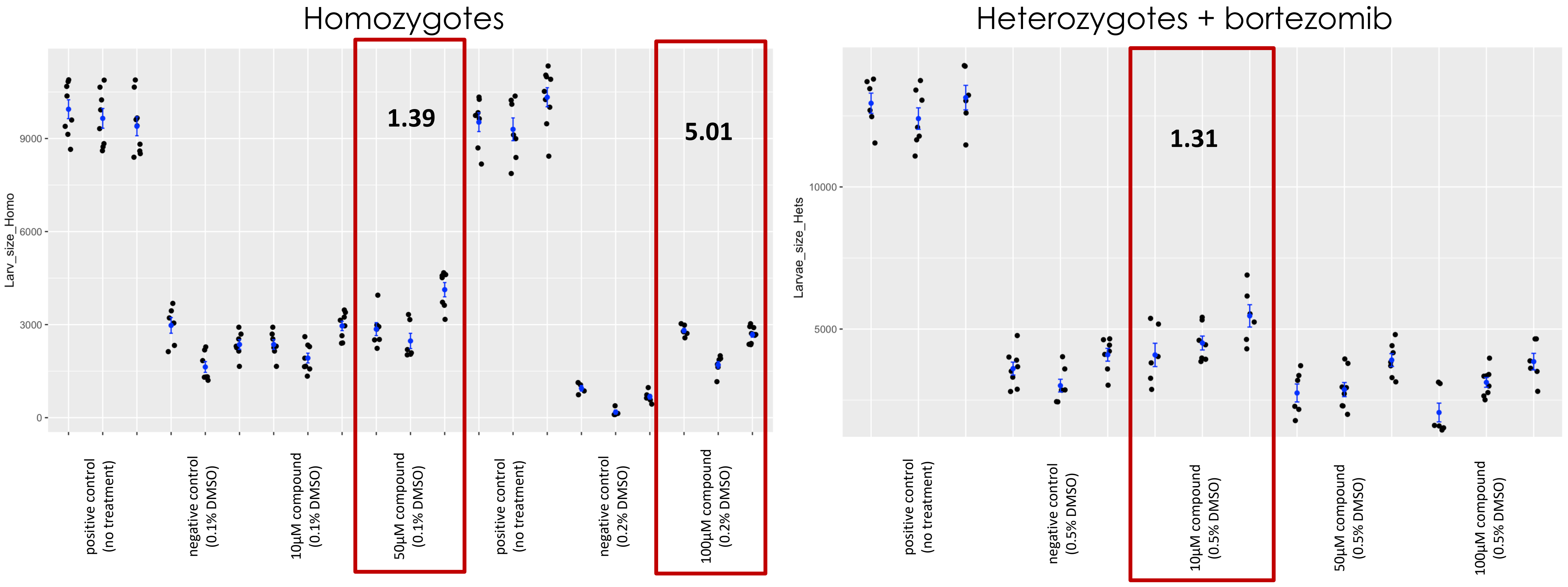

Disease Models & Mechanisms • Supplementary information

Fly Microsource Retest – ARIPIPRAZOLE

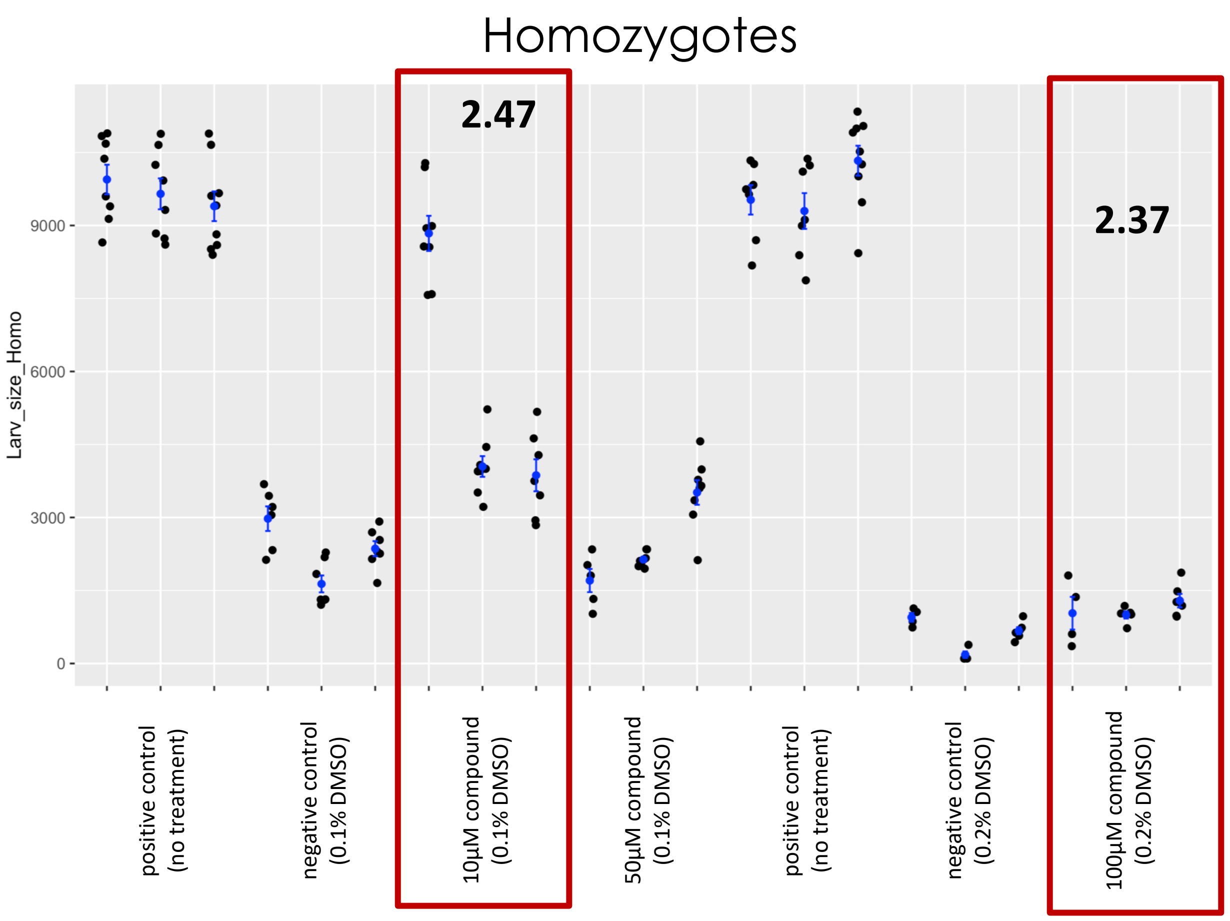

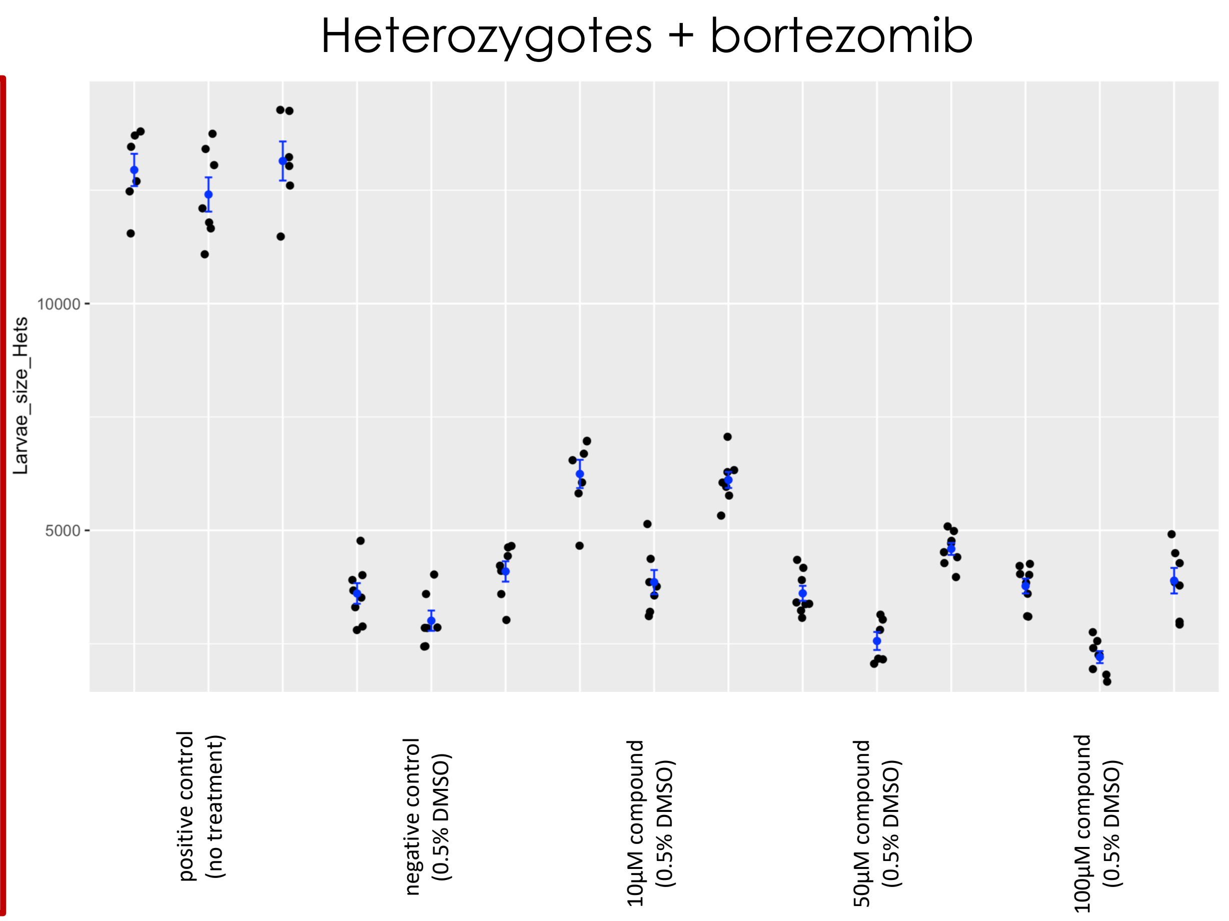

Fly Microsource Retest – beta-AMYRIN

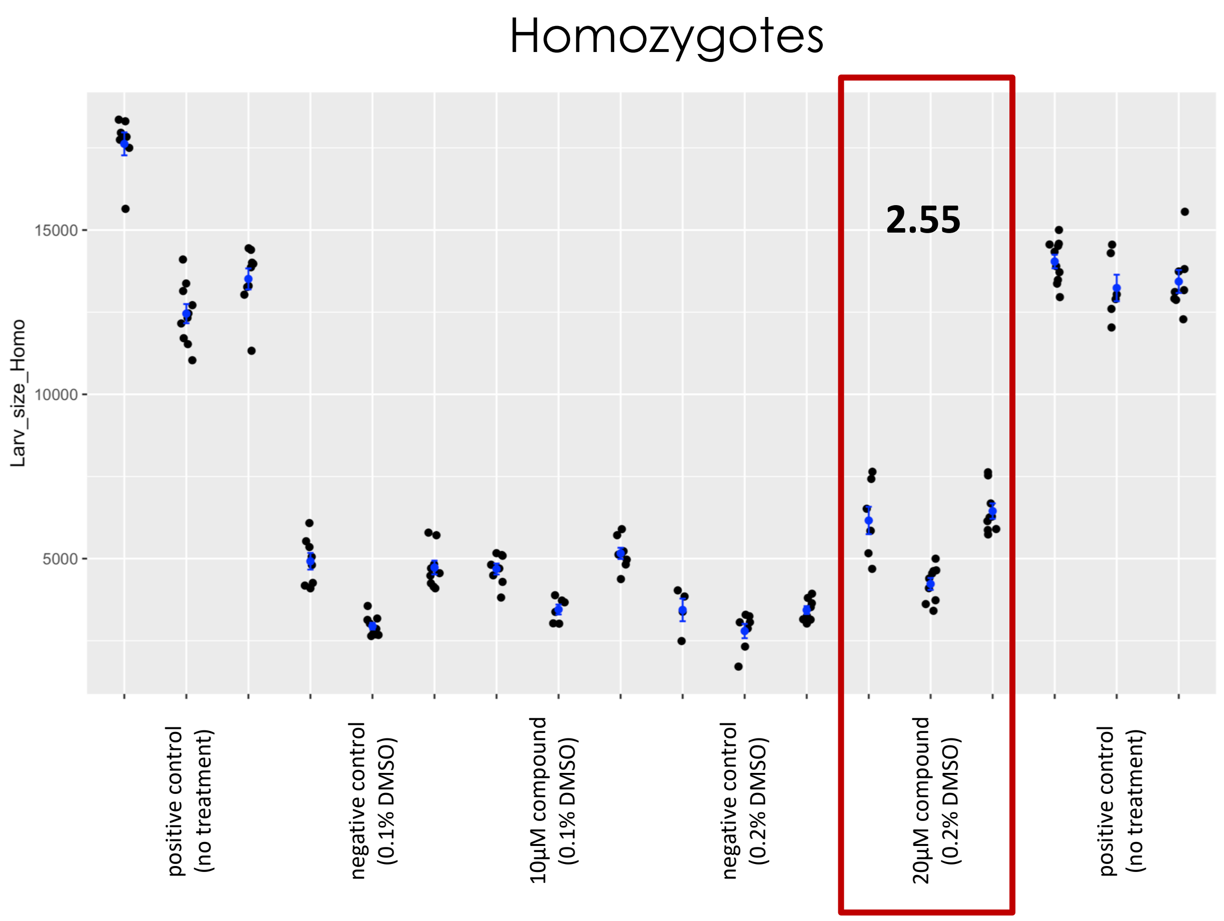

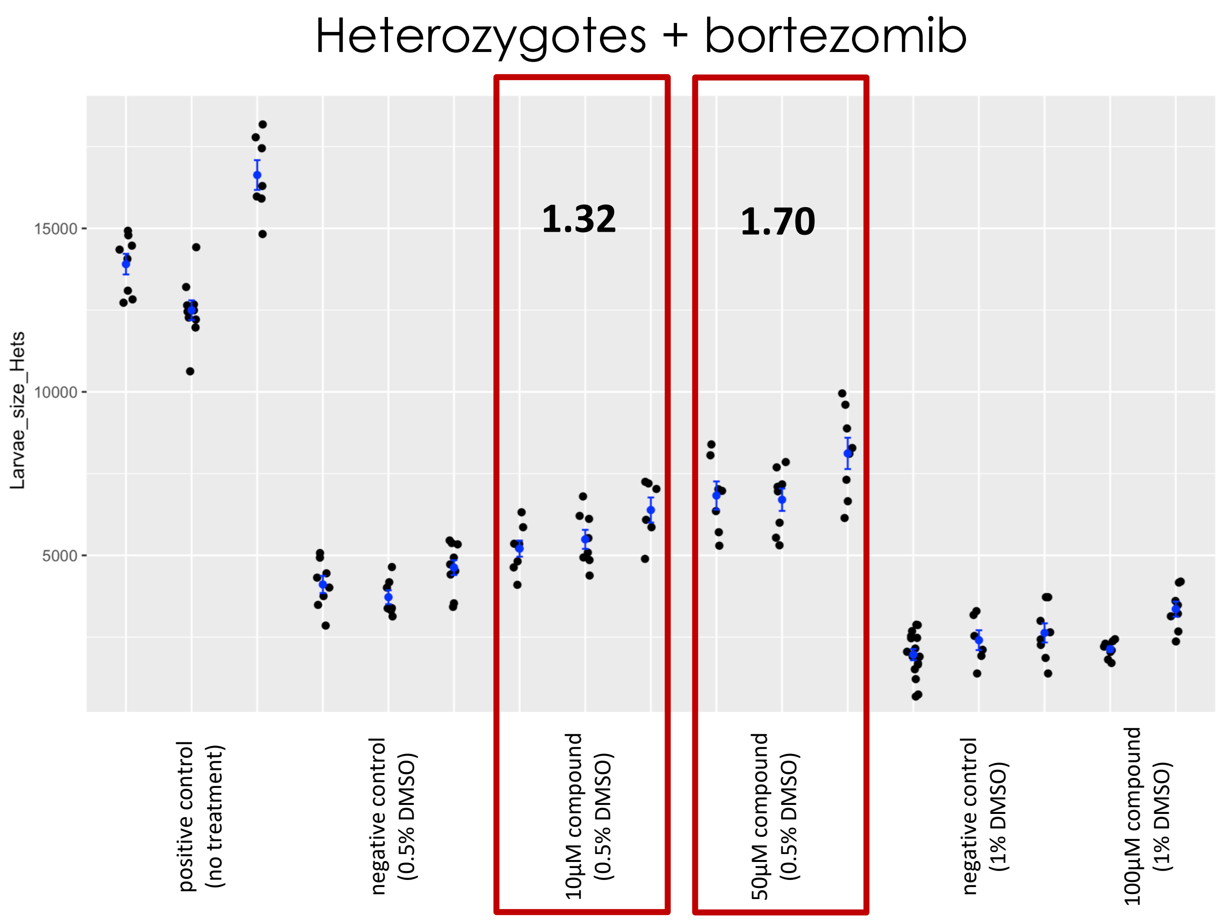

Disease Models & Mechanisms • Supplementary information

Fly Microsource Retest – BUTOPYRONOXYL

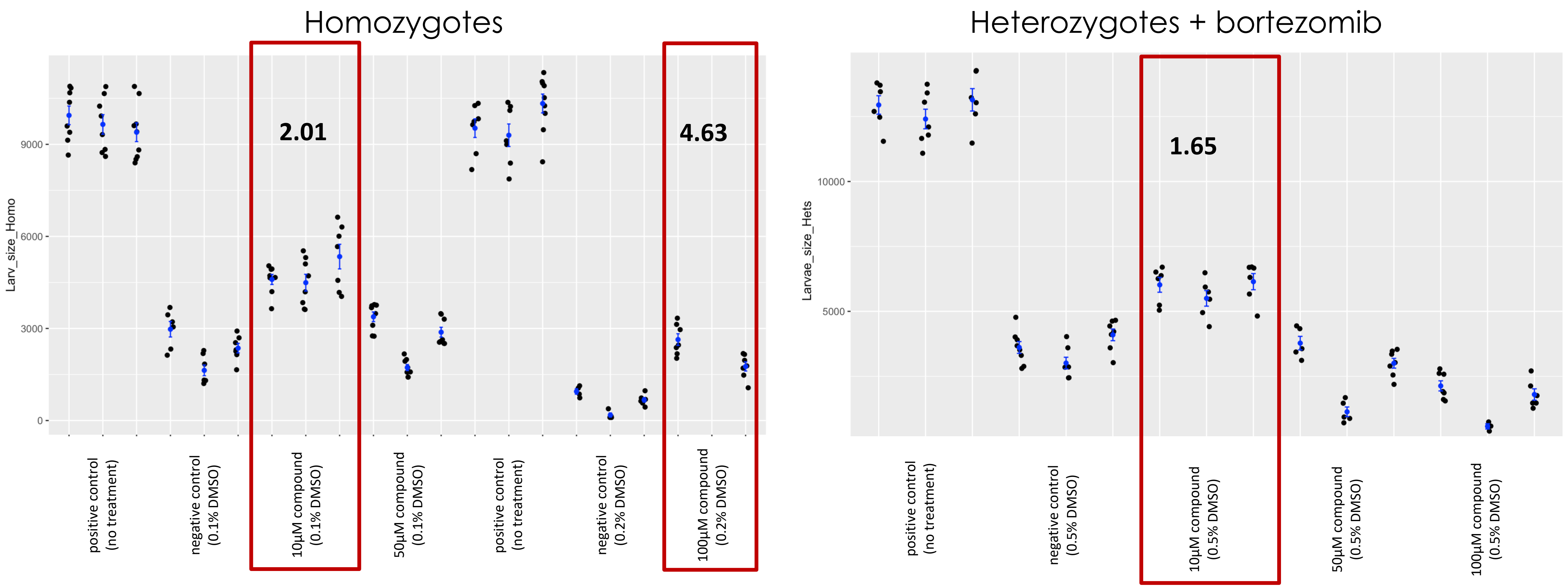

Disease Models & Mechanisms • Supplementary information

Fly Microsource Retest – CIMICIFUGOSIDE

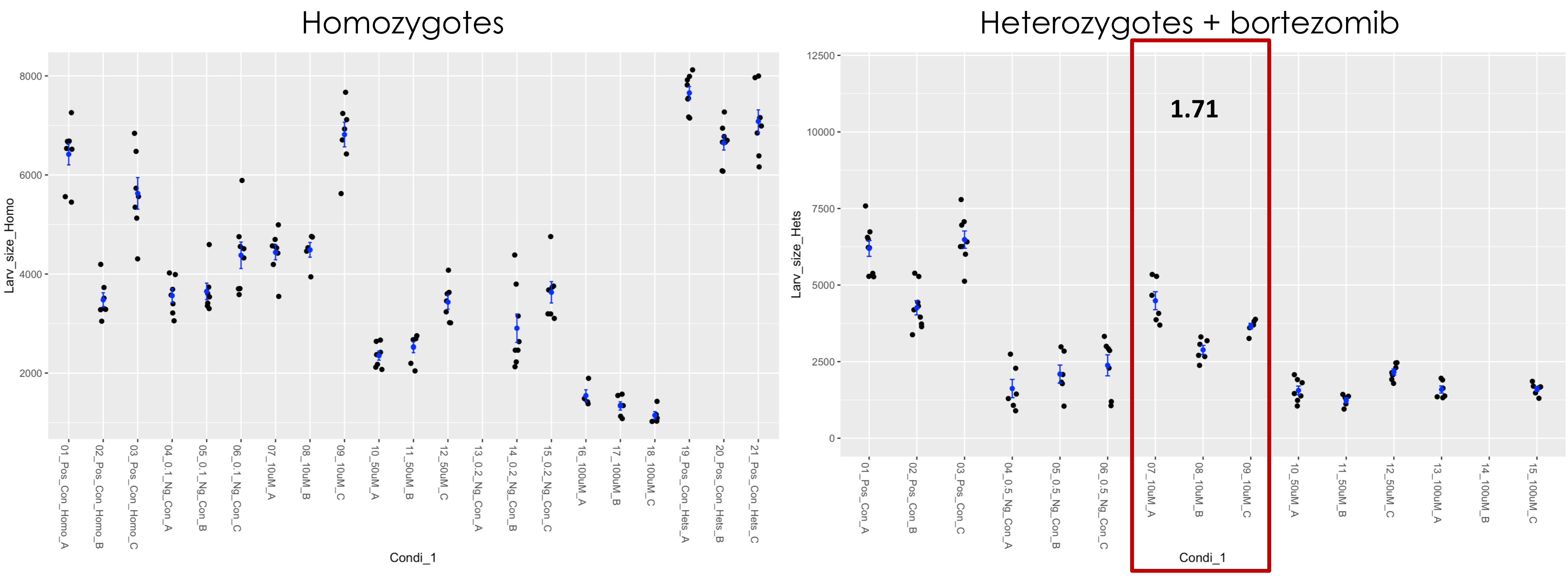

Disease Models & Mechanisms • Supplementary information

Fly Microsource Retest – CINNARAZINE

Homozygotes

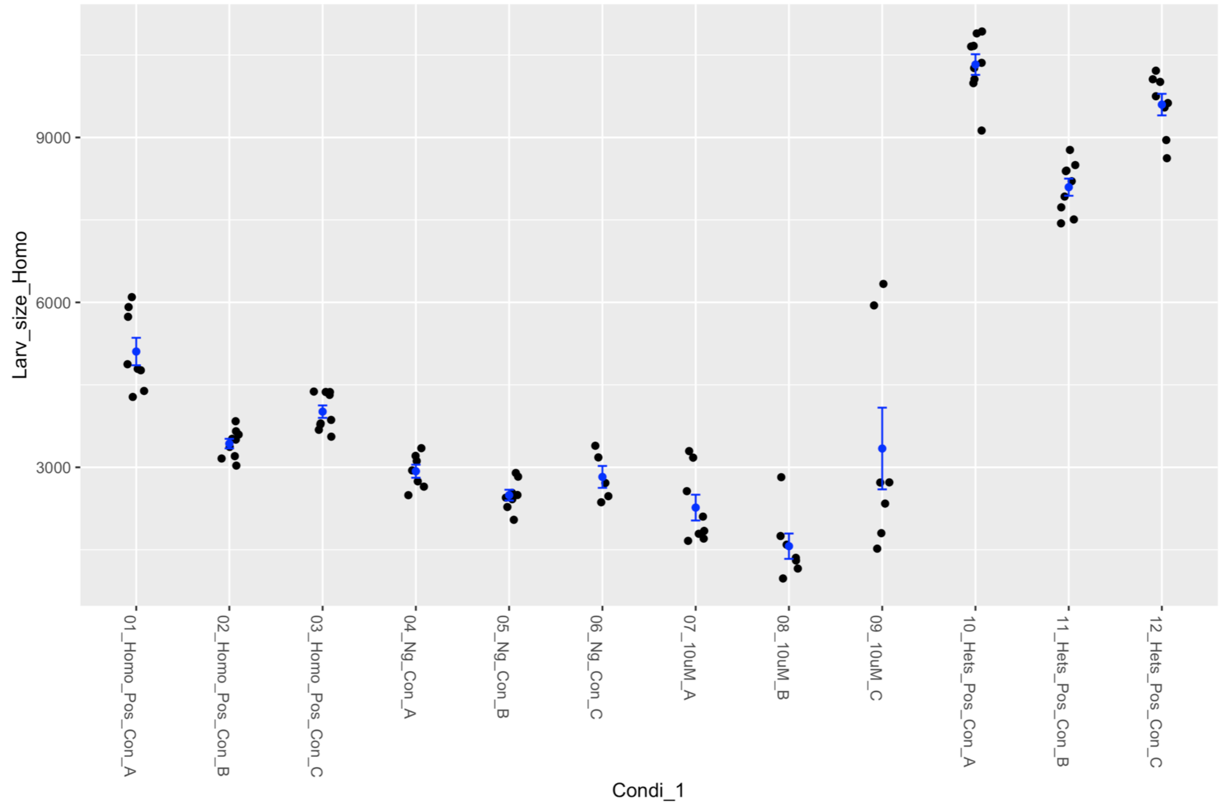

Heterozygotes + bortezomib

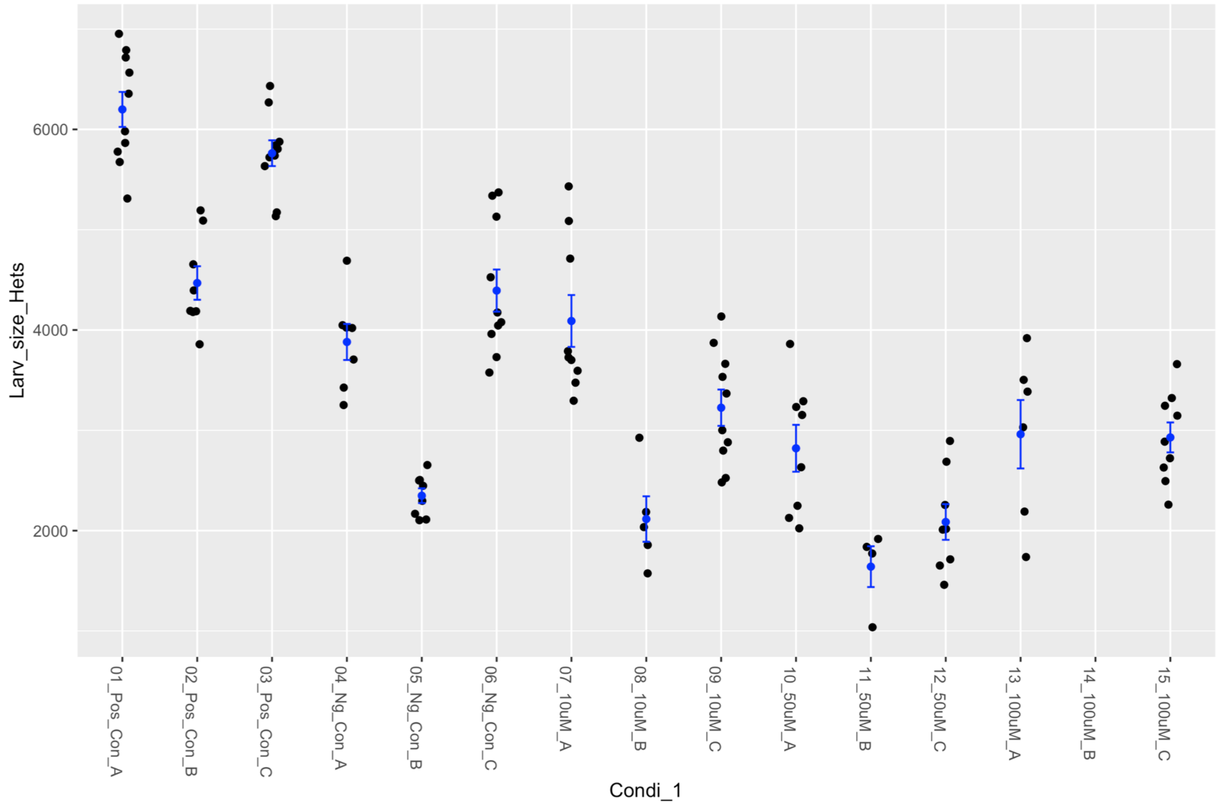

Fly Microsource Retest – CLINDAMYCIN HYDROCHLORIDE

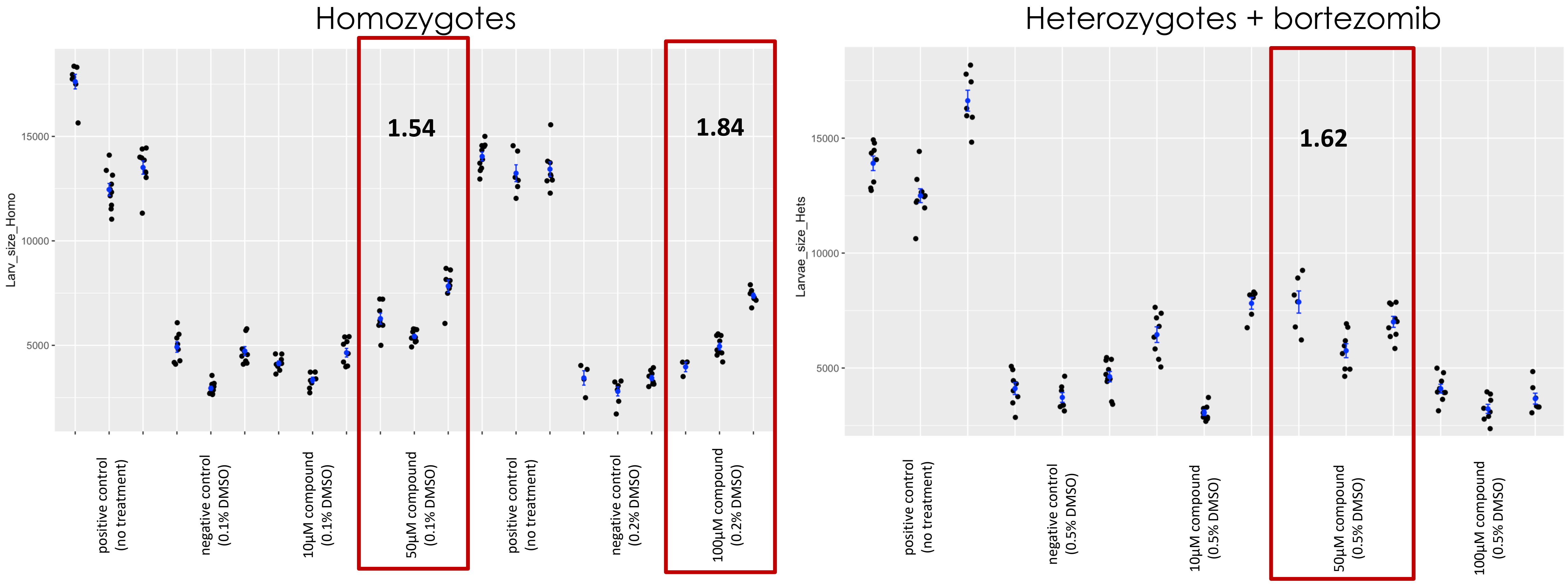

Fly Microsource Retest – CLOPERASTINE HYDROCHLORIDE

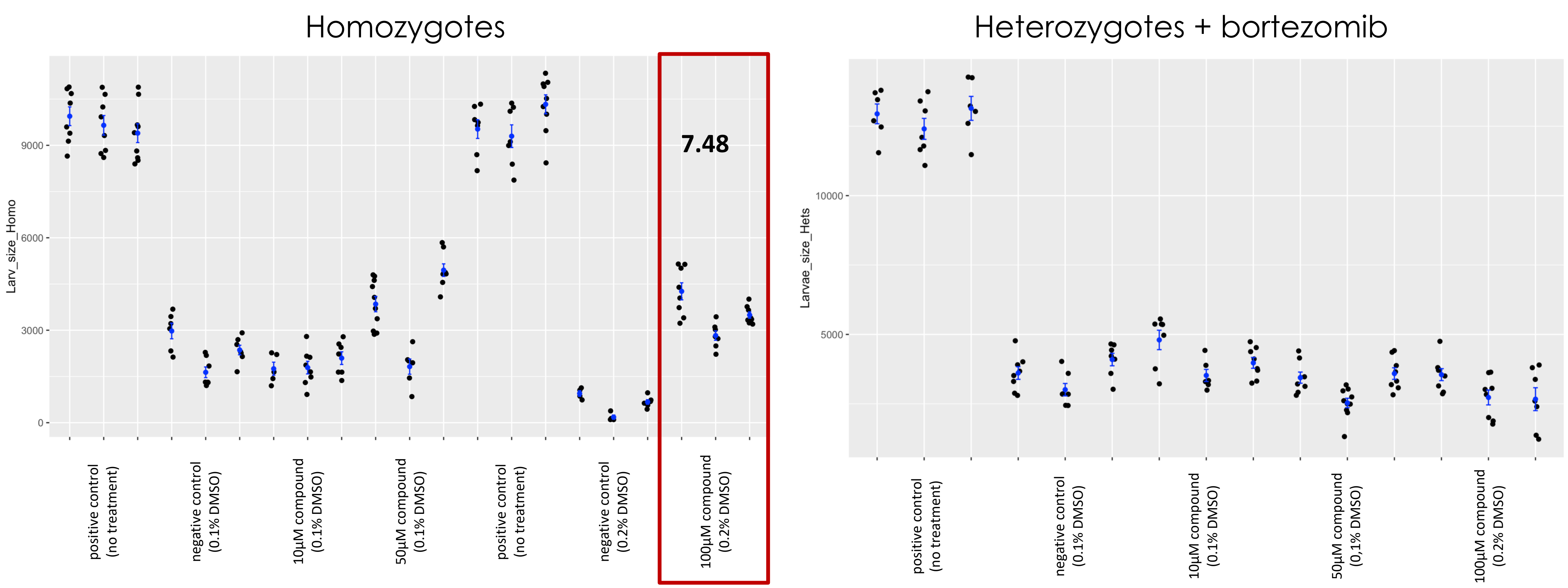

Disease Models & Mechanisms • Supplementary information

Fly Microsource retest – CYCLANDELATE

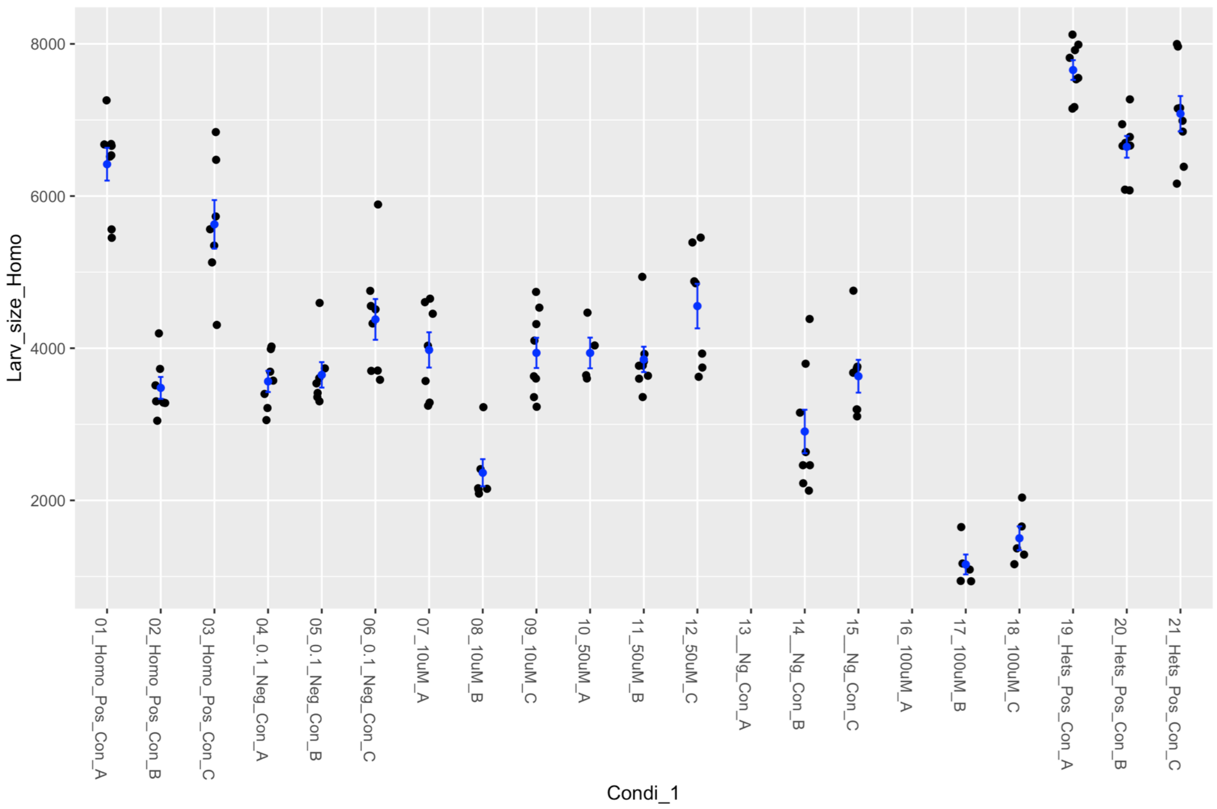

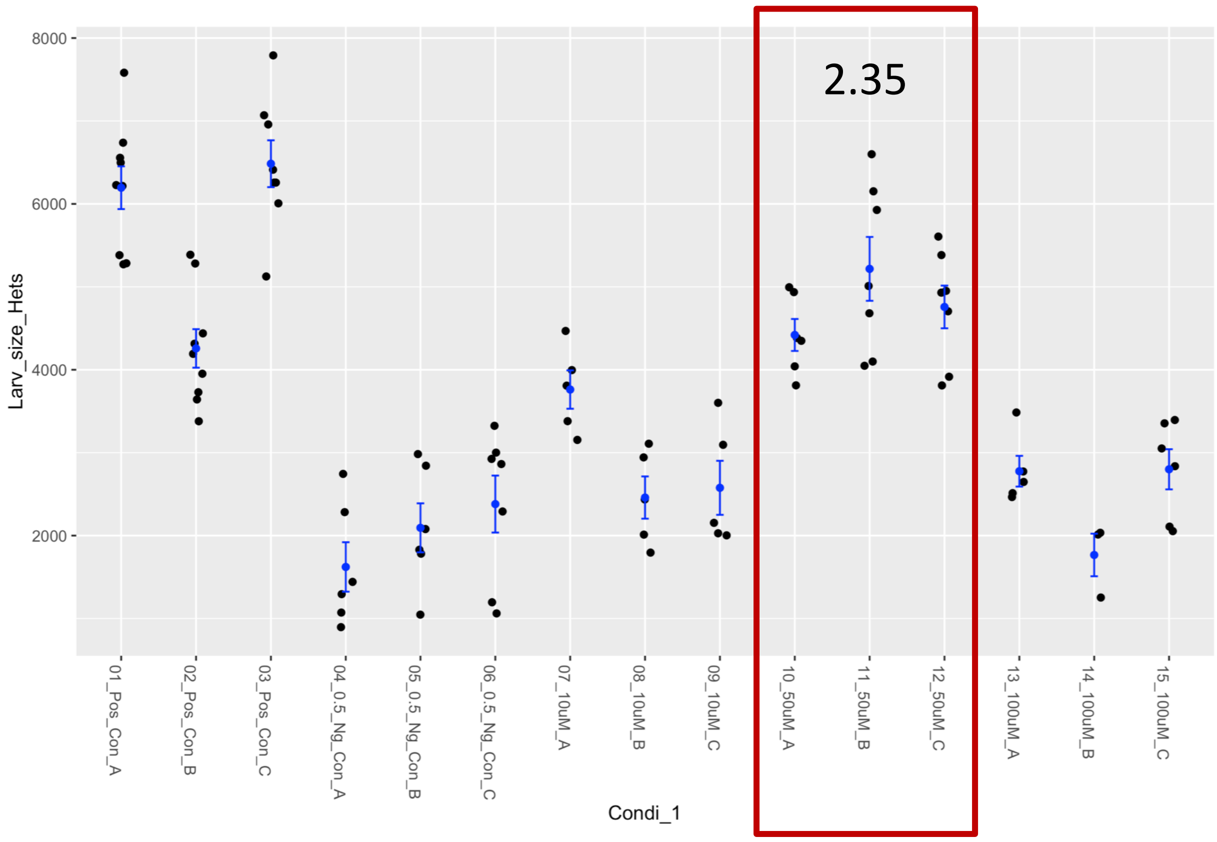

Disease Models & Mechanisms • Supplementary information

Fly Microsource retest – CYSTINE

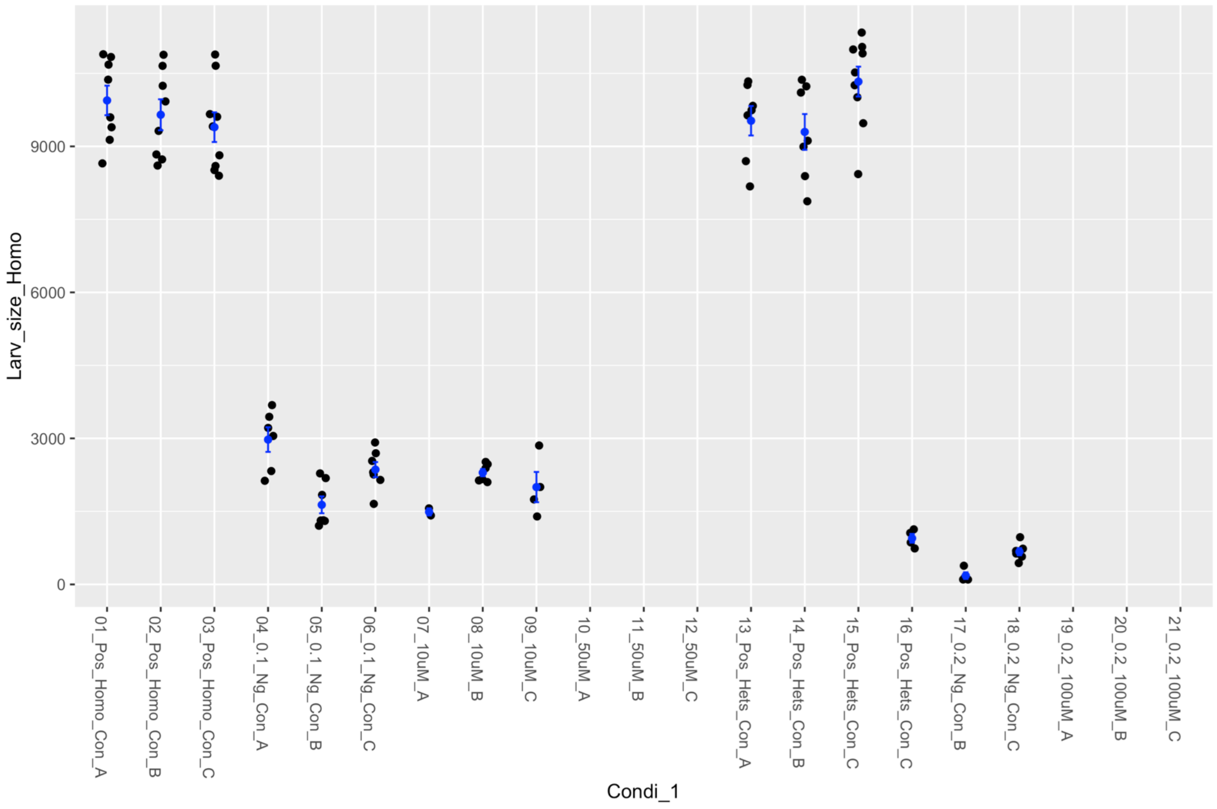

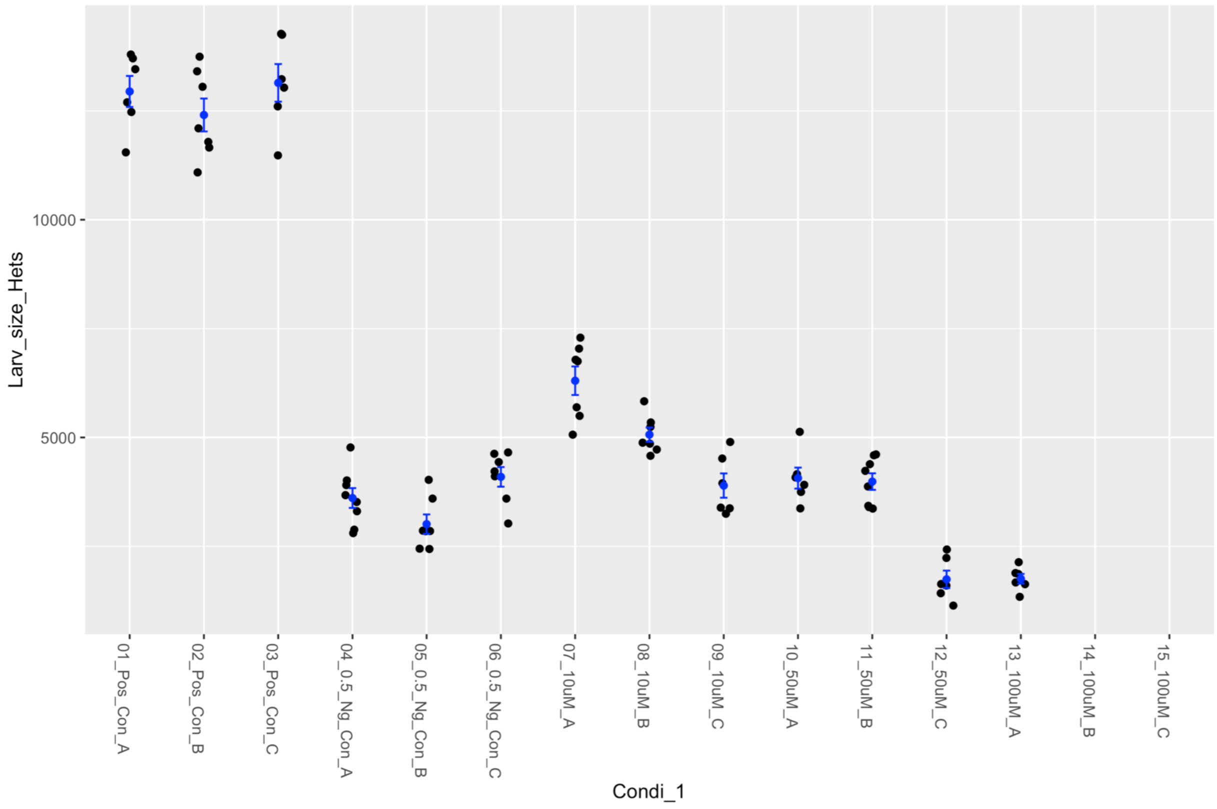

Disease Models & Mechanisms • Supplementary information

Fly Microsource Retest – DYPHYLLINE

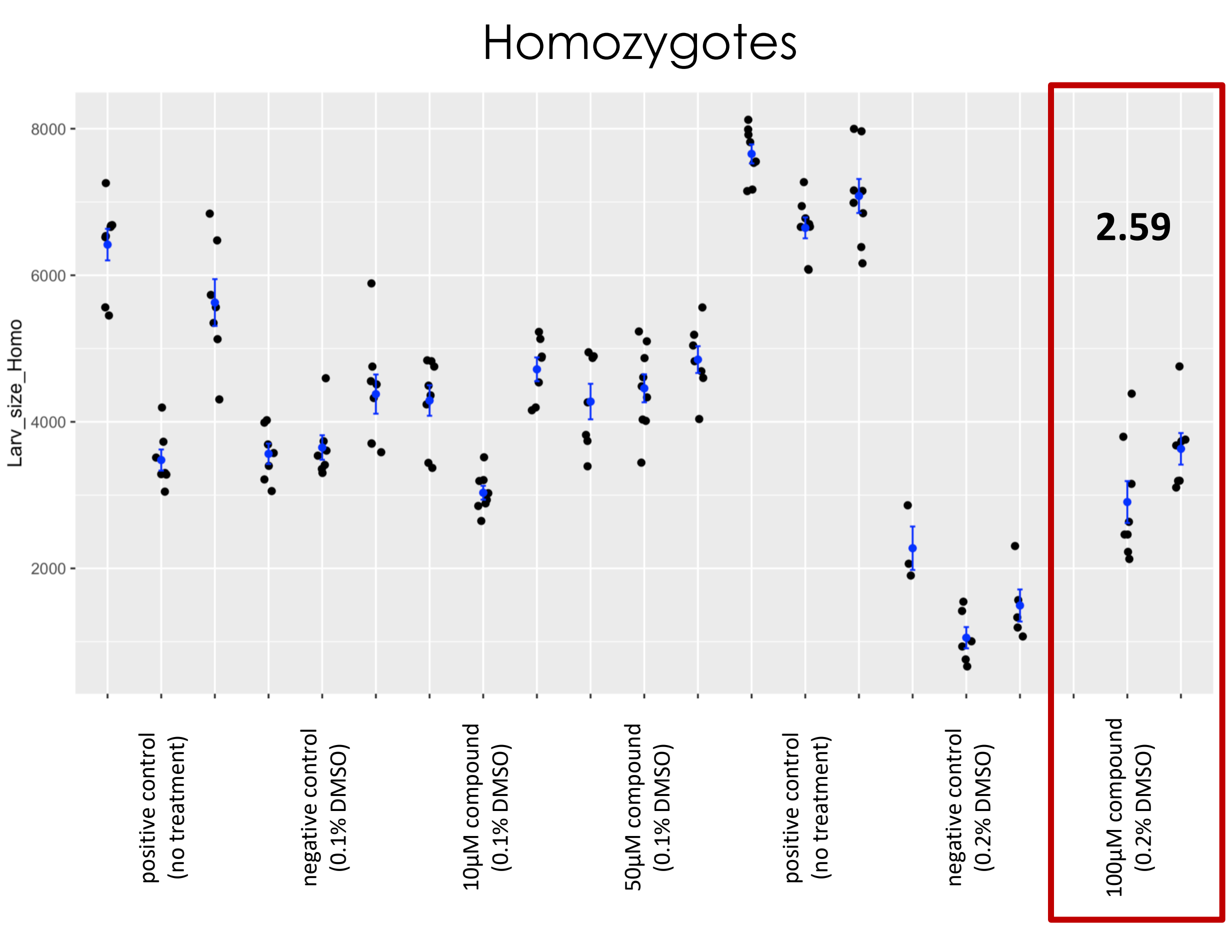

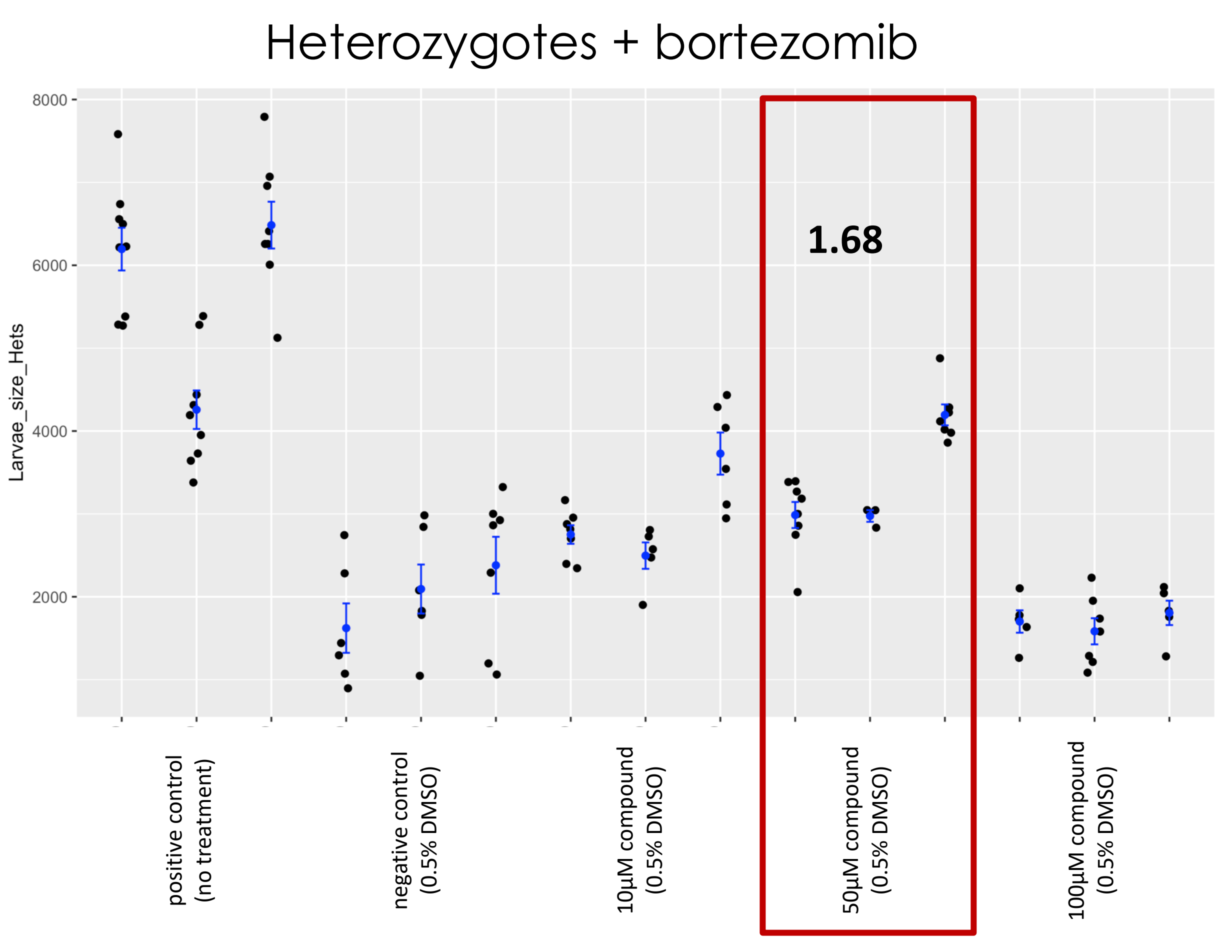

Disease Models & Mechanisms • Supplementary information

Fly Microsource Retest – EPALRESTAT

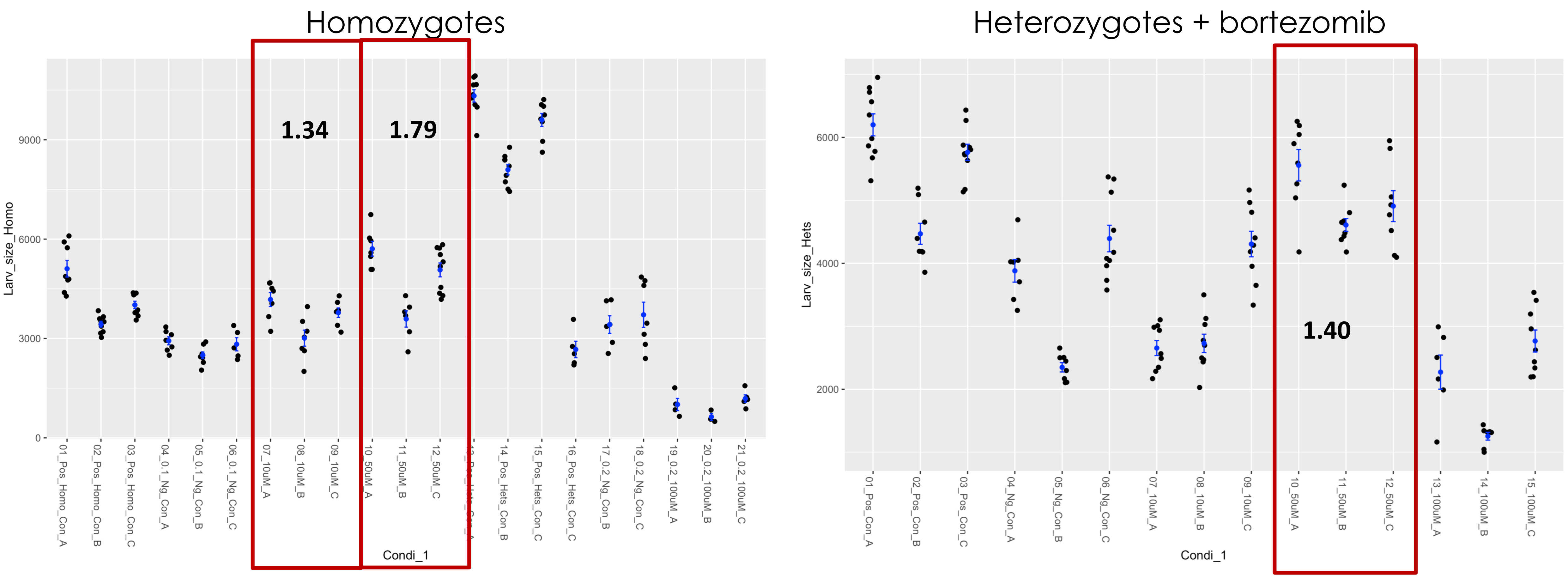

Disease Models & Mechanisms: doi:10.1242/dmm.040576: Supplementary information

Fly Microsource retest – ETHAVERINE HYDROCHLORIDE

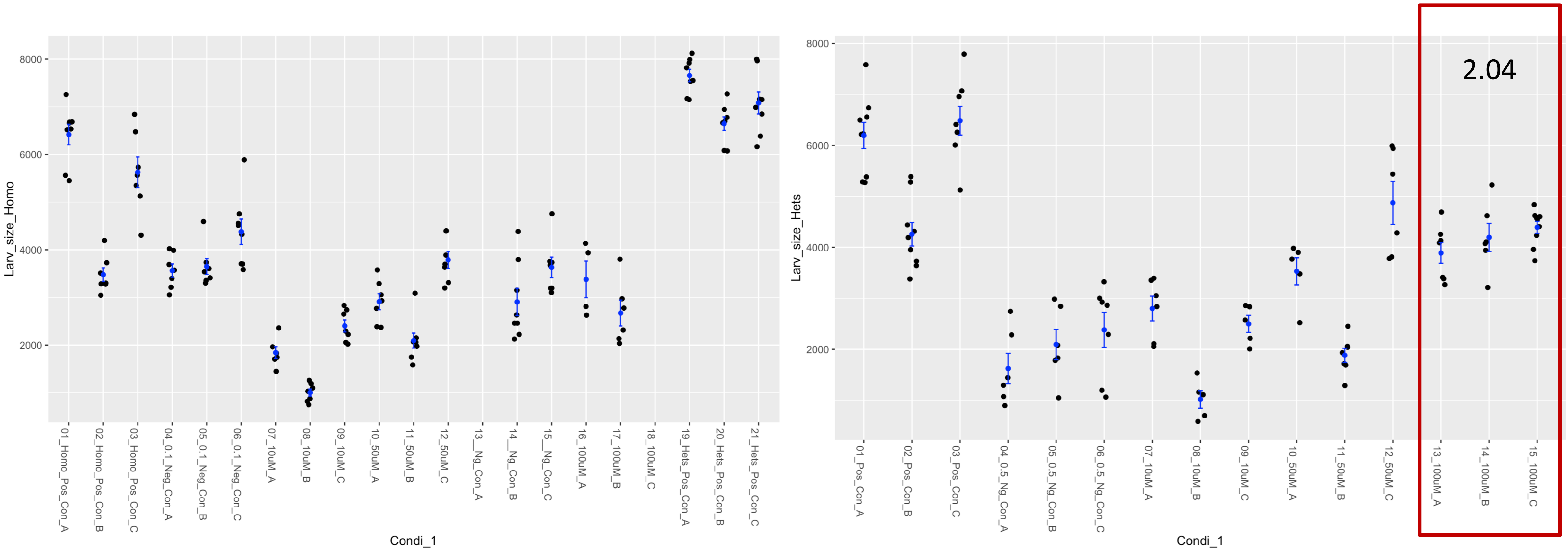

Disease Models & Mechanisms • Supplementary information

Fly Microsource Retest – FOMEPIZOLE HYDROCHLORIDE

Homozygotes

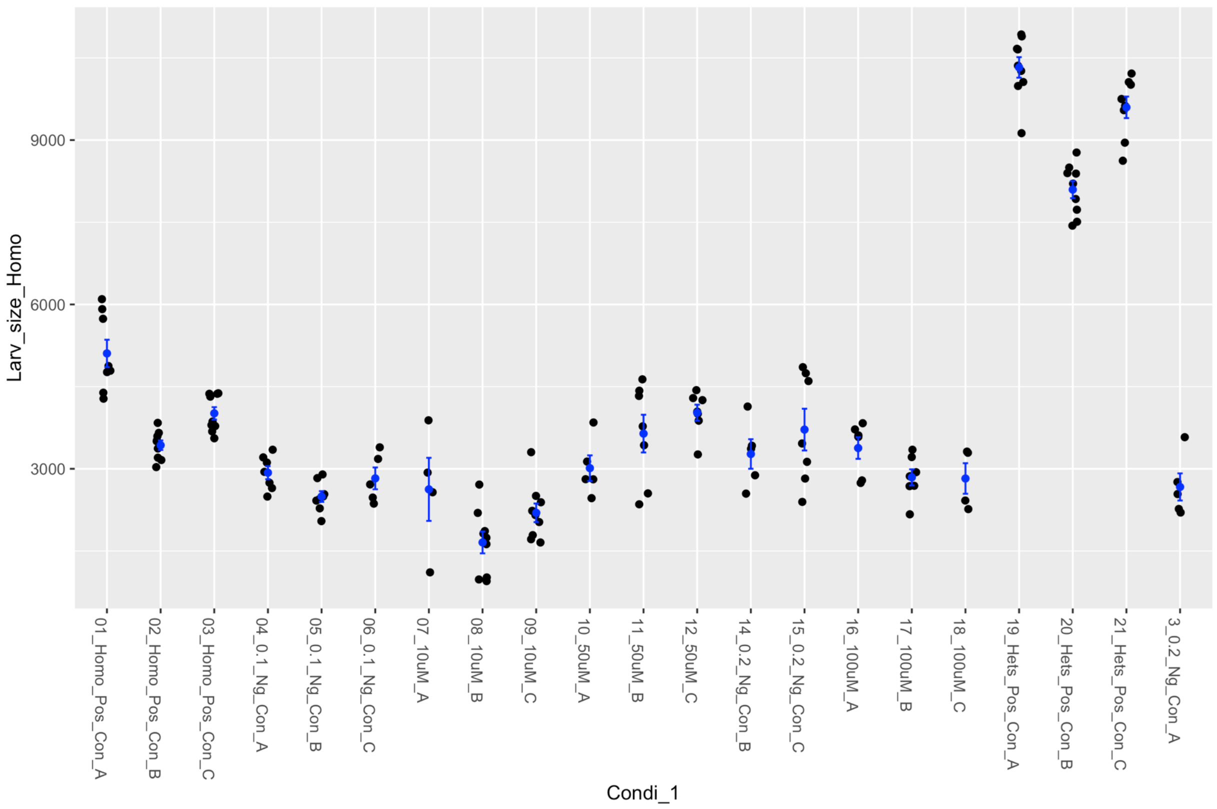

Heterozygotes + bortezomib

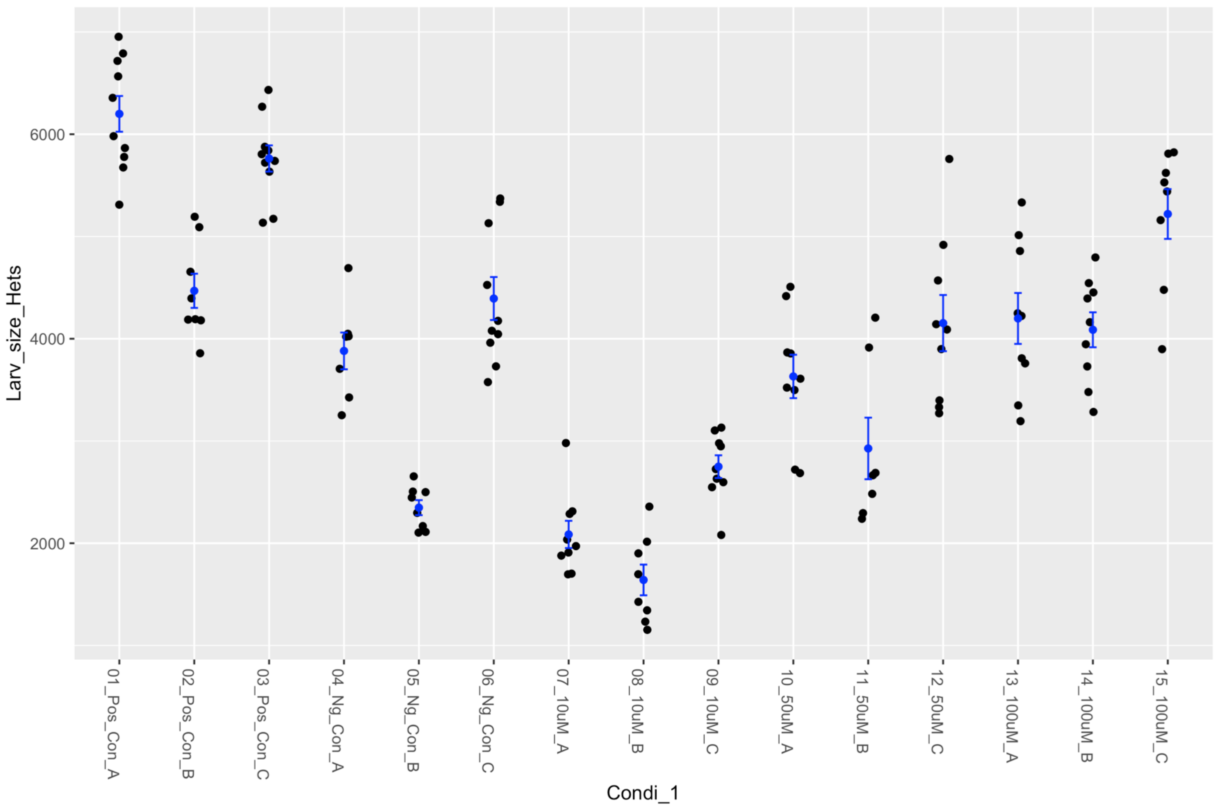

Fly Microsource Retest – GLUTAMINE (L) HYDROCHLORIDE

Homozygotes

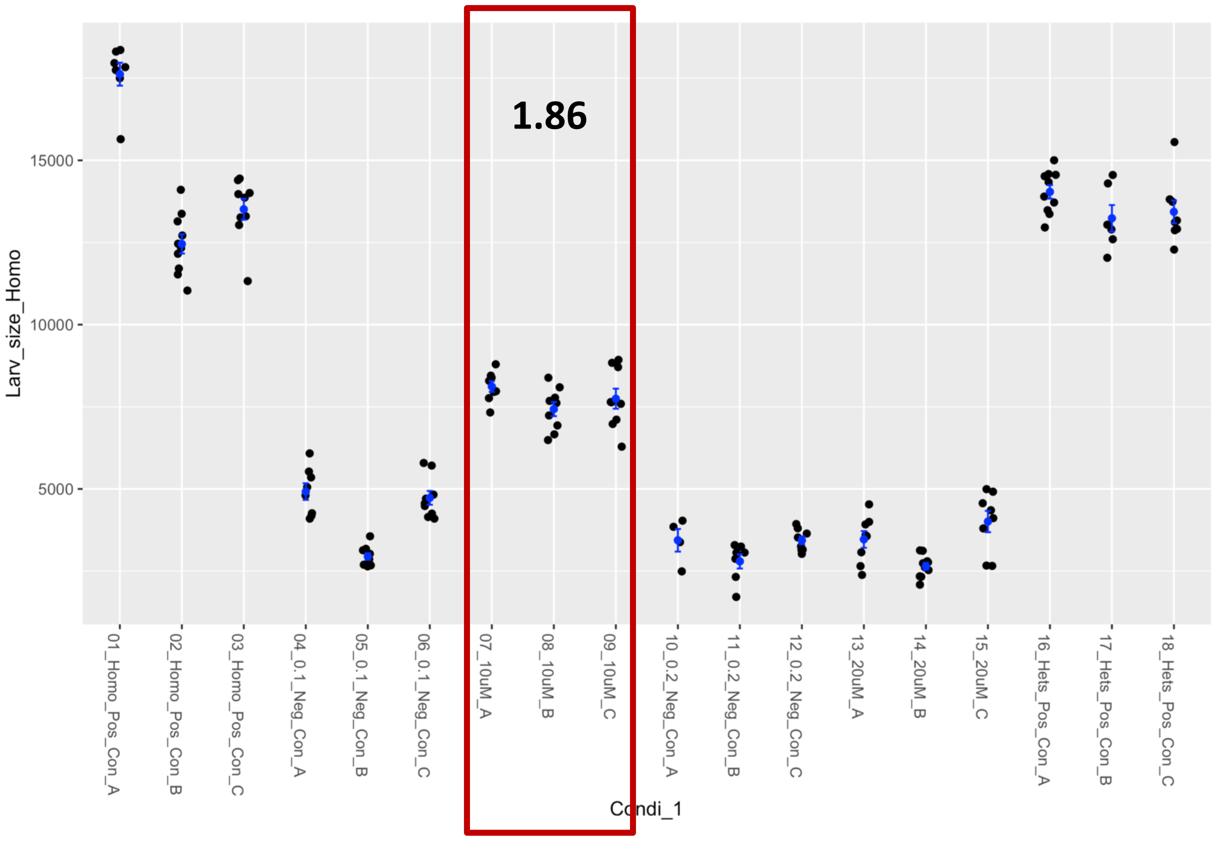

Heterozygotes + bortezomib

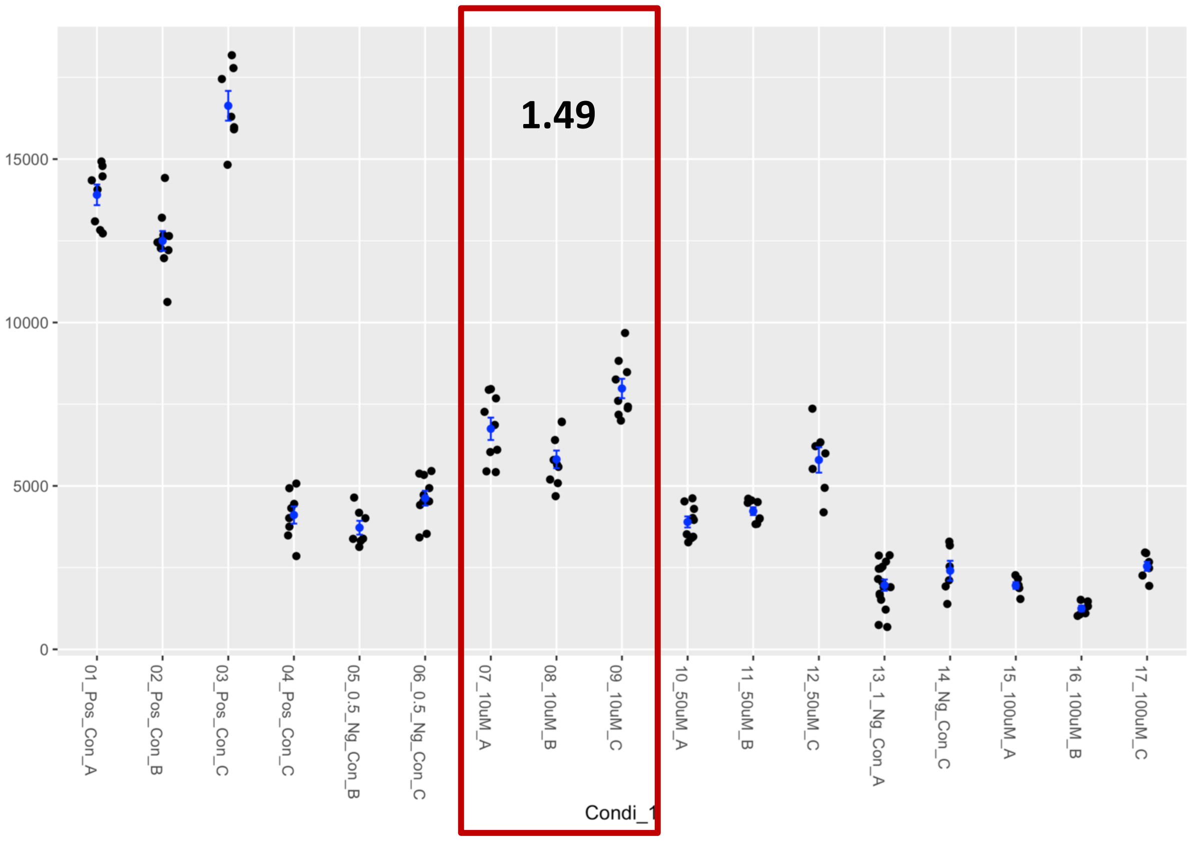

Disease Models & Mechanisms • Supplementary information

Fly Microsource Retest – GOSSYPETIN

Homozygotes

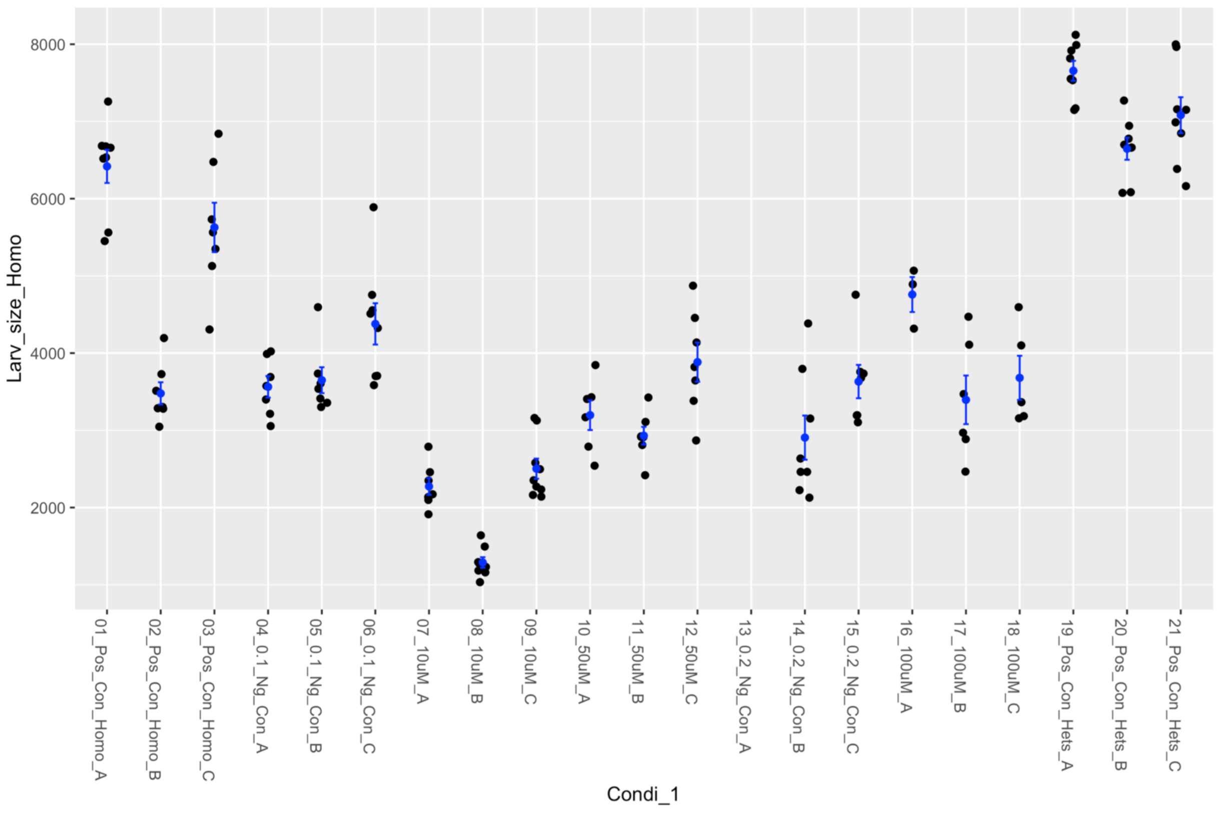

Heterozygotes + bortezomib

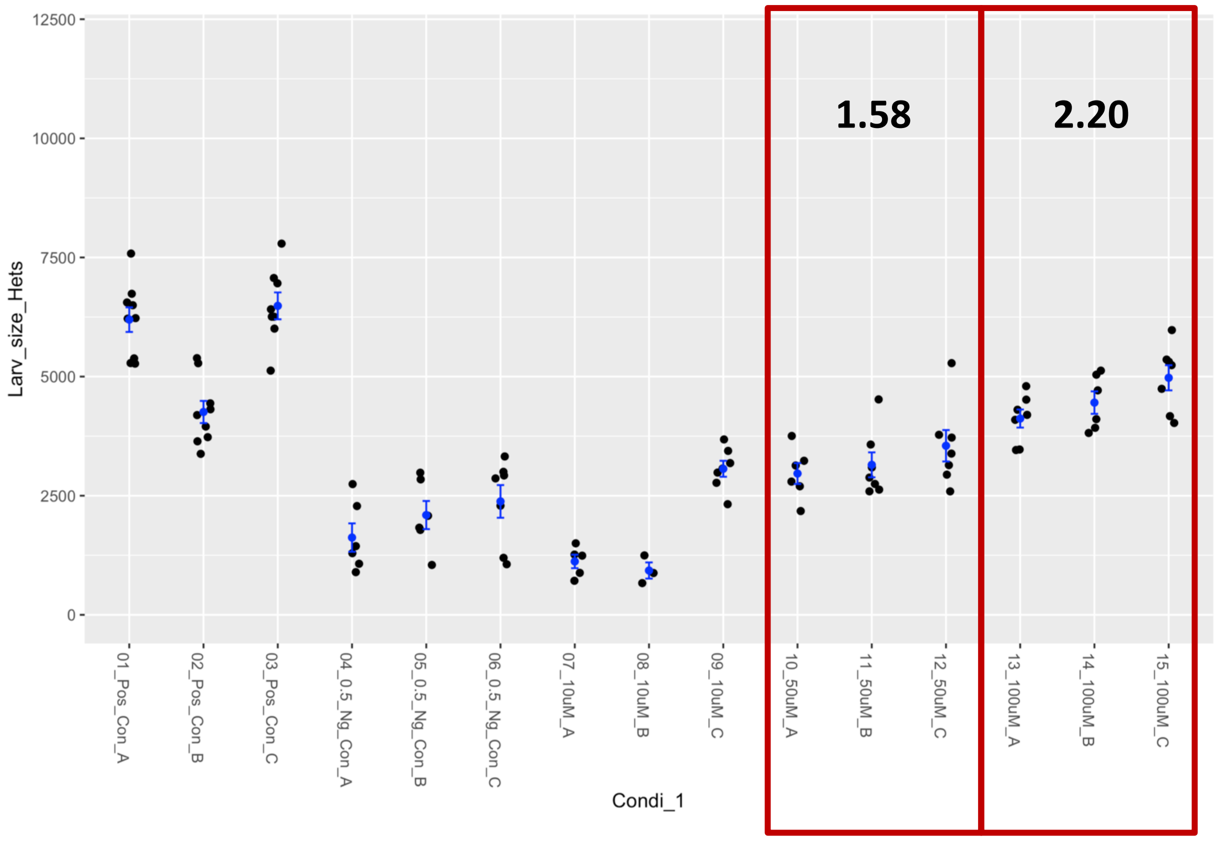

Disease Models & Mechanisms • Supplementary information

Disease Models & Mechanisms: doi:10.1242/dmm.040576: Supplementary information

Fly Microsource retest – HAEMATOXYLIN

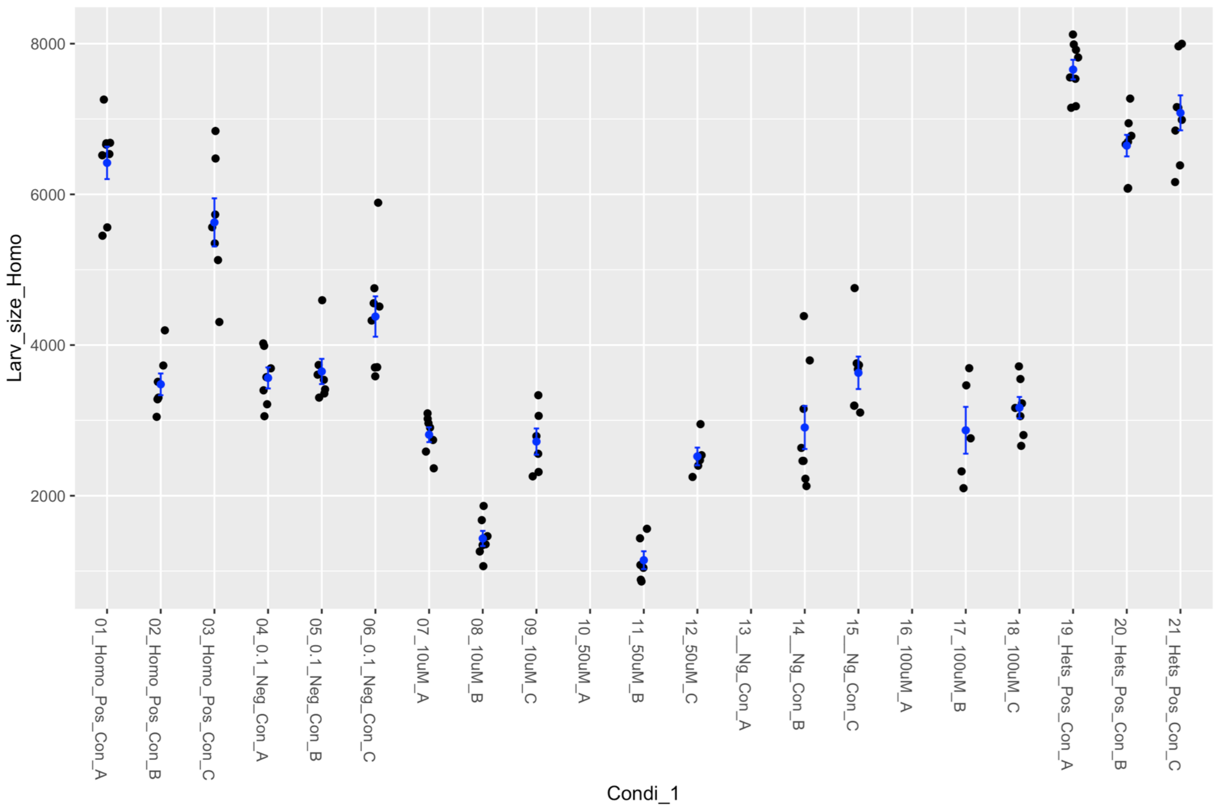

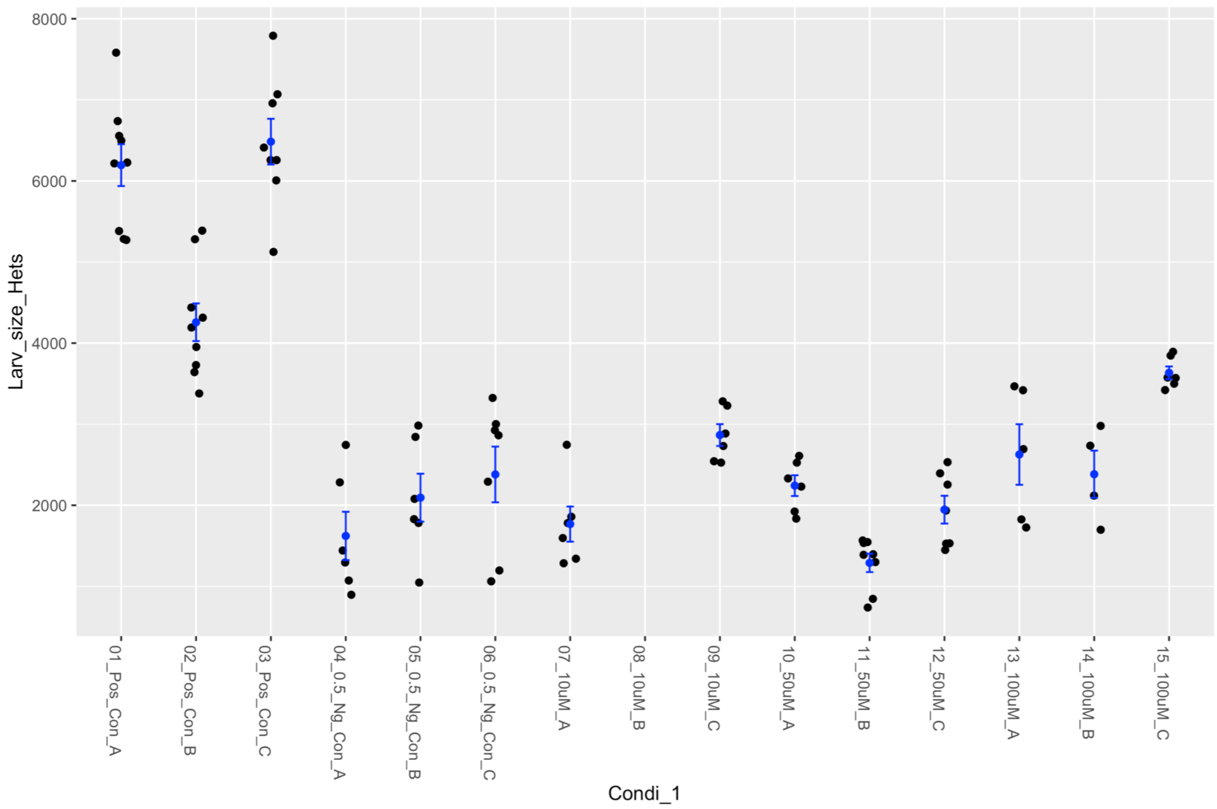

Disease Models & Mechanisms • Supplementary information

Fly Microsource Retest – KETOROLAC TROMETHAMINE

Homozygotes

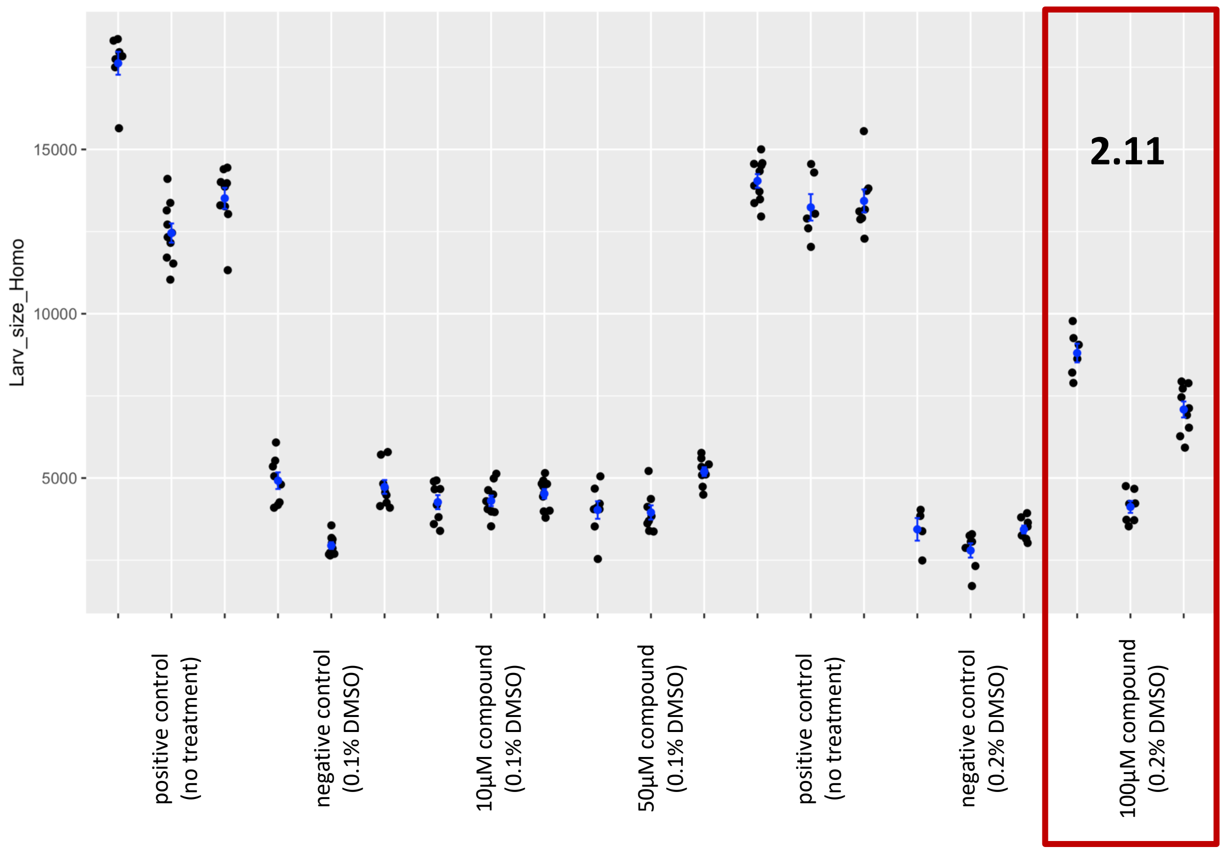

Heterozygotes + bortezomib

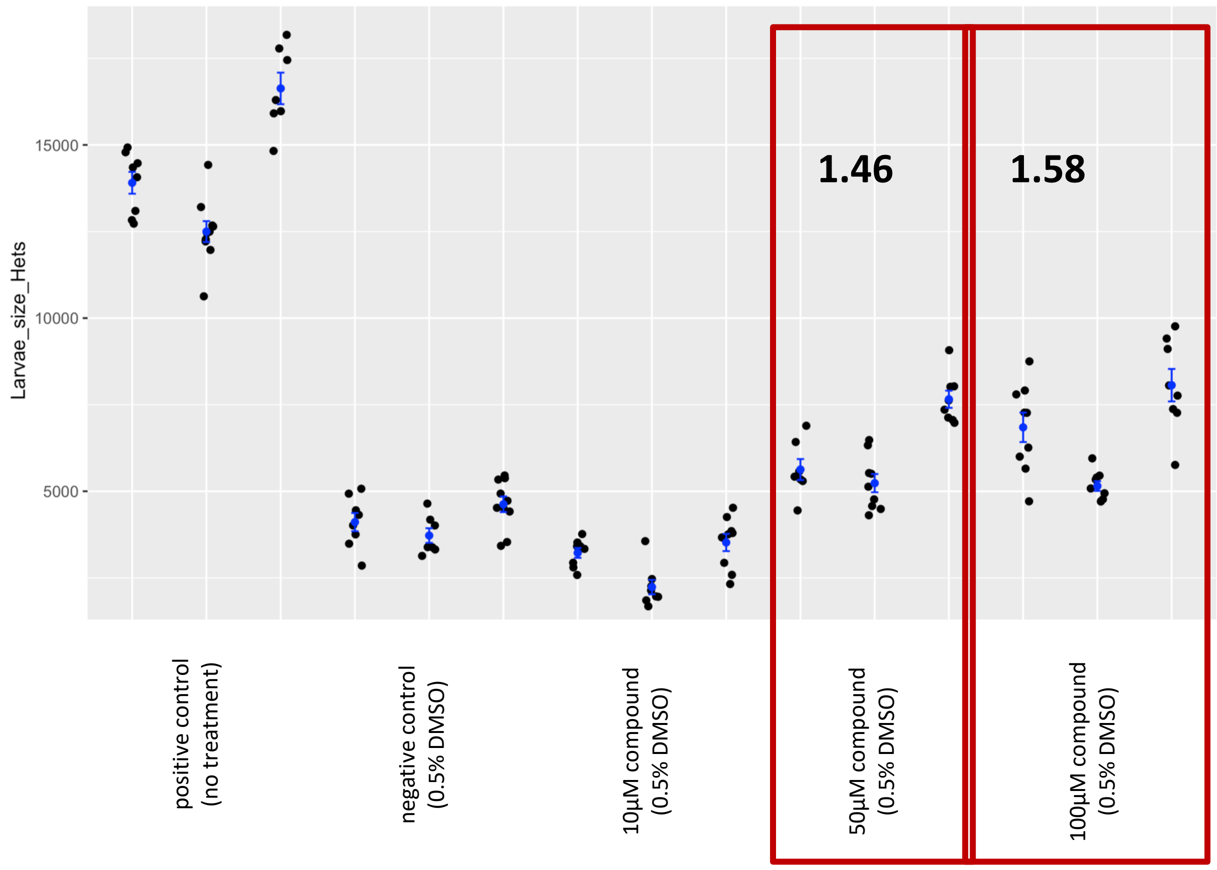

Disease Models & Mechanisms • Supplementary information

Fly Microsource Retest – LUMEFANTRINE

Homozygotes

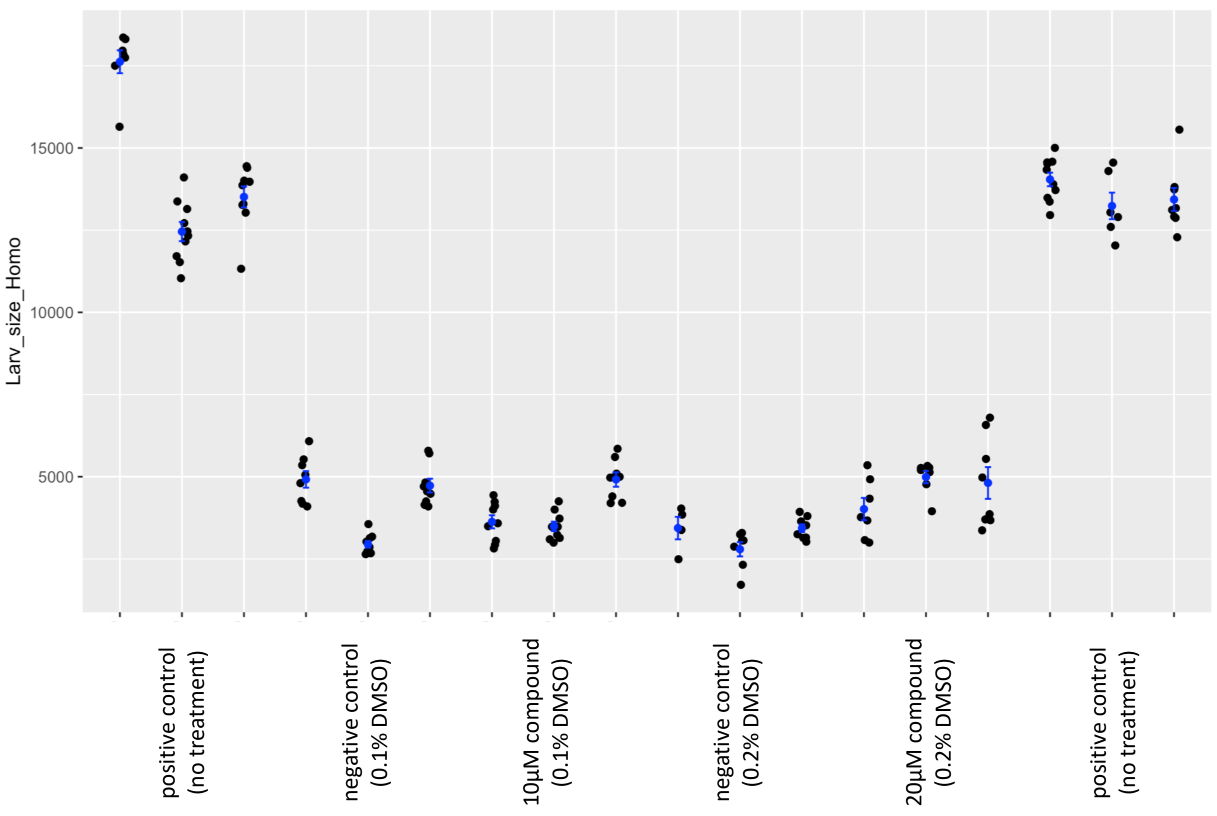

Heterozygotes + bortezomib

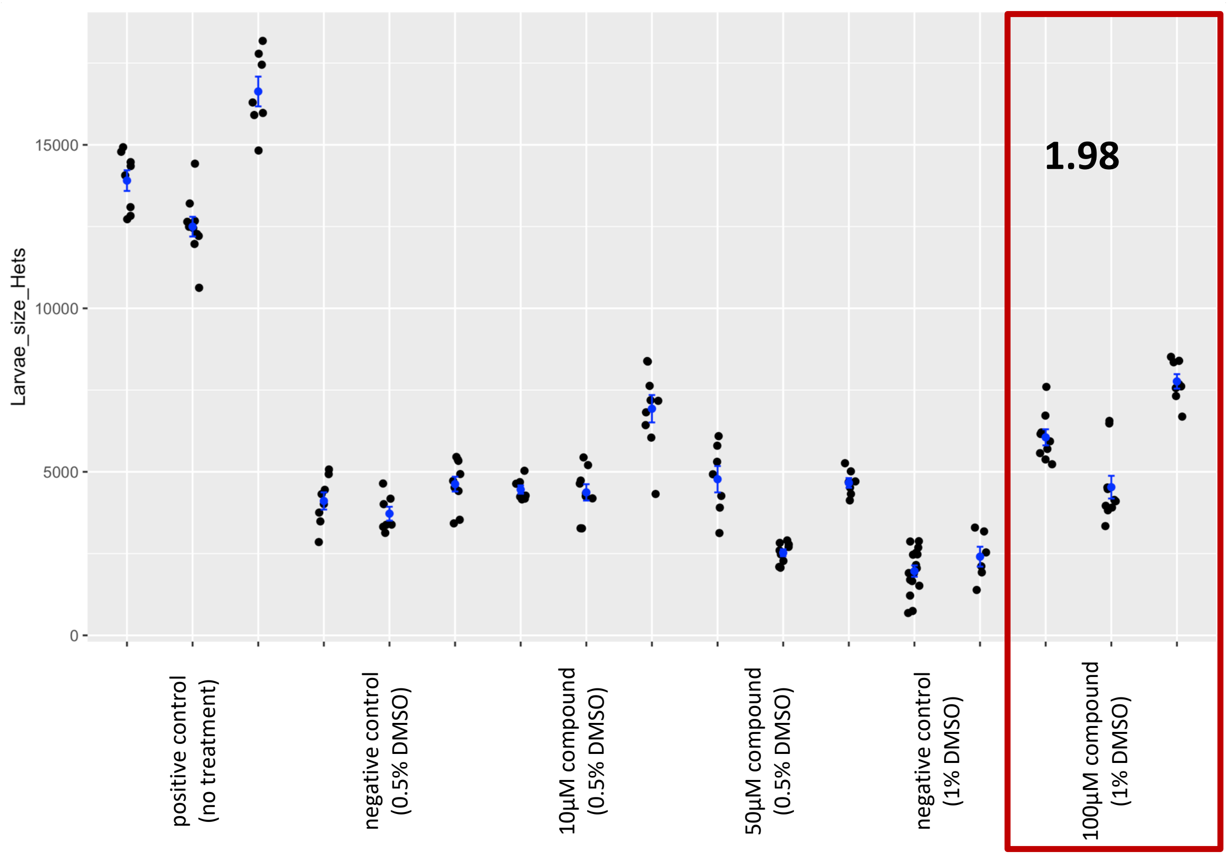

Disease Models & Mechanisms • Supplementary information

Fly Microsource Retest – NICERGOLINE

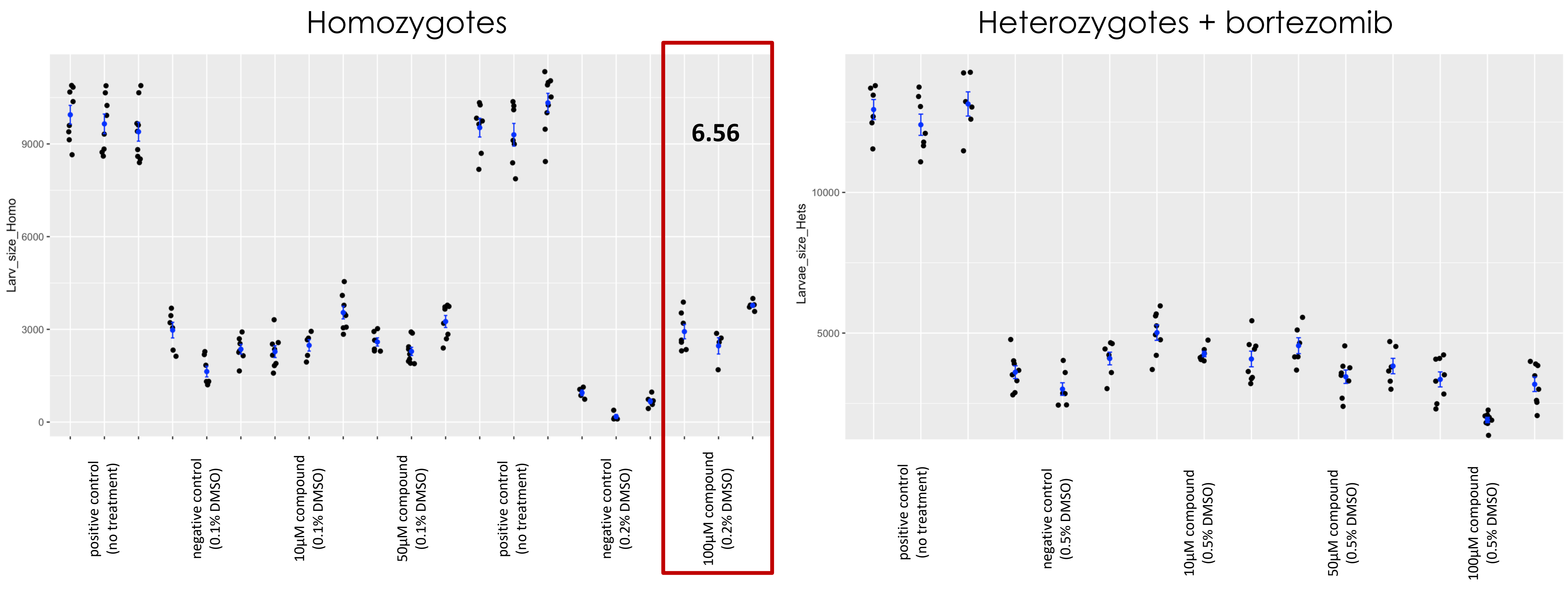

Disease Models & Mechanisms • Supplementary information

Fly Microsource Retest – PIMETHIXENE MALEATE

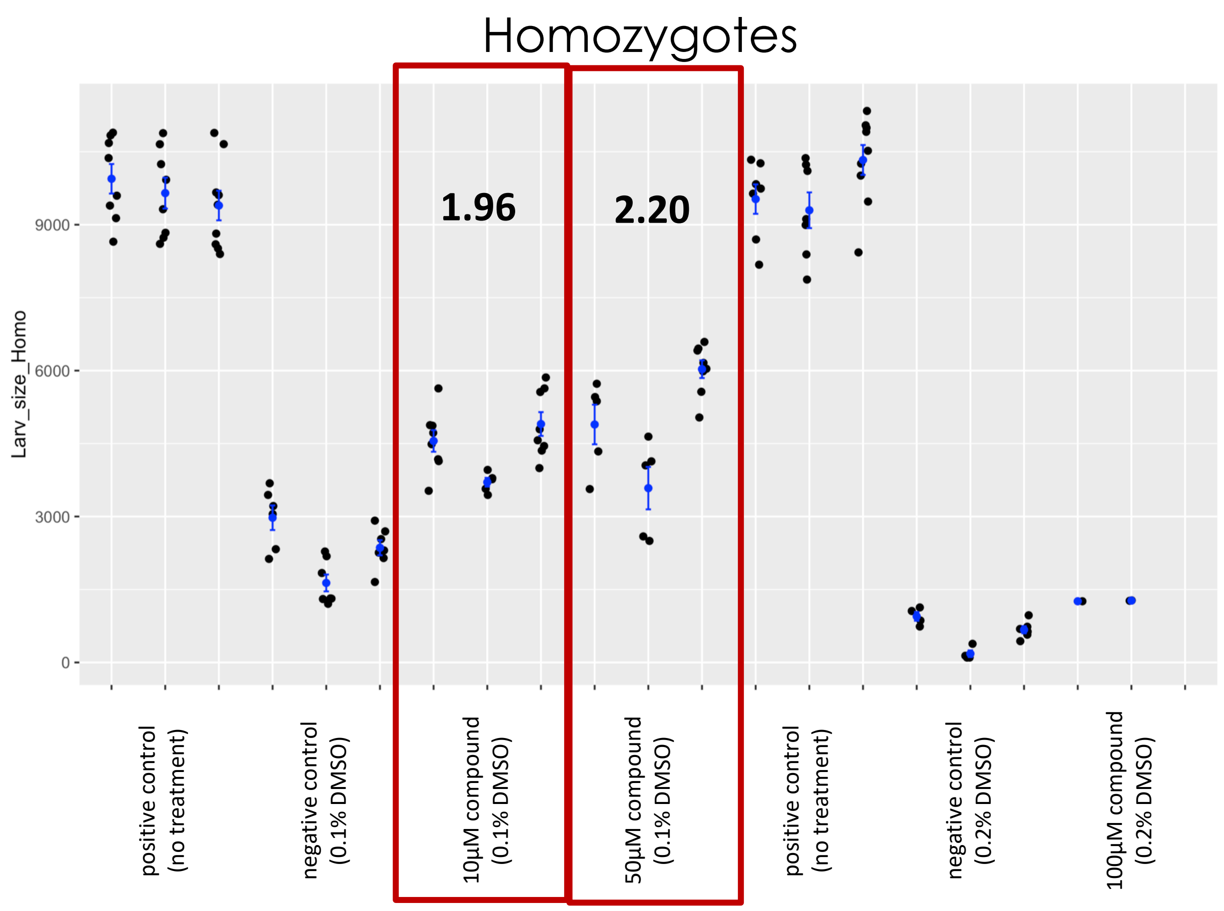

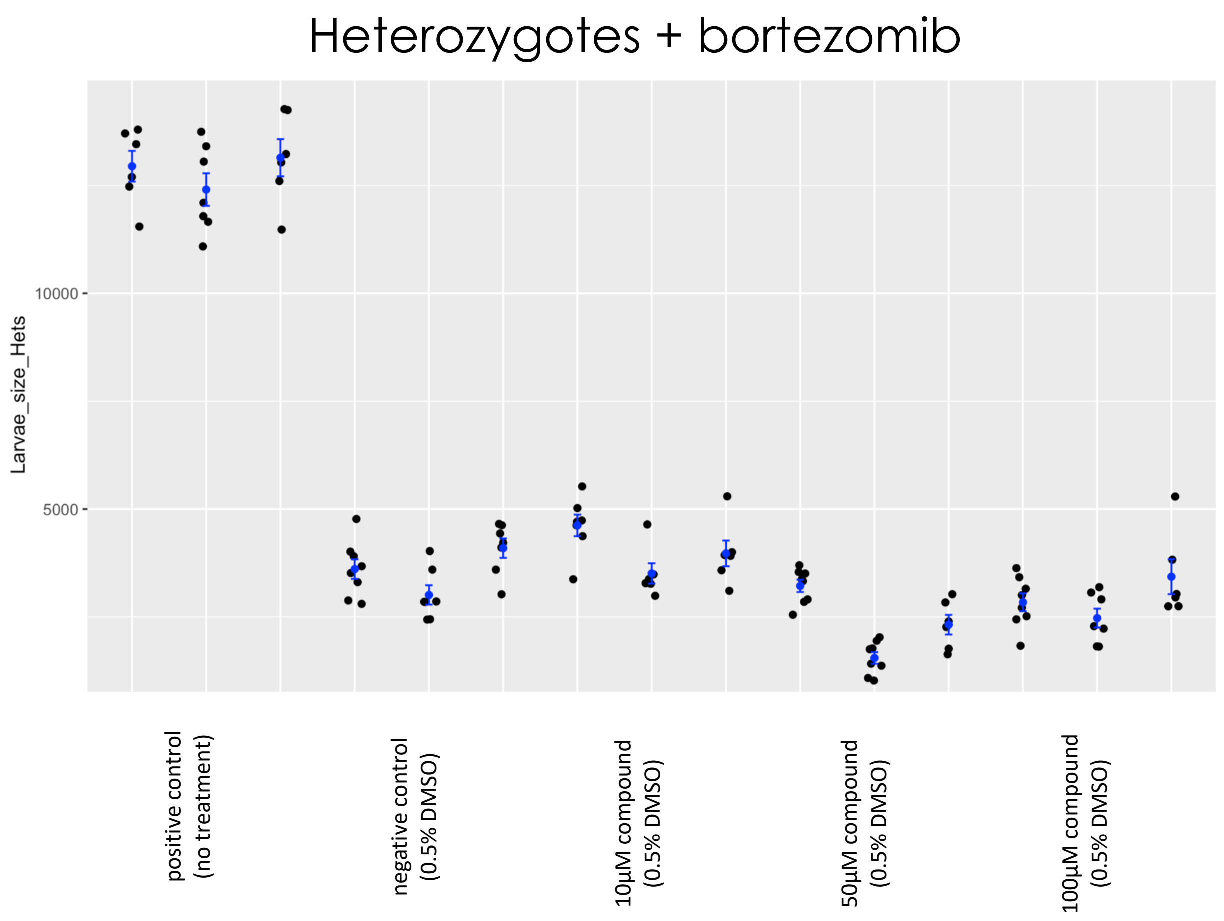

Disease Models & Mechanisms • Supplementary information

Fly Microsource Retest – PIPERINE

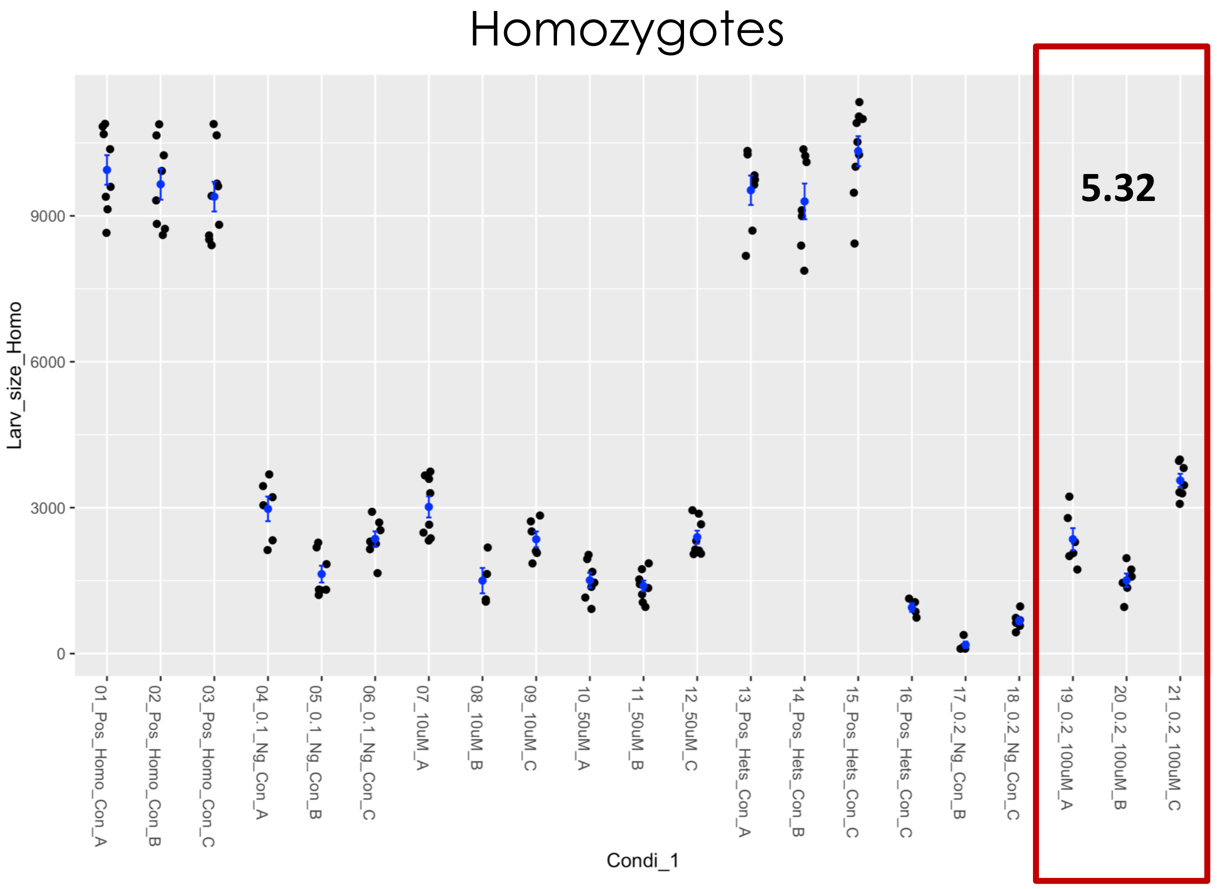

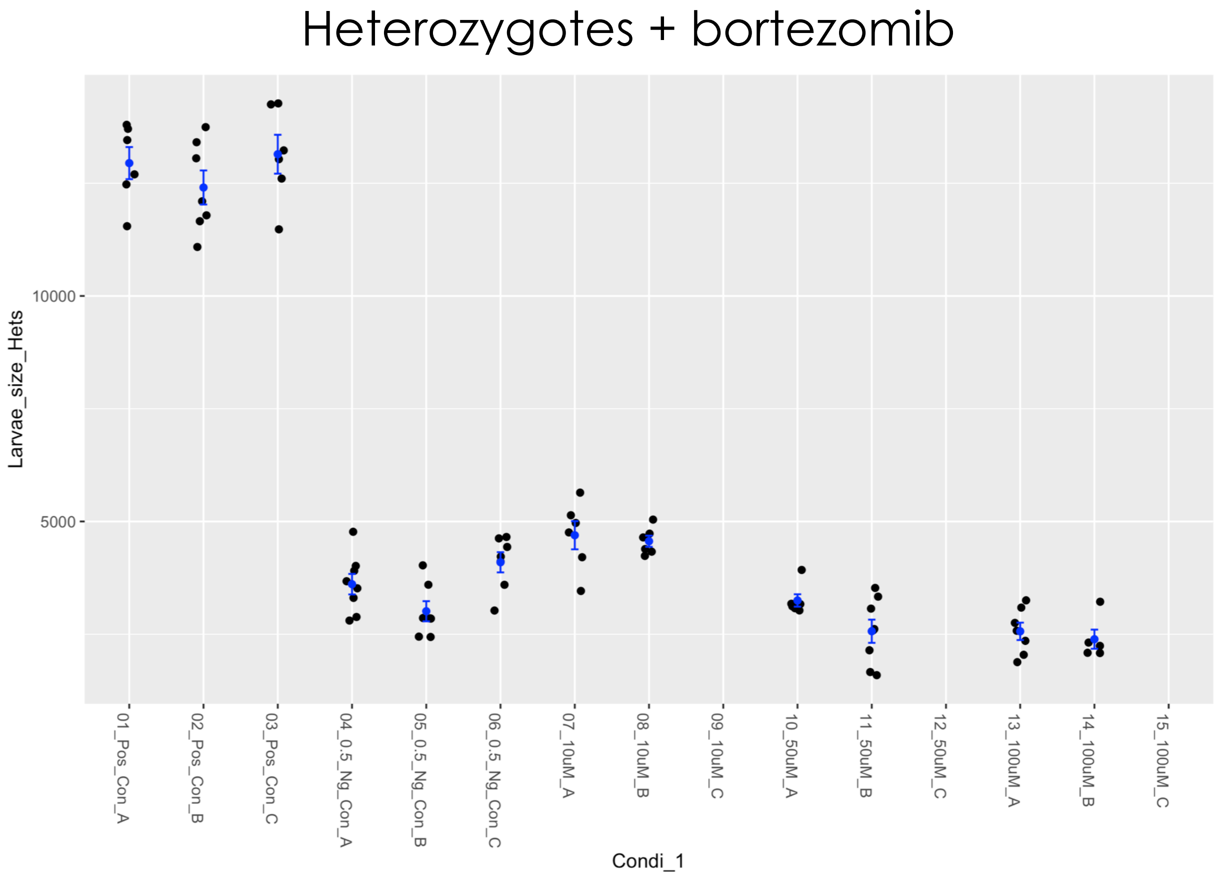

Disease Models & Mechanisms • Supplementary information

Fly Microsource Retest – PRAXADINE HYDROCHLORIDE

Homozygotes

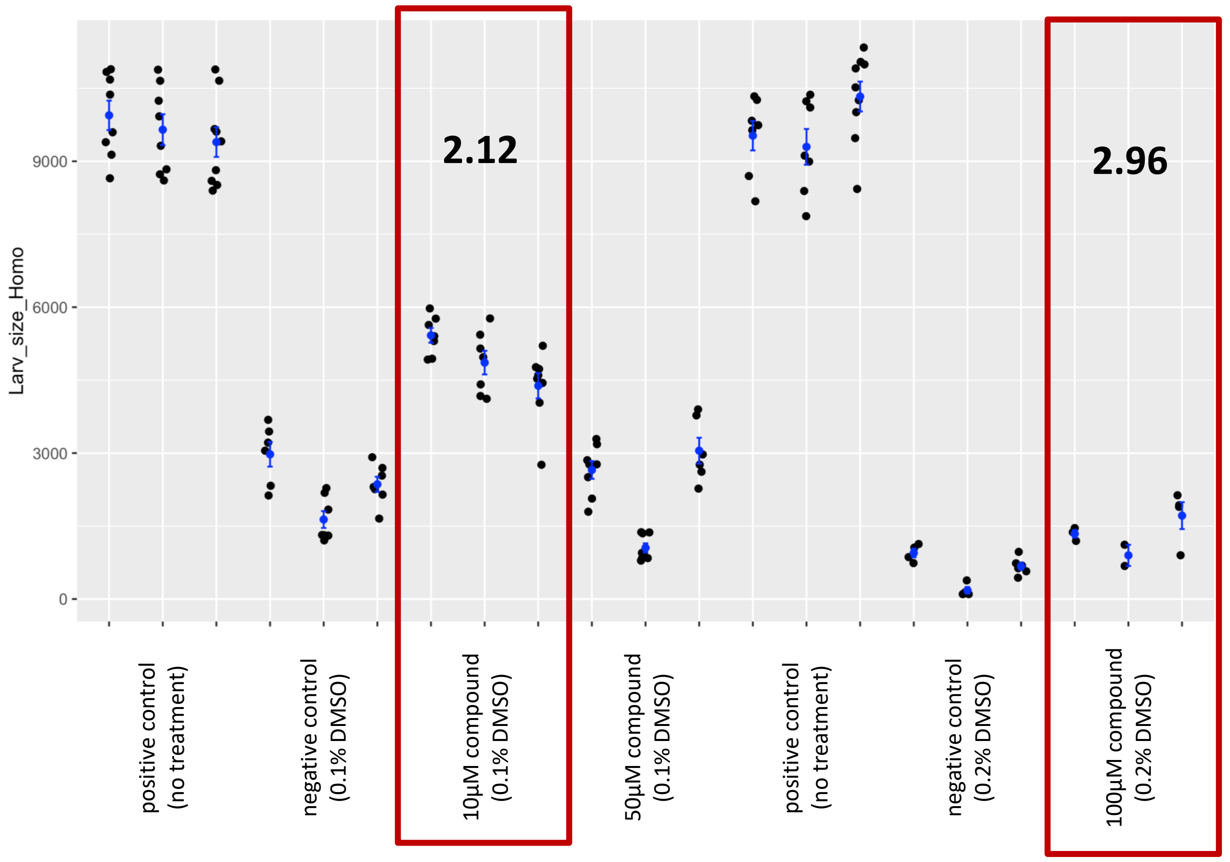

Heterozygotes + bortezomib

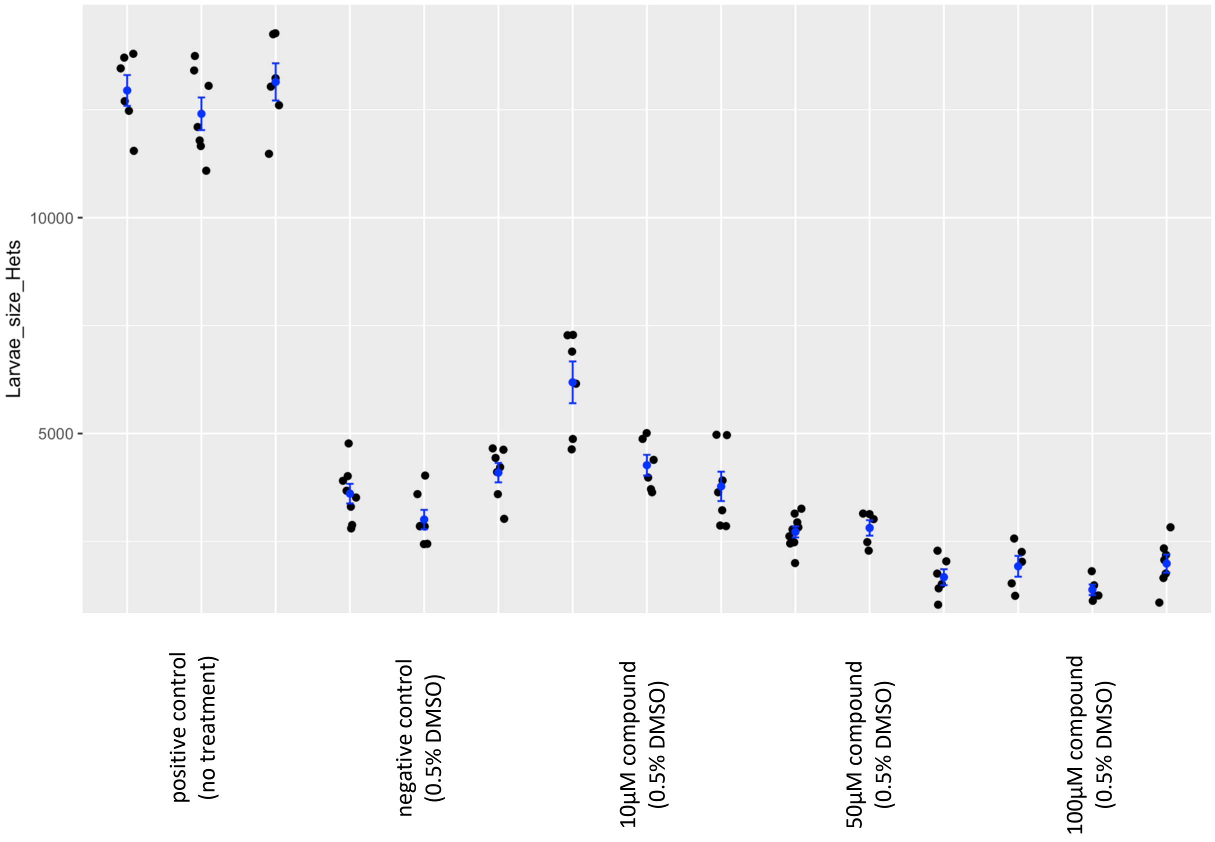

Disease Models & Mechanisms • Supplementary information

Fly Microsource Retest – PROMAZINE HYDROCHLORIDE

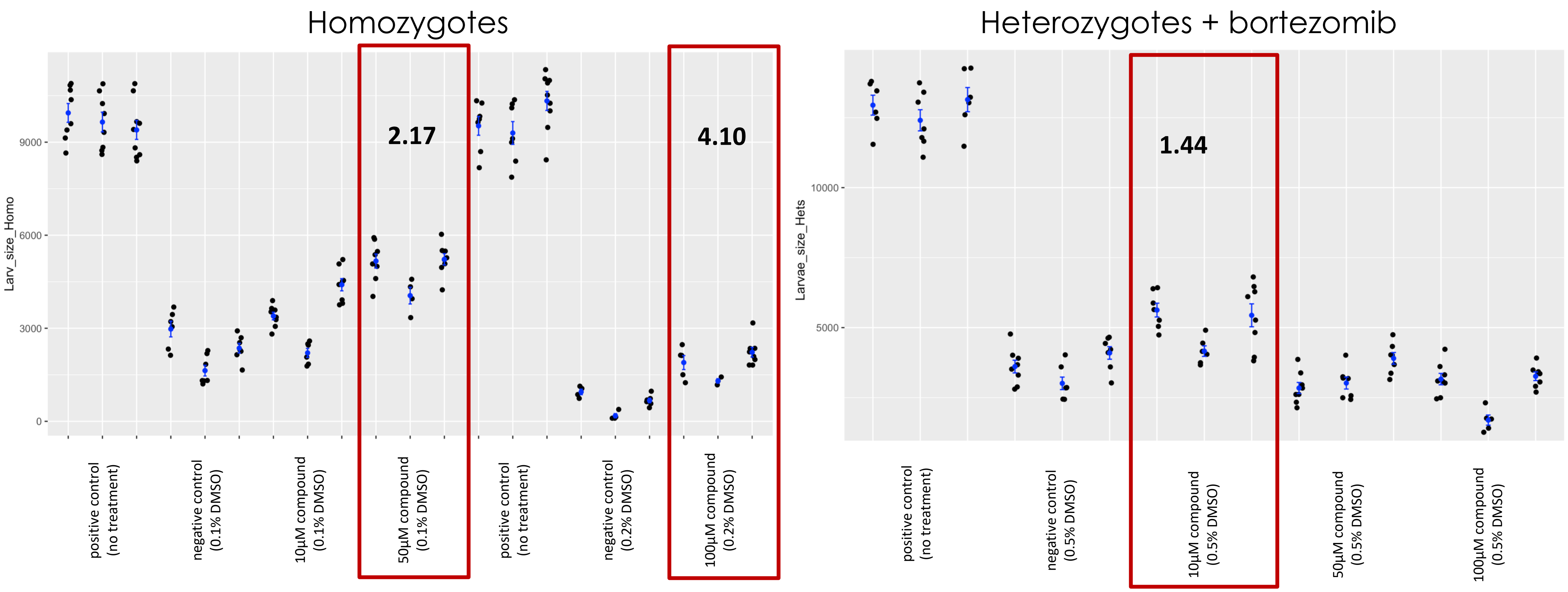

Disease Models & Mechanisms • Supplementary information

Fly Microsource Retest – PYRIMETHAMINE

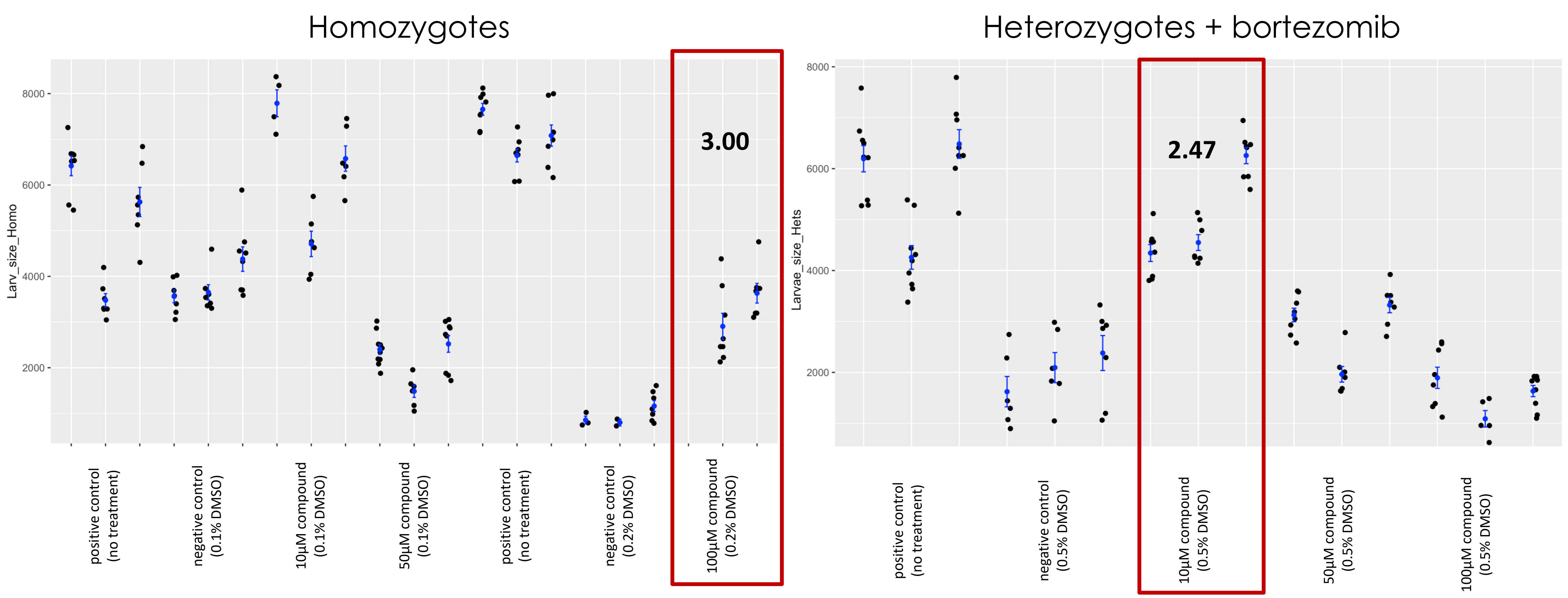

Disease Models & Mechanisms • Supplementary information

Fly Microsource Retest – QUINIZARIN

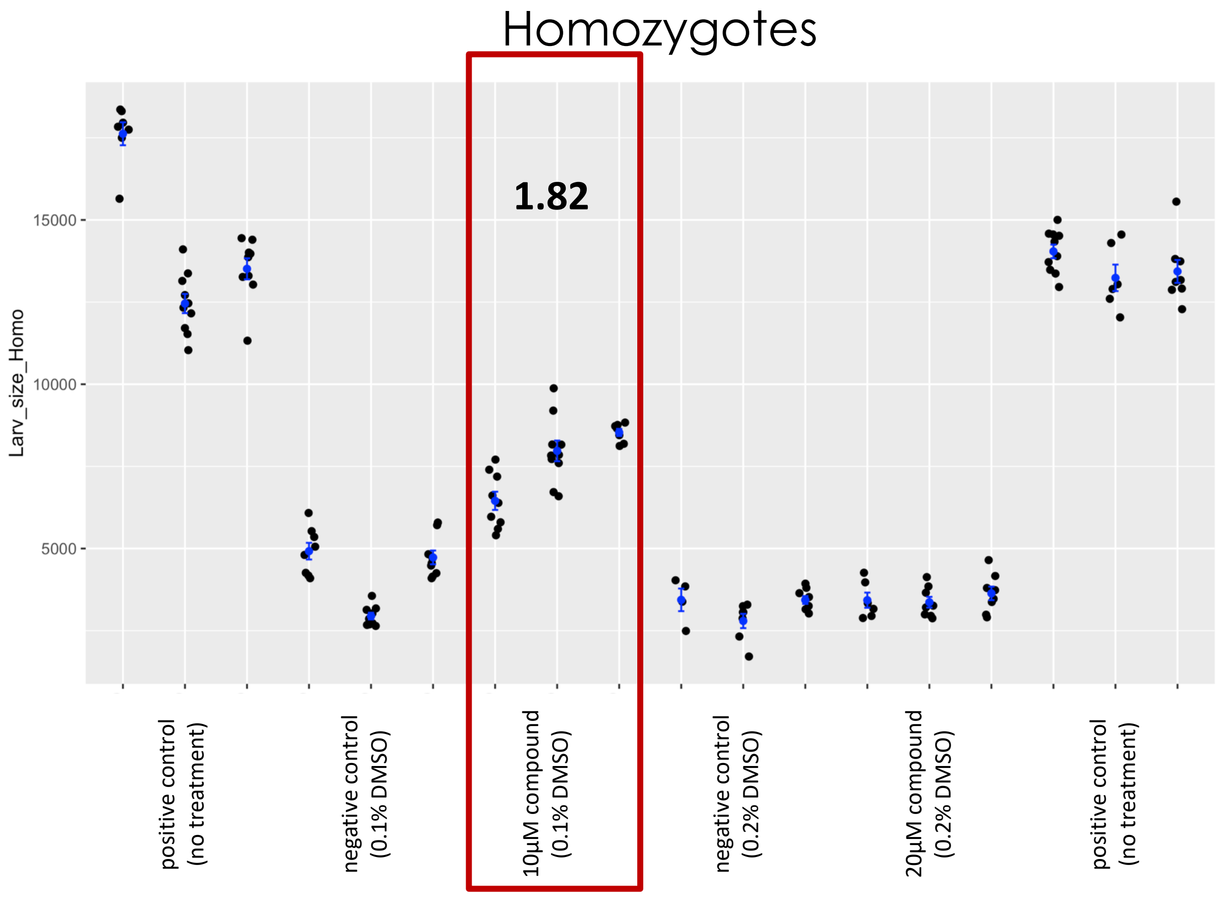

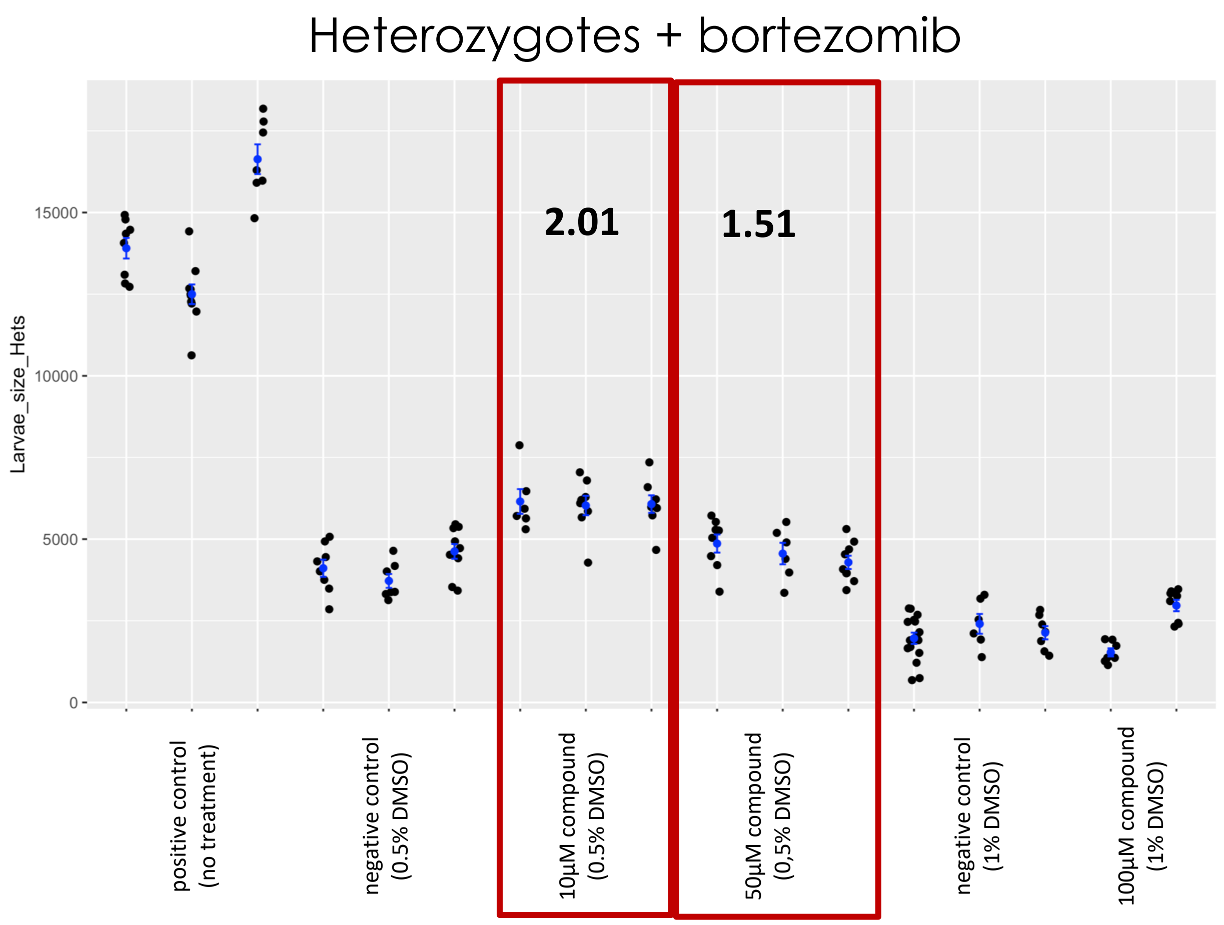

Disease Models & Mechanisms • Supplementary information

Disease Models & Mechanisms: doi:10.1242/dmm.040576: Supplementary information

Fly Microsource Retest – RHETSININE

Homozygotes

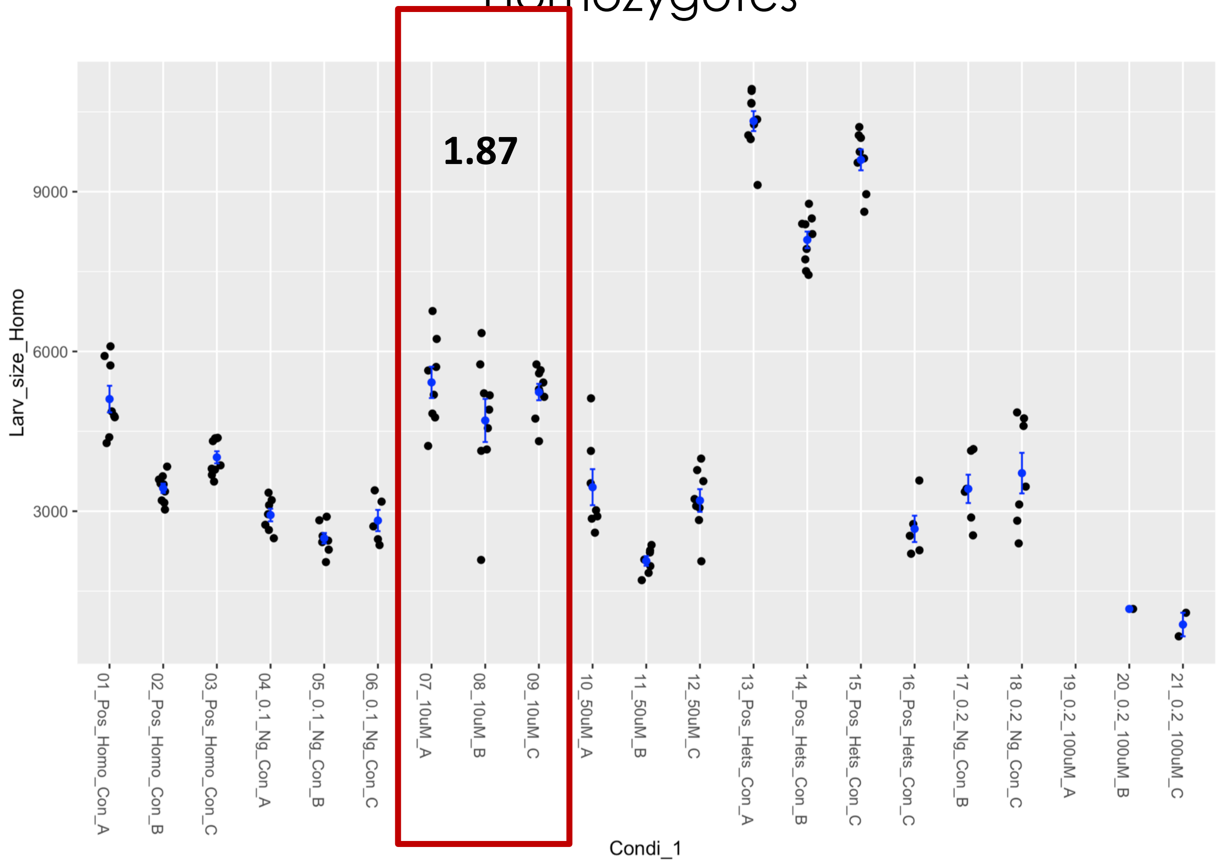

Heterozygotes + bortezomib

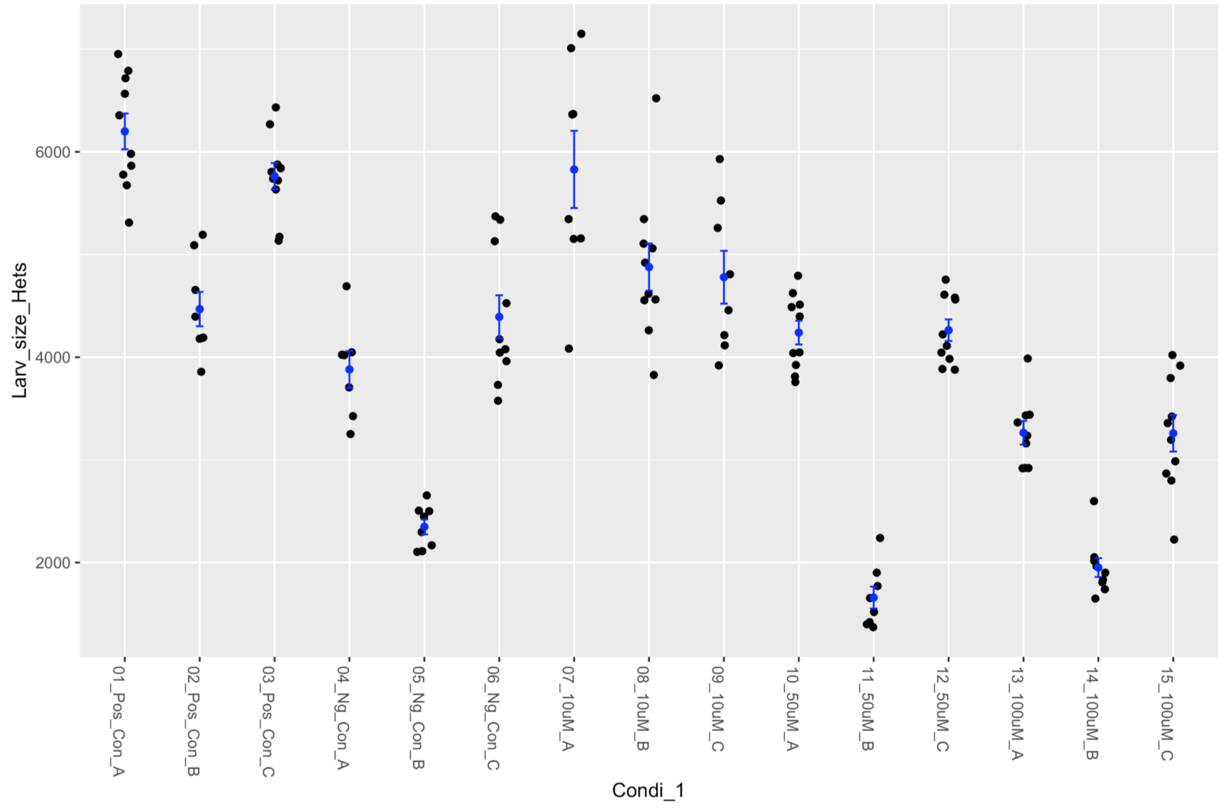

Disease Models & Mechanisms • Supplementary information

Fly GSF tool compound test – SULFORAPHANE

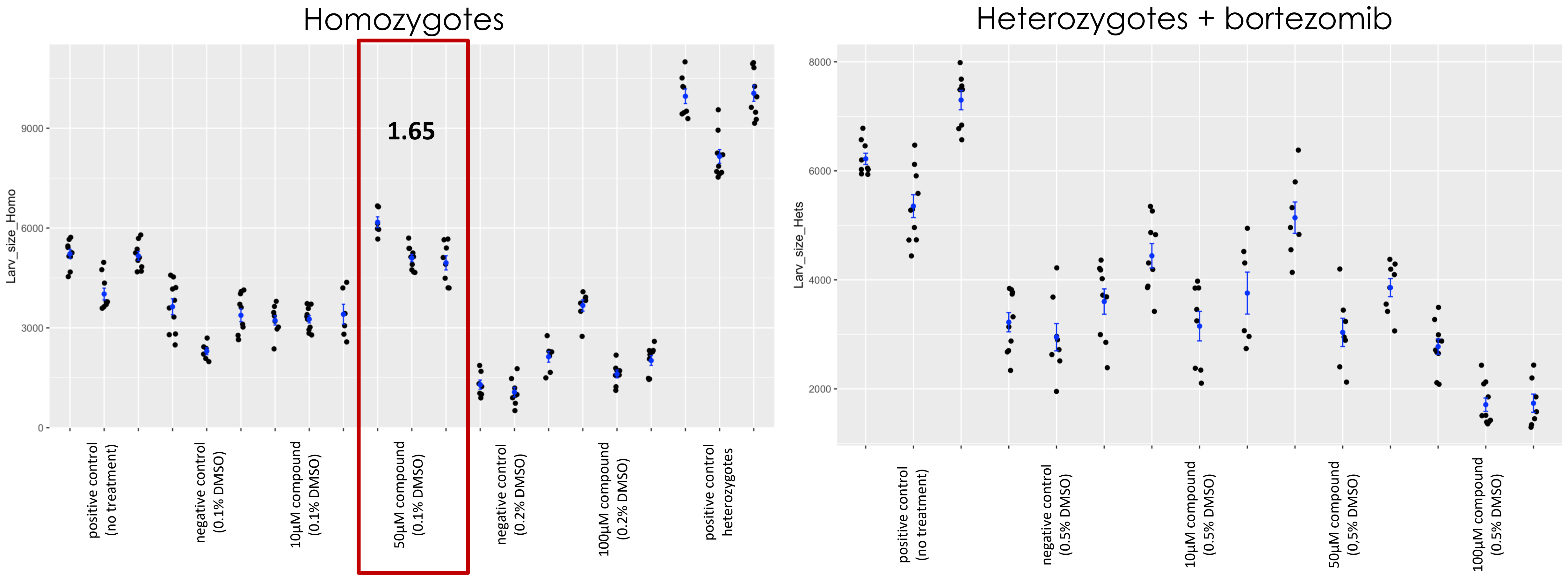

Disease Models & Mechanisms • Supplementary information

Fly Microsource retest – TANNIC ACID

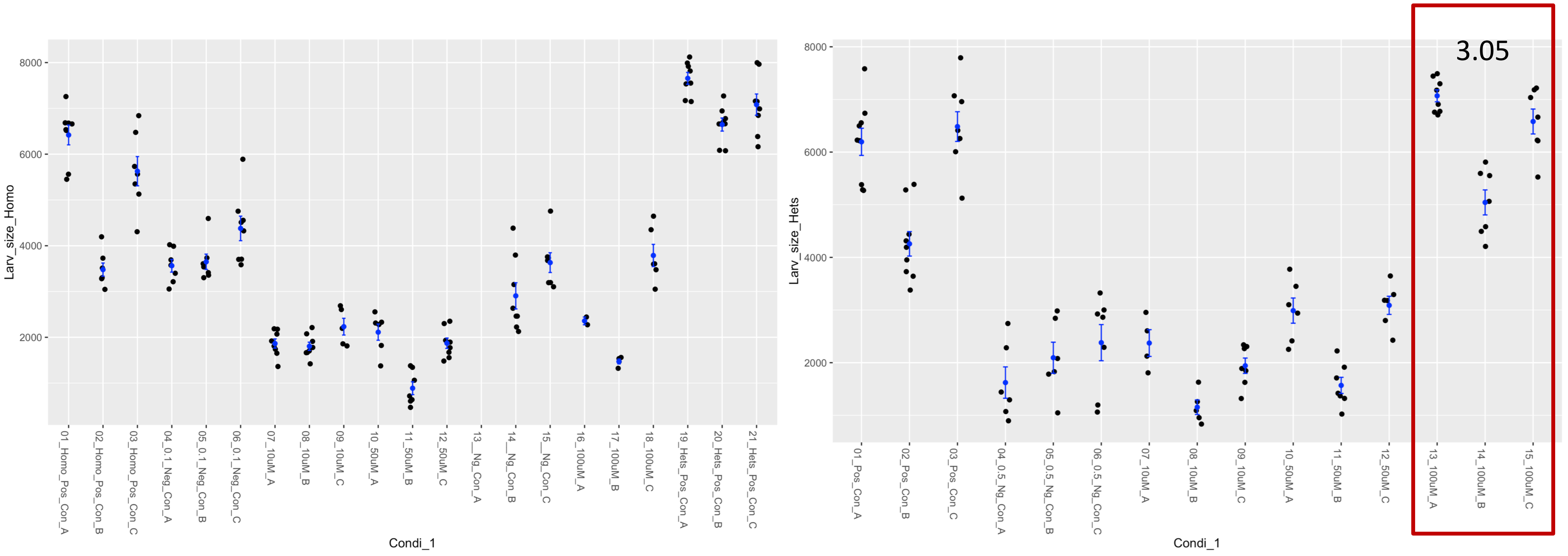

Disease Models & Mechanisms • Supplementary information

Fly Microsource Retest – THIOGUANOSINE

Homozygotes

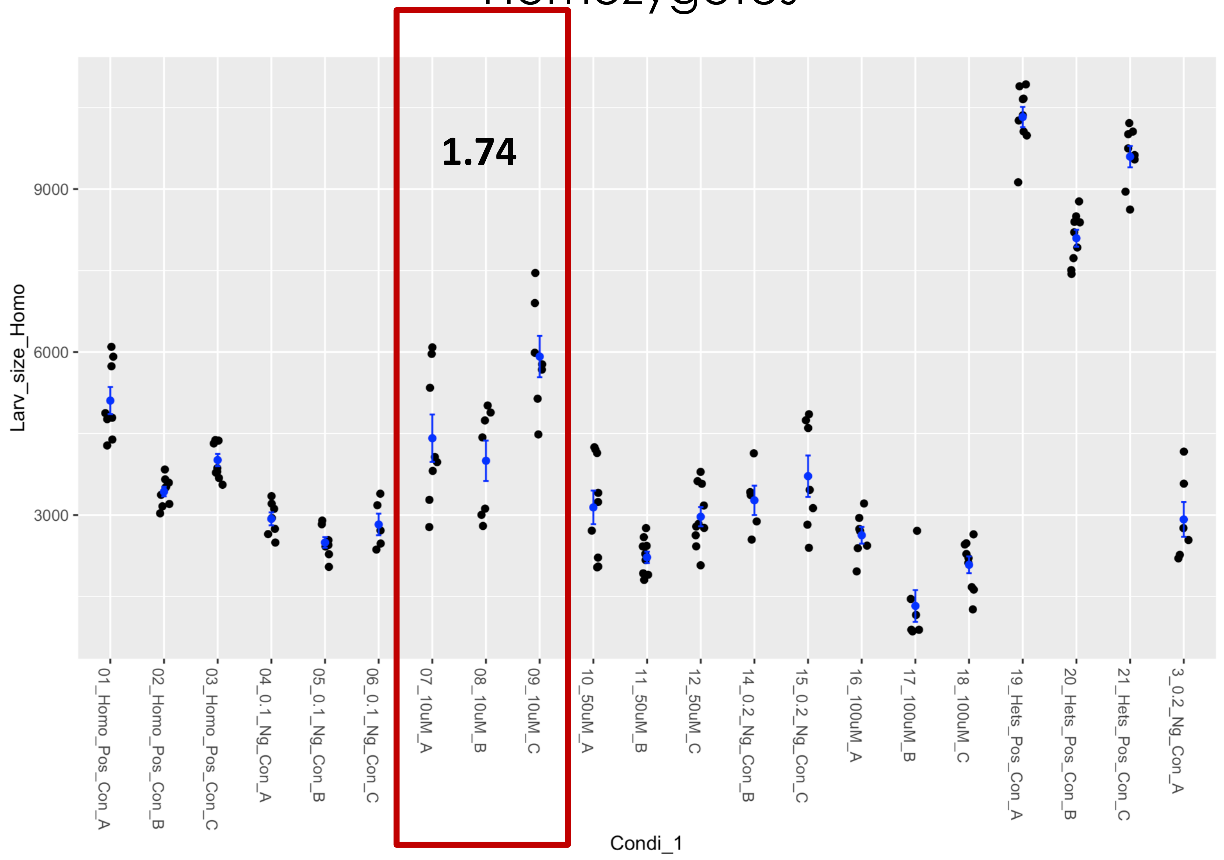

Heterozygotes + bortezomib

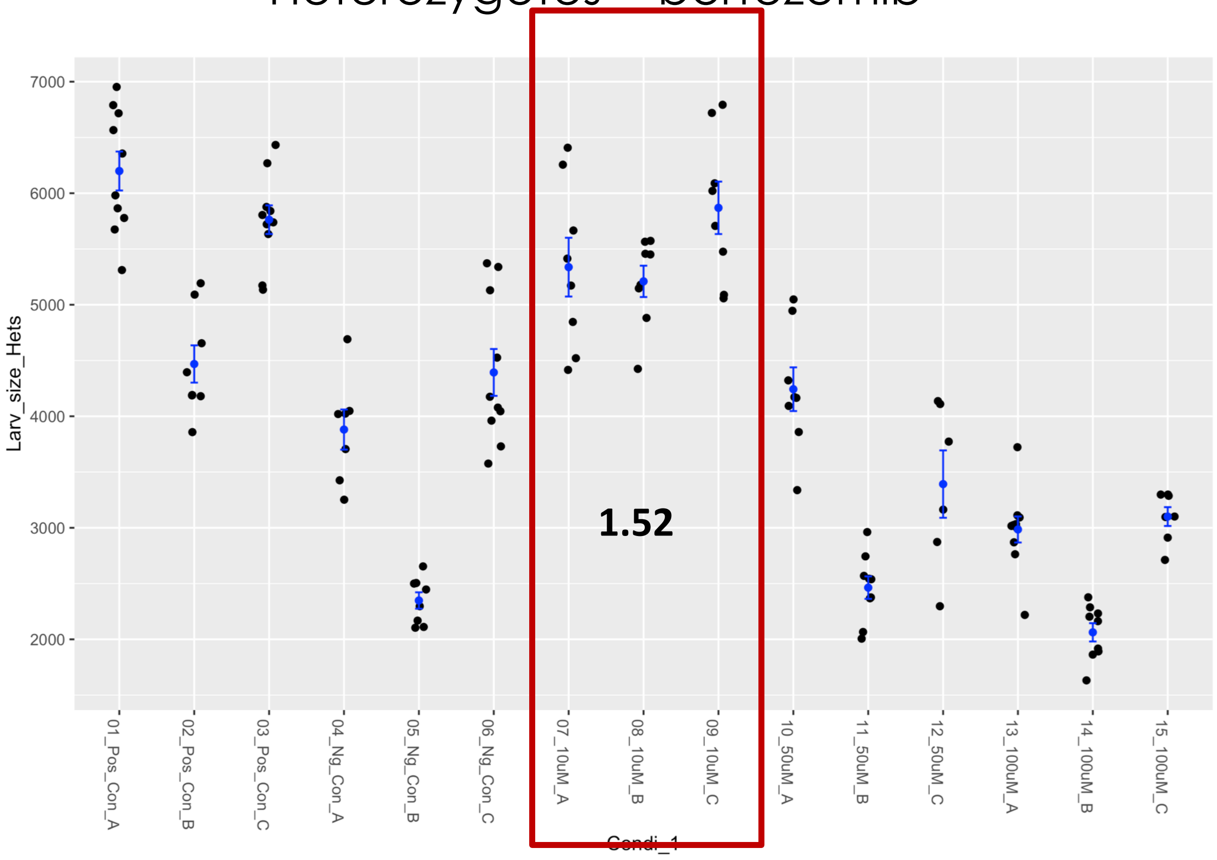

Disease Models & Mechanisms • Supplementary information

Fly Microsource retest – TROLOX

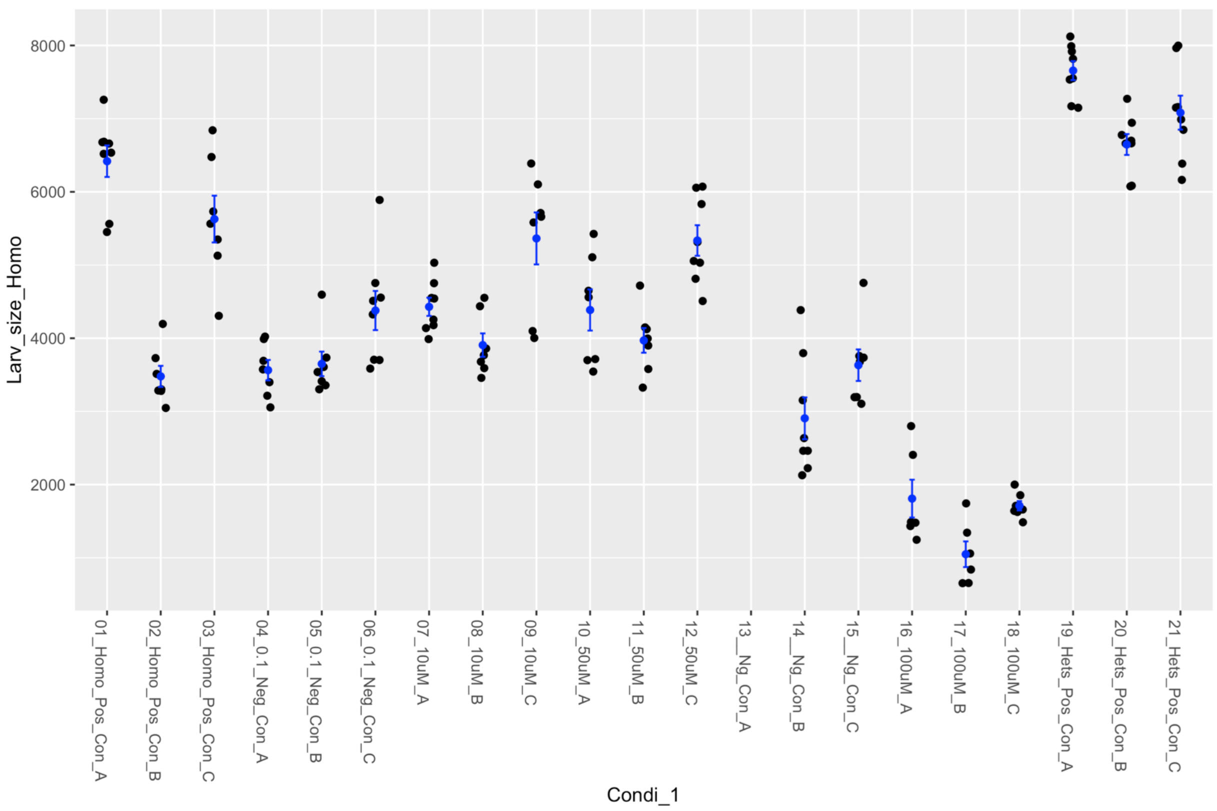

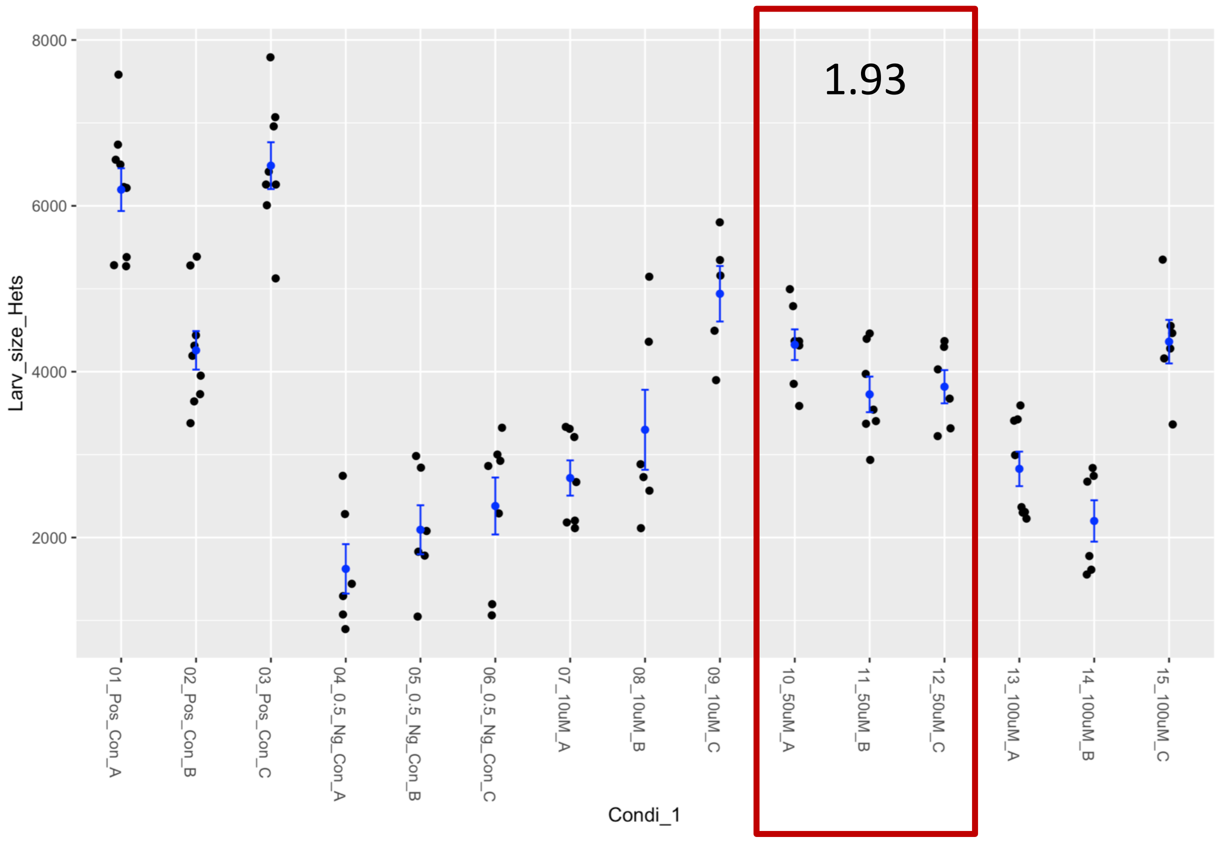

Disease Models & Mechanisms • Supplementary information

Fly Worm MS cross validation

All compounds that scored as hits in the worm *png-1* Microsource Spectrum (drug repurposing) screen were cross-validated in two fly secondary assays: rescue of *Pngl*$^{-/-}$ homozygotes and rescue of bortezomib-treated *Pngl*$^{+/-}$ heterozygotes. Red boxes indicate compounds that successfully retested. The number indicates the size ratio of compound-treated fly larvae to untreated control homozygous fly larvae.

(Please note that all homozygote experiments in this section included an untreated heterozygote control)

Disease Models & Mechanisms: doi:10.1242/dmm.040576: Supplementary information

Fly WormMS cross validation –
3,4-DIDESMETHYL-5-DESHYDROXY-3'-ETHOXYSCLEROIN

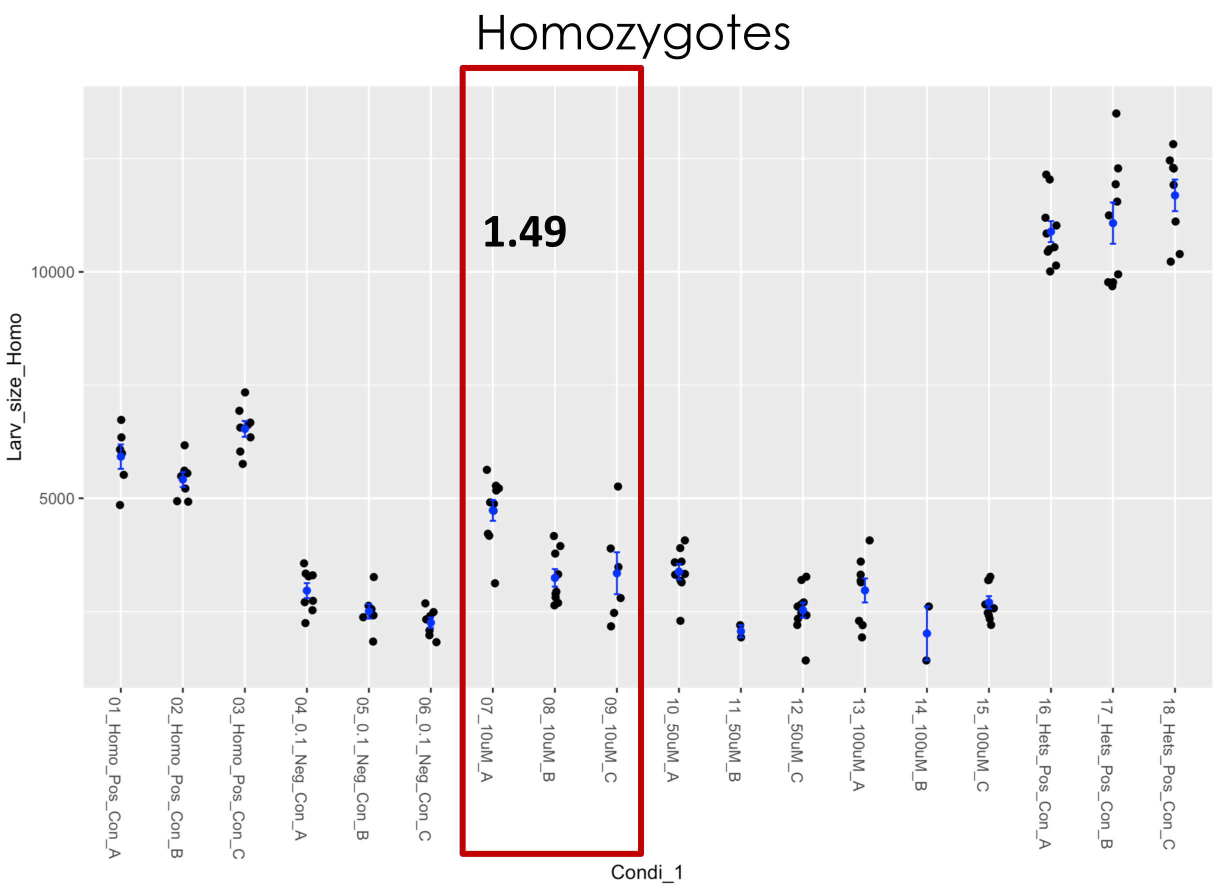

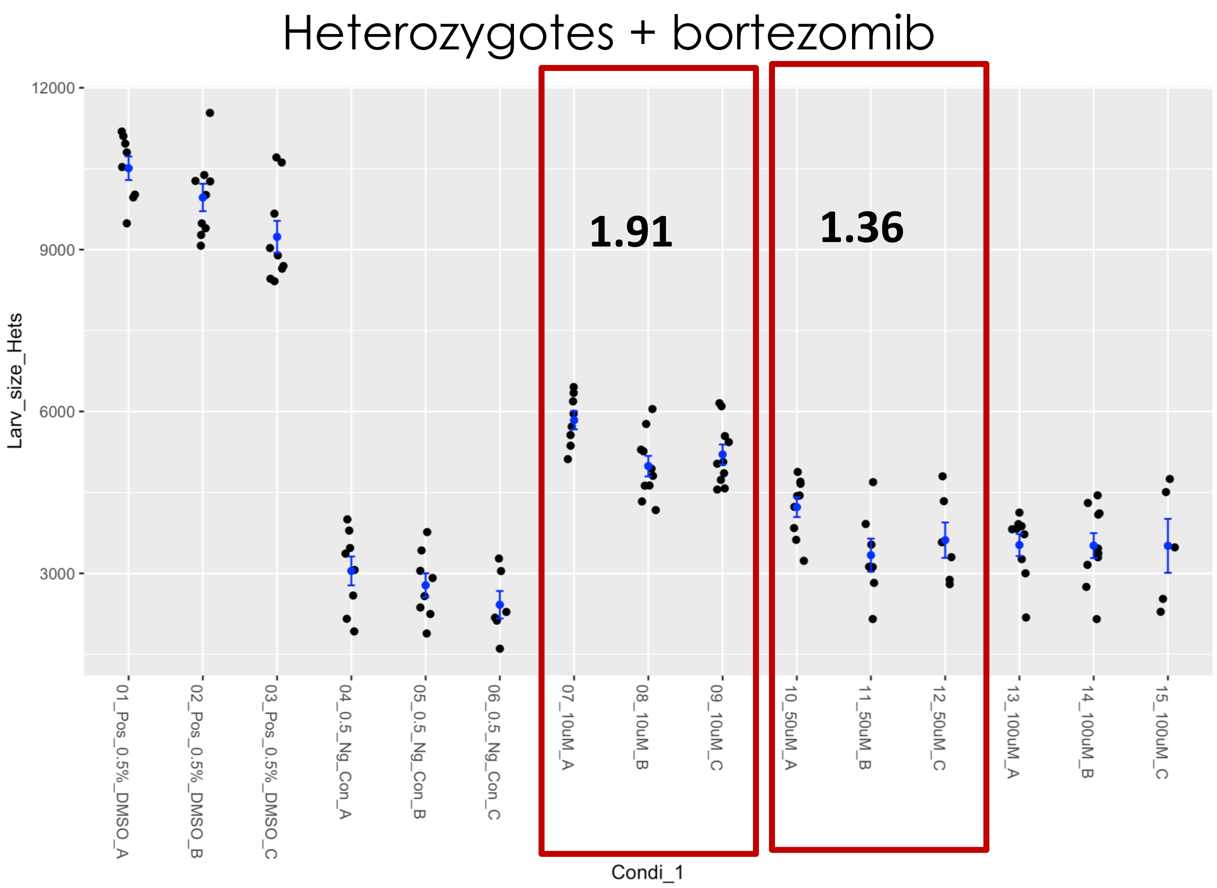

Disease Models & Mechanisms • Supplementary information

Fly WormMS cross validation – BENSERAZIDE

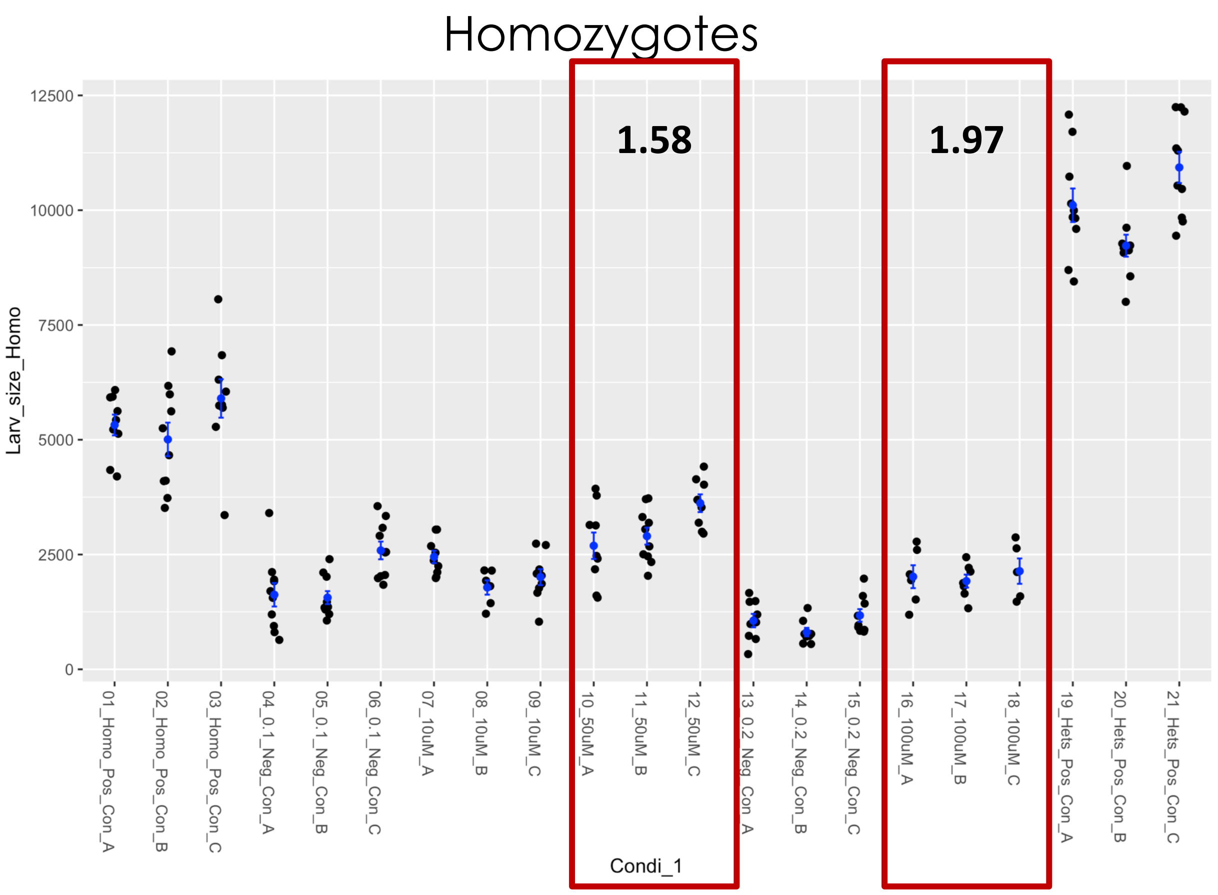

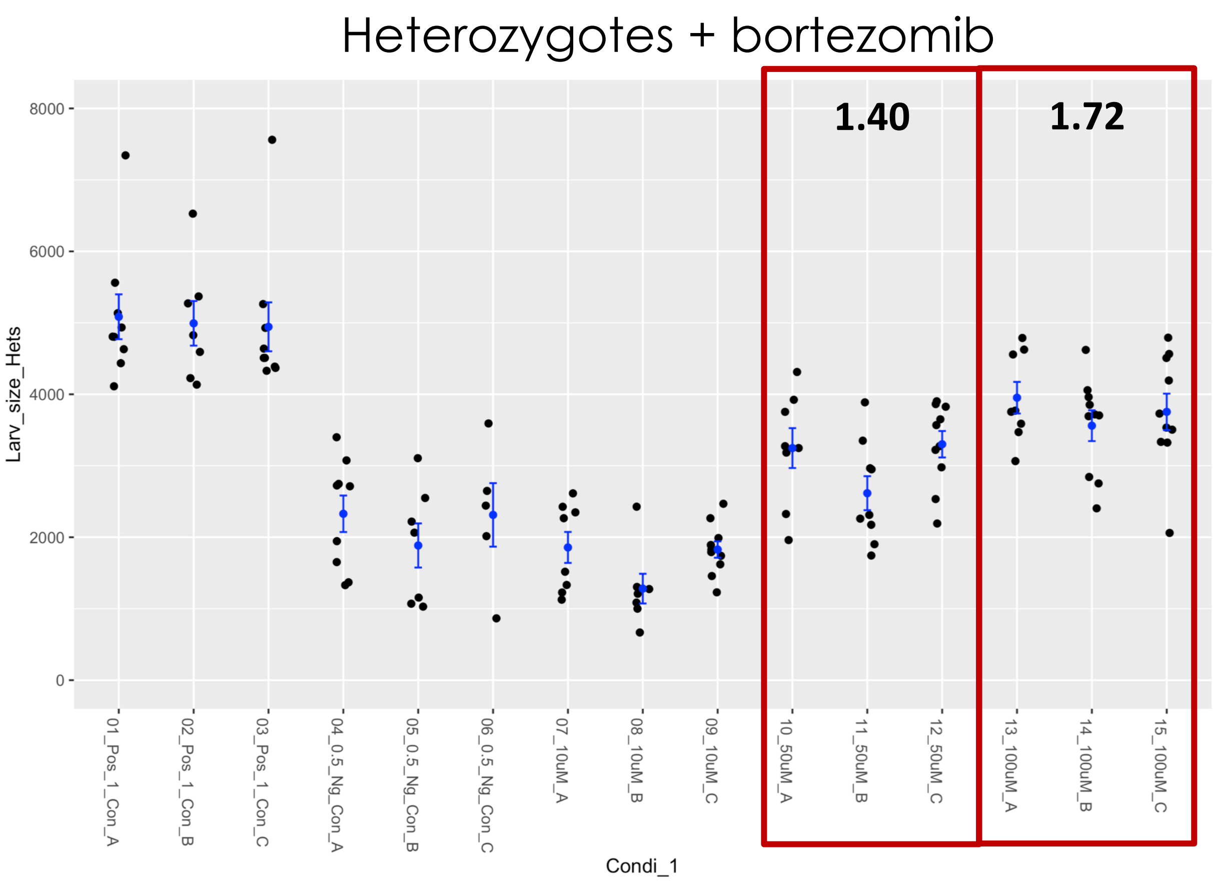

Disease Models & Mechanisms • Supplementary information

Disease Models & Mechanisms: doi:10.1242/dmm.040576: Supplementary information

Fly WormMS cross validation – ELLAGIC ACID

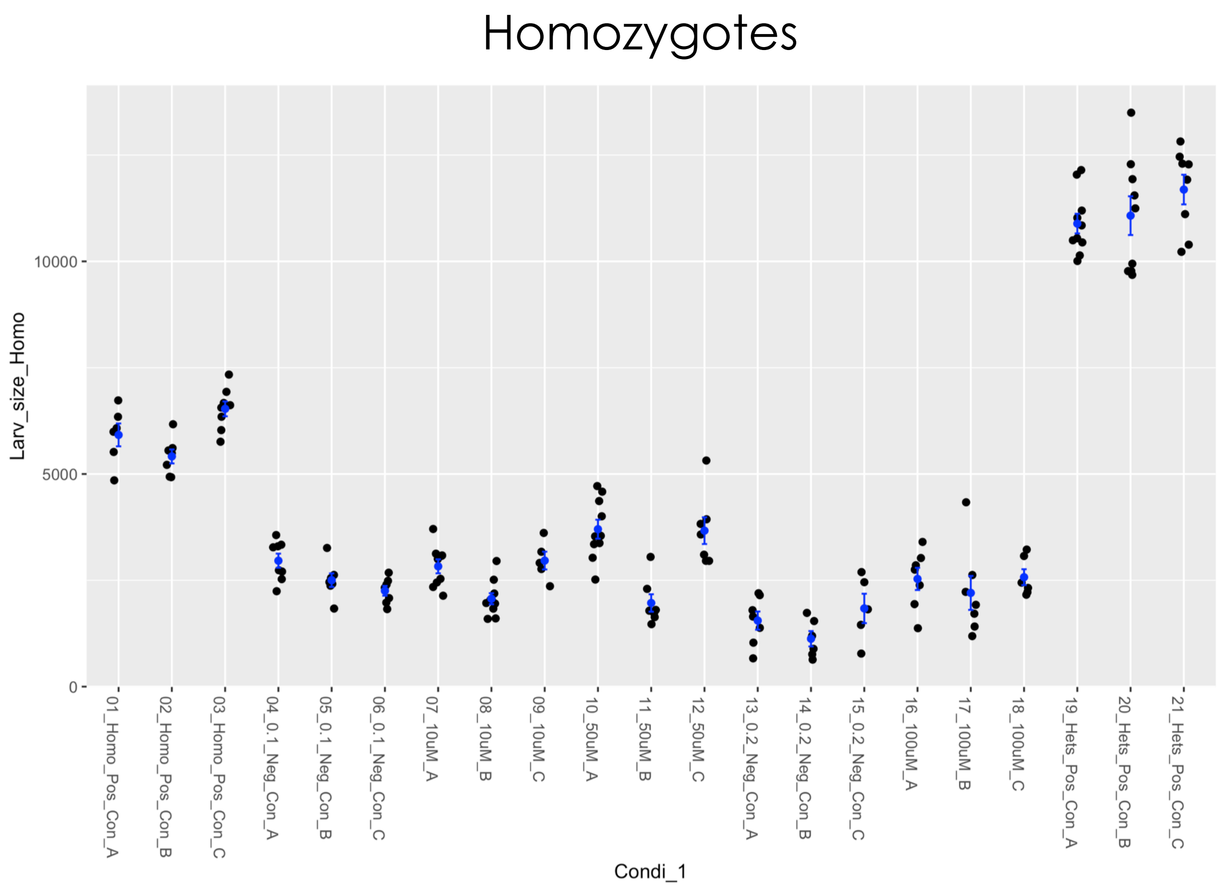

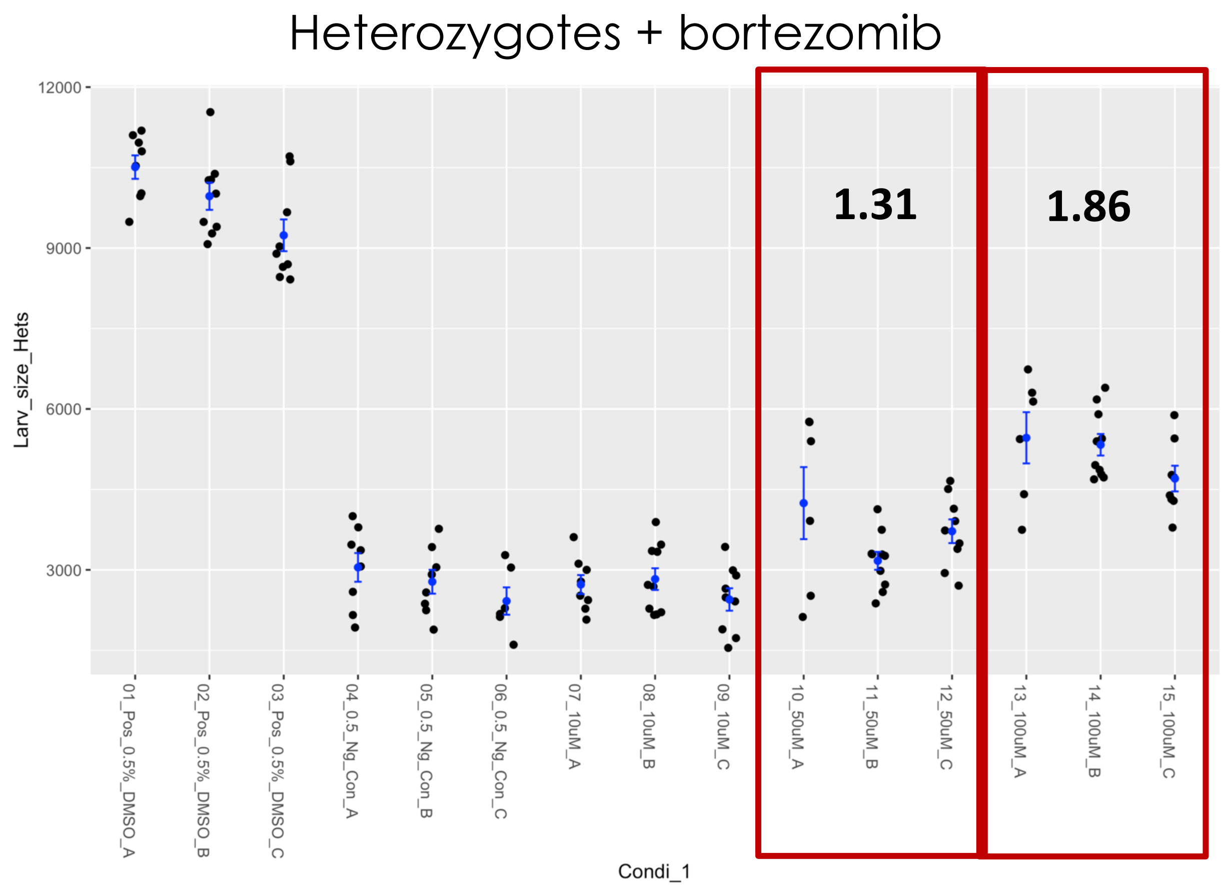

Disease Models & Mechanisms • Supplementary information

Fly WormMS cross validation – EPICATECHIN MONOGALLATE

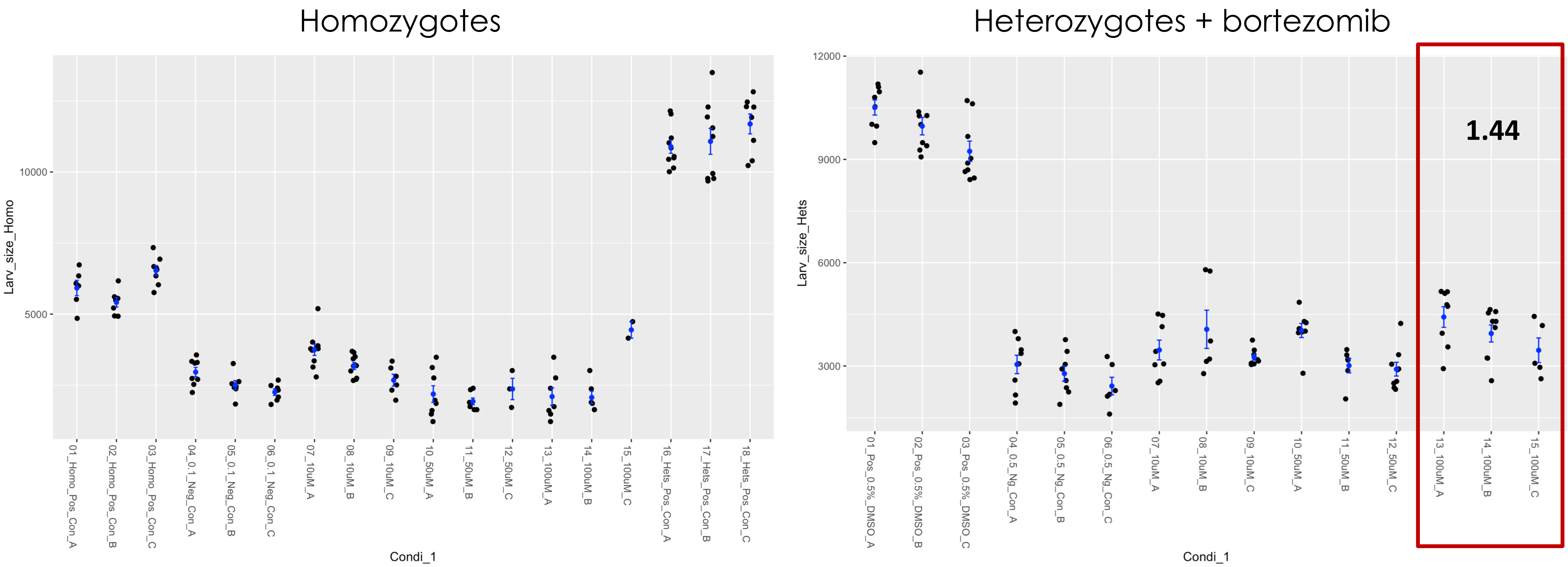

Disease Models & Mechanisms • Supplementary information

Fly WormMS cross validation – KOPARIN

Homozygotes

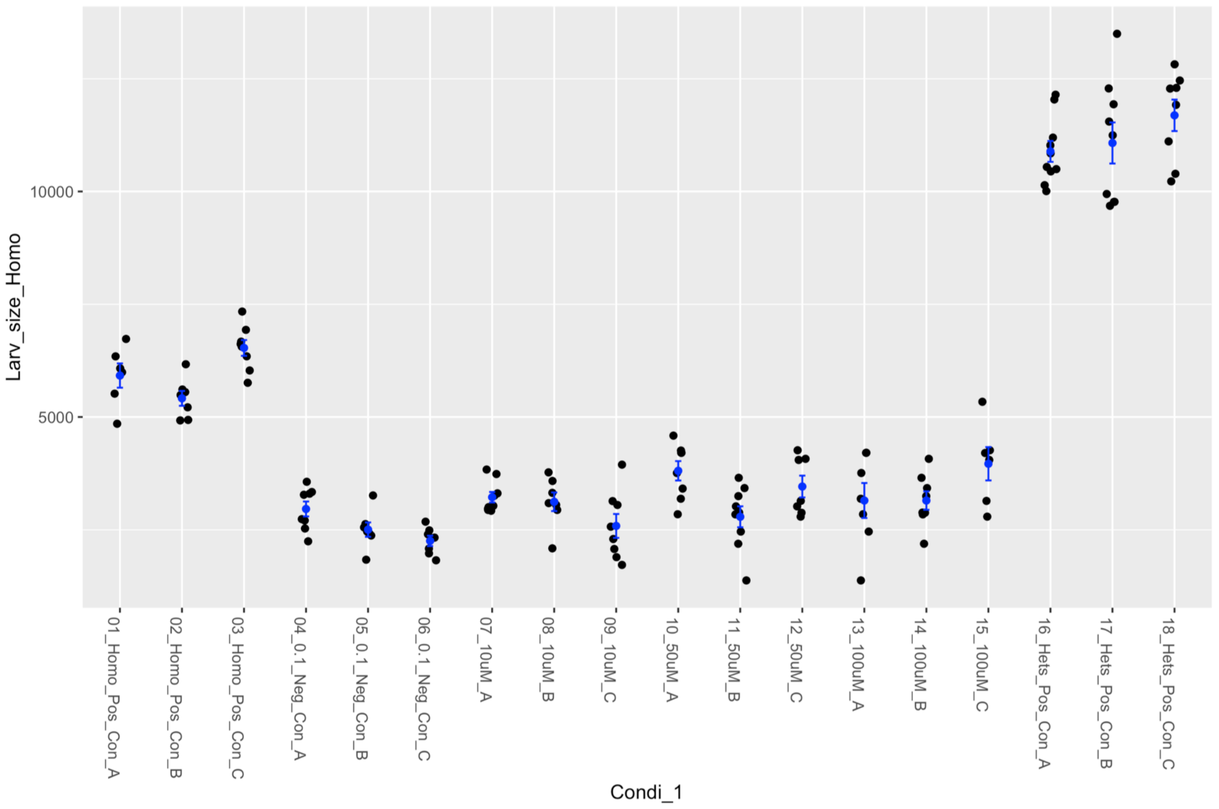

Heterozygotes + bortezomib

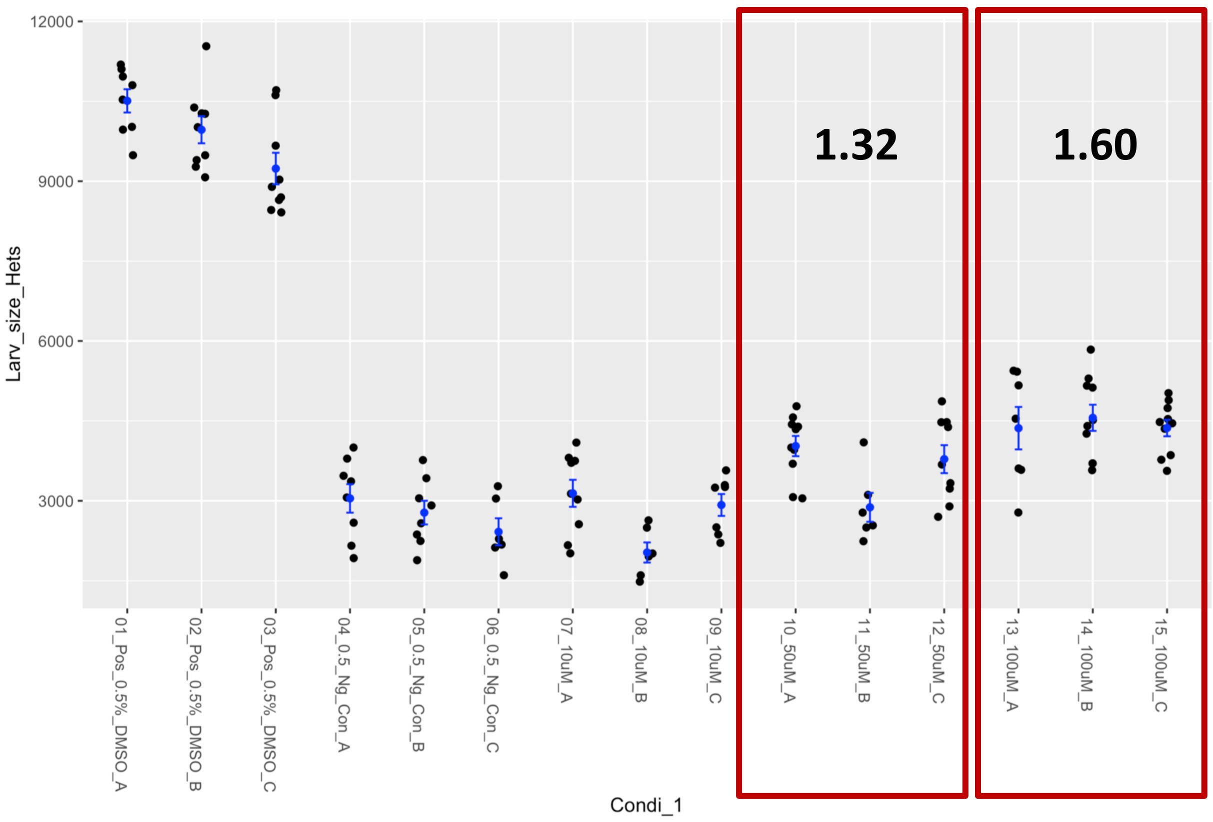

Fly WormMS cross validation – PHENYLBUTAZONE

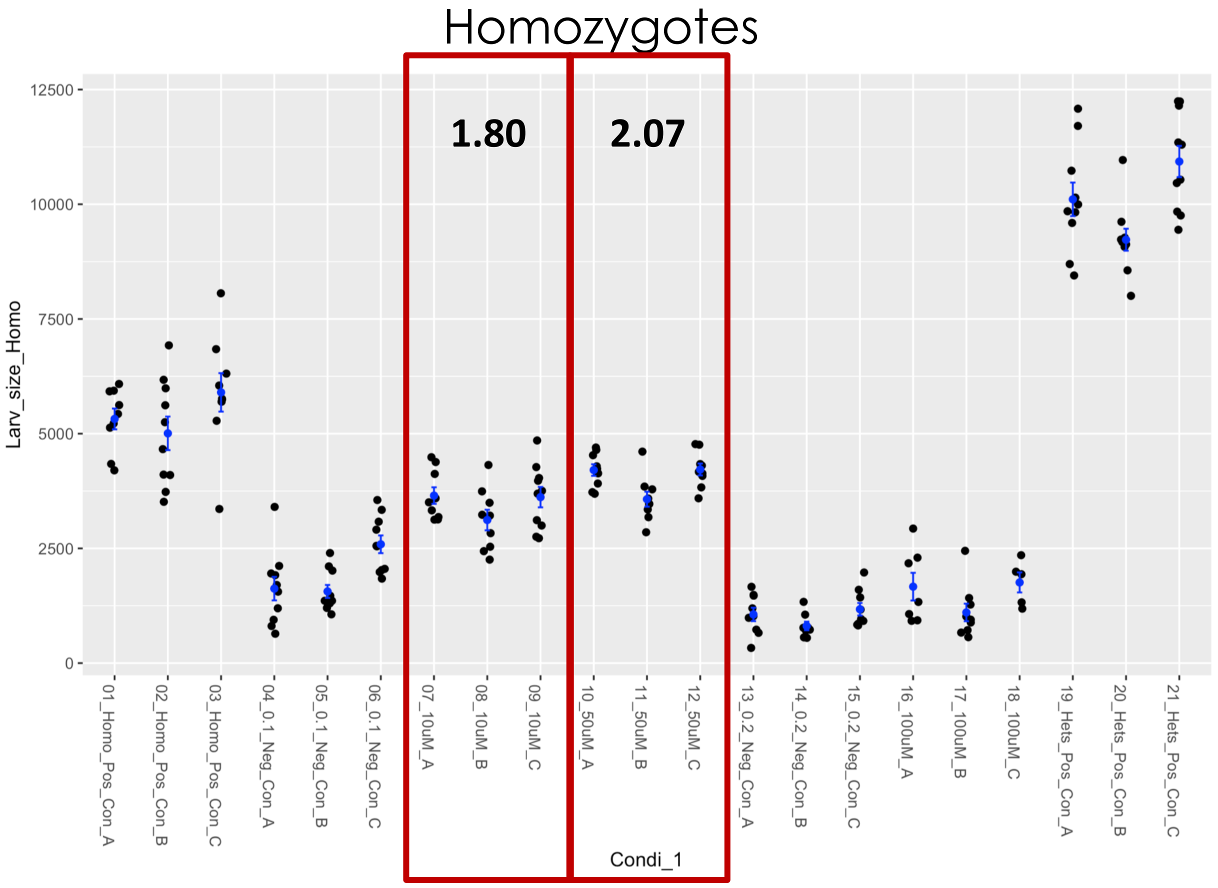

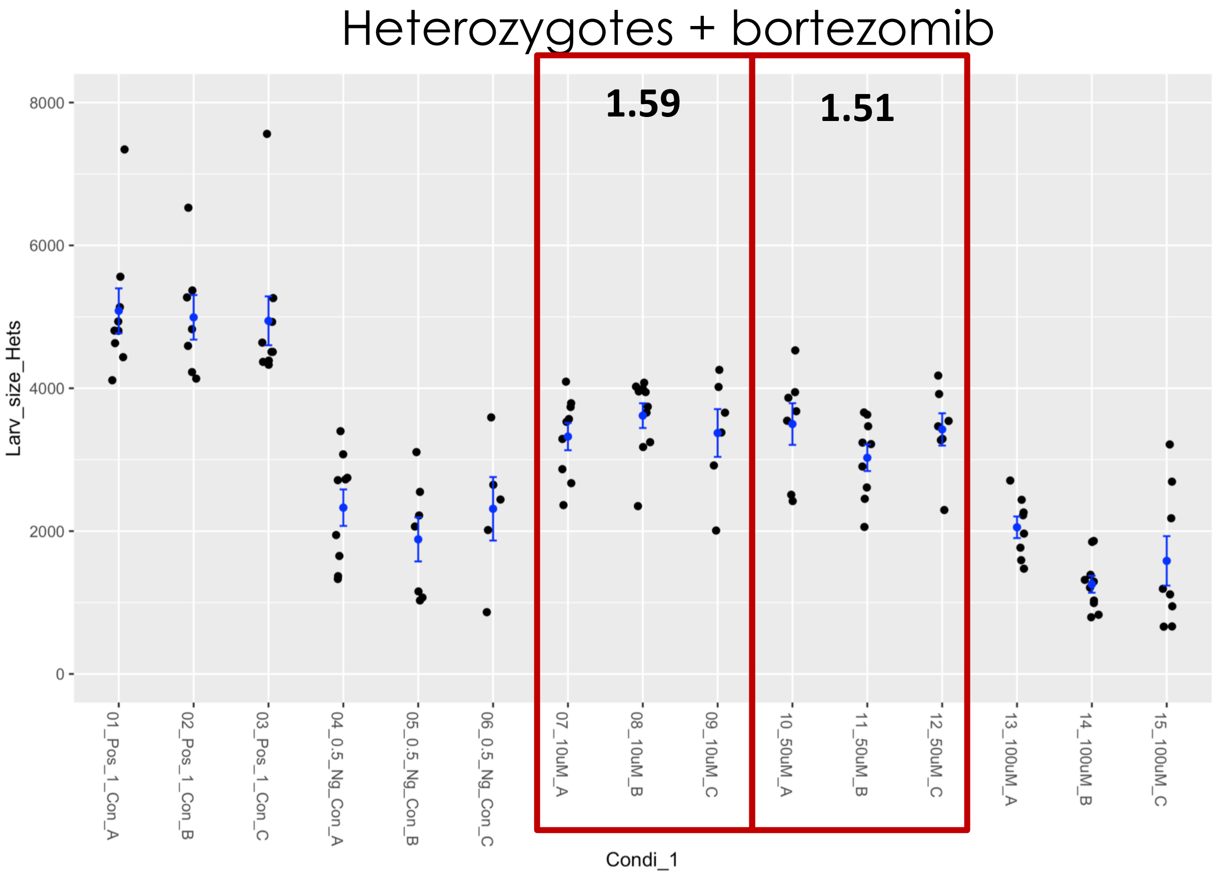

Fly WormMS cross validation – POMIFERIN

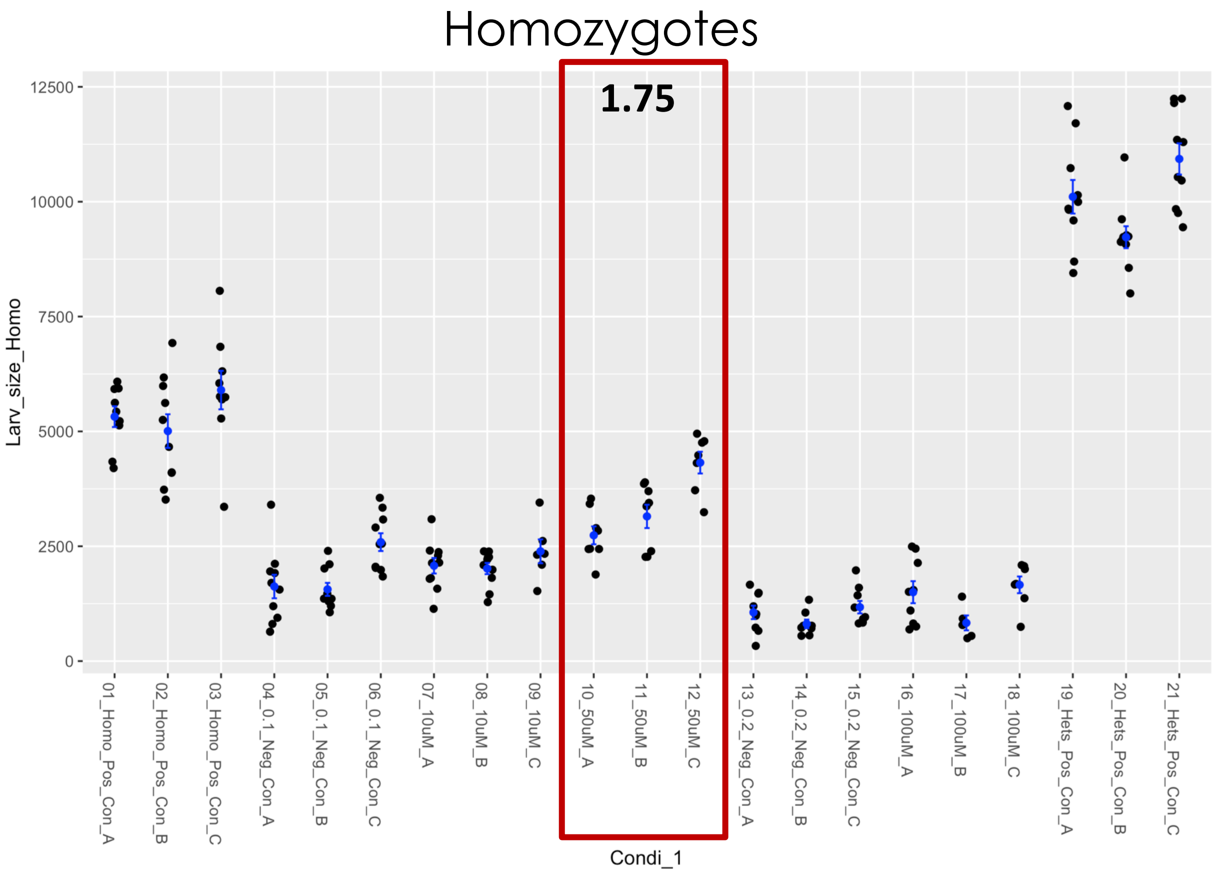

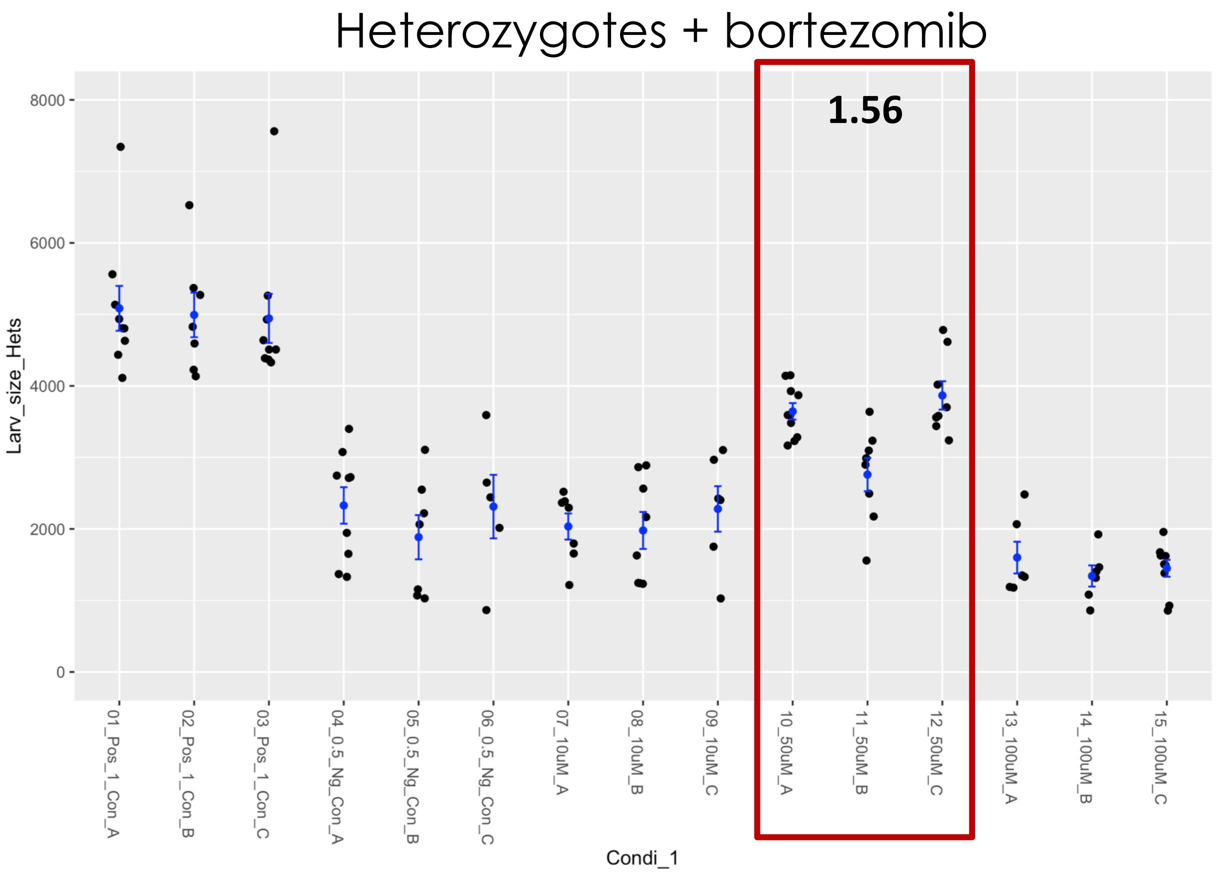

Disease Models & Mechanisms • Supplementary information

Fly WormMS cross validation – PURPUROGALLIN-4-CARBOXYLIC ACID

Homozygotes

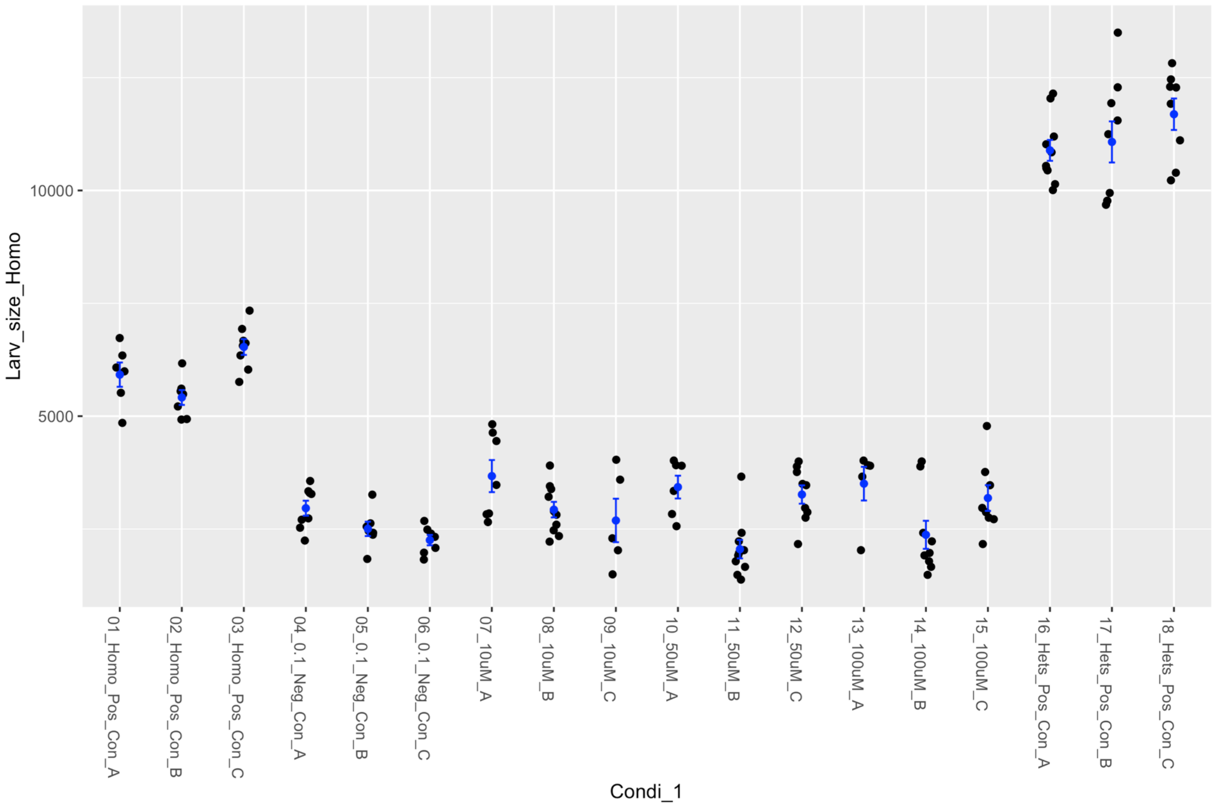

Heterozygotes + bortezomib

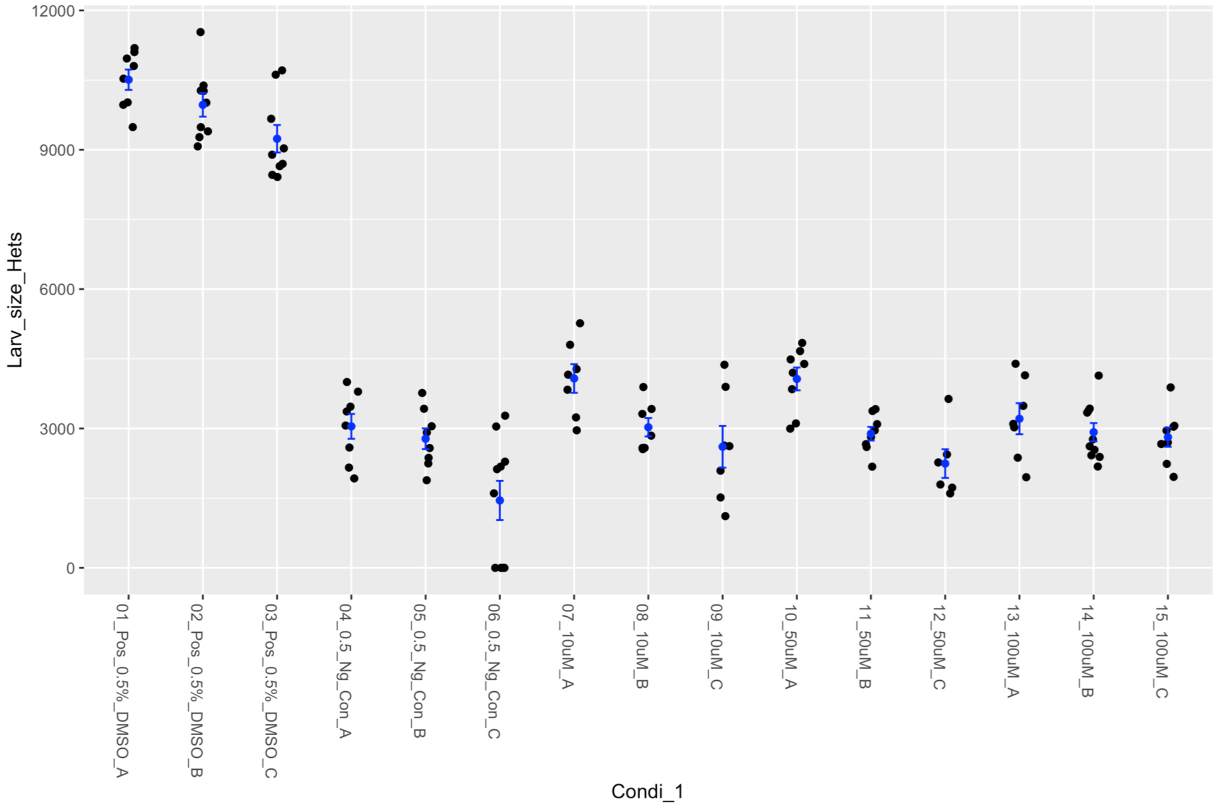

Disease Models & Mechanisms • Supplementary information

Fly WormMS cross validation – QUERCETIN

Homozygotes

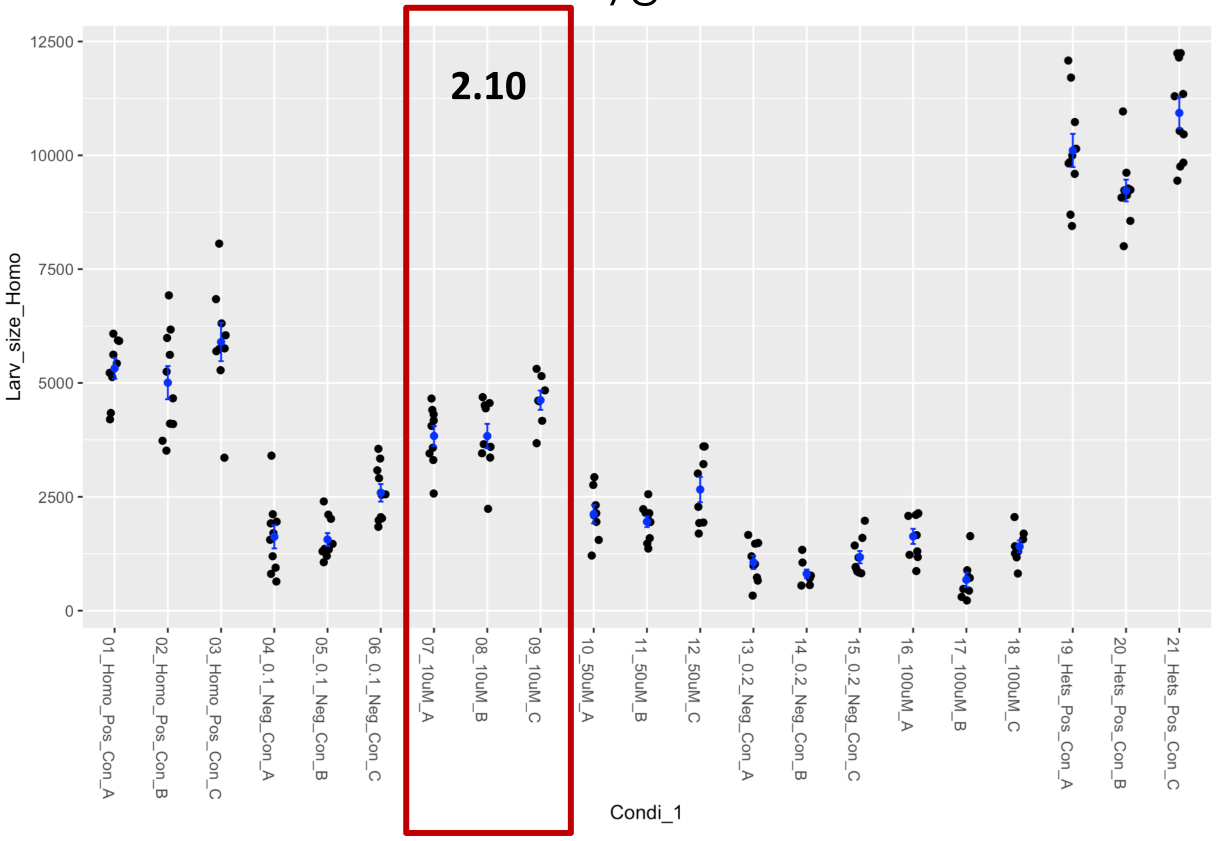

Heterozygotes + bortezomib

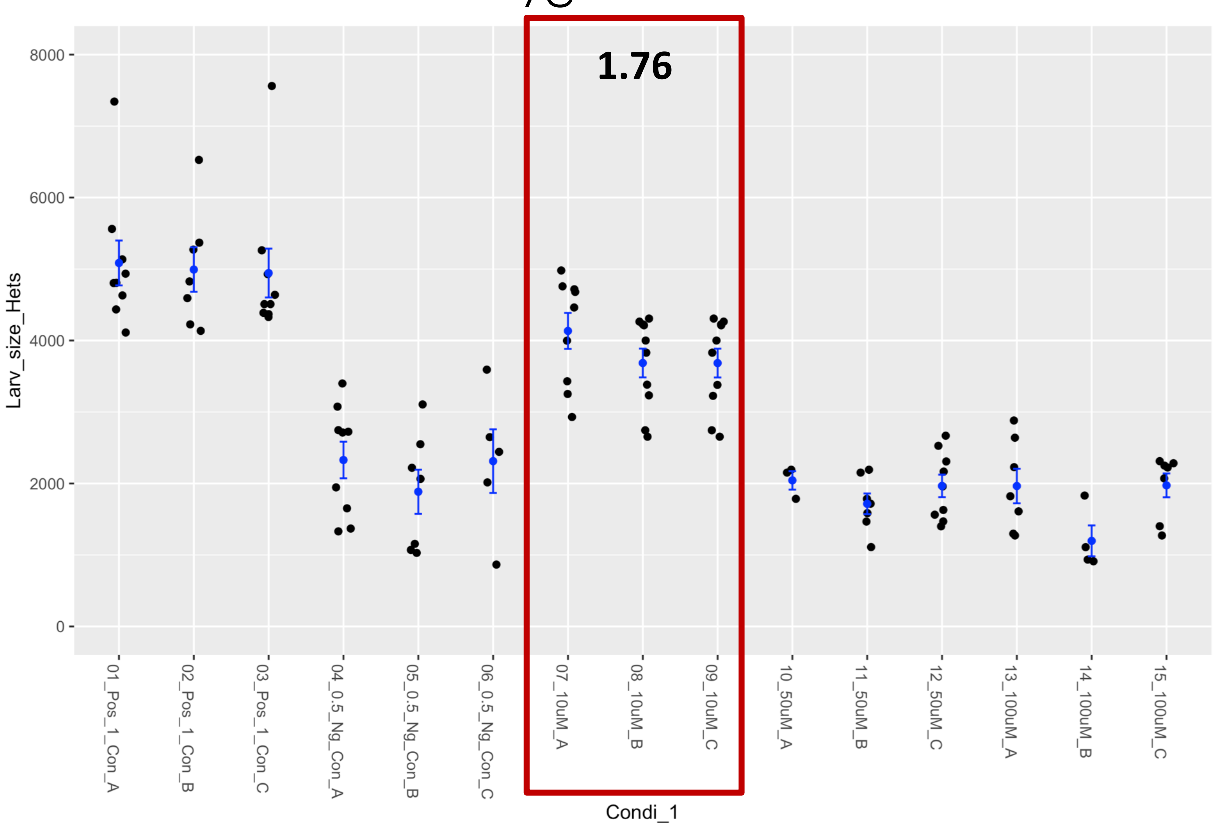

Disease Models & Mechanisms • Supplementary information

Disease Models & Mechanisms: doi:10.1242/dmm.040576: Supplementary information

Fly WormMS cross validation – THEAFLAVIN MONOGALLATE

Homozygotes

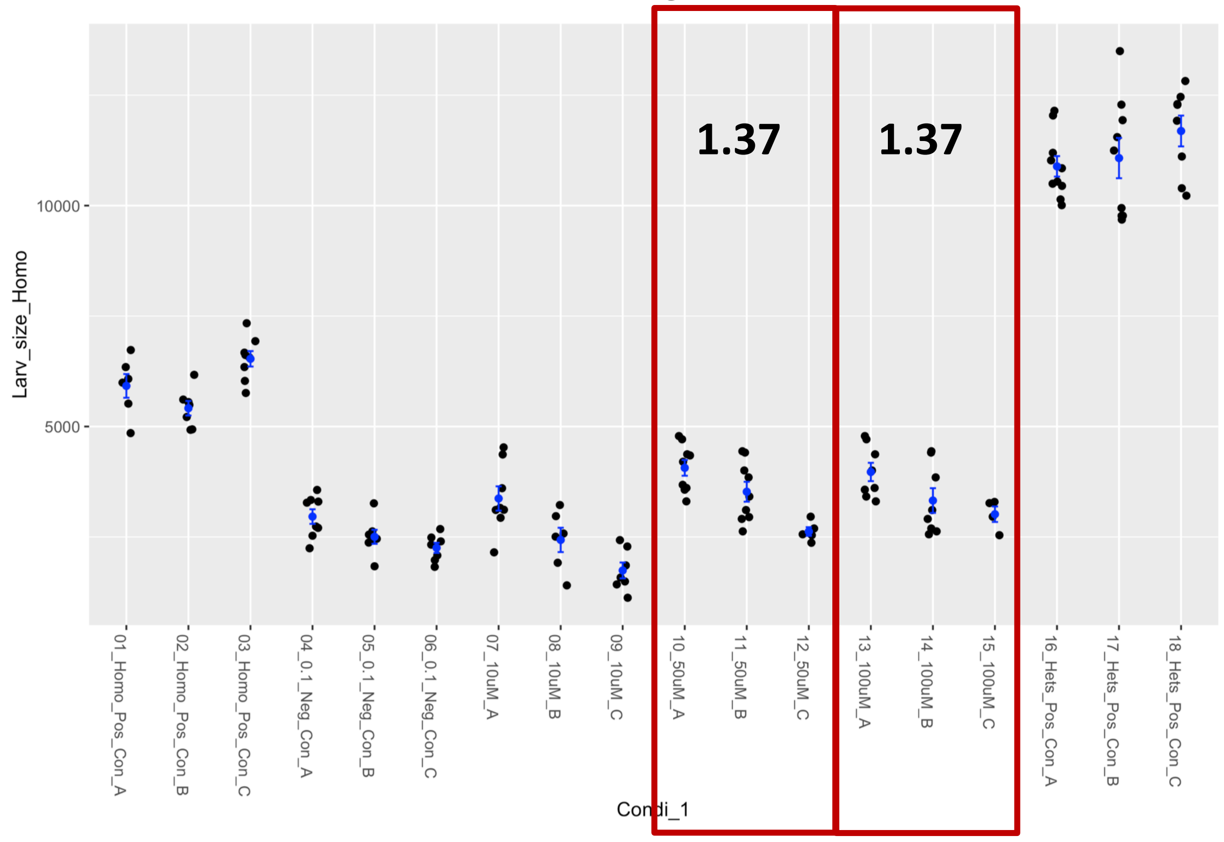

Heterozygotes + bortezomib

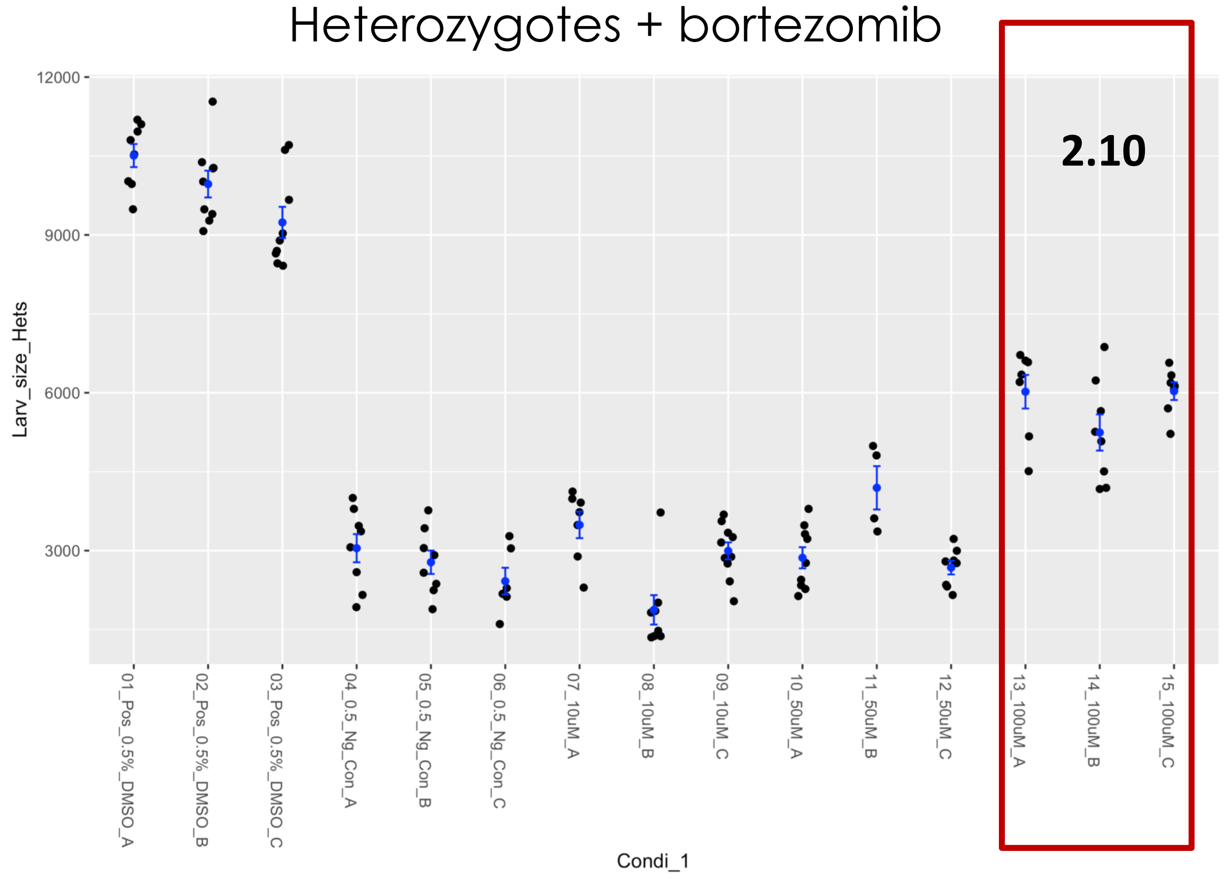

Fly WormMS cross validation – TRIAMCINOLONE

Homozygotes

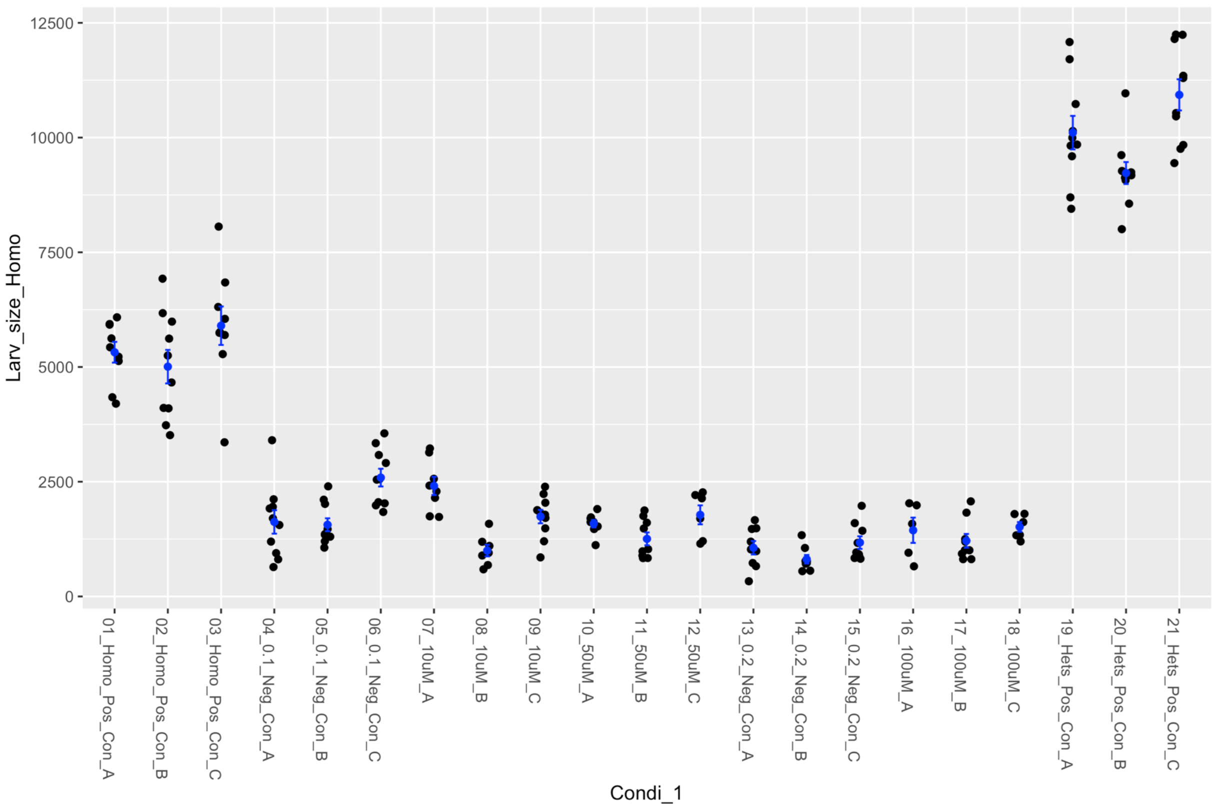

Heterozygotes + bortezomib

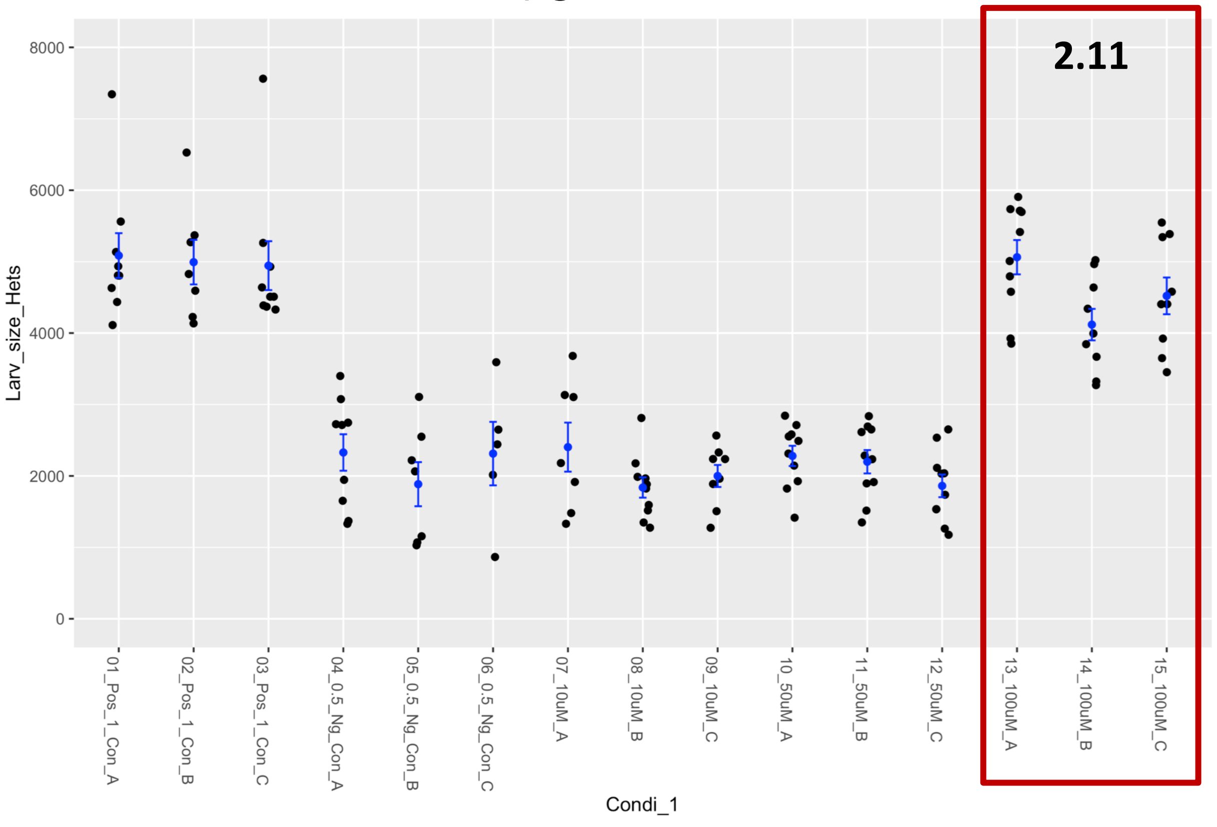

Disease Models & Mechanisms • Supplementary information

Fly Chembridge Retests

All compounds that scored as hits in the fly *Pngl*$^{+/-}$ Chembridge (drug discovery) screen were retested in two fly secondary assays : rescue of *Pngl*$^{-/-}$ homozygotes and rescue of bortezomib-treated *Pngl*$^{+/-}$ heterozygotes. Red boxes indicate compounds that successfully retested. The number indicates the size ratio of compound-treated fly larvae to DMSO-treated control homozygous fly larvae. For example, 2 means that a compound increased the size of mutant larvae by twofold.

Fly Chembridge retest – Supplier ID: 10343708

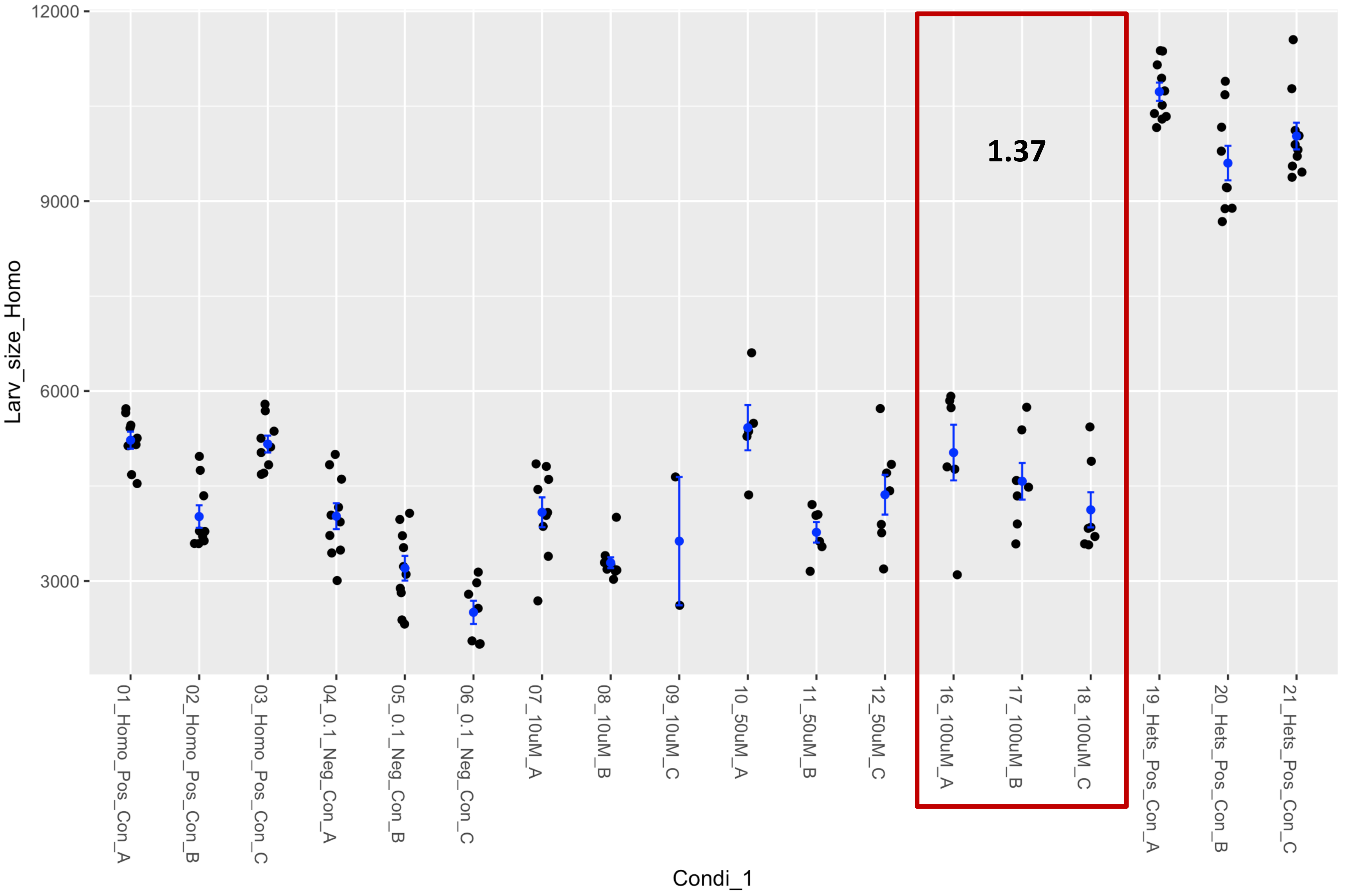

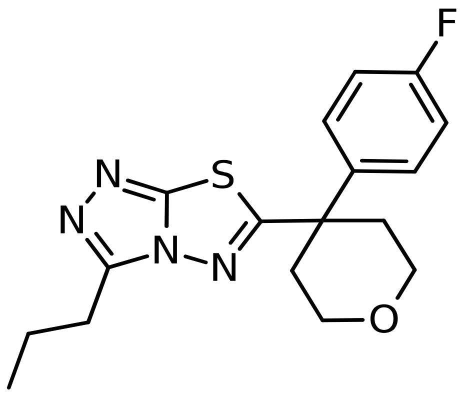

Disease Models & Mechanisms • Supplementary information

Fly Chembridge retest – Supplier ID: 11646952

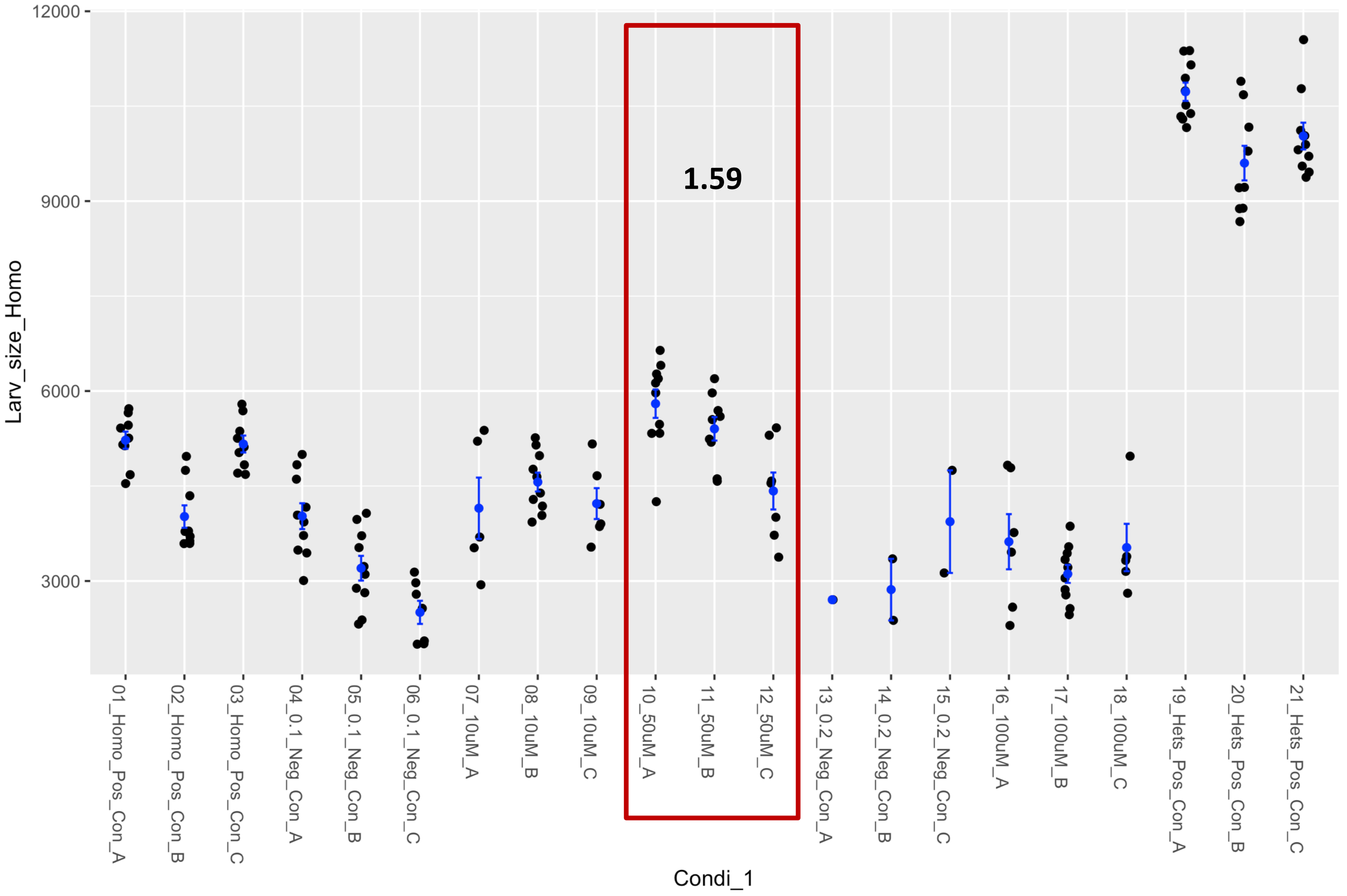

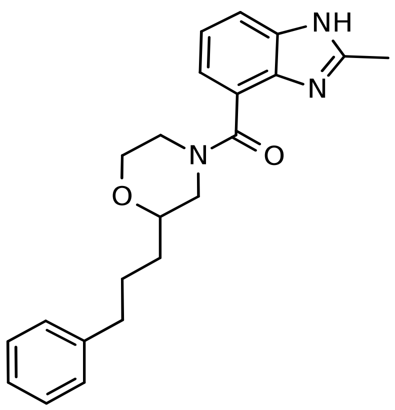

Disease Models & Mechanisms • Supplementary information

Fly Chembridge retest – Supplier ID: 22934709

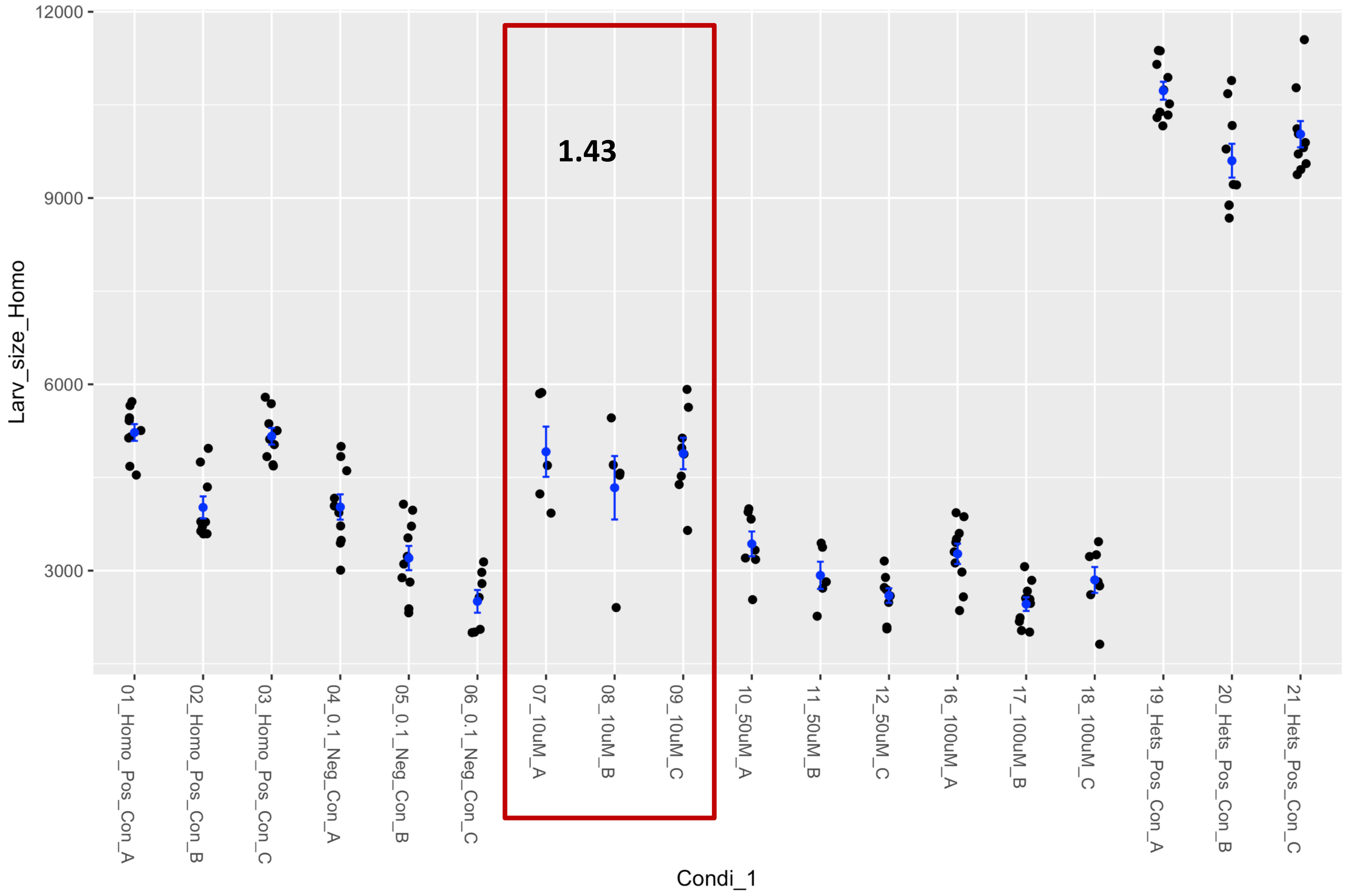

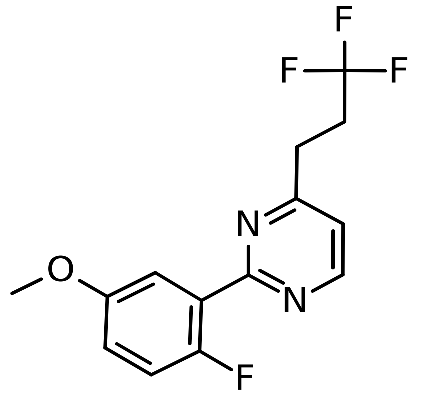

Fly Chembridge retest – Supplier ID: 26889292

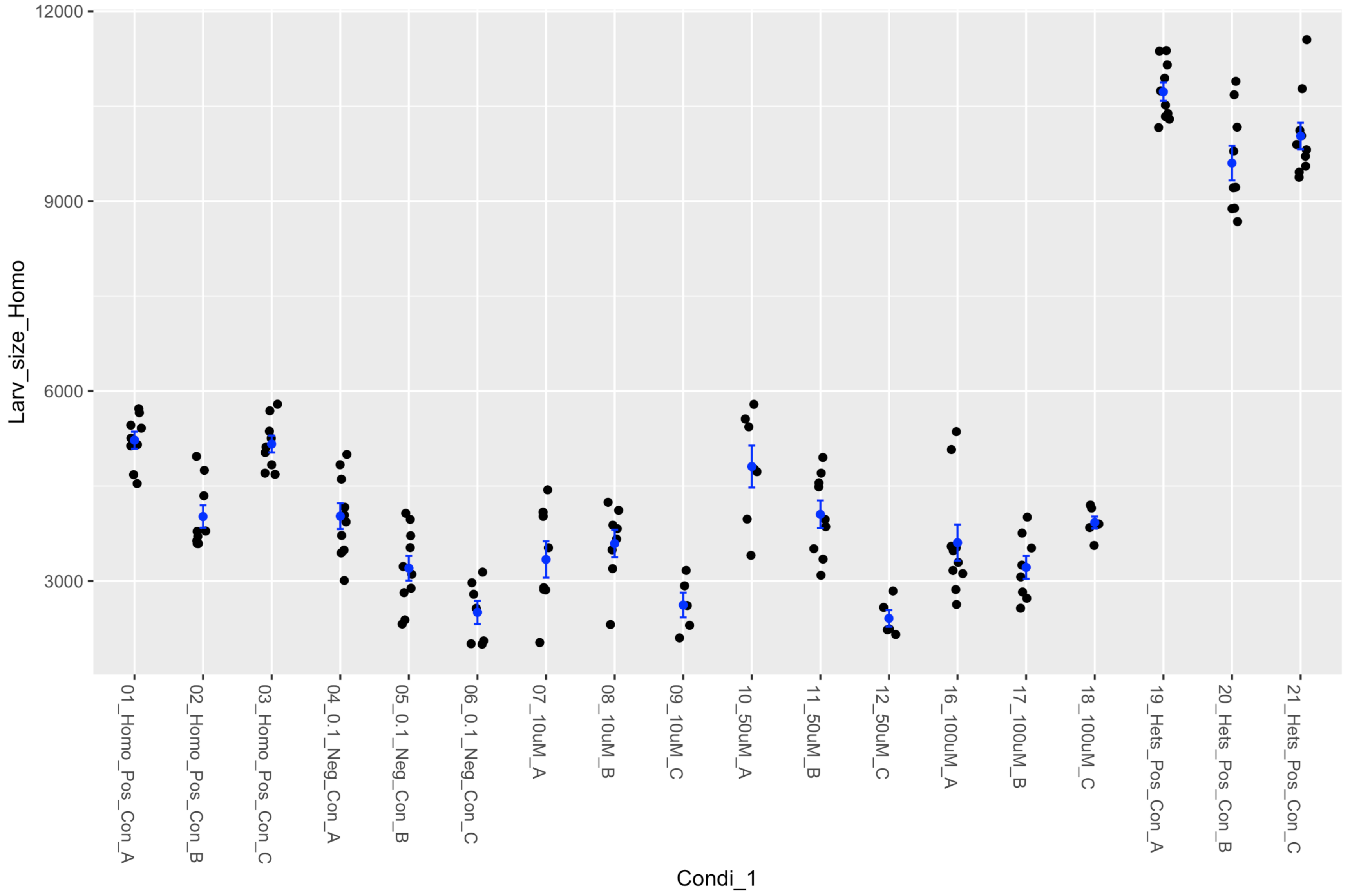

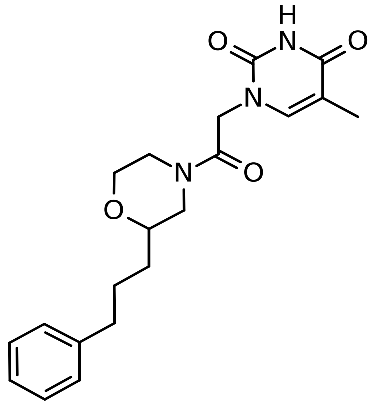

Disease Models & Mechanisms • Supplementary information

Fly Chembridge retest – Supplier ID: 35527962

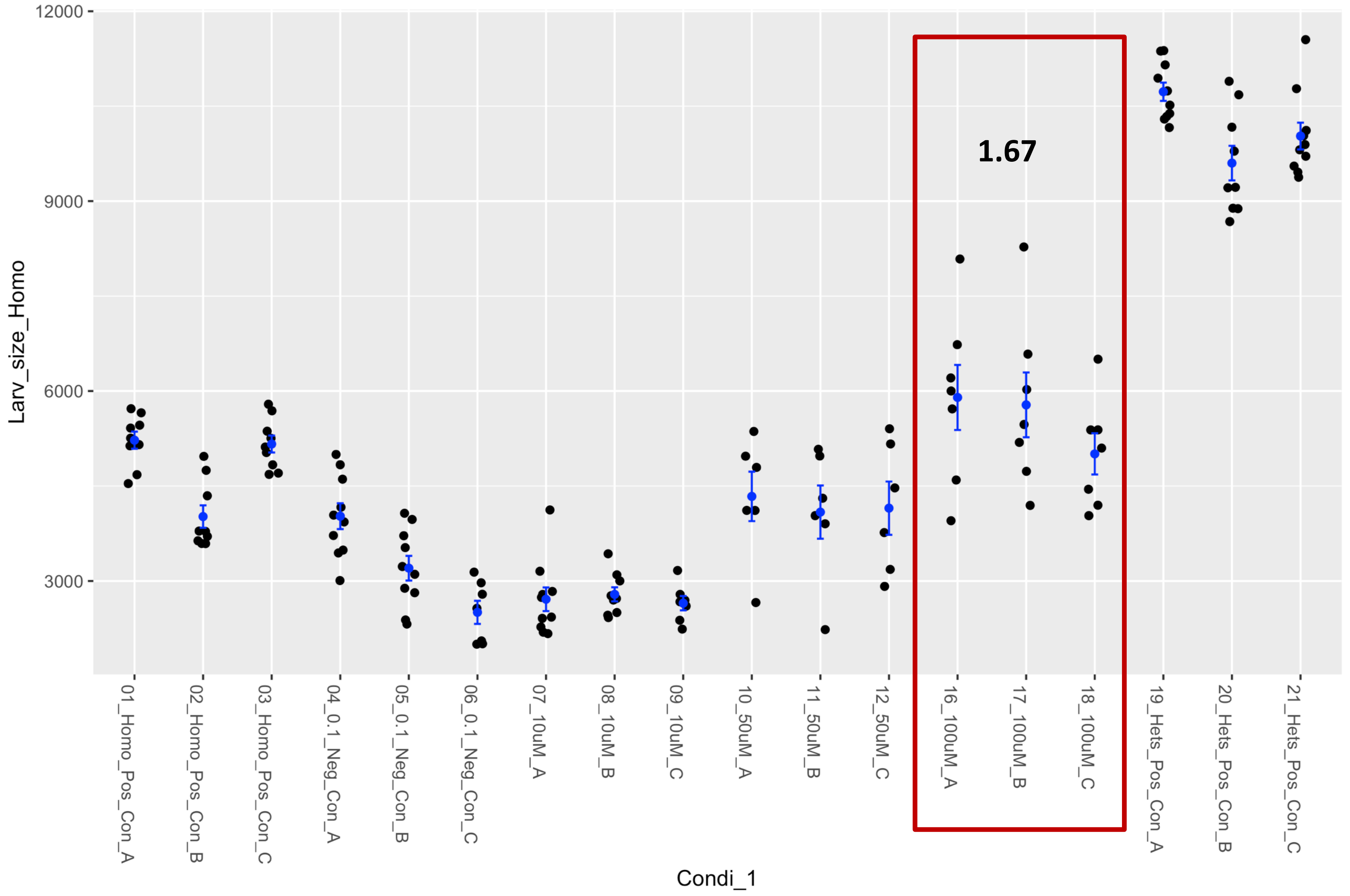

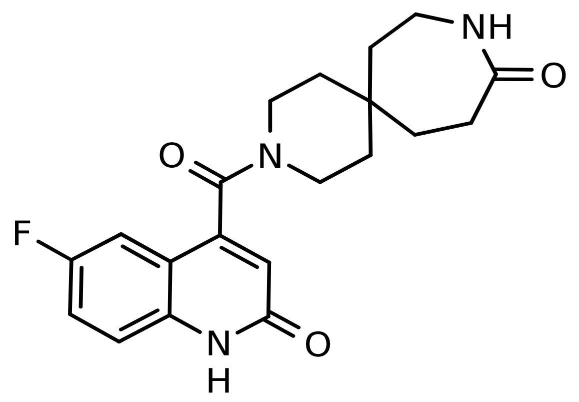

Disease Models & Mechanisms • Supplementary information

Disease Models & Mechanisms: doi:10.1242/dmm.040576: Supplementary information

Fly Chembridge retest – Supplier ID: 40966617

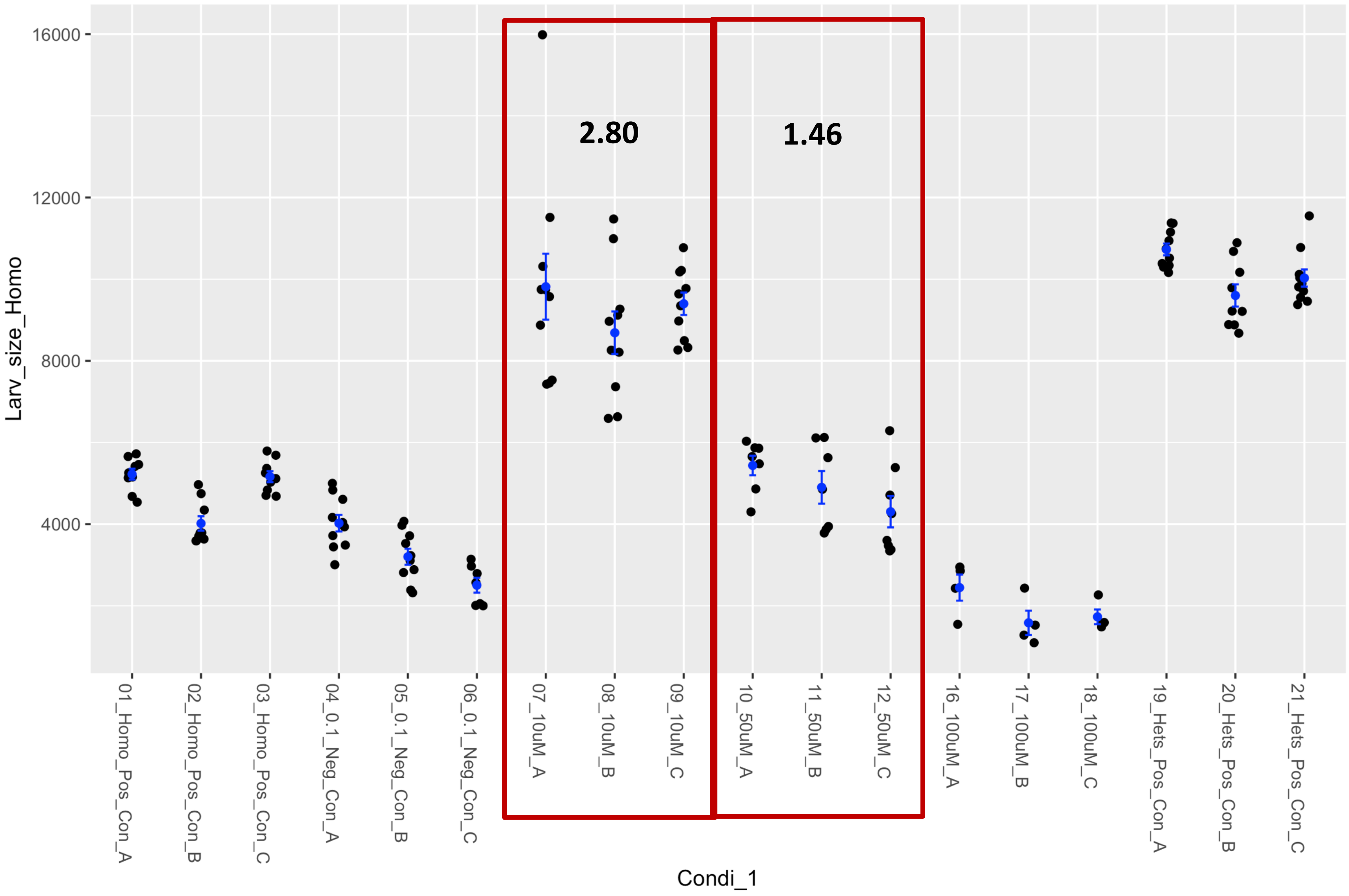

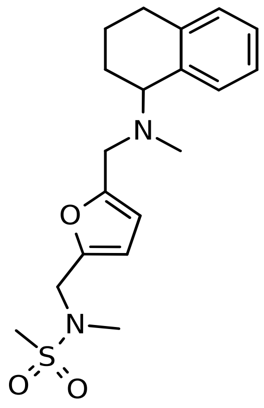

Disease Models & Mechanisms • Supplementary information

Disease Models & Mechanisms: doi:10.1242/dmm.040576: Supplementary information

Fly Chembridge retest – Supplier ID: 58095314

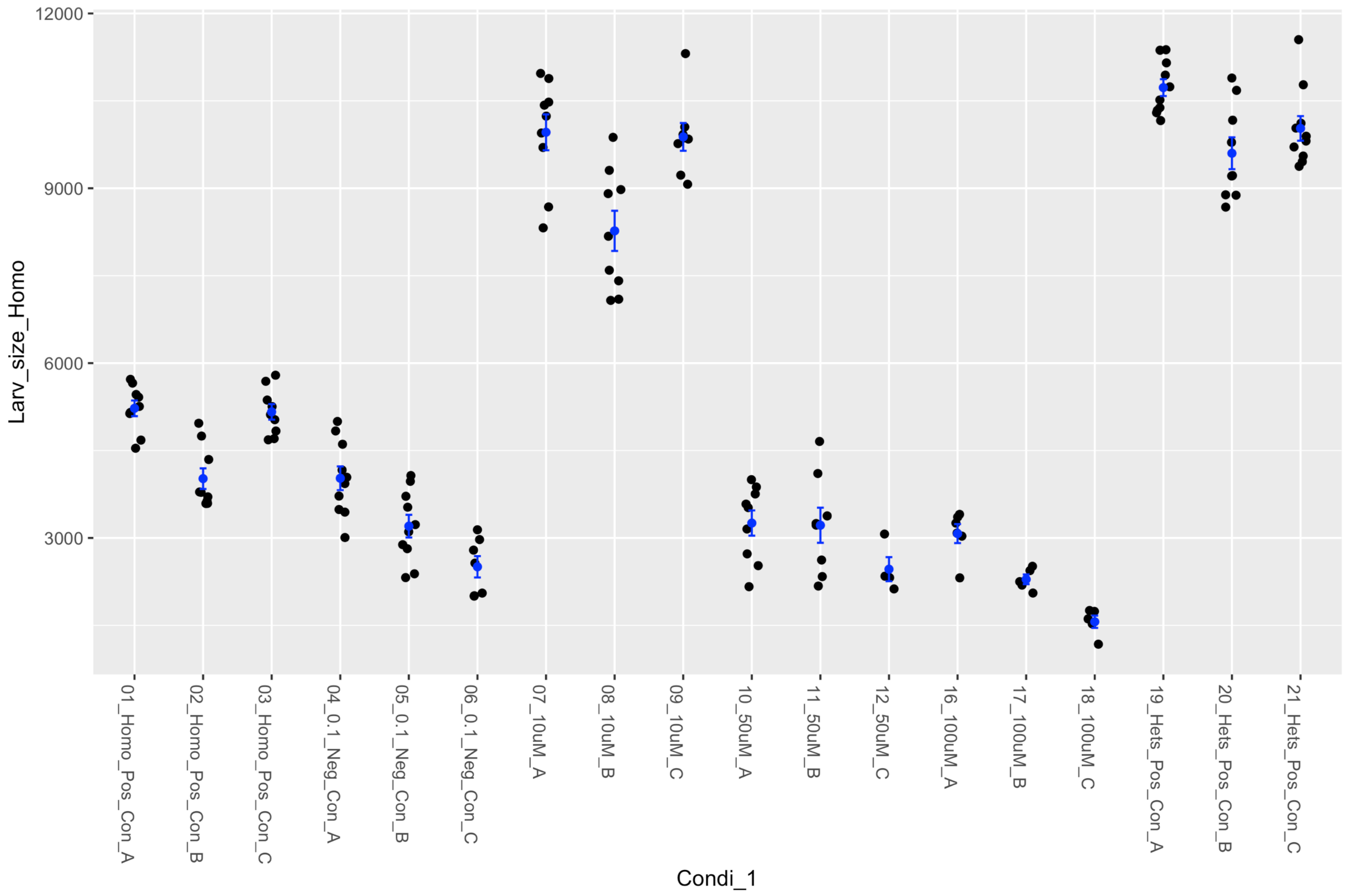

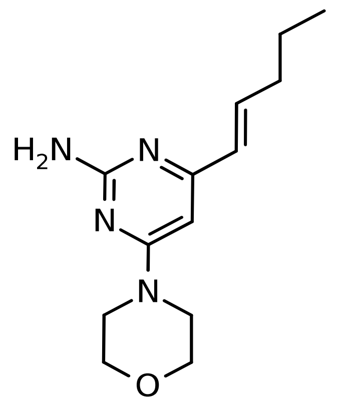

Disease Models & Mechanisms • Supplementary information

Fly Chembridge retest – Supplier ID: 89890646

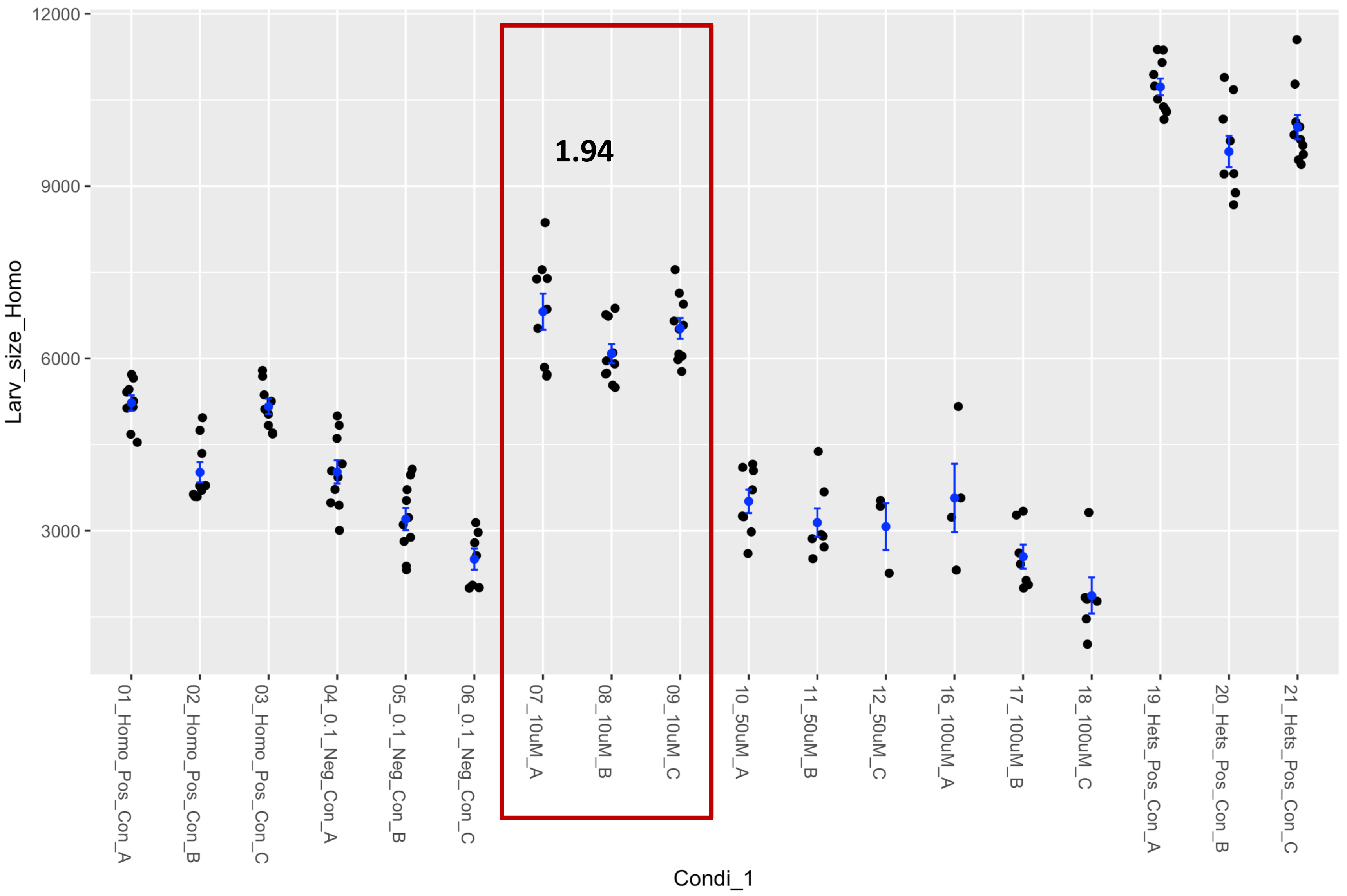

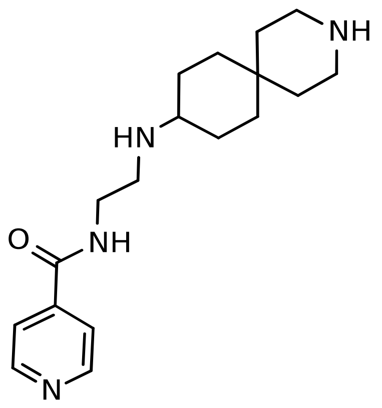

Disease Models & Mechanisms • Supplementary information

Disease Models & Mechanisms: doi:10.1242/dmm.040576: Supplementary information

Fly Chembridge retest – Supplier ID: 9206326

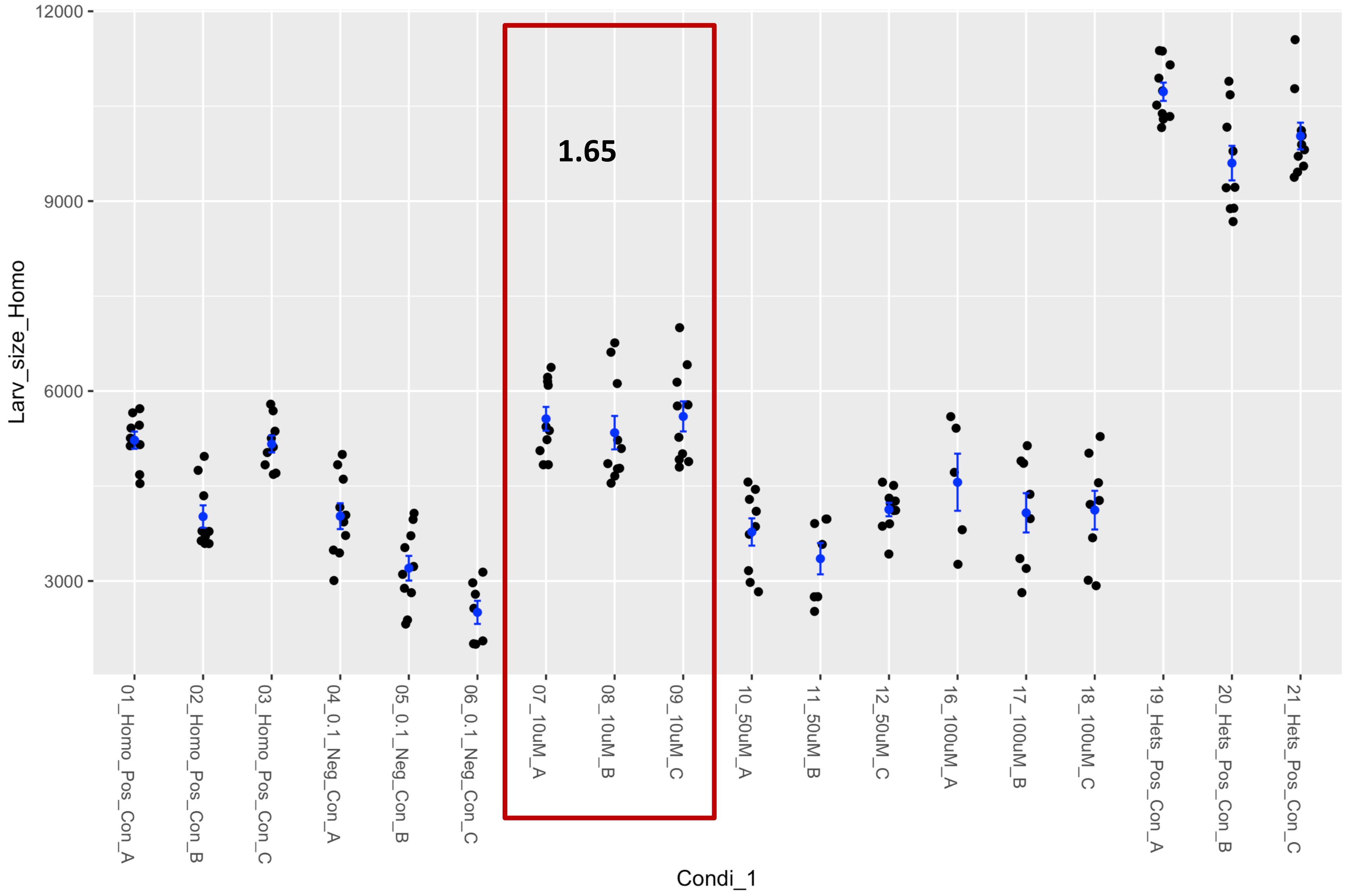

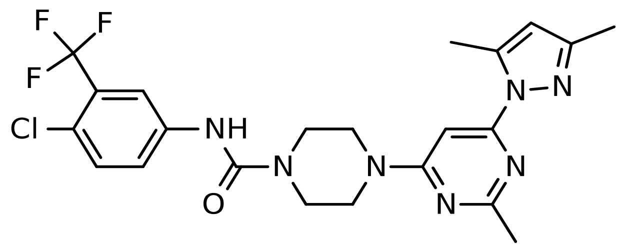

Disease Models & Mechanisms • Supplementary information

Fly Chembridge retest – Supplier ID: 9233974

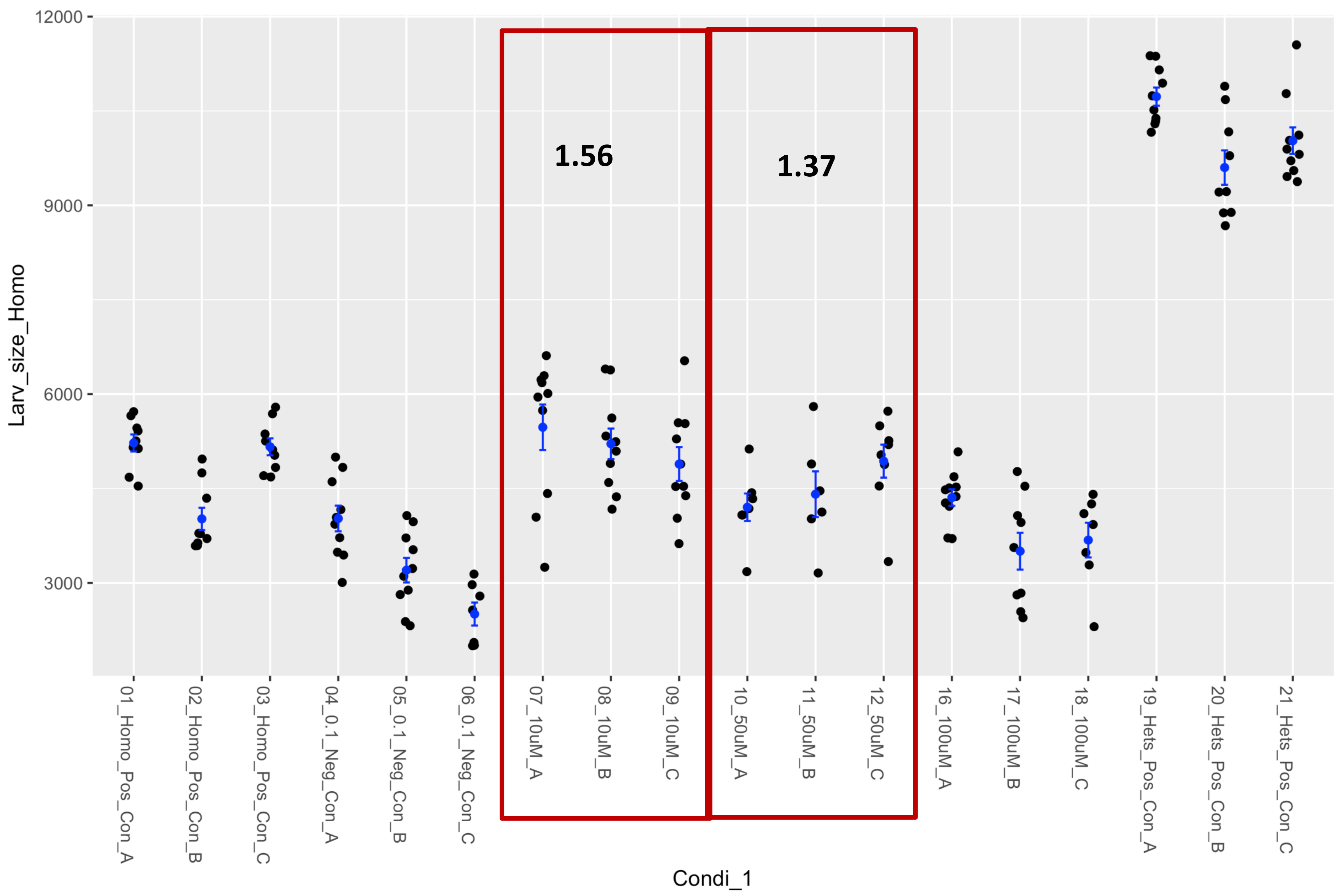

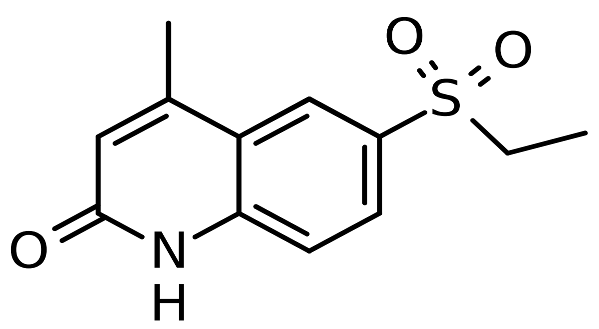

Disease Models & Mechanisms • Supplementary information

Fly Chembridge retest – Supplier ID: 9251119

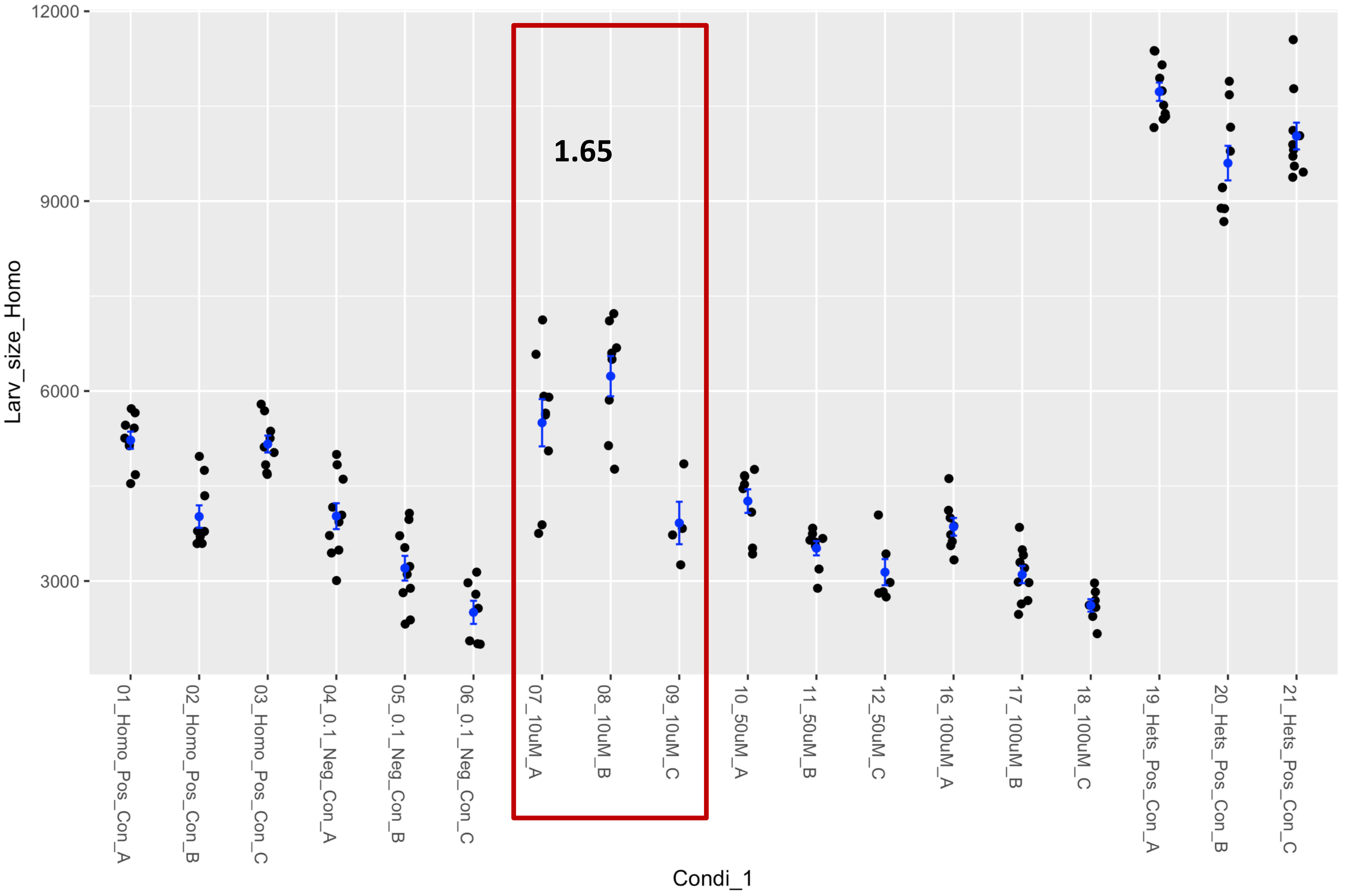

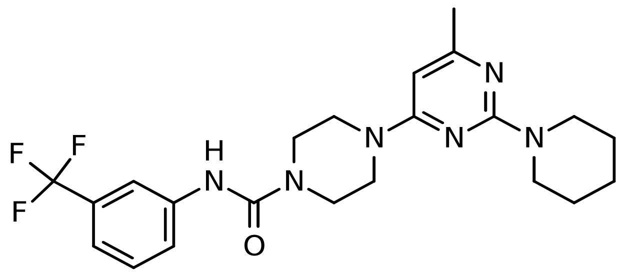

Disease Models & Mechanisms • Supplementary information

Fly Chembridge retest – Supplier ID: 9251633

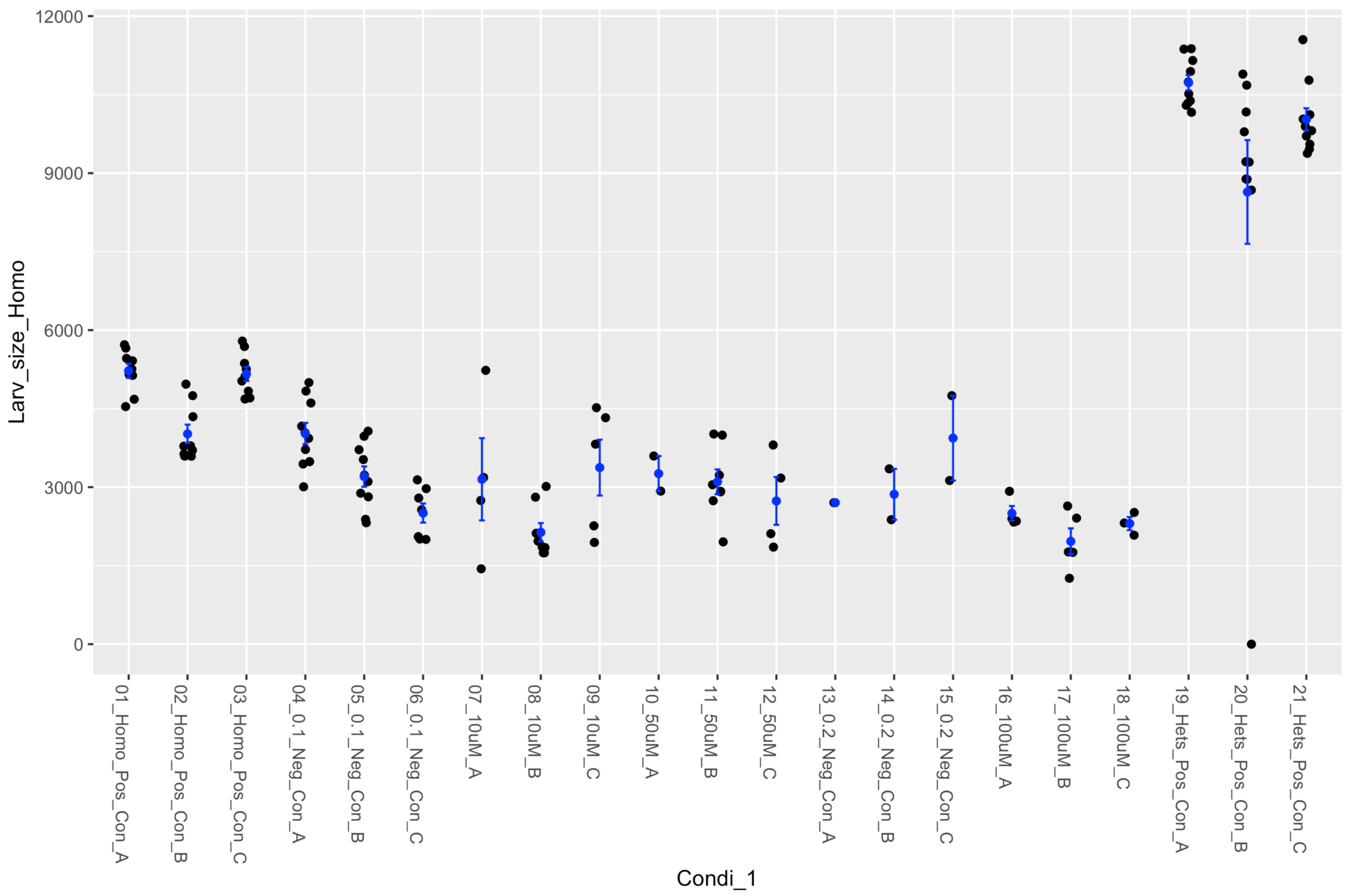

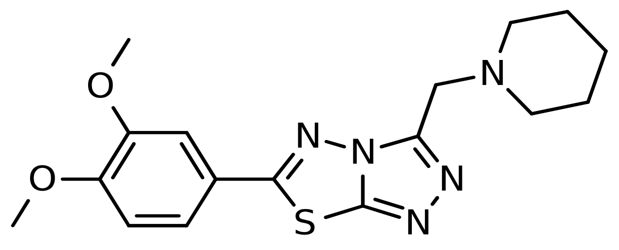

Disease Models & Mechanisms: doi:10.1242/dmm.040576: Supplementary information

Fly Chembridge retest – Supplier ID: 9262220

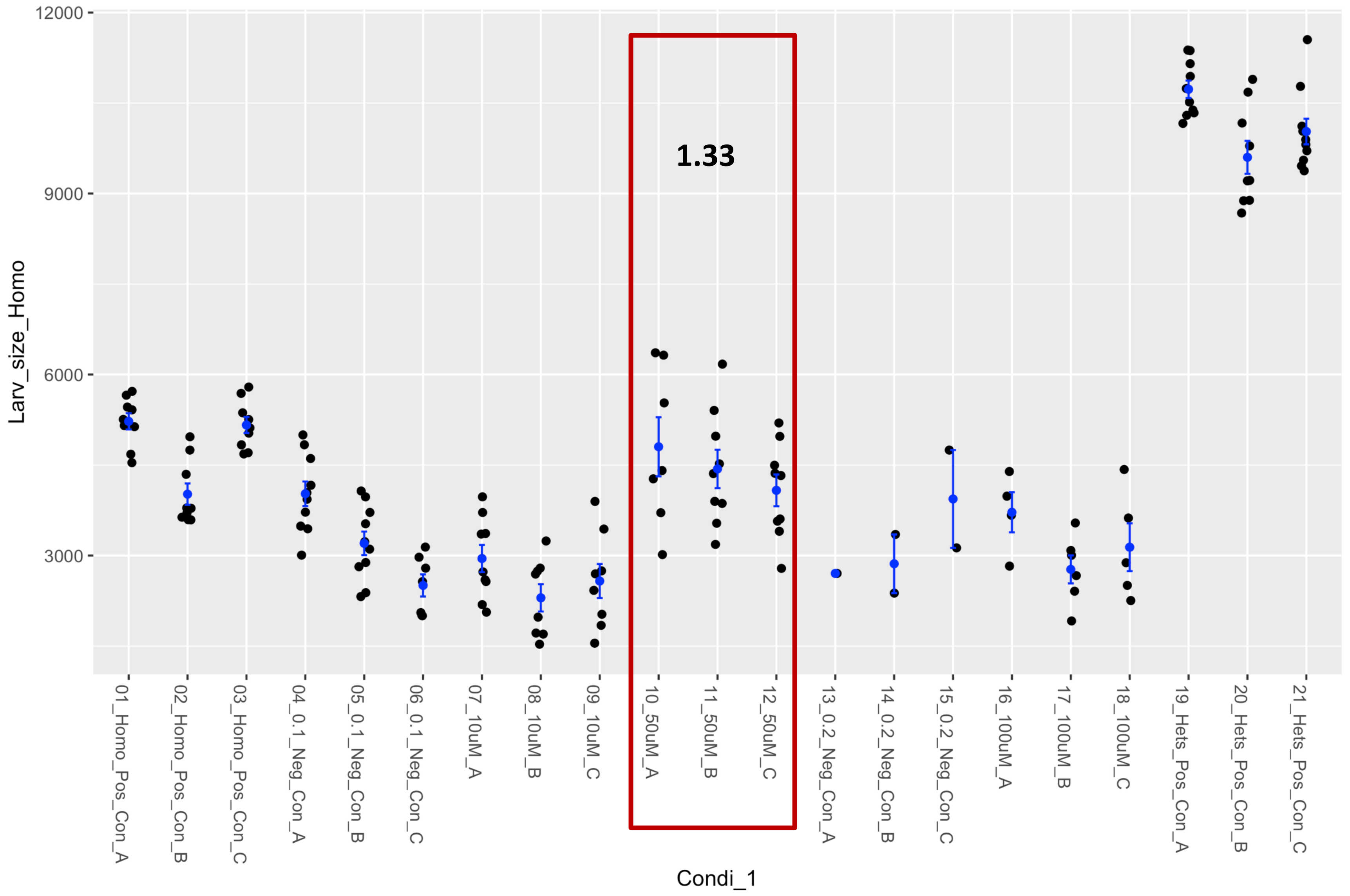

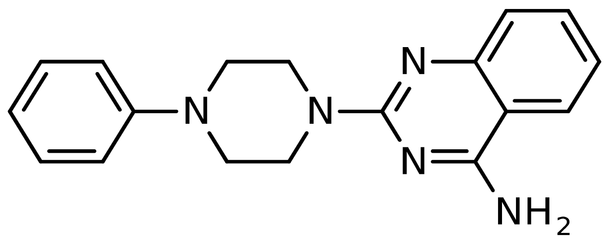

Disease Models & Mechanisms • Supplementary information

Fly Chembridge retest – Supplier ID: 9262955

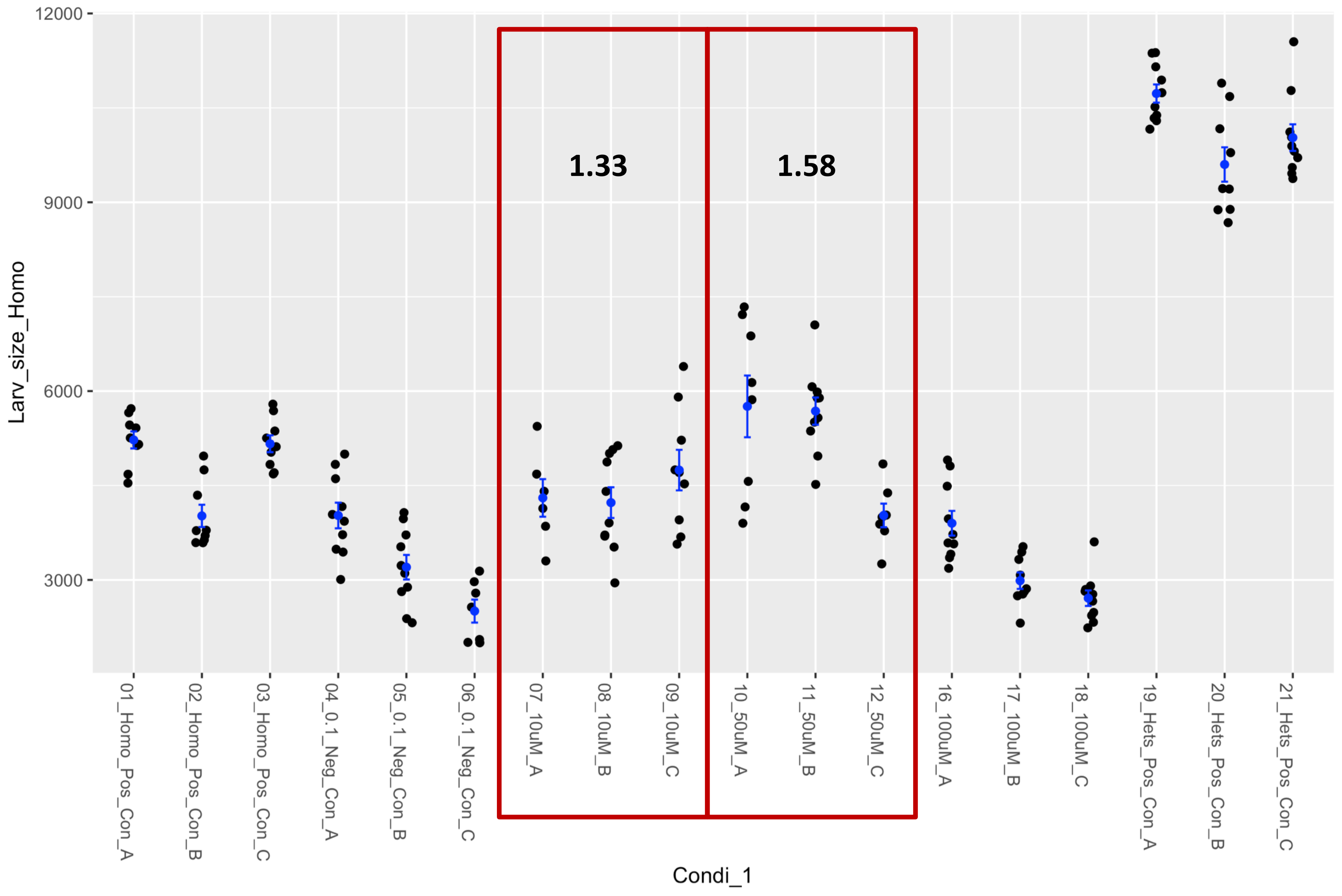

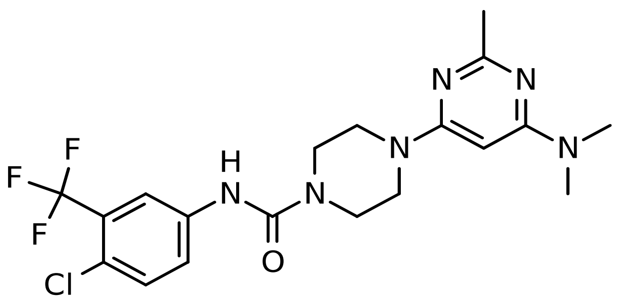

Disease Models & Mechanisms • Supplementary information

Fly Chembridge retest – Supplier ID: 9290684

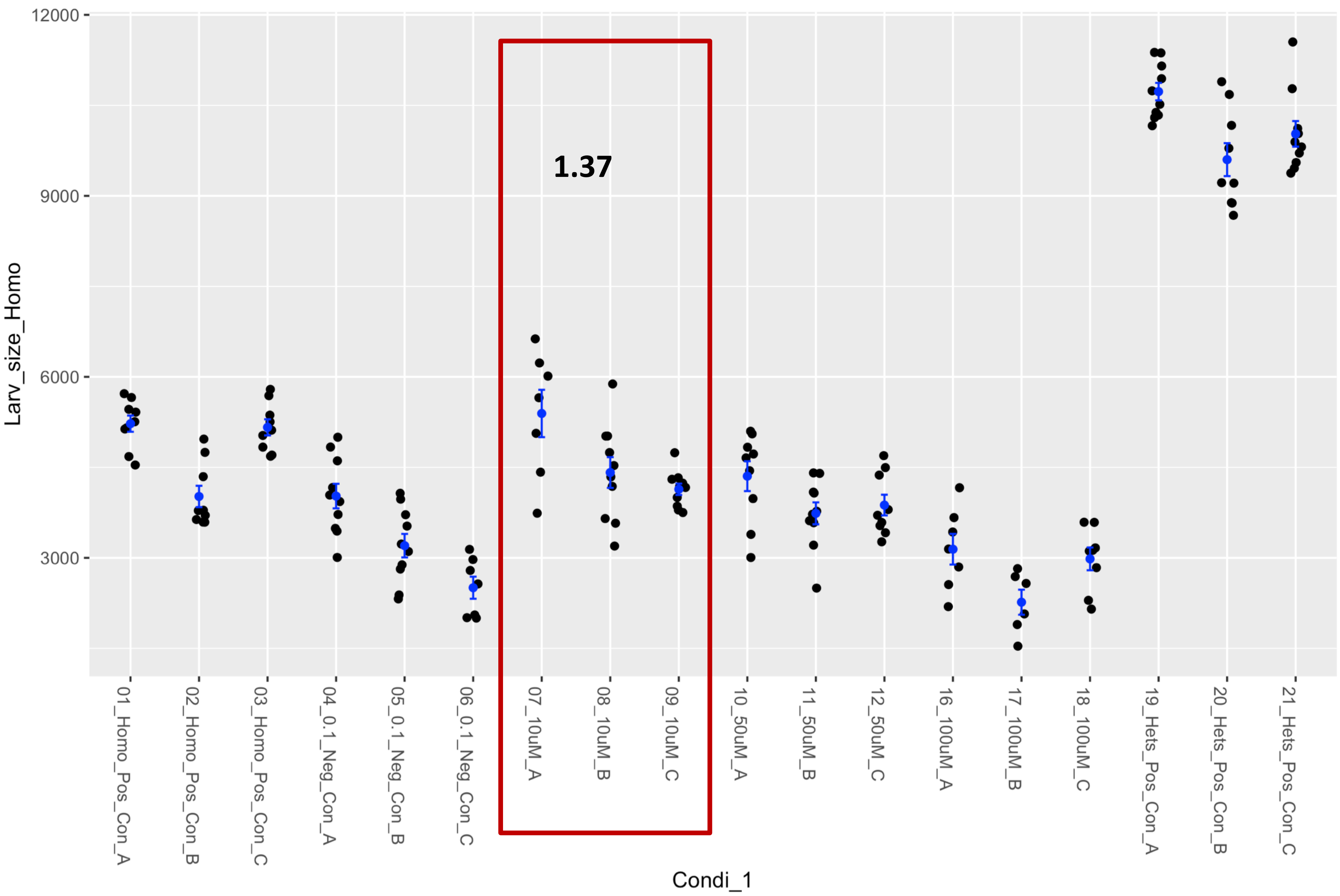

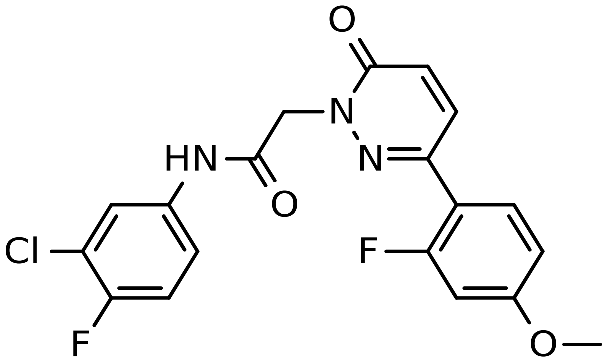

Disease Models & Mechanisms • Supplementary information

Fly Chembridge retest – Supplier ID: 10343708

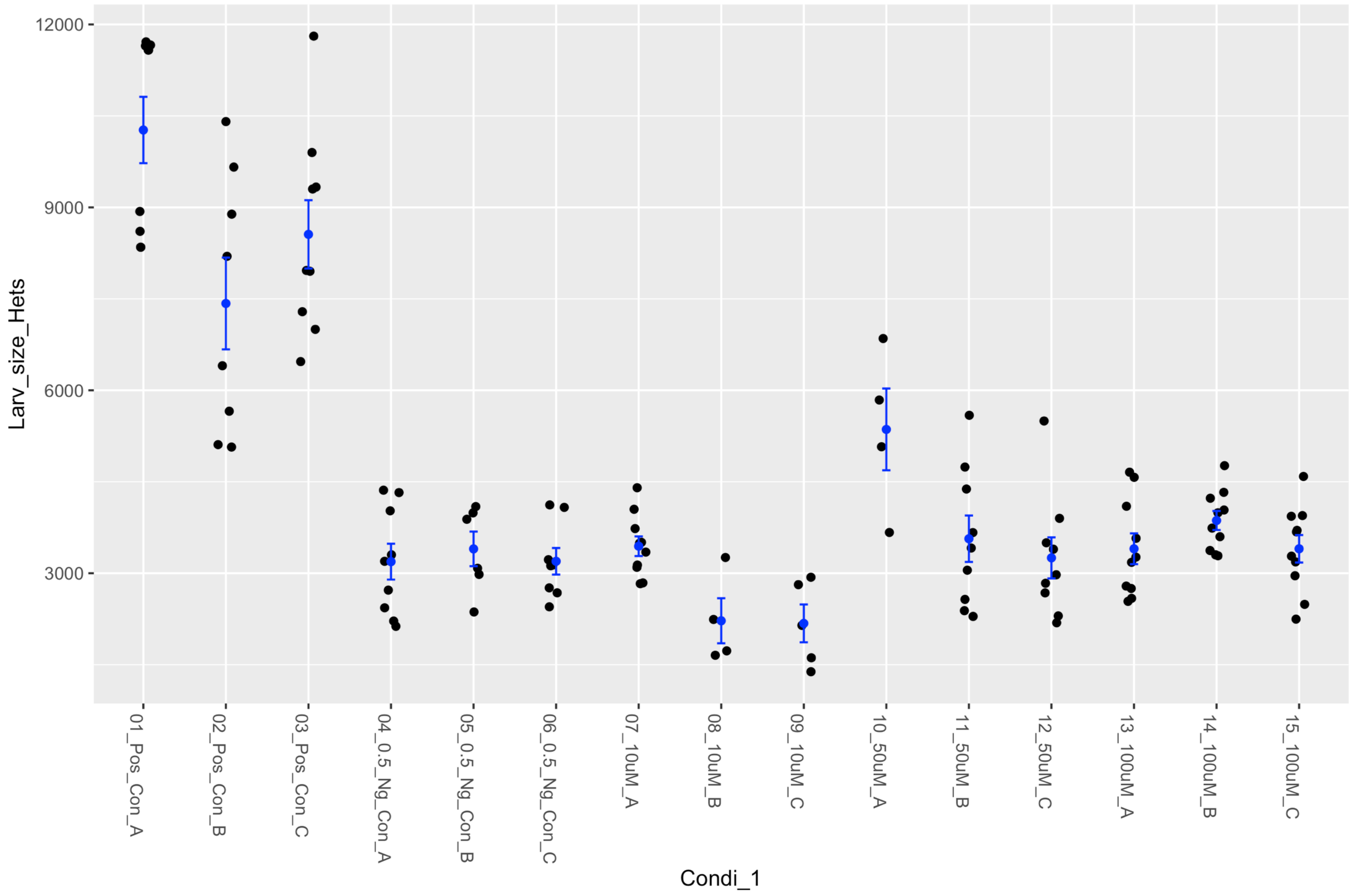

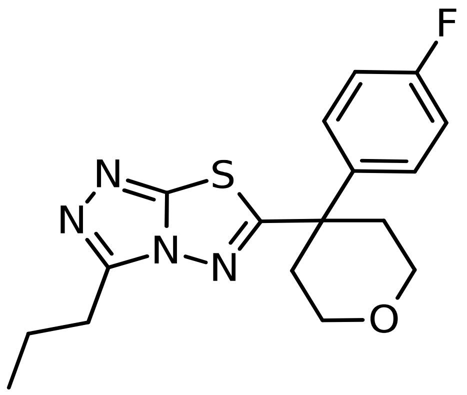

Disease Models & Mechanisms • Supplementary information

Disease Models & Mechanisms: doi:10.1242/dmm.040576: Supplementary information

Fly Chembridge retest – Supplier ID: 11646952

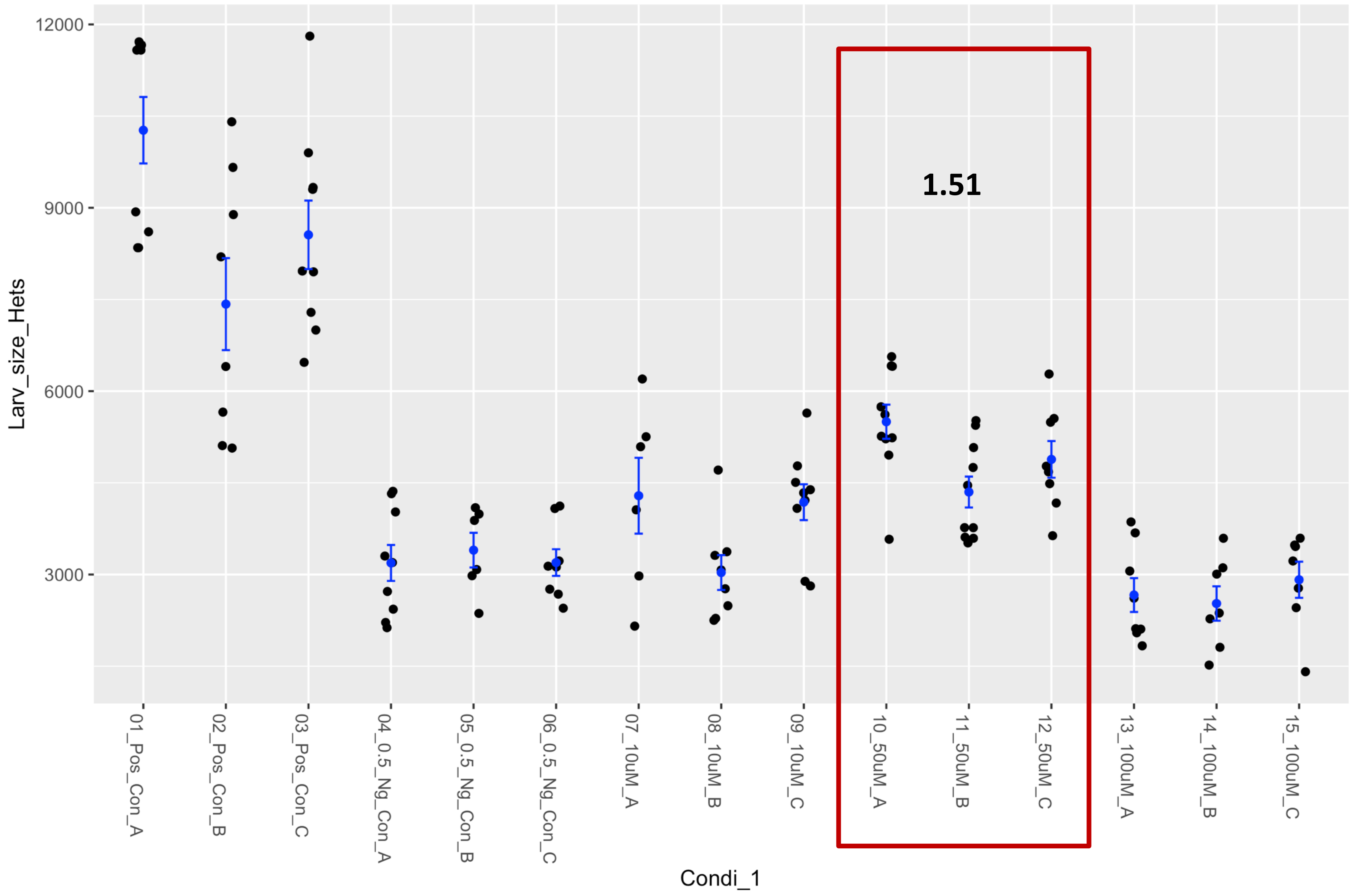

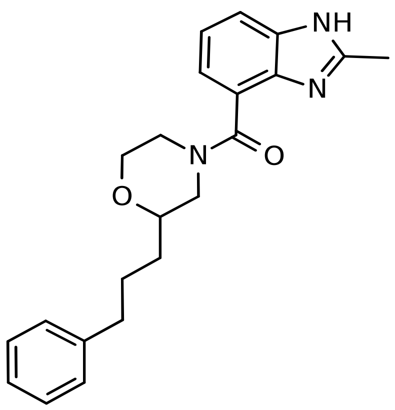

Disease Models & Mechanisms • Supplementary information

Fly Chembridge retest – Supplier ID: 22934709

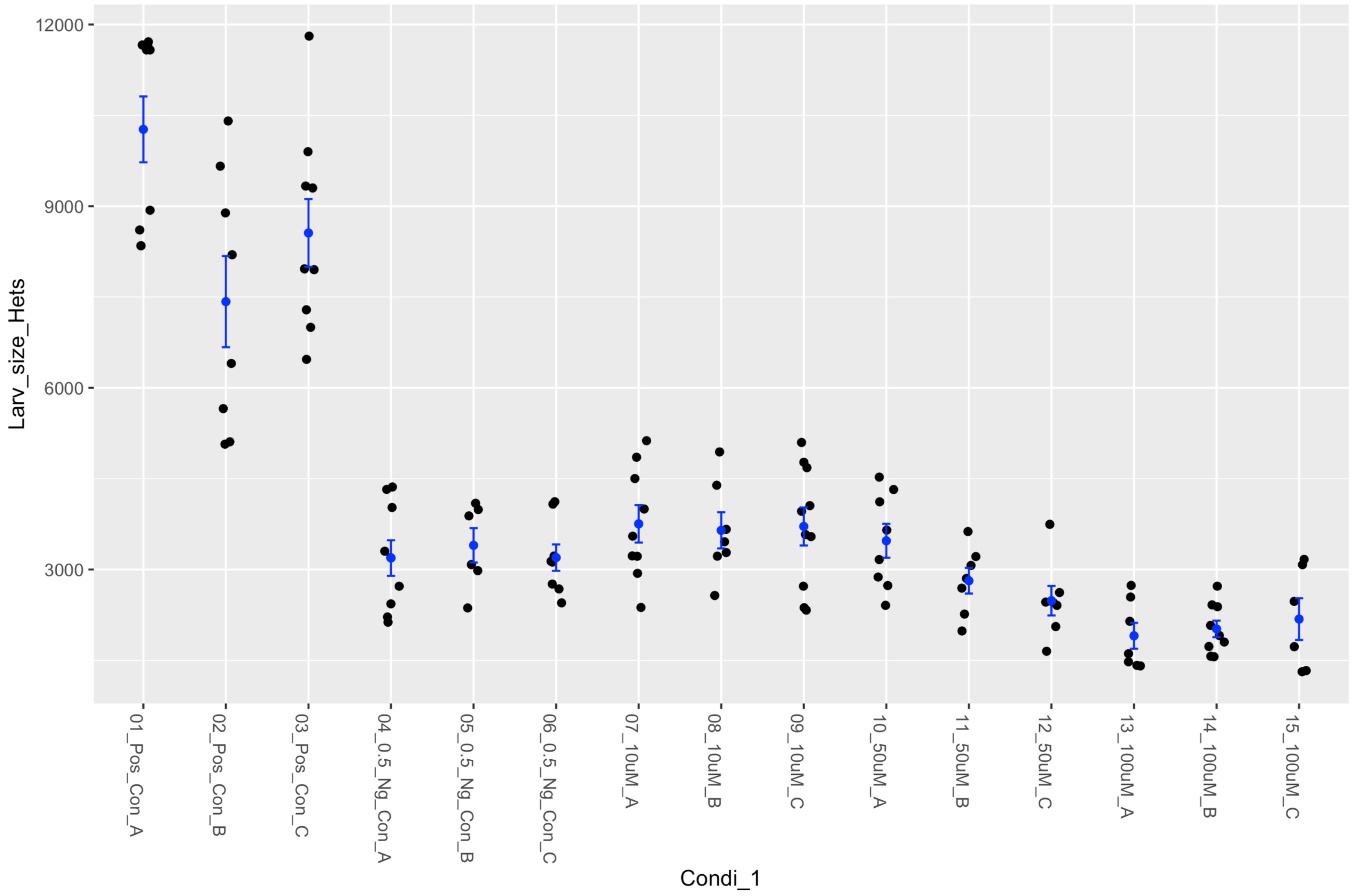

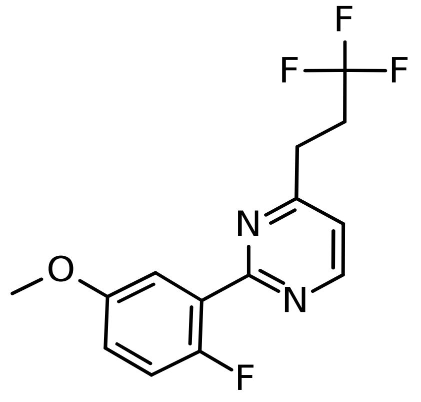

Disease Models & Mechanisms • Supplementary information

Fly Chembridge retest – Supplier ID: 26889292

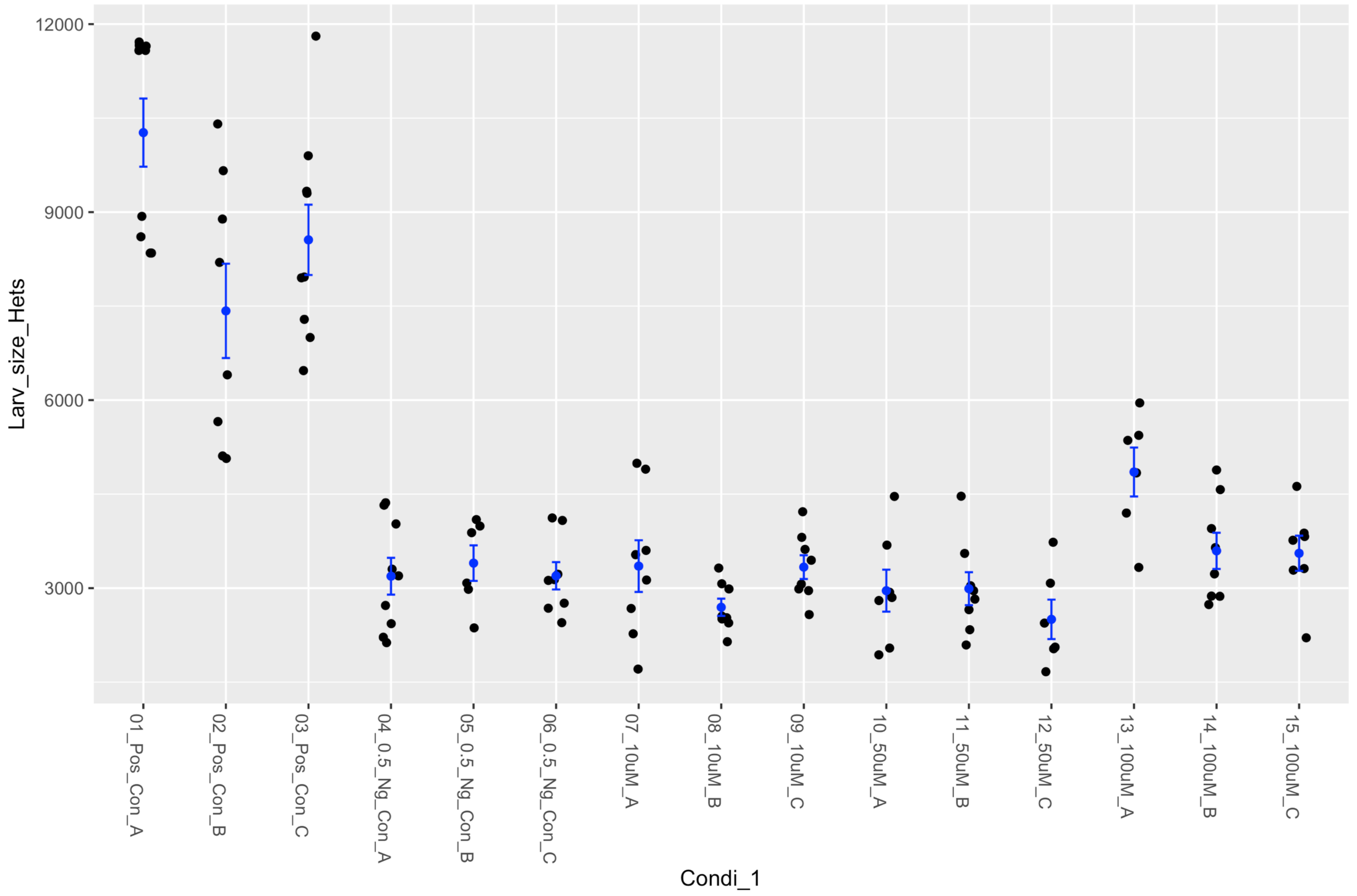

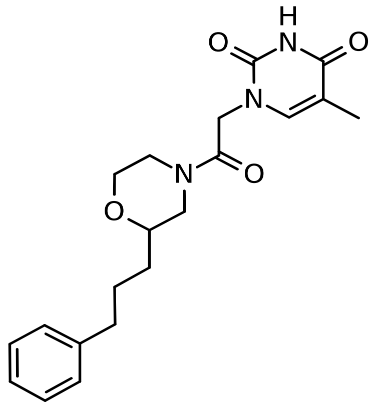

Fly Chembridge retest – Supplier ID: 35527962

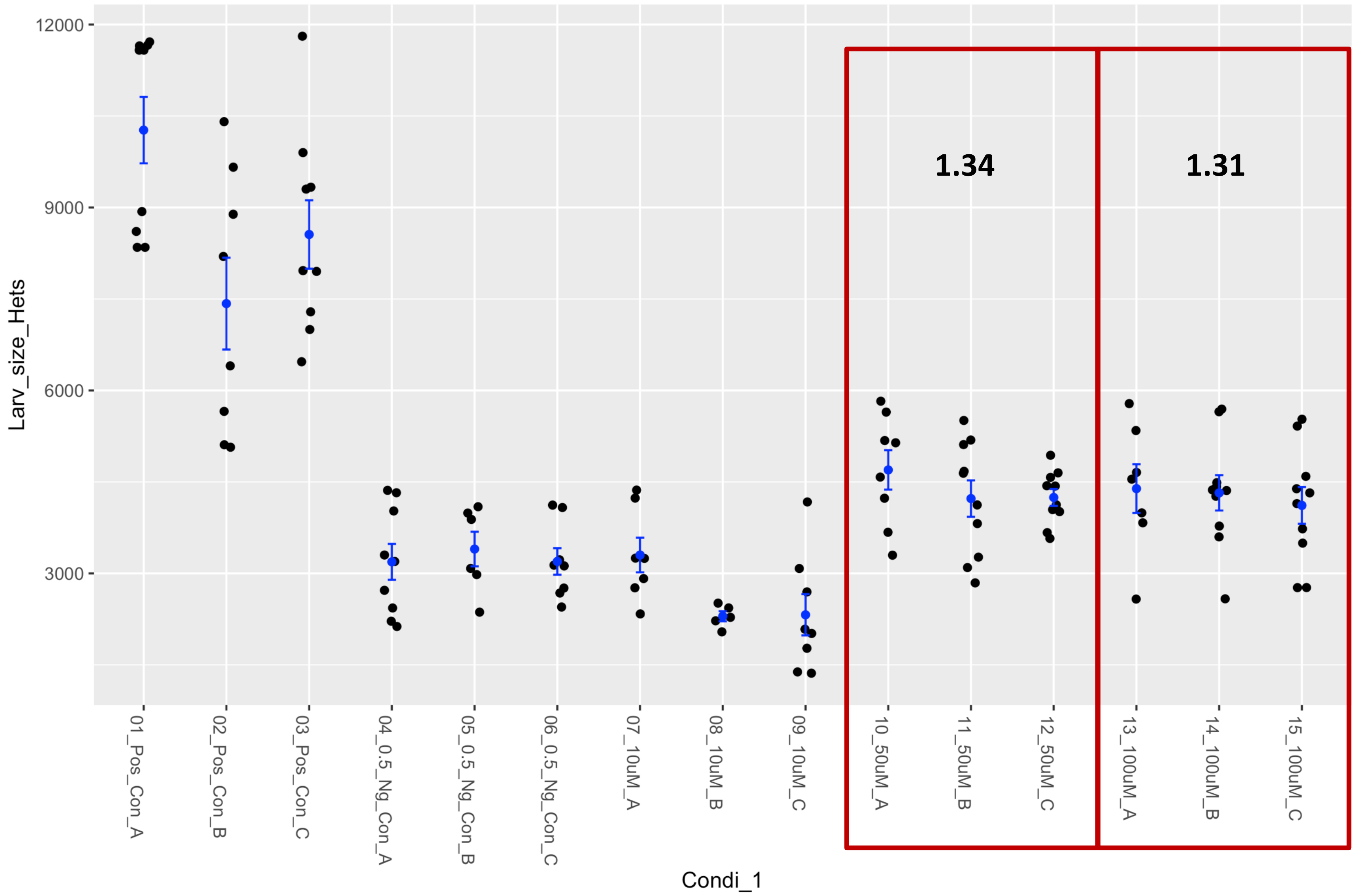

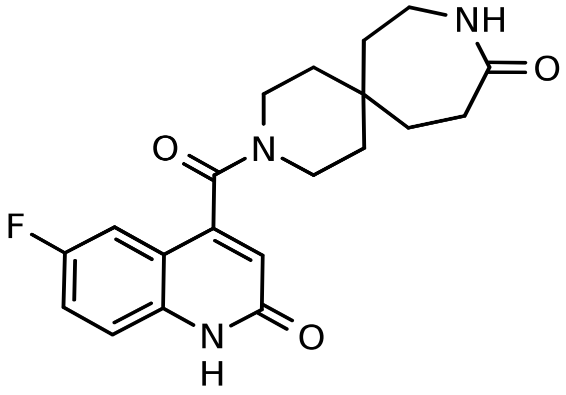

Disease Models & Mechanisms • Supplementary information

Disease Models & Mechanisms: doi:10.1242/dmm.040576: Supplementary information

Fly Chembridge retest – Supplier ID: 40966617

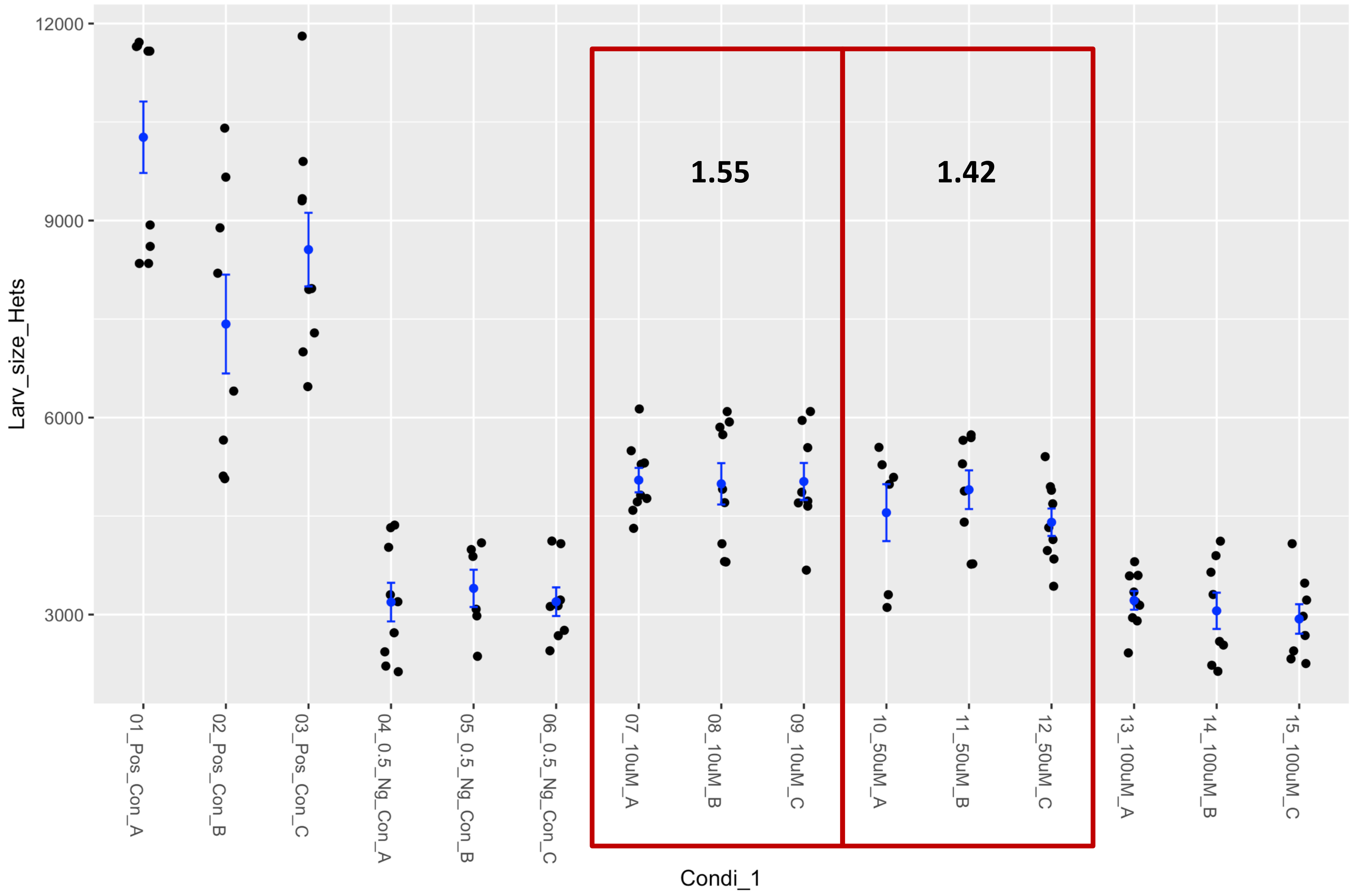

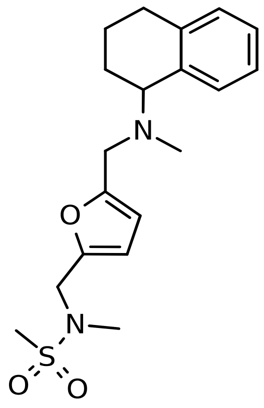

Disease Models & Mechanisms • Supplementary information

Fly Chembridge retest – Supplier ID: 89890646

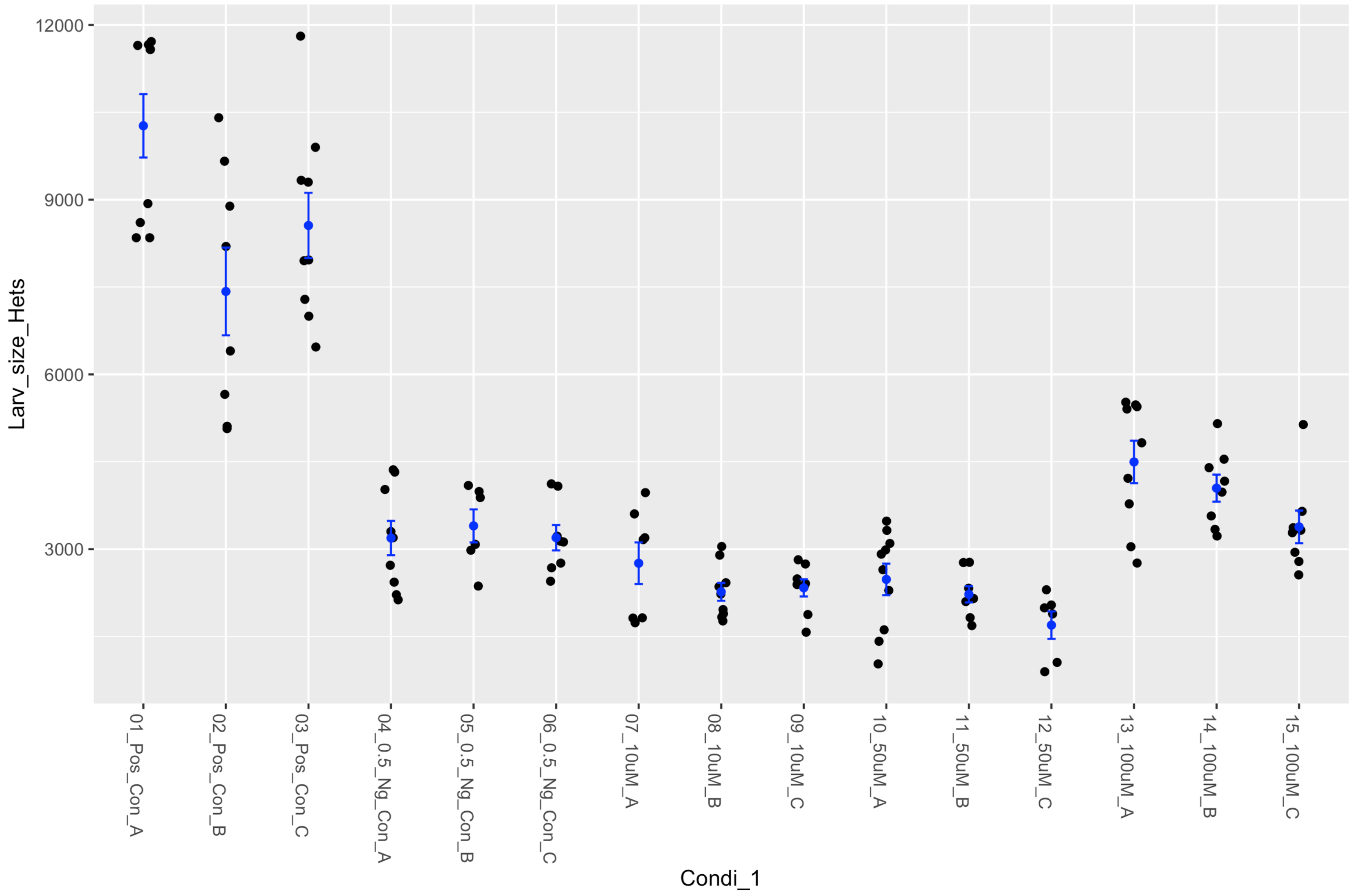

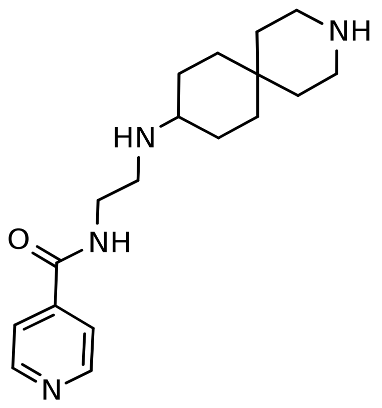

Disease Models & Mechanisms • Supplementary information

Fly Chembridge retest – Supplier ID: 9206326

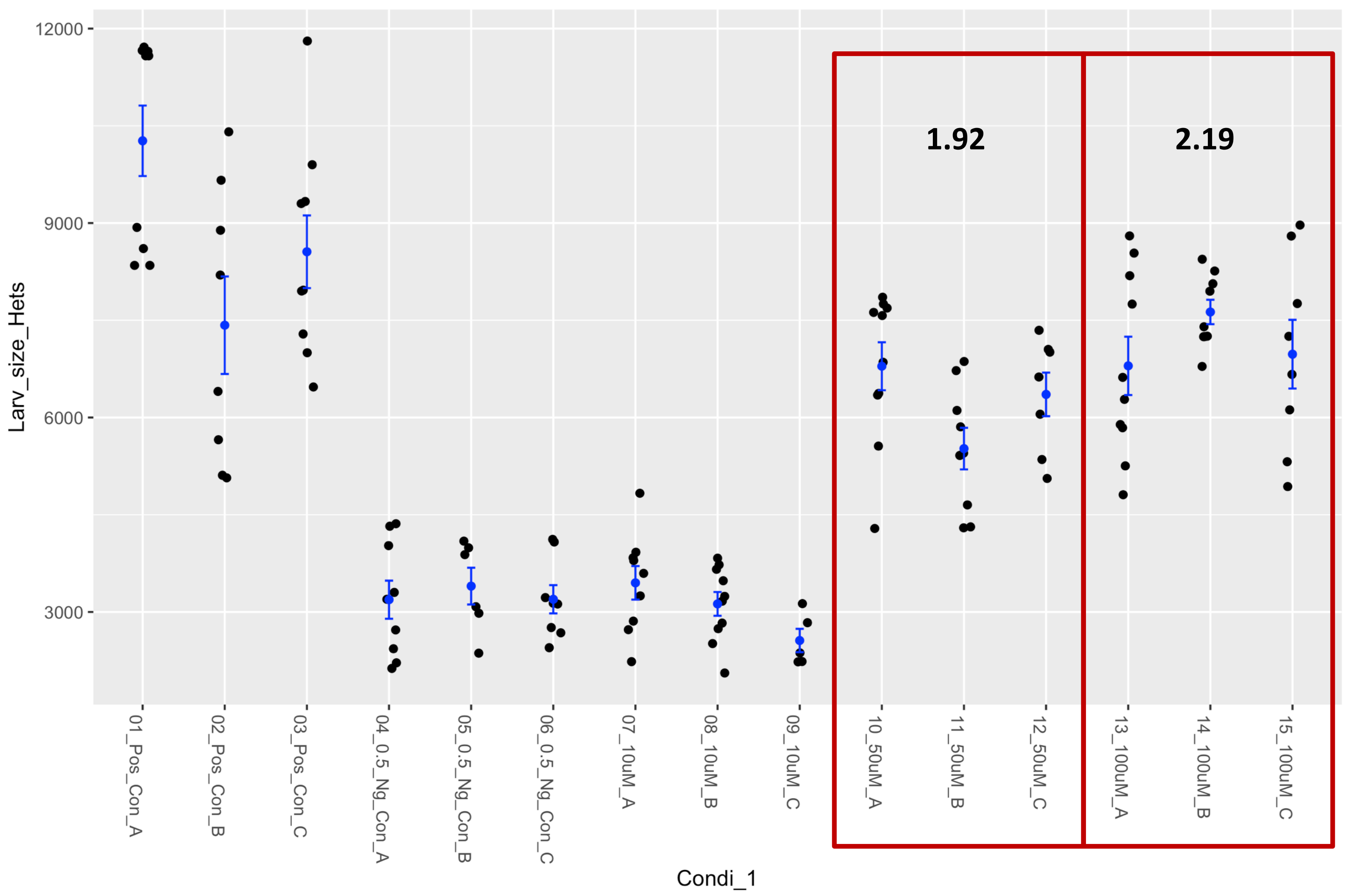

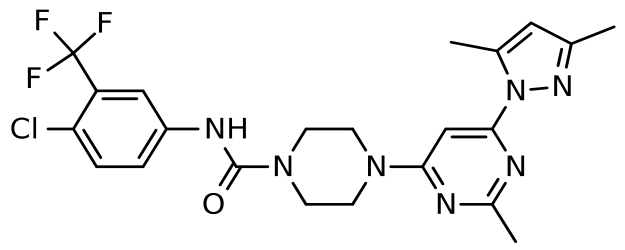

Disease Models & Mechanisms • Supplementary information

Fly Chembridge retest – Supplier ID: 9233974

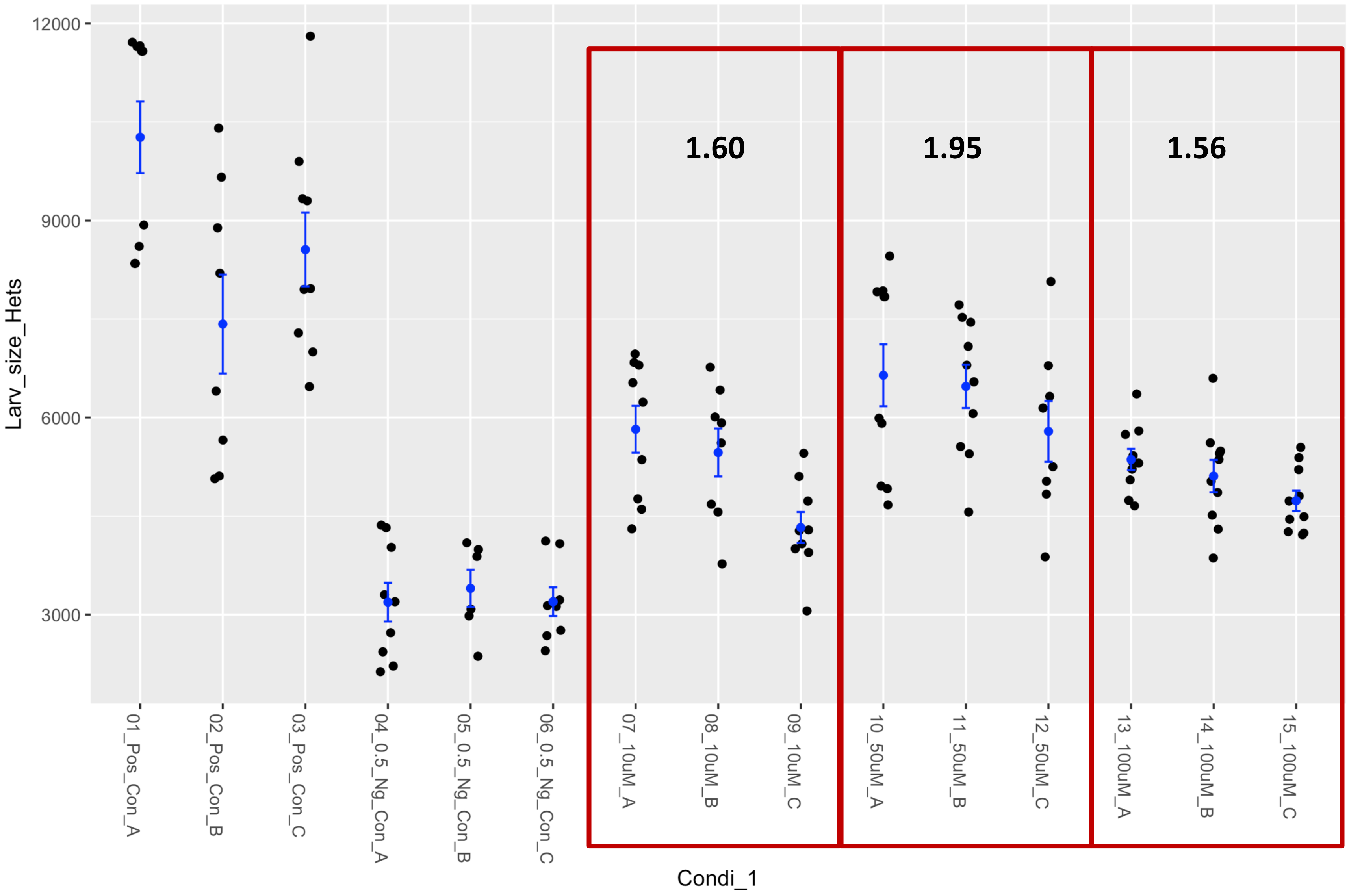

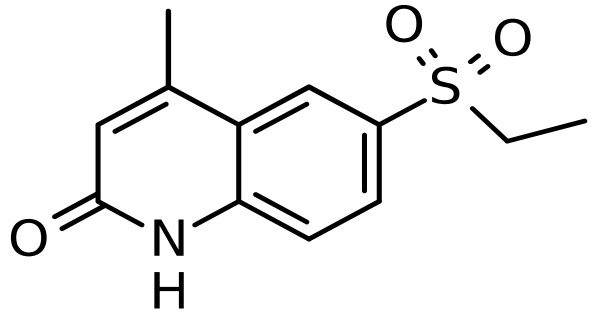

Fly Chembridge retest – Supplier ID: 9251119

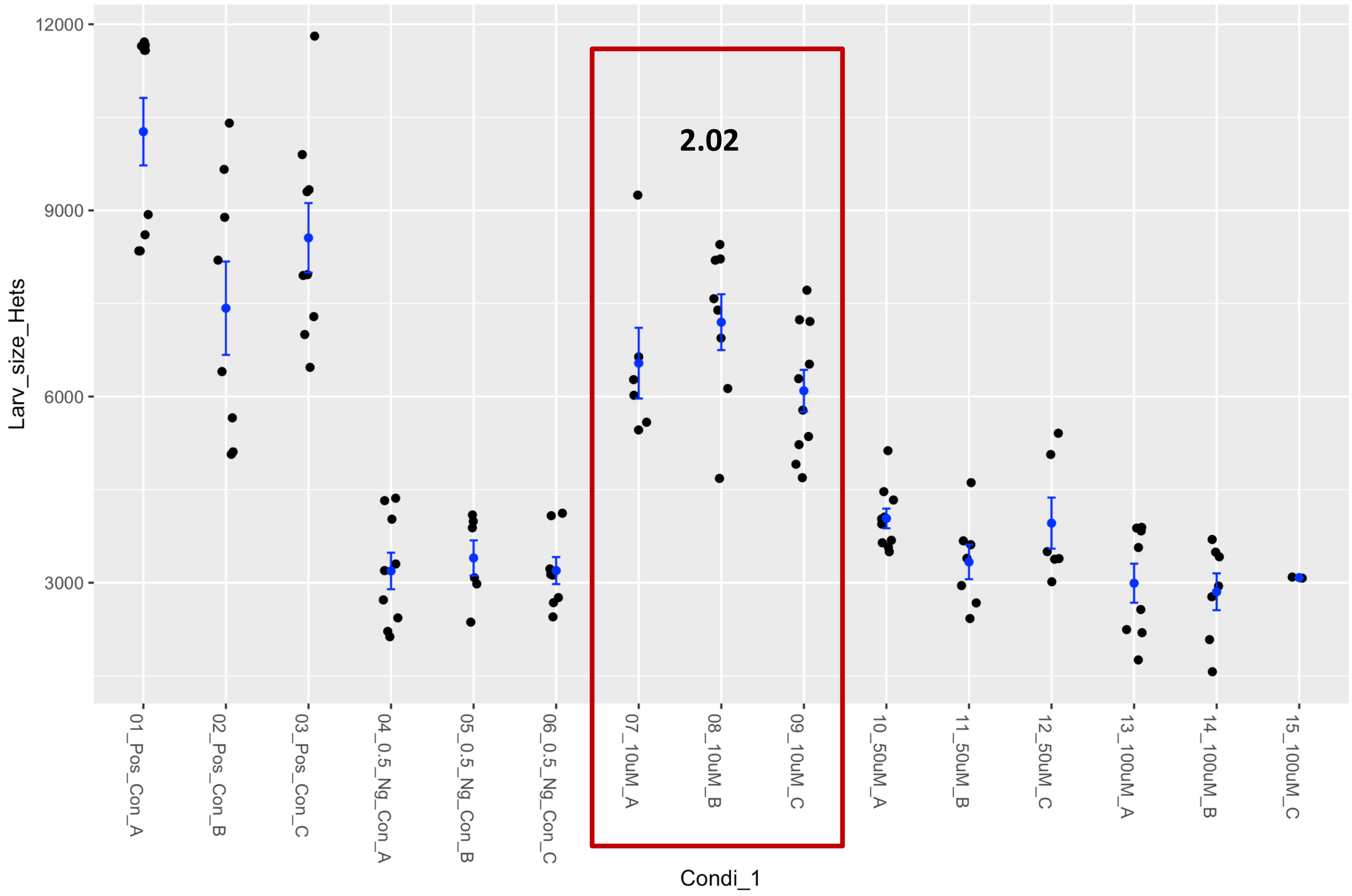

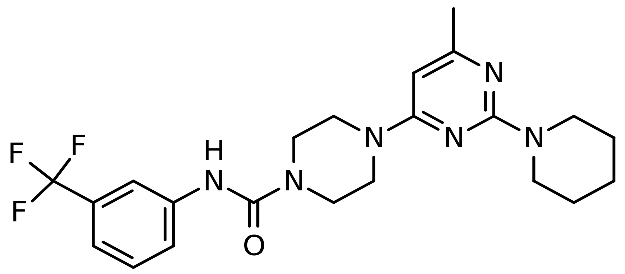

Disease Models & Mechanisms • Supplementary information

Fly Chembridge retest – Supplier ID: 9251633

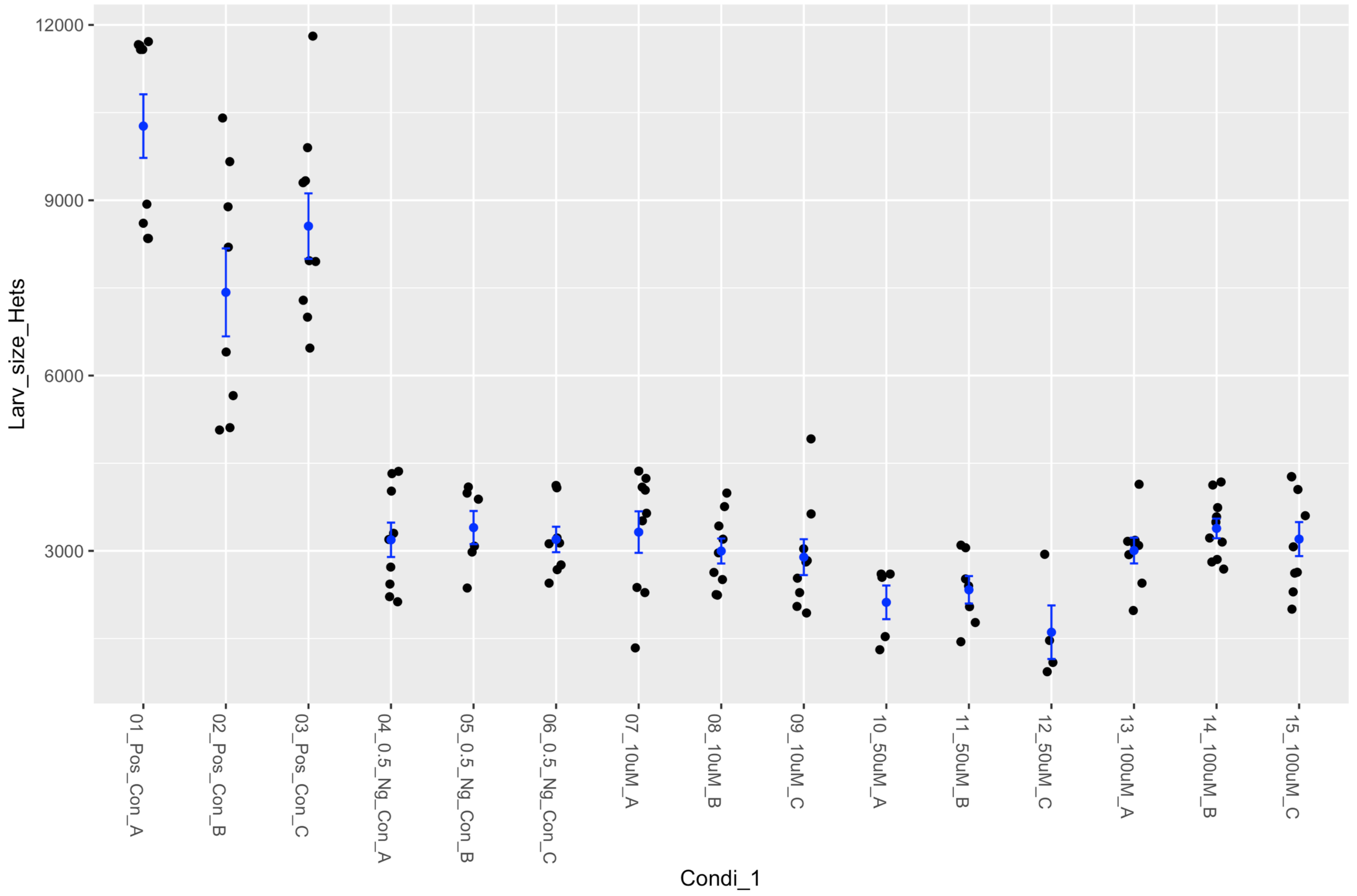

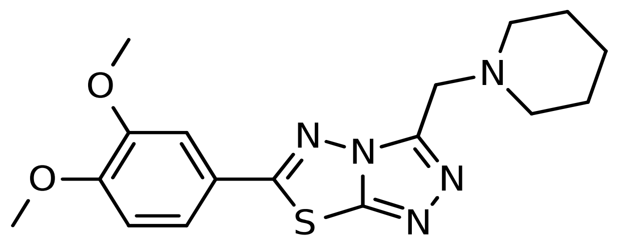

Disease Models & Mechanisms • Supplementary information

Fly Chembridge retest – Supplier ID: 9262220

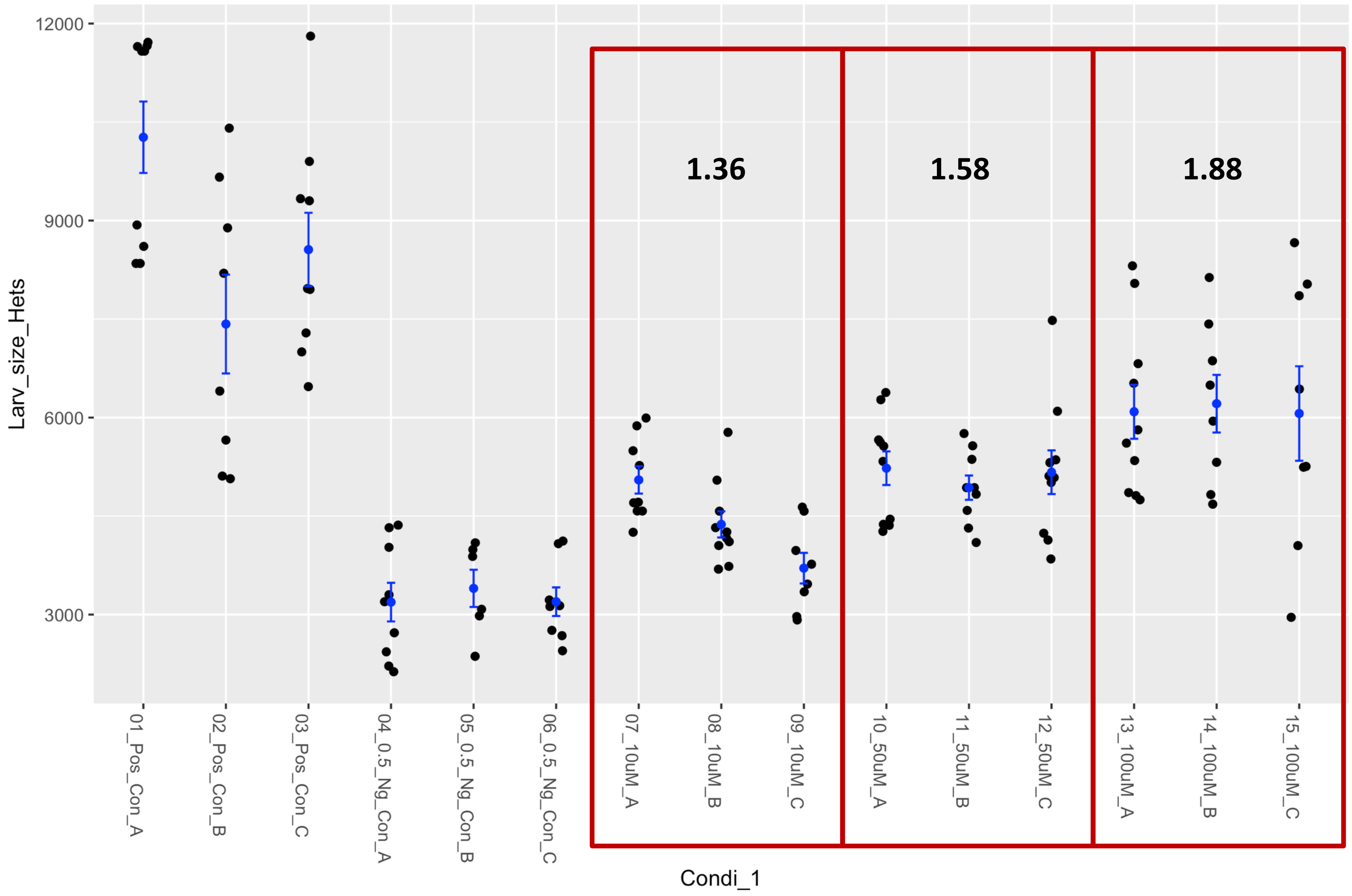

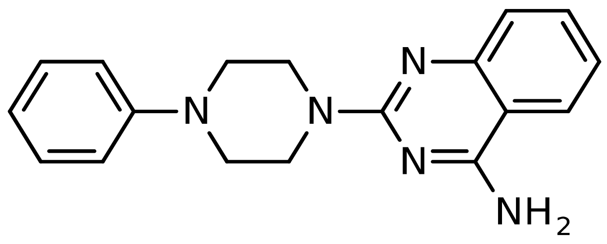

Disease Models & Mechanisms • Supplementary information

Disease Models & Mechanisms: doi:10.1242/dmm.040576: Supplementary information

Fly Chembridge retest – Supplier ID: 9262955

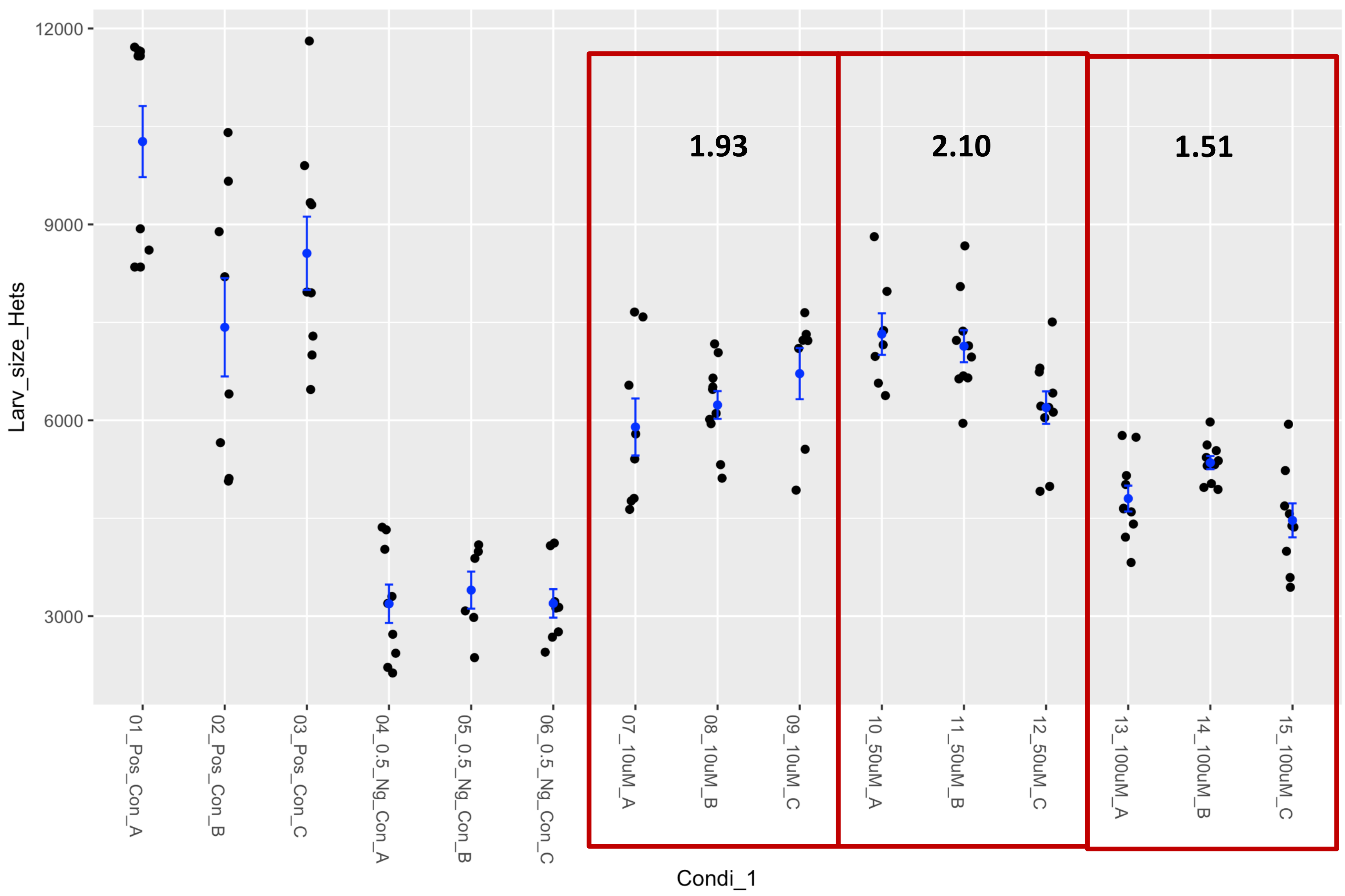

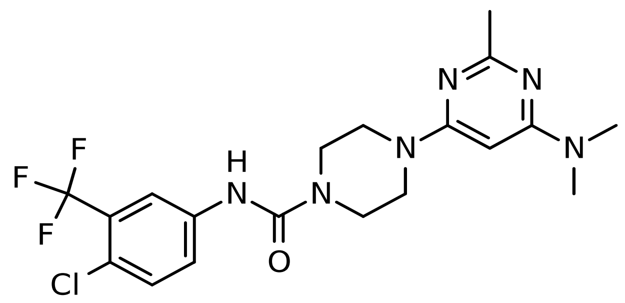

Disease Models & Mechanisms • Supplementary information

Fly Chembridge retest – Supplier ID: 9290684

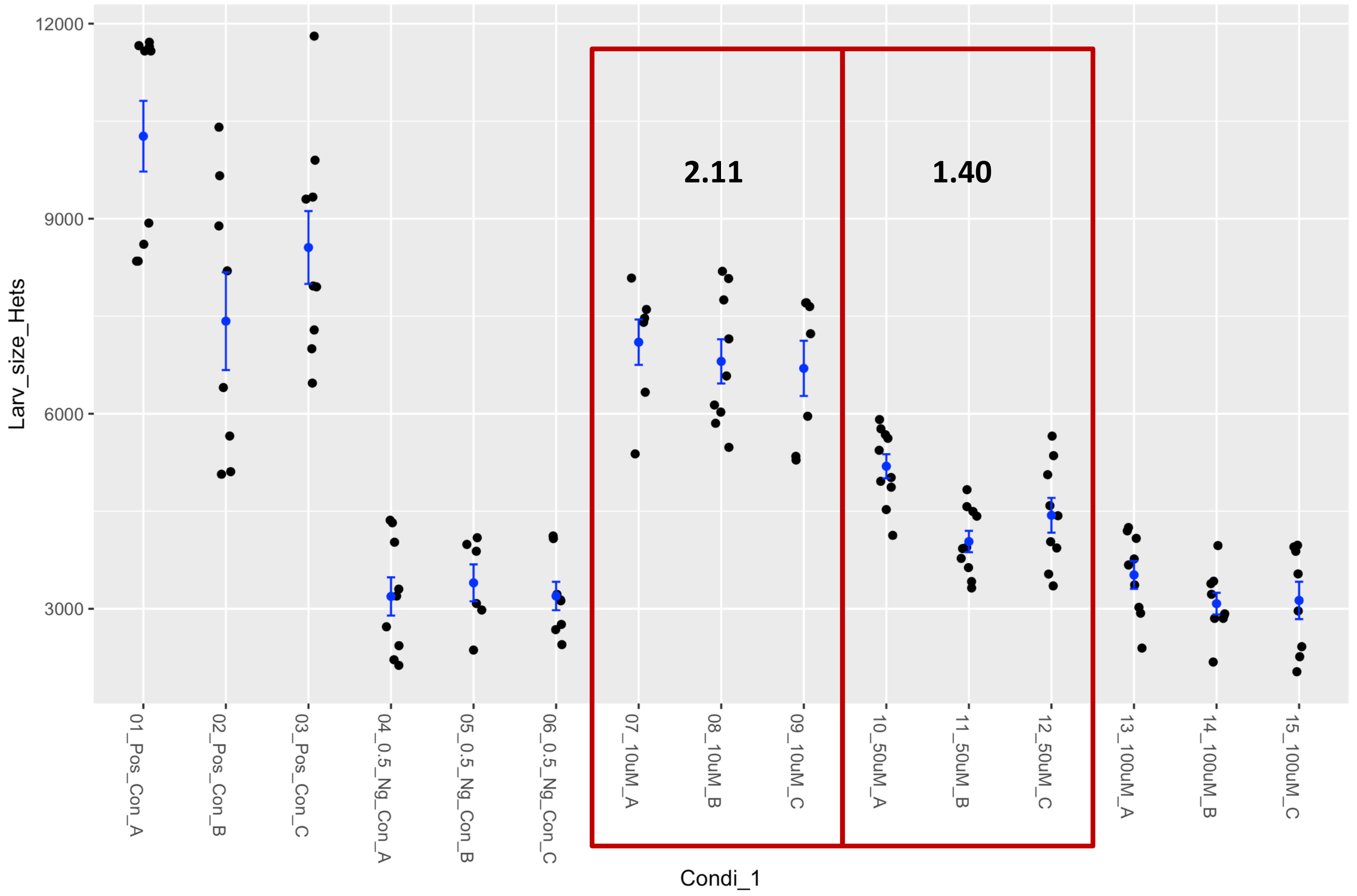

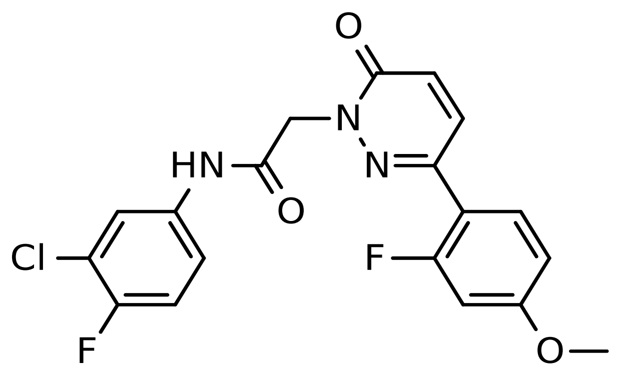

Fly retest of Chembridge Worm hits

All compounds that scored as hits in the worm *png-1* screen were cross-validated in two fly assay paradigms: rescue of *Pngl*$^{-/-}$ homozygotes and rescue of bortezomib-treated *Pngl*$^{+/-}$ heterozygotes. Red boxes indicate compounds that successfully retested. The number indicates the size ratio of compound-treated fly larvae to untreated control homozygous fly larvae.

Fly Chembridge retest – Supplier ID: 4141722

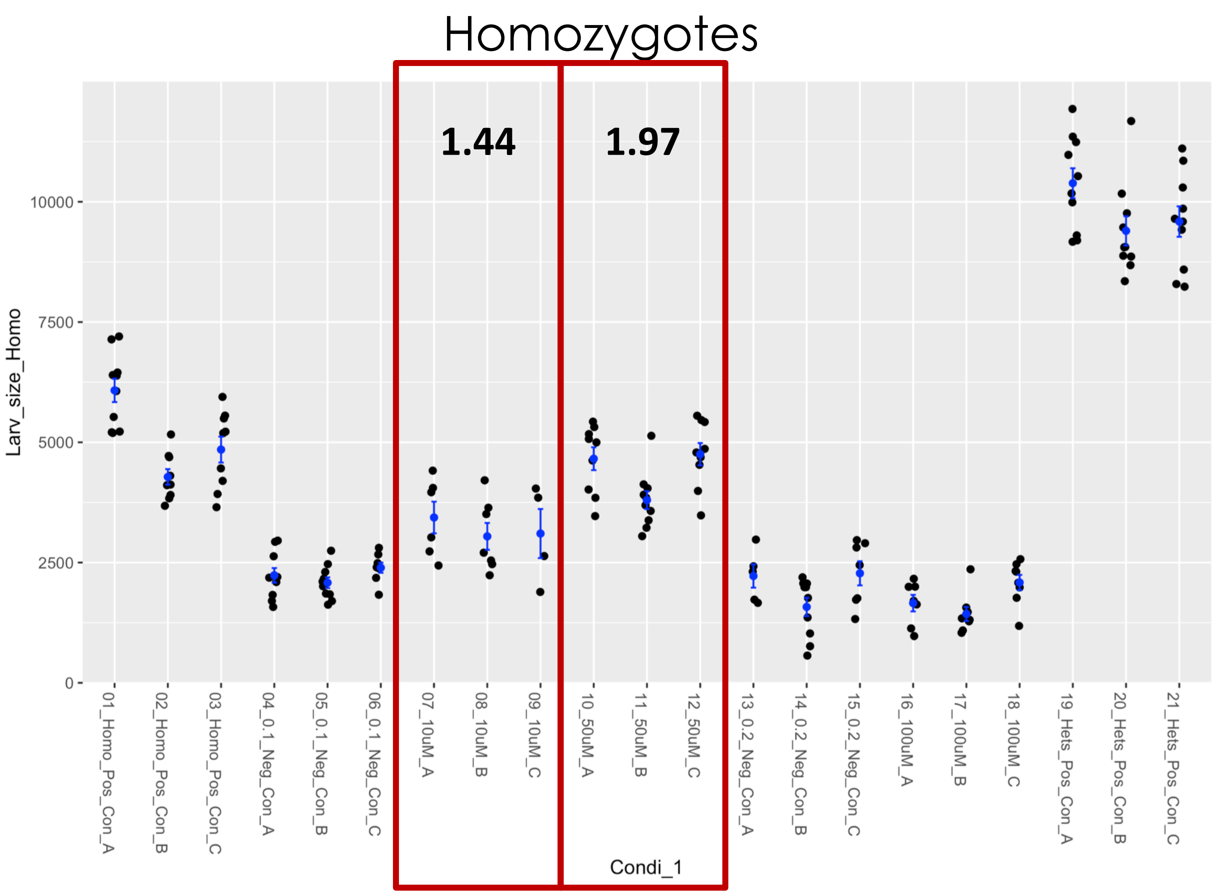

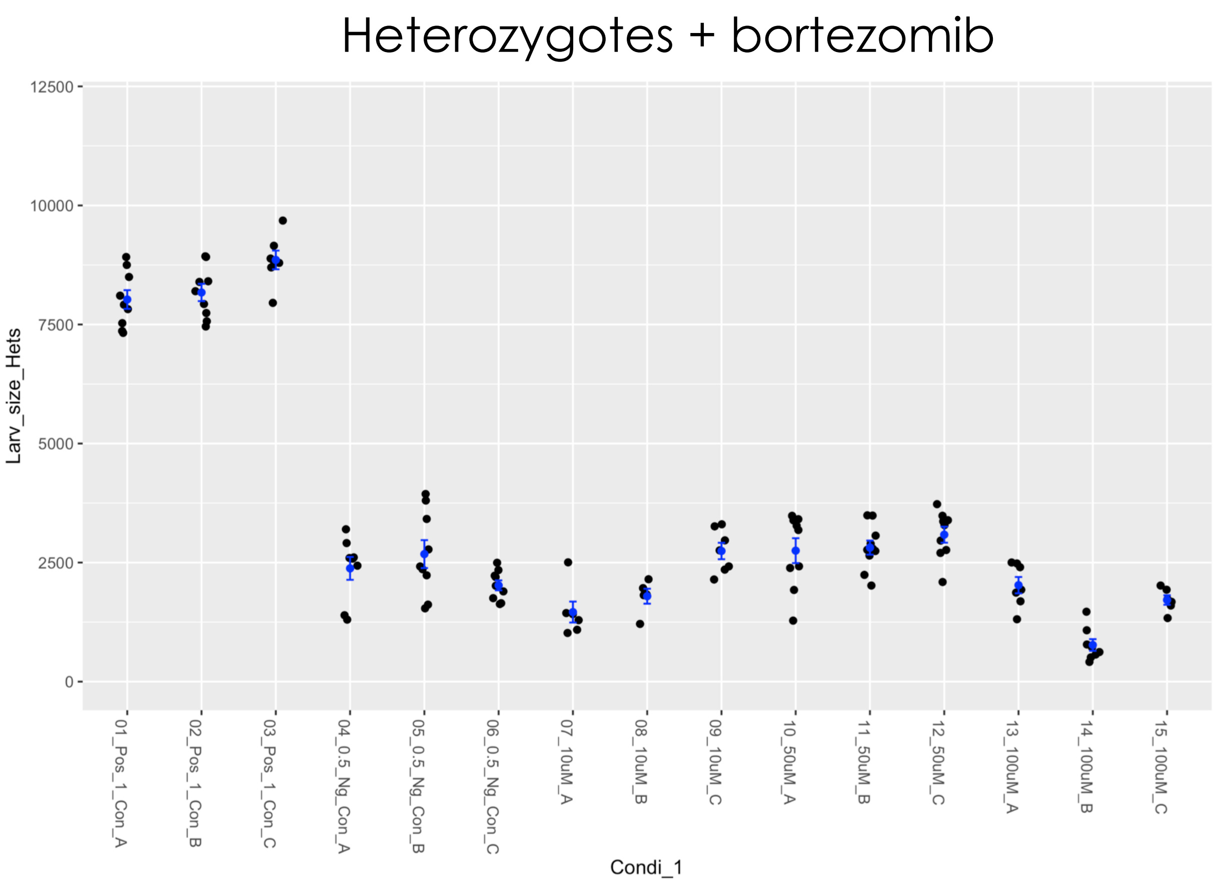

Disease Models & Mechanisms • Supplementary information

Disease Models & Mechanisms: doi:10.1242/dmm.040576: Supplementary information

Fly Chembridge retest – Supplier ID: 9223573

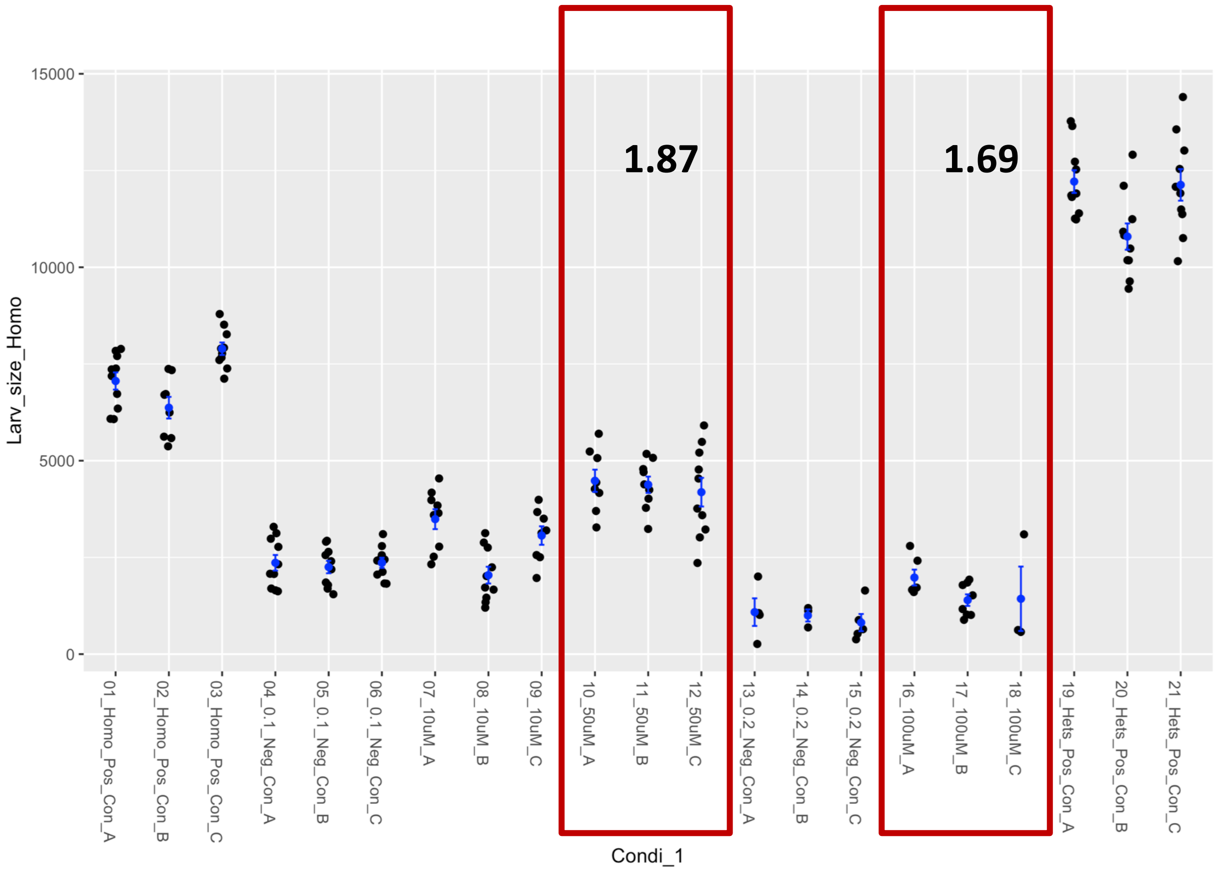

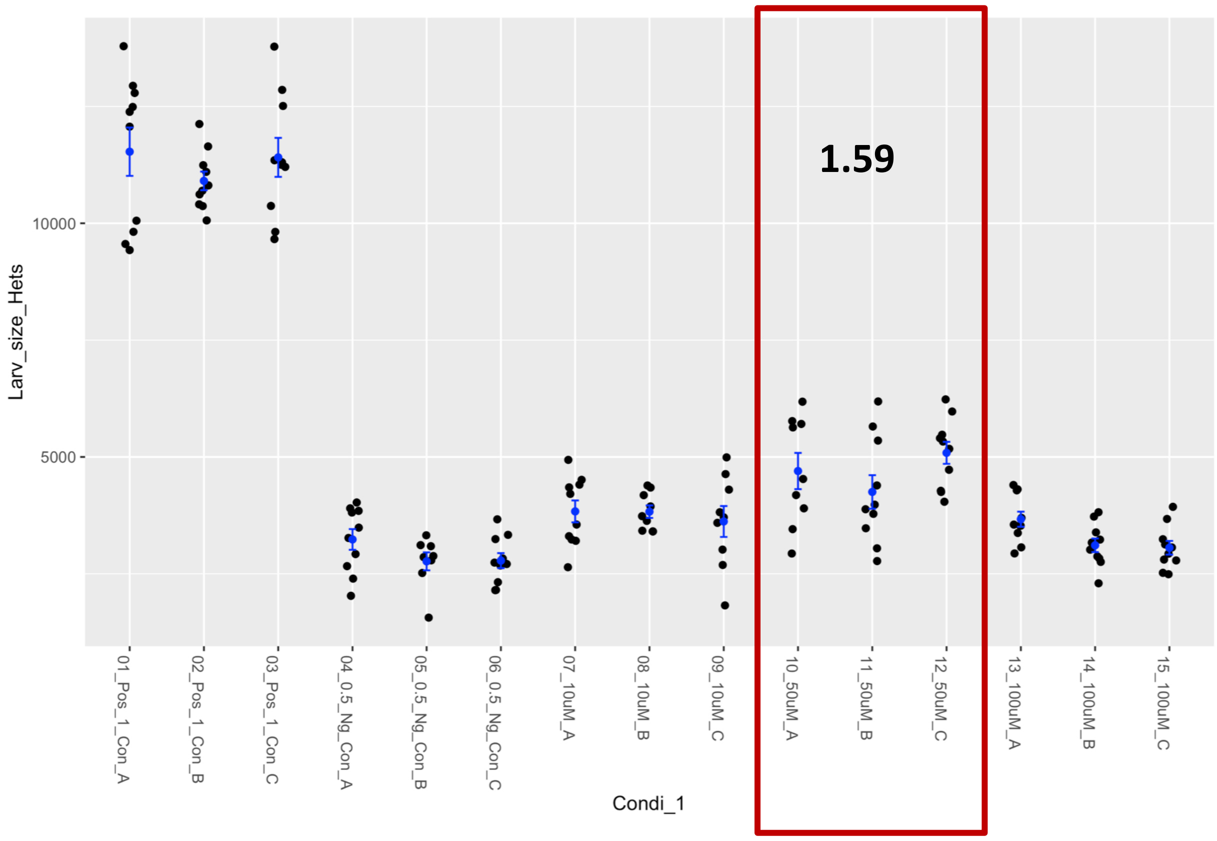

Disease Models & Mechanisms: doi:10.1242/dmm.040576: Supplementary information

Fly Chembridge retest – Supplier ID: 9268787

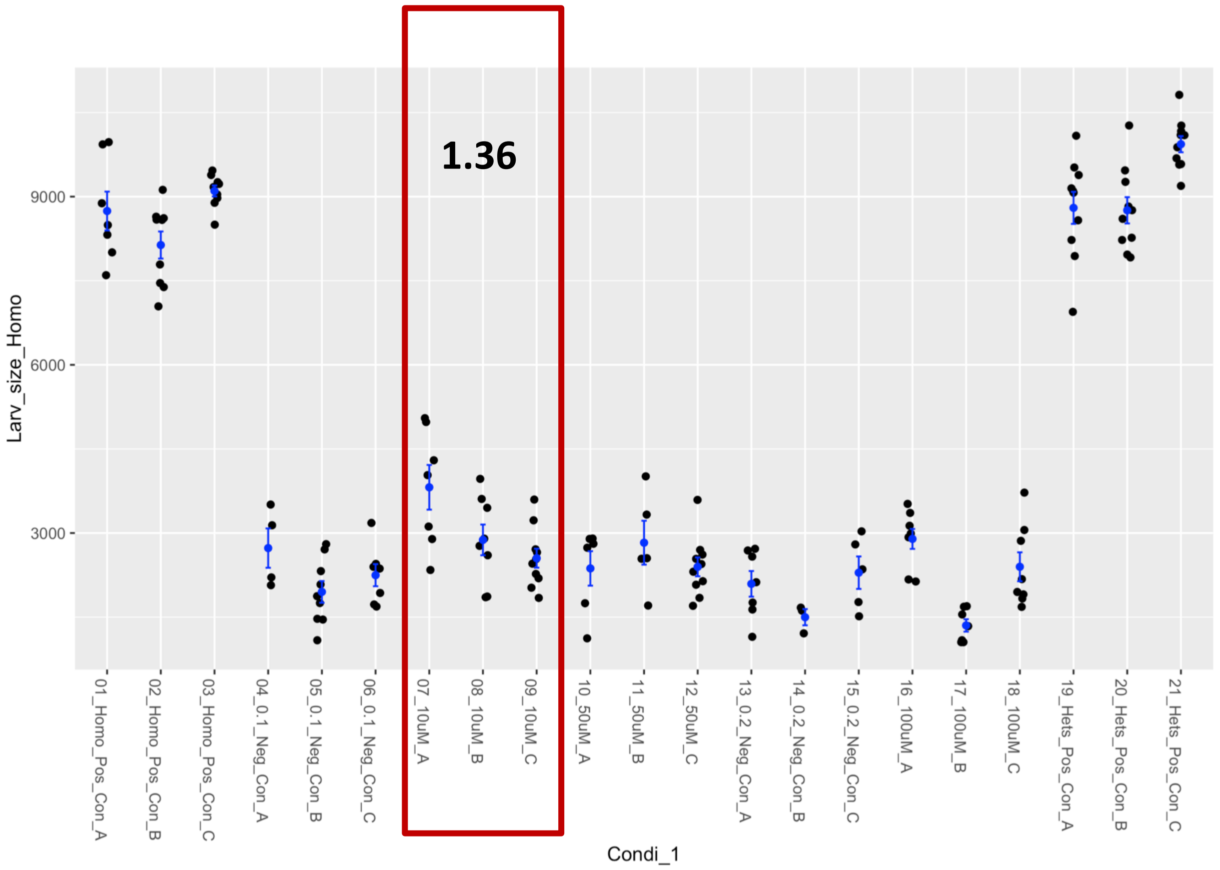

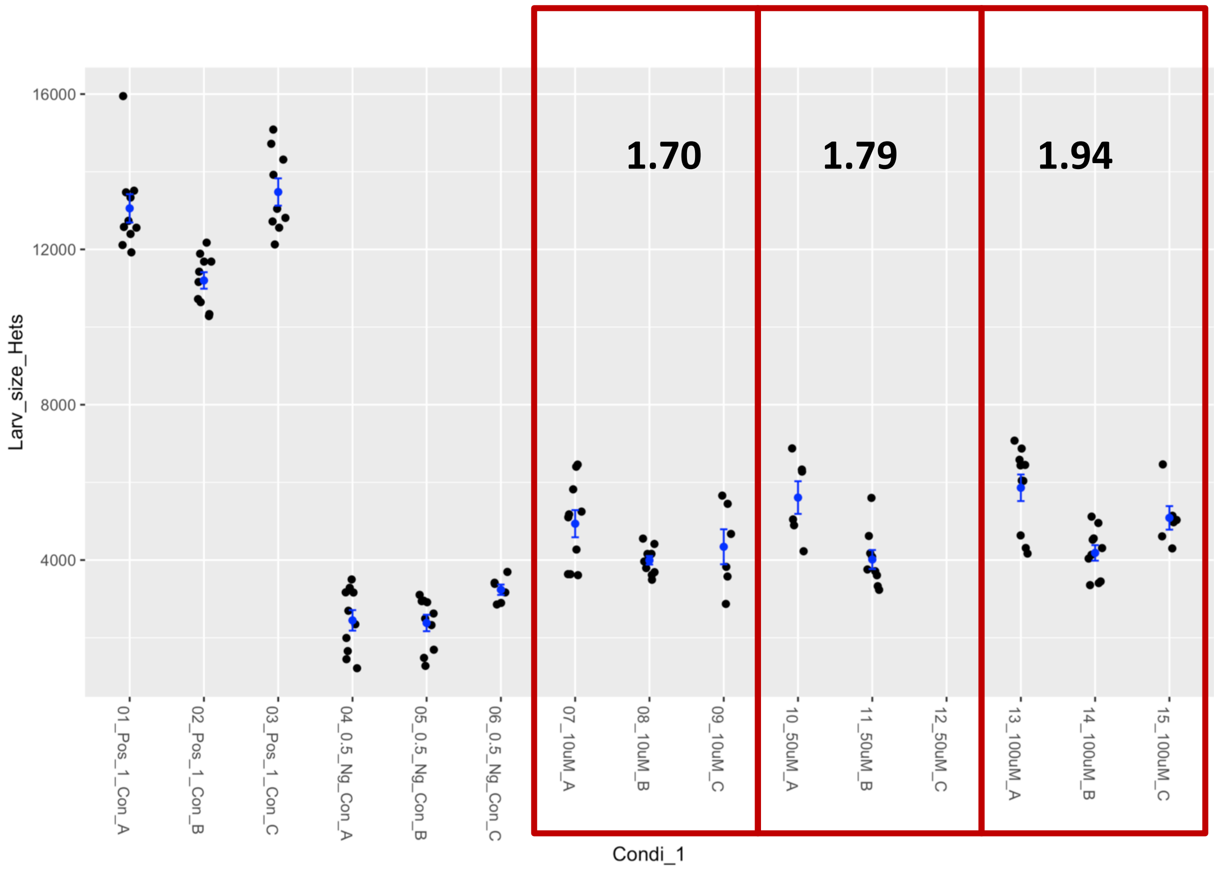

Disease Models & Mechanisms • Supplementary information

Fly Chembridge retest – Supplier ID: 9283120

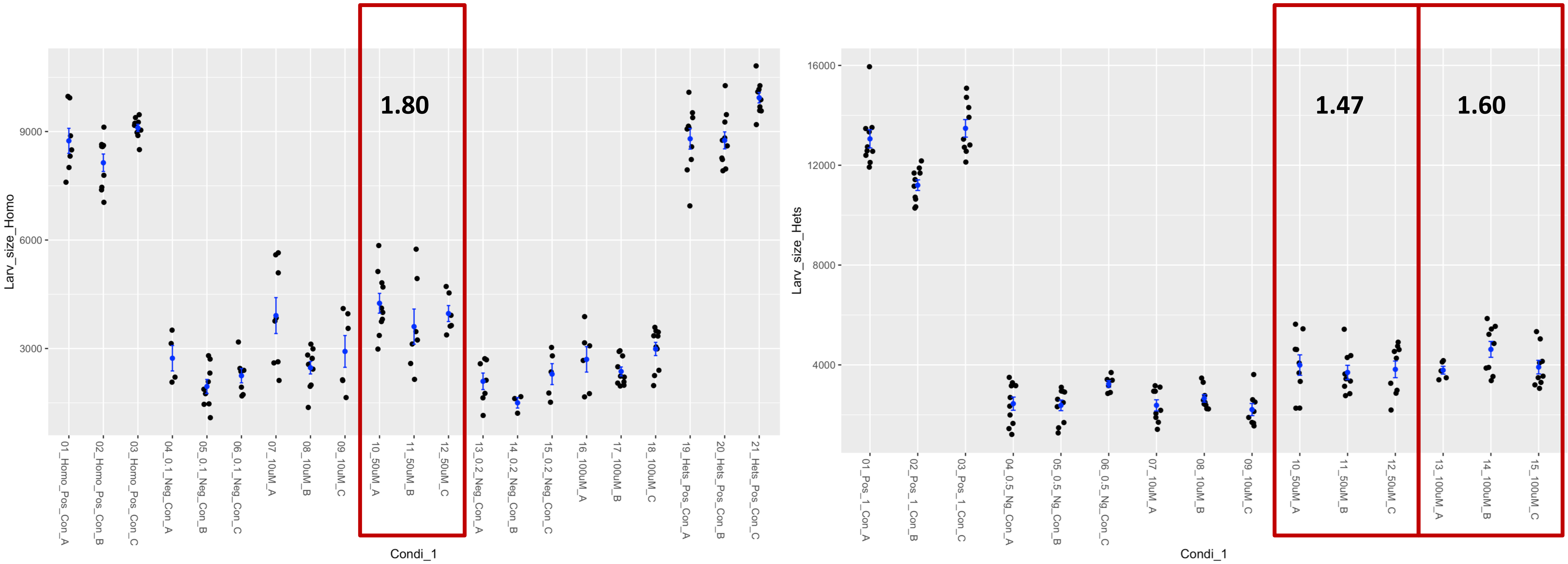

Fly Chembridge retest – Supplier ID: 9313992

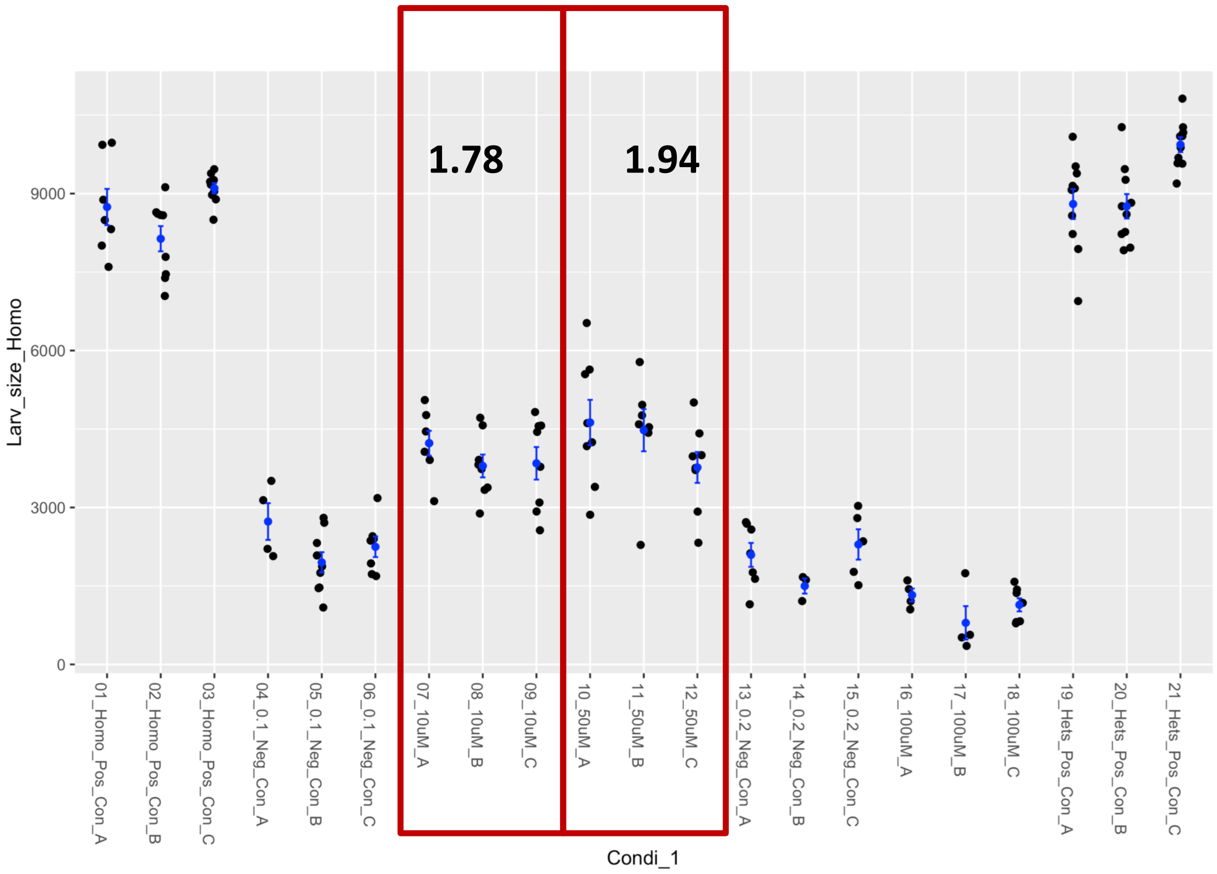

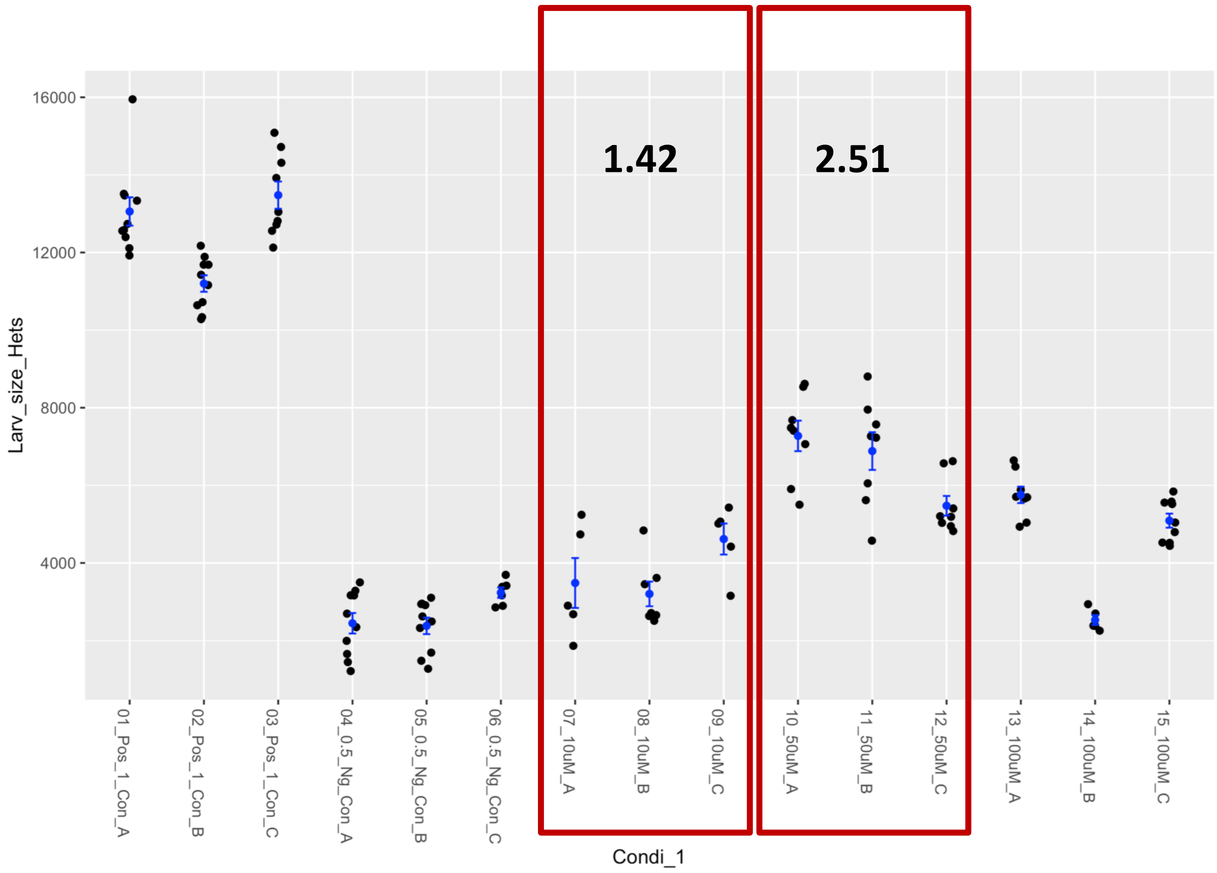

Bortezomib-only

Supplier ID	Structure
4141722	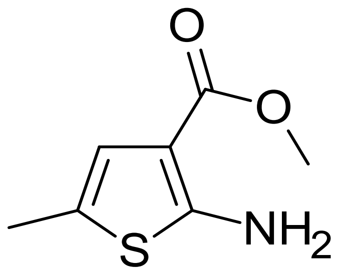
83371714	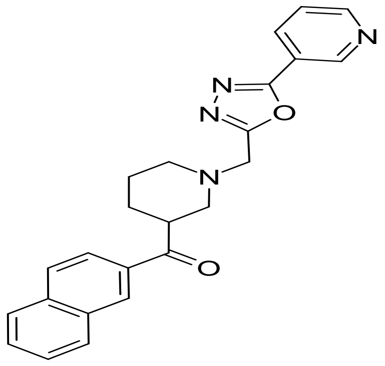
9193234	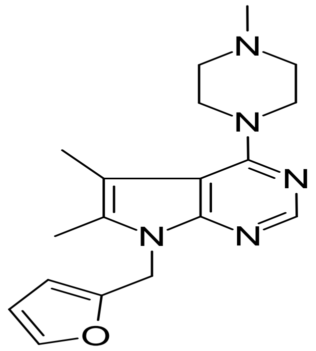
9223573	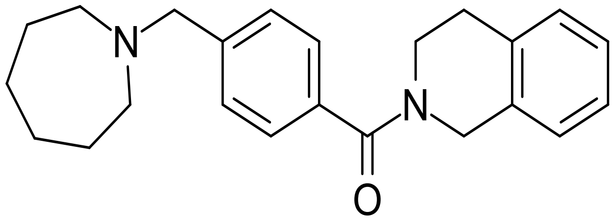

Chembridge hits from the worm *png-1* screen that only retested in the bortezomib-treated worm secondary assay, i.e., it failed to rescue carfilzomib-treated worms.

Disease Models & Mechanisms • Supplementary information

Bortezomib-only

9243480

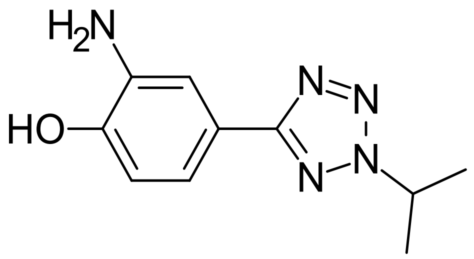

9253438

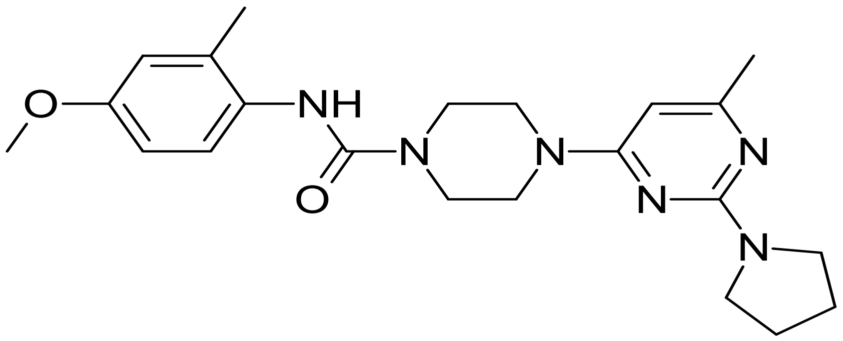

9258723

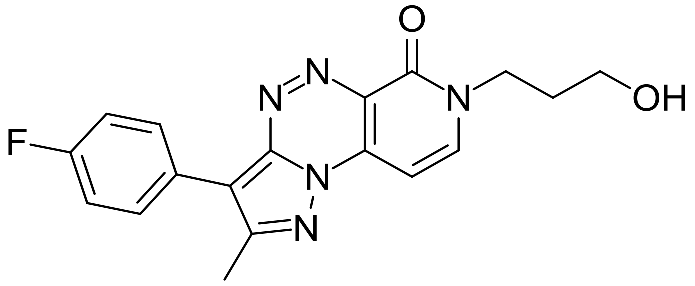

9266225

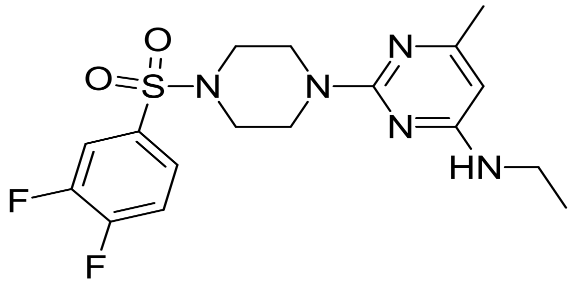

Chembridge hits from the worm *png-1* screen that only retested in the bortezomib-treated worm secondary assay, i.e., it failed to rescue carfilzomib-treated worms.

Bortezomib-only

9266617

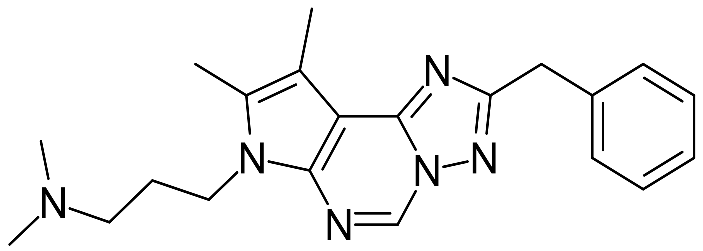

9268787

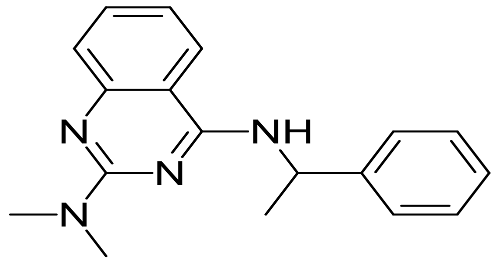

9283120

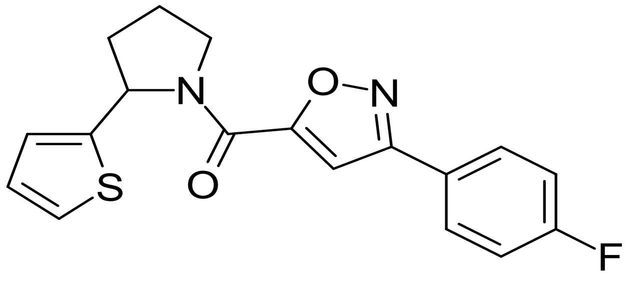

9313992

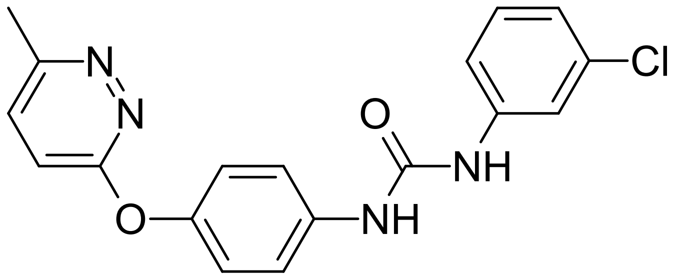

Chembridge hits from the worm *png-1* screen that only retested in the bortezomib-treated worm secondary assay, i.e., it failed to rescue carfilzomib-treated worms.

Carfilzomib-only

Supplier ID	Structure
4141722	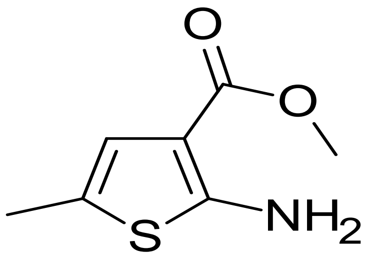
9223301	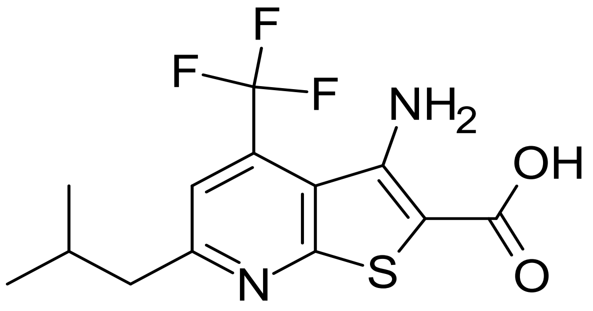
9223573	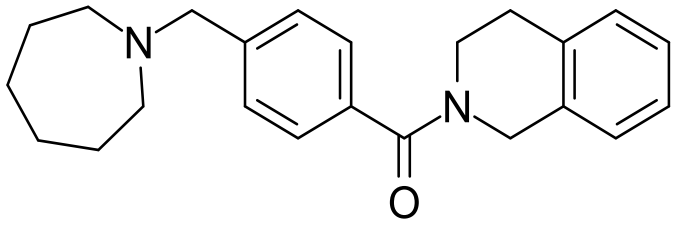
9236078	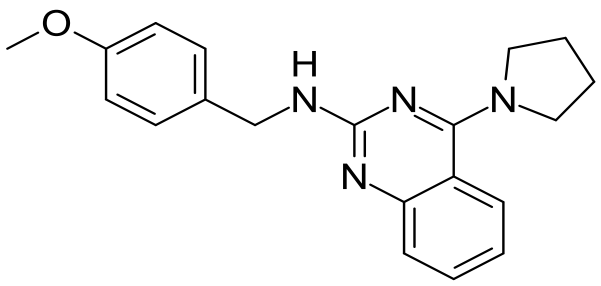

Chembridge hits from the worm *png-1* screen that only retested in the carfilzomib-treated worm secondary assay, i.e., failed to rescue in bortezomib-treated worms. These compounds are likely weak or borderline hits in bortezomib.

Disease Models & Mechanisms • Supplementary information

Carfilzomib-only

9256157

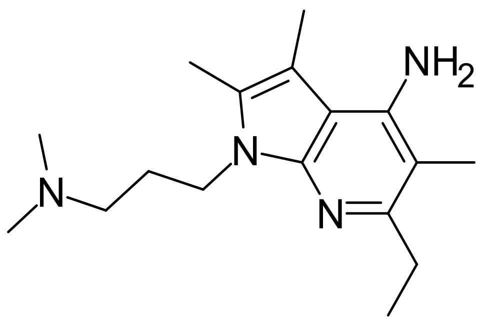

9257006

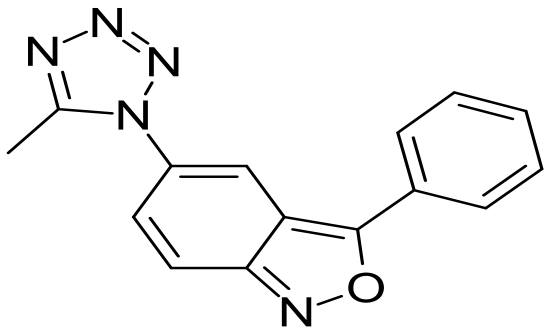

9260754

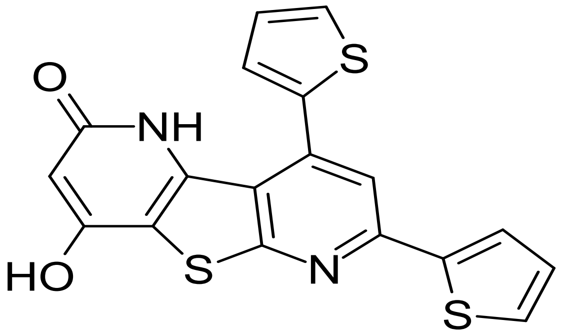

9268787

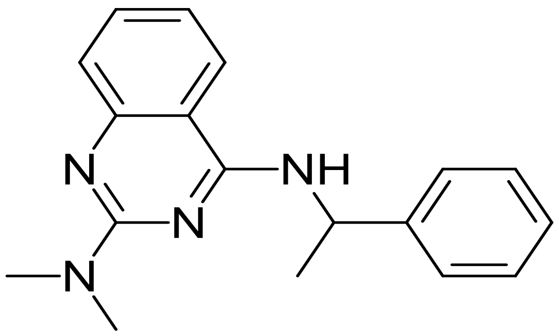

Chembridge hits from the worm *png-1* screen that only retested in the carfilzomib-treated worm secondary assay, i.e., failed to rescue in bortezomib-treated worms. These compounds are likely weak or borderline hits in bortezomib.

Disease Models & Mechanisms • Supplementary information

Carfilzomib-only

9283120

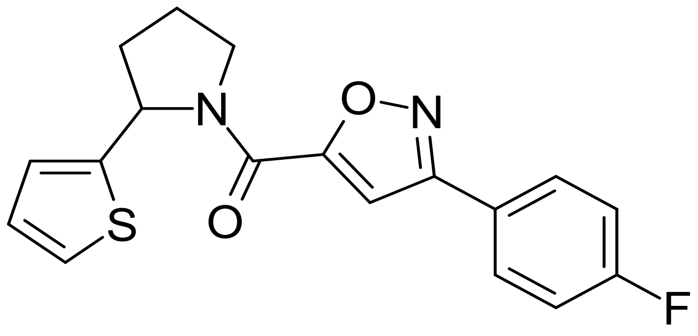

9313992

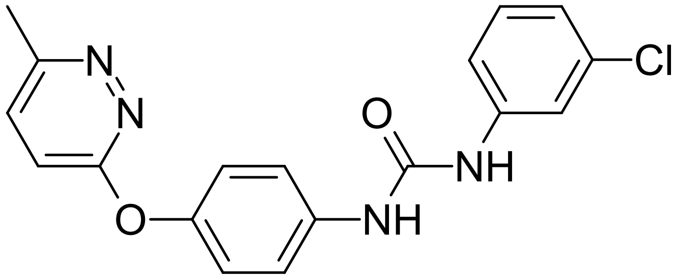

Chembridge hits from the worm *png-1* screen that only retested in the carfilzomib-treated worm secondary assay, i.e., failed to rescue in bortezomib-treated worms. These compounds are likely weak or borderline hits in bortezomib.

Disease Models & Mechanisms • Supplementary information

Bortezomib and carfilzomib

Supplier ID	Structure
4141722	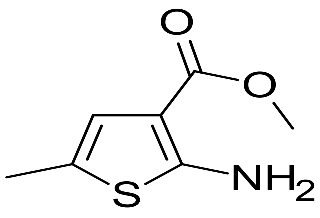
9223573	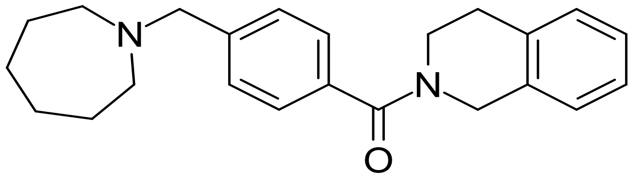
9268787	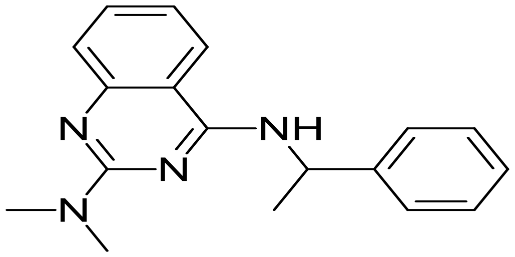
9283120	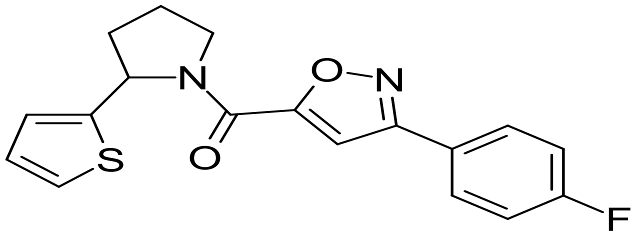
9313992	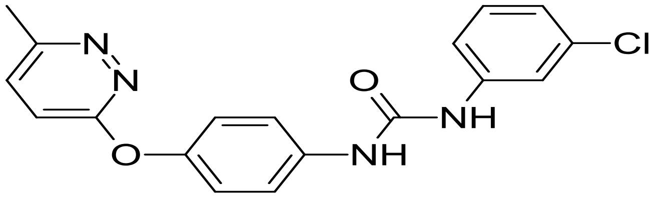

Chembridge hits from the worm *png-1* screen that retested in both bortezomib- and carfilzomib-treated worm secondary assays.

Disease Models & Mechanisms • Supplementary information
